# Autoinflammatory Keratinization Disease With Hepatitis and Autism Reveals Roles for JAK1 Kinase Hyperactivity in Autoinflammation

**DOI:** 10.3389/fimmu.2021.737747

**Published:** 2022-01-03

**Authors:** Takuya Takeichi, John Y. W. Lee, Yusuke Okuno, Yuki Miyasaka, Yuya Murase, Takenori Yoshikawa, Kana Tanahashi, Emi Nishida, Tatsuya Okamoto, Komei Ito, Yoshinao Muro, Kazumitsu Sugiura, Tamio Ohno, John A. McGrath, Masashi Akiyama

**Affiliations:** ^1^ Department of Dermatology, Nagoya University Graduate School of Medicine, Nagoya, Japan; ^2^ St John’s Institute of Dermatology, King’s College London, London, United Kingdom; ^3^ Medical Genomics Center, Nagoya University Hospital, Nagoya, Japan; ^4^ Department of Pediatrics, Nagoya University Graduate School of Medicine, Nagoya, Japan; ^5^ Department of Virology, Nagoya City University Graduate School of Medical Sciences, Nagoya, Japan; ^6^ Division of Experimental Animals, Nagoya University Graduate School of Medicine, Nagoya, Japan; ^7^ Department of Geriatric and Environmental Dermatology, Nagoya City University Graduate School of Medical Sciences, Nagoya, Japan; ^8^ Division of Pediatric Surgery, Department of Surgery, Graduate School of Medicine, Kyoto University, Kyoto, Japan; ^9^ Department of Allergology, Aichi Children’s Health and Medical Center, Obu, Japan; ^10^ Department of Dermatology, Fujita Health University School of Medicine, Toyoake, Japan

**Keywords:** inflammation, brain, liver, skin, STAT

## Abstract

Heterozygous mutations in *JAK1* which result in JAK-STAT hyperactivity have been implicated in an autosomal dominant disorder that features multi-organ immune dysregulation. This study identifies another previously unreported heterozygous missense *JAK1* mutation, H596D, in an individual with a unique autoinflammatory keratinization disease associated with early-onset liver dysfunction and autism. Using CRISPR-Cas9 gene targeting, we generated mice with an identical *Jak1* knock-in missense mutation (*Jak1* ^H595D/+;I596I/+;Y597Y/+^ mice) that recapitulated key aspects of the human phenotype. RNA sequencing of samples isolated from the *Jak1* ^H595D/+;I596I/+;Y597Y/+^ mice revealed the upregulation of genes associated with the hyperactivation of tyrosine kinases and NF-κB signaling. Interestingly, there was a strong correlation between genes downregulated in *Jak1* ^H595D/+;I596I/+;Y597Y/+^ mice and those downregulated in the brain of model mice with 22q11.2 deletion syndrome that showed cognitive and behavioral deficits, such as autism spectrum disorders. Our findings expand the phenotypic spectrum of *JAK1*-associated disease and underscore how JAK1 dysfunction contributes to this autoinflammatory disorder.

## Introduction

The Janus kinase/signal transducers and activators of transcription (JAK/STAT) pathway plays an integral role in the regulation of inflammatory processes by relaying responses between surface receptors and cytokines such as interferons (IFNs) and interleukins (ILs) (1). The binding of ligands to their cognate receptors leads to the activation of JAKs, which subsequently phosphorylate each other and associated receptors. This interaction activates STAT proteins, which can trigger downstream signaling axes or can function as transcription factors themselves (2). JAK1 is a ubiquitous tyrosine kinase that is crucial for signaling by cytokines such as IFNα/β, IFNγ, IL-2, IL-6, and IL-10 (2).

Somatic gain-of-function mutations in *JAK1* have been identified in malignancies such as acute lymphoblastic leukemia, acute myeloid leukemia, and solid-organ cancers (3–5). More recently, heterozygous mutations in *JAK1* that result in JAK-STAT hyperactivity have been implicated in an autosomal dominant disorder that features multi-organ immune dysregulation (MIM 618999) (6, 7). In this study, we identify a further previously unreported heterozygous missense mutation in *JAK1* in an individual with inflammatory skin changes of a unique autoinflammatory keratinization disease (AiKD) associated with early-onset liver dysfunction and autism. Based on supporting analyses using patient samples and a knock-in (KI) mouse model generated by CRISPR-Cas9 editing, we present further evidence that activating mutations in *JAK1* are responsible for this systemic autoinflammatory phenotype.

## Materials and Methods

### Whole-Exome Sequencing

Blood samples from the patient and her parents were obtained for genetic analysis in accordance with the Declaration of Helsinki. Following informed consent, genomic DNA from the proband was used for whole-exome sequencing. Exome capture was performed by in-solution hybridization using SureSelect Human All Exon V6 bait (Agilent Technologies, Santa Clara, CA, USA). Massively parallel sequencing was performed with the Illumina HiSeq2500 platform with 150-bp paired end-reads (Illumina, San Diego, CA). The reads produced were aligned to the hg19 reference human genome using Burrows-Wheeler Aligner software with default parameters and a –mem option (8). PCR duplicates were removed using MarkDuplicates in Picard tools (https://broadinstitute.github.io/picard/). Candidate variants were called using VarScan2 (http://massgenomics.org/varscan) and were annotated using ANNOVAR (http://annovar.openbioinformatics.org/). Common variants defined by >1% minor allele frequency in ExAC (http://exac.broadinstitute.org/), 1000 genomes (http://www.1000genomes.org/), or ESP6500 (http://evs.gs.washington.edu/EVS/) were excluded from analysis.

### Generation of the *Jak1* Knock-in Mice

C57BL/6J mice was purchased from Japan SLC (Hamamatsu, Japan). All mice were fed a commercial CE-2 diet (CREA Japan, Tokyo) and had *ad libitum* access to water. The mice were bred in a pathogen-free facility at the Institute for Laboratory Animal Research, Graduate School of Medicine, Nagoya University, and maintained under a controlled temperature of 23 ± 1°C, a humidity of 55 ± 10%, and a light cycle of 12-hour light (from 09:00 to 21:00)/12-hour dark (from 21:00 to 09:00). Animal care and all experimental procedures were approved by the Animal Experiment Committee, Graduate School of Medicine, Nagoya University, and were conducted according to the Regulations on Animal Experiments of Nagoya University.

Targeted disruption of the *Jak1* gene on a C57BL/6J background was carried out using the CRISPR/Cas9 method as previously described (9). CRISPR RNA (crRNA, 5’- CAA GAA CAC ATA TCT ATT CT-3’) targeting exon 13 was designed using the CRISPOR website (10). The designed crRNA and trans-activating crRNA (tracrRNA) (Genome CraftType CT, FASMAC, Kanagawa, Japan) and Cas9 protein (New England Biolabs, Tokyo, Japan) were mixed and incubated at 37 °C for 20 min to form a ribonucleoprotein complex (RNP). The ssODN (5’- cct tcc tca gGG TGA GCA CCT TGG CAG AGG CAC AAG AAC AGA TAT ATA CTC TGG GAC CCT GCT GGA CTA CAA GGA TGA GGA AGG AAT TG-3’) was designed to include silent (synonymous) c.1788C>A and c.1791T>C mutations to avoid re-cleavage by Cas9, and the target c.1783C>G mutation was obtained from FASMAC. The final concentrations of components in RNP preparation with ssODN were 8 μM guide RNA (crRNA + tracrRNA), 200 ng/μl Cas9 protein, and 250 ng/μl ssODN. The mixture was electroporated into zygotes using a NEPA 21 electroporator (NEPA GENE Co. Ltd., Chiba, Japan) and the embryos were transferred into the oviductal ampulla of pseudo-pregnant ICR mice.

For sequencing and genotyping, genomic DNA was extracted using KAPA Express Extract (Kapa Biosystems, Woburn, MA) from the pinna and tail of the offspring and were used for PCR amplification. The region targeted by the Cas9 nuclease was amplified by using a GoTaq Green Master mix (Promega, Madison, WI, USA) and a primer pair (5’- CAG GTT TGT GAT AGA CTG CAG CTG-3’ and 5’- CAT CAT TCT CCC CTC ACT ACT CCC-3’). Mutations in the *Jak1* gene in offspring were confirmed by Sanger sequencing of the PCR products using Eurofins DNA sequence service (Eurofins Genomics, Tokyo, Japan). Potential off-target cleavage sites predicted by the CRISPOR website (**Supplemental Table 1**, the five regions with the highest Mit off-target scores) were sequenced and no mutations were detected in these sites.

### Cell Culture and Transfection

The HEK293 cell line (JCRB9068, Graham, F.L. established) was obtained from the Japanese Collection of Research Bioresources (Osaka, Japan). *JAK1* complementary DNA carried on the pFN21A vector (Halotag ORF Clone FHC01306) was purchased from the Kazusa DNA Research Institute (Chiba, Japan), and the *JAK1* mutation c.1786C>G;p.H596D was introduced to FHC01306 by Promega Japan (Tokyo, Japan). The HEK293 cells were cultured in DMEM containing 1.8 mM calcium supplemented with 10% fetal bovine serum at 37°C with 5% CO_2_. For the transfections, the HEK293 cells were cultured in 12-well dishes and then transfected with wild-type *JAK1*, mutant *JAK1* (H596D), or HaloTag Control Vector plasmids using Screen Fect A plus transfection reagent (FUJIFILM Wako Pure Chemical Corporation, Tokyo, Japan) according to the manufacturer’s protocol. Cells were cultured for 24 or 48 hours after being transfected with the indicated plasmids and were collected for Western blotting analysis. 

### Immunohistochemistry

Immunohistochemical analysis of skin samples from the participants and mice was performed as described previously (11), with slight modifications. Thin sections (3 μm) were cut from samples embedded in paraffin blocks. The sections were soaked for 20 min at room temperature in 0.3% H_2_O_2_/methanol to block endogenous peroxidase activity. After washing in PBS with 0.01% Triton X-100, the sections were incubated for 30 min in PBS with 4% BSA followed by an overnight incubation with the primary antibodies in PBS containing 1% BSA according to the manufacturer’s instructions. After washing in PBS, the thin sections were stained with the corresponding secondary antibodies for 1 hour at room temperature and washed in PBS. The Vectastain Elite ABC-PO kit (Vector Laboratories, Burlingame, CA) was used for staining. The following polyclonal antibodies were purchased from commercial sources: anti-p-JAK1 [phospho-Tyr1022, anti-p-Jak1 (Tyr1021), Sigma Aldrich, St Louis, MO), anti-p-STAT1 (phospho-Tyr701, anti-p-Stat1 (Tyr701)] (ab30645; Abcam, Cambridge, UK), anti-p-STAT3 (phospho-Tyr705, anti-p-Stat3 (Tyr705) (#11045; Signalway Antibody, College Park, MD). The following monoclonal antibodies were purchased from commercial sources: anti-p-STAT5 [phospho-Tyr694, anti-p-Stat5 (Tyr694)] (ab32364; Abcam), anti-p-STAT6 [phospho-Tyr641, anti-p-Stat6 (Tyr641)] (ab263947; Abcam). n=3. Each experiment was performed twice.

### Western Blotting

Zirconia balls were added to the proteins extracted from the liver of P0 newborns. Then, the proteins were dissolved in 1 ml sample buffer (NuPAGE LDS sample buffer 250μL, sample reducing agent 100μL, 25×protease inhibiter 40μL, and water 610μL) and crushed. After centrifugation at 10,000 rpm for 10 min at 4°C, the supernatant of each sample was subjected to SDS-PAGE. Strips of membrane were incubated with anti-p-JAK1 (Tyr1021), anti-JAK1 (ab125051; Abcam), anti-p-STAT1 (Tyr701), anti-STAT1 (ab99415; Abcam), anti-p-STAT3 (Tyr705), anti-STAT3 (SAB4300708, Sigma Aldrich), anti-p-STAT5 (Tyr694), anti-STAT5 (ab16276; Abcam), anti-p-STAT6 (Tyr641), anti-STAT6 (ab32520; Abcam) or anti-GAPDH (ab9485; Abcam) antibodies. The antibody–antigen complexes were detected with horseradish peroxidase–conjugated goat anti-rabbit IgG (Dako, Glostrup, Denmark) at a dilution of 1:1,000, followed by detection with enhanced chemiluminescence Western blotting substrate (GE Healthcare BioSciences, Little Chalfont, UK), as described by the manufacturer. n=3. Each experiment was performed three times. For HEK293 cell lysates, additional anti-HaloTag monoclonal antibody (G9211; Promega Corporation, WI) and anti-JAK1 rabbit monoclonal antibody (#3344; Cell Signaling Technology, MA) were used as the primary antibodies.

### RNA Sequencing

Total RNA extracted from the brain, liver, and skin of P0 newborns was purified using the RNeasy Mini Kit (QIAGEN, Hiden Germany). Two *Jak1* ^H595D/+;I596I/+;Y597Y/+^ mice and five WT mice were analyzed. The quality of RNA was assessed with a 2100 Bioanalyzer (Agilent Technologies). The skin samples (n=7) had an average RNA integrity number (RIN) values of 6.48 (5.8 - 7.1). The blood and liver samples (n=7 each) had an average RIN value of 9.63 (9.2 - 10). RNA sequencing was performed by the Macrogen Japan Corp. using the TruSeq RNA Library Prep Kit v2 (Tokyo, Japan). Next-generation sequencing was performed using the Illumina Novaseq 6000 platform to obtain 101-bp paired-end reads. The reads were adapter-trimmed using our in-house script and were aligned to the mm9 reference genome using TopHat2 (https://ccb.jhu.edu/software/tophat/). Reads Per Kilobase of transcript, per Million mapped reads (RPKM) were calculated using GFOLD (12). Changes in expression levels between groups were estimated using GFOLD, and the resulting GFOLD (0.1) values (conservative estimation of fold changes at the confidence level of q = 0.1) were used for gene set enrichment analyses (GSEA) (13, 14). Volcano plots were generated using log2-fold change values and adjusted p values calculated by DESeq2 (15).

## Results

### A *De Novo* Mutation in *JAK1* in a Patient With AiKD With Hepatitis and Autism

**Figure 1:**
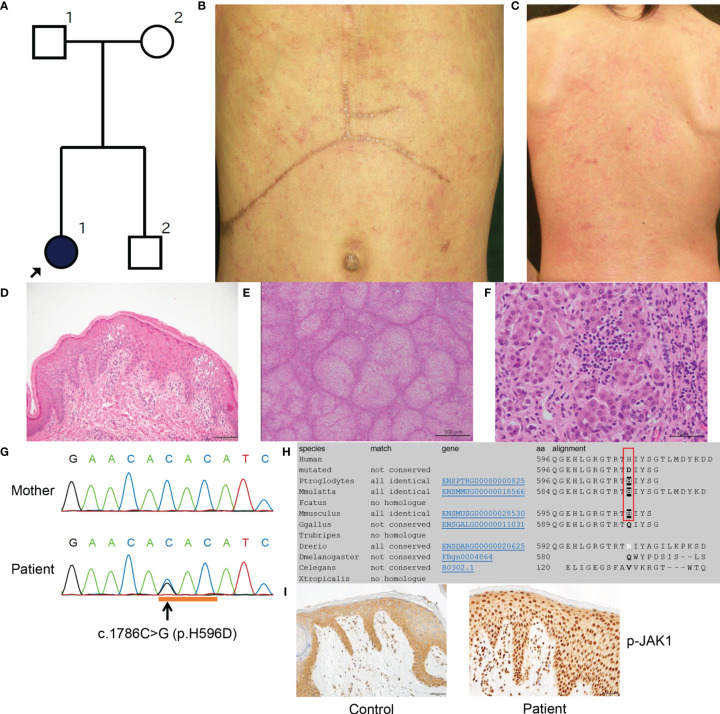
Clinicopathologic features of the present patient with a *JAK1* mutation. **(A)** Family tree of the present pedigree. **(B, C)** Scratched erythema and small red papules are seen on the trunk **(B)** and the back **(C)**. **(D)** A biopsy sample from erythematous skin of the proband shows compact hyperkeratosis, moderate acanthosis, mild spongiosis and lymphocytic infiltration within the epidermis and the upper dermis. Scale bar = 100 µm. **(E)** Histologically, the structure of the liver parenchyma has been remodelled into a nodule by fibrosis, resulting in cirrhosis. Scale bar = 500 µm. **(F)** Inflammatory cell infiltration mainly composed of small lymphocytes (a few plasma cells) is observed in the nodule, accompanied by a mild necrotic inflammatory reaction with hepatocyte shedding. Scale bar = 50 µm. **(G)** Sanger sequencing reveals the heterozygous missense mutation c.1786C>G (H596D) in *JAK1*. **(H)** *JAK1* amino-acid sequence alignment shows the level of conservation in diverse species of the amino-acid p.H596 (red box), which was altered by the missense mutation in the present patient. **(I)** The epidermis in an affected lesion from the patient and normal skin from a healthy donor were stained with anti-p-JAK1 antibody. Scale bars = 50 µm. MutationTaster (http://www.mutationtaster.org/).

Our proband was a 22-year-old Japanese female who was the older of two siblings born to non-consanguineous parents with no significant family history (**Figure 1A**). She was delivered at 38 weeks of gestation by spontaneous vaginal delivery with a birth weight of 2718g. From birth, she was noted to have dry skin with mild erythema and was diagnosed with atopic dermatitis (AD) at the age of 2 months. At around the same time, she was found to have hepatosplenomegaly. Other clinical manifestations noted included low height and body weight, moderate motor impairment and learning disability, hyperlipidemia, and autism. She was treated with a growth hormone, although it was ineffective. She was provisionally diagnosed with glycogen storage disease type IV, and at 3 years of age, she received a living donor liver transplant due to severe liver failure. At 8 years of age, she developed erythematous cheeks and ichthyotic erythema on her trunk (**Figures 1B, C** and **S1**) and extremities, with a SCORing Atopic Dermatitis (SCORAD) score of 70.8%. She was treated with narrow-band UVB therapy, but it led to only minimal improvement. She had eosinophilia and elevated serum thymus and activation-regulated chemokine (TARC; 4,092 pg/mL (normal range, <450 pg/mL)) and IgE with high titers for various antigens (**Supplemental Table 2**). She died of unknown cause at the age of 22 years.

At 7 months of age, a skin biopsy specimen from the affected skin showed compact hyperkeratosis with normal-appearing granular layers, acanthosis, spongiosis, and lymphocytic infiltration from the upper dermis to the granular layers (**Figure 1D**). Additionally, excised liver tissue demonstrated liver cirrhosis with remodeling of the nodular structure due to fibrosis (**Figure 1E**). Most hepatocytes were brightened or vacuolar due to presumed lipid or glycogen deposition. Although the inflammatory cell infiltrates mainly consisted of lymphocytes (with a minority of plasma cells) and were located principally within fibrotic lesions, they were also observed in hepatocyte nodules and sinusoids, and they caused a mild necrotic inflammatory reaction accompanied by hepatocyte shedding (**Figure 1F**).

Following institutional ethical approval, informed written consent was obtained in compliance with the Declaration of Helsinki guidelines. We started by searching for mutations in *ABHD5* (αβ-hydrolase domain-containing 5), the gene implicated in Chanarin–Dorfman syndrome (MIM 275630). Sanger sequencing of *ABHD5* revealed no mutations in the genomic DNA of the patient. Moreover, intra-cytoplasmic lipid droplets within venous neutrophils were not observed. Whole-exome capture was then performed (using peripheral blood genomic DNA isolated from the patient and both parents) by in-solution hybridization using SureSelect All Exon 50 Mb Version 5.0 (Agilent, Santa Clara, CA, U.S.A.) followed by massively parallel sequencing (HiSeq2500; Illumina, San Diego, CA, U.S.A.) with 150-bp paired-end reads. After filtering for low frequency variants (frequency of less than 0.1% in the ExAC, 1000 genomes and ESP6500 databases) and against the parental exome dataset, 53 *de novo* heterozygous variants were identified (**Supplemental Table 3**). Amongst these variants, a nonsynonymous heterozygous mutation was identified in *JAK1* (c.1786C>G; H596D), which was validated by Sanger sequencing (**Figure 1G**). This mutation had not been described in our in-house database of 777 Japanese exomes, nor in the gnomAD Database (16), which includes data for 125,748 whole exomes and 15,708 whole- genomes. *In silico* analysis with MutationTaster (17) and SIFT (http://sift.jcvi.org/) predicted this variant to be ‘damaging’. Results from other predictive tools included a Combined Annotation Dependent Depletion (CADD) score of 15.27 and Genomic Evolutionary Rate Profiling (GERP) score of 4.09 (highly conserved); thus, the mutation is thought to be functionally relevant. The histidine residue at codon 596 of JAK1 is conserved among three diverse species: *P. troglodytes*, *M. mulatta* and *M. musculus* (**Figure 1H**). The variant allele frequency of H596D mutation in the patient’s peripheral blood mononuclear cells was 0.421053. As such, we cannot determine if this mutation is a germline or somatic mutation. We did not identify potentially pathogenic mutations in other genes implicated in autoinflammatory disease, ichthyosis, or glycogen storage disorders.

### Strong Nuclear Staining of STAT Family Members in the Epidermis of the AiKD Patient With the *JAK1* Mutation

We conducted immunohistochemical analyses of phosphorylated-JAK1 (p-JAK1), -STAT1 (p-STAT1), -STAT3 (p-STAT3), -STAT5 (p-STAT5) and -STAT6 (p-STAT6) in lesional skin from the patient. The patient’s epidermis showed strong nucleocytoplasmic JAK1 expression, in contrast to the mostly cytoplasmic staining of normal skin (**Figure 1I**). Epidermal cytoplasmic p-STAT1 expression with focal nucleocytoplasmic localization was also seen in the skin from the patient (**Figures S2A, B**). In addition, p-STAT3, p-STAT5 and p-STAT6 were strongly expressed in the nuclei of keratinocytes in the patient (**Figures S2D, F, H**) compared with predominantly cytoplasmic staining in normal control skin samples (**Figures S2C, E, G**).

### *Jak1* Knock-in (*Jak1* ^H595D/+;I596I/+;Y597Y/+^) Mice Recapitulate Aspects of Human AiKD With Hepatitis due to *JAK1* Mutation

**Figure 2:**
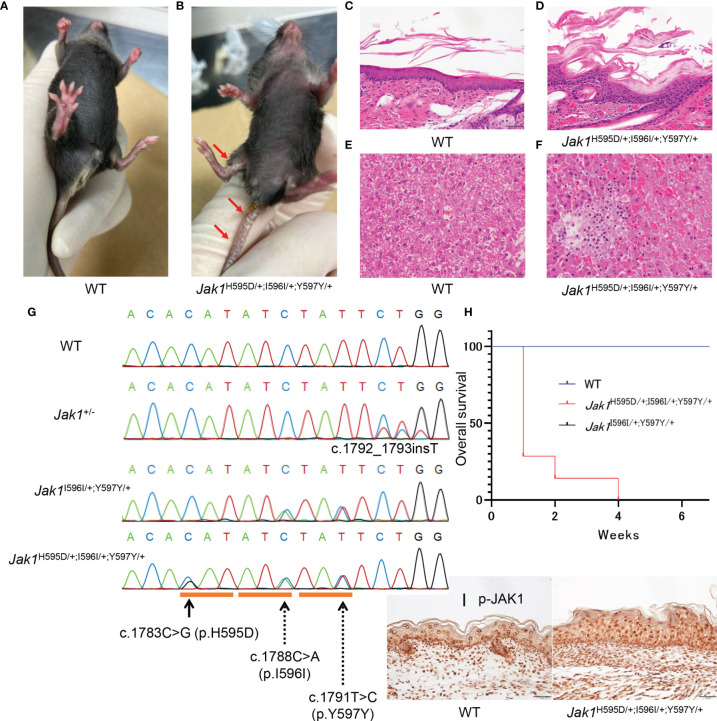
Phenotypic features in *Jak1* ^H595D/+;I596I/+;Y597Y/+^ mice. **(A, B)** The gross appearance of WT **(A)** and *Jak1* ^H595D/+;I596I/+;Y597Y/+^ **(B)** at 4 weeks of age. The *Jak1* ^H595D/+;I596I/+;Y597Y/+^ mouse shows scaling on the ears, the extremities, and the tail. **(C, D)** Hematoxylin and eosin staining of the skin reveals acanthosis, hyperkeratosis and parakeratosis in the *Jak1* ^H595D/+;I596I/+;Y597Y/+^ mouse **(D)**, but not in the WT mouse **(C)**. Scale bars = 50 µm. (n = 3) **(E, F)** Histology of liver sections from the *Jak1* ^H595D/+;I596I/+;Y597Y/+^ mice **(F)** and the WT mice **(E)** stained with hematoxylin and eosin. Scale bars = 50 μm. (n = 3) **(G)** Sequence data of *Jak1* around the mutations in the *Jak1* ^H595D/+;I596I/+;Y597Y/+^, *Jak1* ^I596I/+;Y597Y/+^ and *Jak1* ^+/-^ mice. The black line indicates c.1783C>G (H595D). The dotted lines indicate two synonymous changes: c.1788C>A (I596I) and c.1791T>C (Y597Y). **(H)** A graph of the overall survival rate (*Jak1* ^H595D/+;I596I/+;Y597Y/+^: n = 7, WT: n = 10, *Jak1* ^I596I/+;Y597Y/+^: n=10). Log-rank test, WT vs *Jak1* ^H595D/+;I596I/+;Y597Y/+^ mice: p value < 0.0001, *Jak1* ^I596I/+;Y597Y/+^ vs *Jak1* ^H595D/+;I596I/+;Y597Y/+^ mice: p value < 0.0001. **(I)** Immunohistochemical analysis by using the anti-p-JAK1 antibody for the palmar skin of the *Jak1* ^H595D/+;I596I/+;Y597Y/+^ mice and the WT mice. Scale bars = 50 µm. (n = 3).

To gain deeper insights into the role of JAK1 hyperactivity in the autoinflammatory pathogenesis *in vivo*, we used a CRISPR-Cas9 gene-targeting approach to generate KI mice. We initially attempted to generate mice harboring only the c.1783C>G (H595D) substitution (*Jak1* ^H595D/+^), which is identical to that found in the patient. However, we could not design a high-quality guide RNA for CRISPR to target the conserved histidine residue. To avoid re-cutting by Cas9, we introduced two additional synonymous variants at the following amino acids: c.1788C>A (I596I) and c.1791T>C (Y597Y) (**Figure 2G**). Furthermore, we utilized the *Jak1*KI-mosaic male mouse born by chance because heterozygous *Jak1* ^H595D/+;I596I/+;Y597Y/+^ mice (H595D, I596I, Y597Y) could not survive longer than 4 weeks. Mosaic*-Jak1* ^H595D/+;I596I/+;Y597Y/+^ male mouse manifested a milder phenotype than *Jak1* ^H595D/+;I596I/+;Y597Y/+^ mice. The majority of somatic cells in the mosaic*-Jak1* ^H595D/+;I596I/+;Y597Y/+^ mice harbored only wild-type (WT) alleles. The mosaic*-Jak1* ^H595D/+;I596I/+;Y597Y/+^ male mouse survived for more than 1 year and were able to produce offspring including heterozygous *Jak1* ^H595D/+;I596I/+;Y597Y/+^ mice (**Supplemental Table 4**).

*Jak1* ^H595D/+;I596I/+;Y597Y/+^ mice seemed to have small body sizes and low weights at birth. From a few days after birth, these mice showed hyperkeratosis and scales on the ears, feet, and tail (**Figures 2A, B**). KI mice with only the two introduced synonymous heterozygous mutations (*Jak1* ^I596I/+;Y597Y/+^) did not show any abnormal phenotypes and survived as long as WT mice. Of note, the survival rate was significantly lower for *Jak1* ^H595D/+;I596I/+;Y597Y/+^ mice than for WT and *Jak1* ^I596I/+;Y597Y/+^ mice (**Figure 2H**). We incidentally generated two types of heterozygous *Jak1* mice carrying null alleles caused by frame-shift mutations (c.1792_1793insT and c.1790_1794del5; *Jak1* ^+/-^). Both types of heterozygous *Jak1* ^+/-^ mice exhibited no cutaneous phenotypes.

As expected, *Jak1* ^H595D/+;I596I/+;Y597Y/+^ mice exhibited moderate thickening of the stratum corneum, a reduced number of keratohyalin granules in the uppermost stratum granulosum, and epidermal hyperplasia (**Figures 2C, D** and **S3B**). Additionally, lymphocytic infiltration was seen in the liver of *Jak1* ^H595D/+;I596I/+;Y597Y/+^ mice (**Figures 2F** and **S3D**), but not in the liver of WT mice (**Figures 2E** and **S3C**). These findings are consistent with the histological features of the skin and liver samples from our AiKD patient with the *JAK1* mutation (**Figures 1E, F**).

### Phosphorylation of JAK1 and STAT Family Members Also Seen in *Jak1* ^H595D/+;I596I/+;Y597Y/+^ Mice

Stronger nucleocytoplasmic staining of p-JAK1 was observed in the palmar skin of *Jak1* ^H595D/+;I596I/+;Y597Y/+^ mice than in the palmar skin of WT mice (**Figure 2I**). p-STAT3 and p-STAT6 were also detected in the nucleus (**Figures S3E-H**). We also performed Western blot analyses to determine whether the phosphorylation of JAK1 and STAT were increased in the liver tissue of *Jak1* ^H595D/+;I596I/+;Y597Y/+^ mice. JAK1, STAT1, STAT3, STAT5, and STAT6 were more highly phosphorylated in the liver cells of *Jak1* ^H595D/+;I596I/+;Y597Y/+^ mice compared to WT mice (**Figure S3I**).

### Gene Expression Profile in the Brain, the Liver, and the Skin of *Jak1* ^H595D/+;I596I/+;Y597Y/+^ Mice

**Figure 3:**
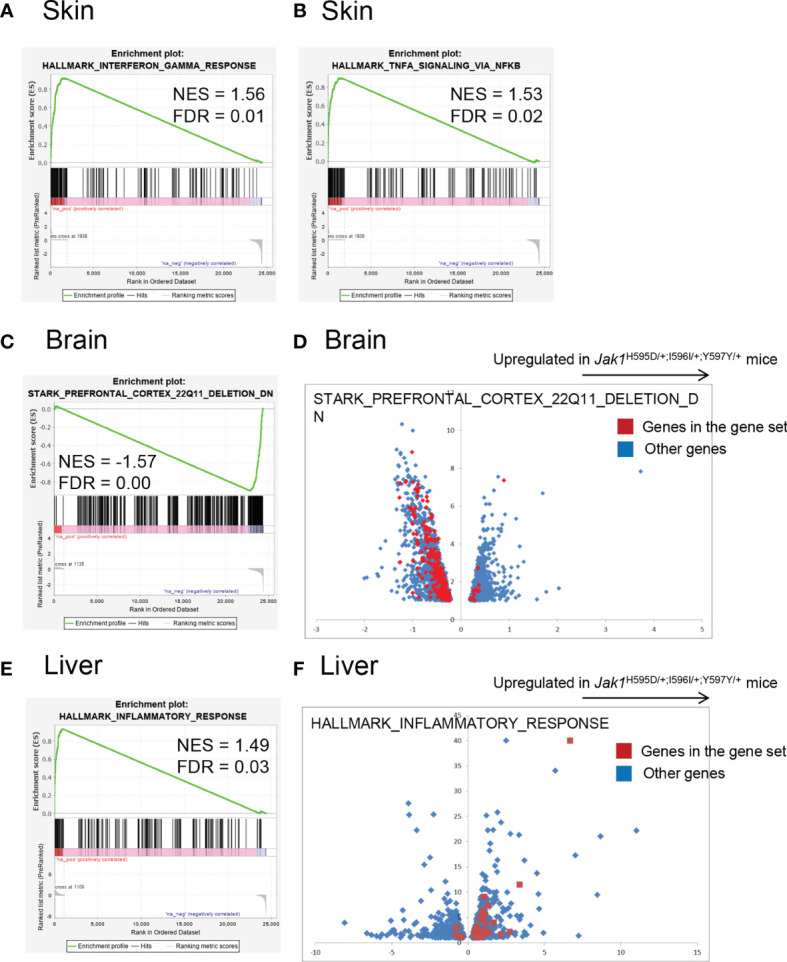
Global gene expression profiling of the RNA samples in *Jak1* ^H595D/+;I596I/+;Y597Y/+^ mice. Gene set enrichment analysis using the hallmark gene set database. **(A, B)** Skin. Genes associated with HALLMARK_INTERFERON_GAMMA_RESPONSE **(A)** and HALLMARK_TNFA_SIGNALING_VIA_NFKB **(B)** are relatively upregulated in skin samples from the *Jak1* ^H595D/+;I596I/+;Y597Y/+^ mice. **(C, D)** Brain. Genes associated with STARK_PREFRONTAL_CORTEX_22Q11_DELETION_DN are downregulated in brain samples from the *Jak1* ^H595D/+;I596I/+;Y597Y/+^ mice. The red dots in **(D)** indicate genes in this gene set. Blue dots: the other genes. **(E, F)** Liver. Genes associated with HALLMARK_INTERFERON_GAMMA_RESPONSE are upregulated in liver samples from the *Jak1* ^H595D/+;I596I/+;Y597Y/+^ mice. The red dots in **(F)** indicate genes in this gene set. Blue dots: the other genes. NES, normalized enrichment score; FDR, false discovery rate.

To compare global gene expression profiles, RNA sequencing was performed using extracted RNA from the brain, liver, and skin of newborn *Jak1* ^H595D/+;I596I/+;Y597Y/+^ mice and WT mice (**Figure 3** and **Tables S5-S7**). We performed a gene set enrichment analysis using the hallmark gene set database. In all three samples (brain, liver, and skin) of *Jak1* ^H595D/+;I596I/+;Y597Y/+^ mice, genes associated with the hyperactivation of tyrosine kinases (**Figure 3A** and **Figure S4A**) and with the activation of NF-κB signaling (downstream of tyrosine kinases) were upregulated (**Figure 3B** and **Figure S4B**). Brain samples also showed the upregulation of genes associated with the extracellular matrix and the downregulation of genes associated with oxidative phosphorylation, metabolism of lipid-associated genes, and haptotaxis-associated genes (**Figure S4C**). Intriguingly, the genes downregulated in *Jak1* ^H595D/+;I596I/+;Y597Y/+^ mice showed a very strong correlation with those downregulated in the brain of the model mice with 22q11.2 deletion syndrome (q<0.001) (**Figures 3C, D** and **Table S5**) (18). Liver tissue from the *Jak1* ^H595D/+;I596I/+;Y597Y/+^ mice also showed the upregulation of genes associated with inflammation, including IL-6 (**Figures 3E, F** and **Table S6**). In terms of STAT family genes, *Stat3* was upregulated in differentially expressed genes in the skin (**Table S7**), and *Stat4* was upregulated in differentially expressed genes in the liver (**Table S6**).

The enrichment of genes associated with interferon-gamma and tumor necrosis factor-alpha was similar among the three types of tissues. The enrichment of genes associated with 22q11 deletion was specific to the brain. The enrichment of genes associated with inflammation was prominent in the liver, while in the other two tissues the enrichment did not reach the statistical significance (q = 0.59 and 0.187 for the brain and the skin, respectively).

### Gain of Function of H596D Mutant JAK1 in Patient *In Vitro*

Finally, to investigate whether JAK1-STAT pathways were upregulated due to H596D mutation in *JAK1*, we transfected the *JAK1* mutant (*JAK1*-H596D) into HEK293 cells and performed Western blotting analyses to evaluate the phosphorylation of JAK1 and STAT proteins. As shown in **Figure S5**, JAK1 was more highly phosphorylated in the *JAK1*-H596D-transfected HEK293 cells than in the wild-type *JAK1*-transfected cells. In addition, the *JAK1*-H596D-transfected cells showed higher phosphorylation for STAT1 (48h), STAT5 (24h and 48h), and STAT6 (48h) than wild-type *JAK1*-transfected cells did. We observed no significant difference in the STAT3 phosphorylation levels between wild type and mutant in the present experiments. The results for phosphorylation levels of STAT1 were similar to those obtained in a transfection study of *JAK1* A634D in the previous report (6).

## Discussion

Our clinical observations and supportive findings from a KI mouse model implicate the H596D mutation in *JAK1* as crucial for chronic systemic inflammation. The proband’s inflammatory skin phenotype was typical of autoinflammatory keratinization disease (AiKD) (19) and presented with extracutaneous features, including hepatitis and autism. *Jak1* ^H595D/+;I596I/+;Y597Y/+^ mice demonstrated evidence of inflammation involving the skin and the liver. Immunohistochemical analyses of skin samples from the patient and the KI mouse model suggest that the *JAK1* H596D mutation confers a gain-of-function effect leading to the hyperactivation of JAK-STAT signaling (**Figure 2B**). Accelerated phosphorylation of JAK1 and STATs seen in the 293 cells transfected with *JAK1* H596D mutation verifies the gain-of-function effects *in vivo* (**Figure S5**).

**Figure 4:**
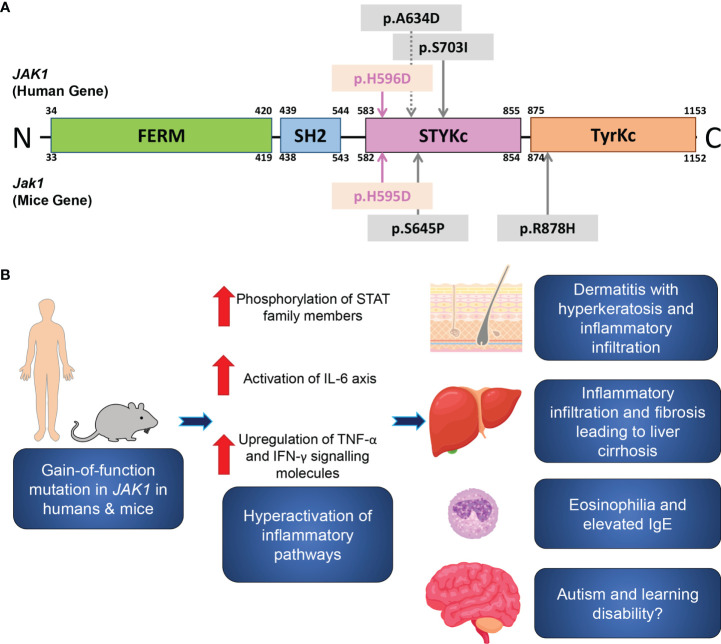
A schematic of the JAK1 domain structure, and inflammatory pathways associated with JAK1 hyperactivation. **(A)** A schematic of the functional domains and the site of the reported gain-of-function germline mutations in humans (upper) and in mice (lower). The mutations in the present human case and in *Jak1* ^H595D/+;I596I/+;Y597Y/+^ mice are marked by pink arrows. Sites of the previously reported mutations are indicated by grey arrows. FERM, FERM (F for 4.1 protein, E for ezrin, R for radixin and M for moesin) domain; SH2, Src homology 2 domain; STYKc, the serine-threonine/tyrosine-protein kinase, catalytic domain (also called the pseudokinase domain); TyrKc, the tyrosine kinase catalytic domain. **(B)** Theory on the pathogenesis of the inflammatory pathways associated with JAK1 hyperactivation.

The amino acid residue altered by this mutation localizes to the pseudokinase domain of JAK1 (**Figure 4A**). The pseudokinase (or JAK homology-2, JH2) domain is critical for the regulation of the kinase (or JH1) domain of the JAK family proteins (20). *In vitro* analyses demonstrated that the deletion of the JH2 domain of Jak2 led to a dramatic increase in the level of kinase activity (21). The I597F mutation in JAK1, situated within the JAK1 JH2 ATP-binding site and in close proximity to our H596D variant, increased basal STAT5 activation and did not inhibit hyperactivation driven by JAK1-3 (20). Similarly, the H596D substitution may lead to upregulated JH1 kinase activity due to less efficient inhibition by the mutant pseudokinase domain.

Interestingly, the variants identified in three previously reported families with *JAK1* mutations are also situated within the pseudokinase domain, comprising two gain-of-function mutations and one loss-of-function mutation (6, 7, 22). The clinical features reported in the other patients with gain-of-function variants are similar to our proband, including skin inflammation, impairment of growth, and liver abnormalities, although there is already evidence of phenotypic heterogeneity among the patients with this autoinflammatory syndrome. Indeed, our patient had no significant gastrointestinal tract involvement. One previously reported individual developed membranous nephropathy that recurred despite renal transplantation and eventually necessitated hemodialysis (7), although other patients with gain-of-function variants in *JAK1*, including the present case, did not develop any renal impairment. It is evident from these cases that JAK1 dysfunction is associated with multi-system involvement consistent with the widespread expression of JAK1 (2). Nevertheless, the precise phenotypic spectrum associated with gain-of-function *JAK1* mutations may become more defined as more cases are identified.

In contrast to our patient, the case reported by Eletto *et al.* was an immunodeficient patient with different pathobiology due to biallelic *JAK1* mutations (22). The patient harbored two homozygous missense mutations, P733L and P832S, also within the pseudokinase domain of *JAK1* and had recurrent atypical mycobacterial infections and early-onset metastatic bladder carcinoma (22). Cells from this patient showed reduced phosphorylation of JAK1 and STAT following cytokine stimulation, impaired induction of the expression of interferon-regulated genes, and dysregulated cytokine production, suggesting a probable loss of function of JAK1 (22). Collectively, these human data suggest that point mutations in the pseudokinase domain of JAK1 are critical to pathological processes, similarly to the well-known *JAK2* V617F mutation found in polycythaemia vera, myelofibrosis, and essential thrombocythemia (23).

Two published mouse models of increased JAK1 activity had similar features to the human cases, including an inflammatory skin phenotype (24, 25). Sabrautzki et al. (25) reported that an *N*-ethyl *N*-nitrosourea (ENU)-induced mutagenesis-derived mouse with a heterozygous missense mutation in the pseudokinase domain (*Jak1* ^S645P/+^) had reduced body size and weight at birth, inflammatory ear and skin lesions, and erythema and thickening of tails. Liver samples from *Jak1* ^S645P/+^ mice macroscopically demonstrated nodular regenerative hyperplasia with irregular margins, but without hepatomegaly or ascites (25). Histologically, there was sinusoidal dilatation, areas of hyperplastic hepatocytes surrounded by atrophic hepatocytes, prominent vessels and increased vascularization (25). Loss of megakaryocytes and an increase in Russell bodies in the spleen were also noticed (25). Skin histology revealed hyperkeratosis and acanthosis of the epidermis with predominantly neutrophilic infiltration, and immunohistochemical staining showed the activation of the IL-6-gp130-JAK-STAT axis in skin lesions (25). Similarly, our findings also support the involvement of IL-6 in the disease process. Specifically, liver samples from *Jak1*KI mice showed significantly increased mRNA levels of IL-6 and IL-6-related inflammatory pathway molecules (**Figures 3E, F**).

Yasuda et al. (24) reported ENU-induced mutagenized mice carrying the homozygous R878H mutation, termed *Spade* (stepwise, progressive atopic dermatitis). *Jak1^Spade/Spade^* exhibited redness and desquamation of the ears, and skin lesions showed epidermal hyperplasia and infiltration of mononuclear inflammatory cells including mast cells, eosinophils, and CD4^+^ T cells (24). Immunological abnormalities (elevated IgE, IgG1, histamine, IgG2b and IgG2c) developed in a stepwise manner (24). Moreover, *Jak1^Spade/Spade^* demonstrated defective skin barrier function that was postulated to be due to an overexpression of serine proteases such as kallikrein-6 and marapsin. Treatment with petrolatum delayed the onset and reduced the severity of the skin lesions (24).

The global mRNA expression profiling in our study identified a strong correlation between the downregulated genes in the brain tissue of *Jak1* ^H595D/+;I596I/+;Y597Y/+^ mice and those of the model mice with 22q11.2 deletion syndrome (**Figures 3C, D**). 22q11.2 microdeletions are associated with cognitive and behavioral deficits, including autism spectrum disorders (18, 26). Our patient had autism and a moderate learning disability, but these characteristics were not present in the other two reports of individuals with gain-of-function *JAK1* mutations. The sole patient with homozygous loss-of-function *JAK1* mutations did have mild developmental delay (22).

22q11.2 deletion syndrome exhibits significant phenotypic heterogeneity with a wide range of potential features, including congenital cardiac disease, palatal defects, endocrine dysfunction (hypocalcemia, thyroid disease, growth hormone deficiency), autoimmune disease, immunodeficiency, skeletal abnormalities and renal anomalies (27, 28). There are more than 40 protein-coding genes located within the 22q11.2 region (29). Of these, *TBX1* (T-box transcription factor 1) has emerged as a functionally important candidate, as heterozygous mutations in this gene have been found in patients with clinical features resembling 22q11.2 deletion syndrome (30, 31). Another potential gene of interest is *CRKL* (CRK like proto-oncogene, adaptor protein), which is implicated in the development of the heart, aortic arch, parathyroid and thymus glands (32, 33). Interestingly, mice harboring an allele with both *Tbx1* and *Crkl* inactivated recapitulated aspects of the 22q11.2 deletion syndrome phenotype (33).

With regards to the neurobehavioral features seen in 22q11.2 deletion syndrome, the roles of *COMT* (catechol-O-methyltransferase) and *PRODH* (proline dehydrogenase) have been investigated, given their relevance to dopaminergic and glutamatergic neurotransmission, respectively. However, at present there is insufficient evidence to assuredly link these genes functionally to the cognitive and behavioural symptoms of 22q11.2 deletion syndrome (34–37).

There is a growing body of evidence implicating neuro-inflammation in both autism and 22q11.2 deletion syndrome. Individuals with both conditions have elevated pro-inflammatory cytokines, and IL-6 in particular appears to play a substantial role (38, 39). Elevated levels of IL-6 correlate with the extent of cognitive deficit and psychosis in 22q11.2 deletion syndrome (38). An *in vitro* investigation of mouse cerebellar granule cells demonstrated that the overexpression of IL-6 perturbs cellular adhesion and migration, and leads to an imbalance between excitatory and inhibitory circuits (40). The upregulation of JAK-STAT signaling has also been noted in autistic children (41), but there is insufficient evidence at present to establish reliable causal links between aberrant IL-6, JAK1 dysfunctions and autism from this data. However, further investigation is warranted to elucidate the precise pathomechanisms by which JAK1 hyperactivation may lead to neurobehavioral abnormalities.

A key goal of genomic diagnostics is to help identify potential options for targeted therapeutics based on the underlying molecular defects and associated pathways. In the present case, a rational hypothesis is that JAK inhibitors could be an effective treatment for these patients with JAK-STAT hyperactivity. Indeed, both Del Bel *et al.* and Gruber *et al.* noted the amelioration of their patients’ symptoms following treatment with JAK inhibitors (6, 7). The two patients reported by Del Bel *et al.* were treated with ruxolitinib (a JAK1/2 inhibitor), and they demonstrated improvements in pruritus, appetite loss, sleep disturbance and eosinophilia, and achieved weight gain, as well as resolution of skin lesions and hepatosplenomegaly (6). Based on evidence that tofacitinib (a JAK1/2/3 inhibitor) rescues STAT hyperphosphorylation *in vitro*, the individual described by Gruber *et al.* was treated with tofacitinib, resulting in the resolution of her dermatitis and gastrointestinal symptoms (7). JAK inhibitors are increasingly being used to treat autoimmune conditions, including rheumatoid arthritis, inflammatory bowel disease, and psoriasis vulgaris (2). Selective JAK1 inhibitors such as upadacitinib and abrocitinib are also being evaluated in clinical trials for AD (42). These recent developments suggest that JAK inhibitors may be a potent treatment option for controlling the systemic inflammation in our patient.

In summary, we report an individual with a heterozygous gain-of-function mutation in *JAK1*, with consequent hyperactivation of JAK-STAT signaling pathways leading to a syndrome of multi-organ inflammation (**Figure 4B**): AiKD with hepatitis and autism. Our clinicopathologic and functional data from the present patient and the corresponding mouse model support findings from recent reports, with new insights into the significance of the JAK-STAT signaling pathway in human health and disease.

## Data Availability Statement

The NGS data can be accessed at the GEO repository under the accession numbers GSE163100.

## Ethics Statement 

The studies involving human participants were reviewed and approved by the ethics committee of the Nagoya University Graduate School of Medicine. The patients/participants provided their written informed consent to participate in this study. The animal study was reviewed and approved by the Animal Experiment Committee, Graduate School of Medicine, Nagoya University. Written informed consent was obtained from the individual(s) for the publication of any potentially identifiable images or data included in this article.

## Author Contributions

Research design: TT and MA. Experiments: TT, JL, YO, YukM, KT, and KS. Data acquisition: YO, YukM. Data analysis: TT, JL, YO, YuyM, TY, and KT. Collection of clinical samples and information: TT, KI, EN, TOk, and KS. Manuscript writing: TT and JL. Writing assistance: YoM, TOh, JM, and MA. All authors contributed to the article and approved the submitted version.

## Funding

This work was supported by funding from Advanced Research and Development Programs for Medical Innovation (AMED-CREST) 19gm0910002h0105 to MA from the Japan Agency for Medical Research and Development (AMED). This research was also supported by AMED under Grant Number JP20ek0109488 to TT and MA. This research was supported by Health and Labor Sciences Research Grant for Research on Intractable Diseases (20FC1052) from the Ministry of Health, Labor and Welfare of Japan to MA. This study was supported in part by JSPS KAKENHI Grants Number 18H02832 to MA and 20K08648 to TT. This investigation was also supported in part by the Hori Science and Arts Foundation to TT, by grants from the Maruho Takagi Dermatology Foundation to TT, and by the Japanese Dermatological Association Dermatological Research Fund, supported by ROHTO Pharmaceutical Co., Ltd. to TT.

## Conflict of Interest

The authors declare that the research was conducted in the absence of any commercial or financial relationships that could be construed as a potential conflict of interest.

## Publisher’s Note

All claims expressed in this article are solely those of the authors and do not necessarily represent those of their affiliated organizations, or those of the publisher, the editors and the reviewers. Any product that may be evaluated in this article, or claim that may be made by its manufacturer, is not guaranteed or endorsed by the publisher.

## Abbreviations

AD, atopic dermatitis; AiKD, autoinflammatory keratinization disease; JAK1, Janus kinase 1; STAT, signal transducers and activator of transcription.
