# Supplementary material for: Autoinflammatory Keratinization Disease With Hepatitis and Autism Reveals Roles for JAK1 Kinase Hyperactivity in Autoinflammation
Source: Front Immunol. 2022 Jan 3;12:737747. doi: 10.3389/fimmu.2021.737747 (PMC8761858; doi:10.3389/fimmu.2021.737747)
Supplement: Supplementary file 1 [file DataSheet_1.pdf]

Autoinflammatory keratinization disease with hepatitis and autism reveals roles for JAK1 kinase hyperactivity in autoinflammation

Takeichi T, et al.

Supplemental Figures pp. 2-6

Supplemental Figure 1. Cutaneous photos of the patient.

Supplemental Figure 2. Cutaneous hyperactivation of the JAK1-STAT pathway in the patient with *JAK1* mutation.

Supplemental Figure 3. Histological features, IHC and WB results of *Jak1*$^{H595D/+;I596I/+;Y597Y/+}$ mice.

Supplemental Figure 4. Gene set enrichment analysis using the hallmark gene set database of *Jak1*$^{H595D/+;I596I/+;Y597Y/+}$ mice.

Supplemental Figure 5. Western blotting of HEK293 cell lysates to verify hyperactivation of the JAK1-STAT pathways due to H596D substitution of the patient *in vitro*.

Supplemental Tables pp. 7-268

Supplemental Table 1. List of loci and primers for potential off-target cleavage sites predicted by CRISPOR

Supplemental Table 2. Results of blood analysis for the present patient

Supplemental Table 3. List of filtered exome variants

Supplemental Table 4. Frequencies of *Jak1*$^{H595D/+;I596I/+;Y597Y/+}$ genotypes in pups derived from intercrosses between mosaic-*Jak1*$^{H595D/+;I596I/+;Y597Y/+}$ male mouse and wild-type mice

Supplemental Table 5. Differentially expressed genes in the brain at q <0.1. (pp. 18-105)

Supplemental Table 6. Differentially expressed genes in the liver at q <0.1. (pp. 106-169)

Supplemental Table 7. Differentially expressed genes in the skin at q <0.1. (pp. 170-268)

Supplemental Figures

Supplemental Figure 1.

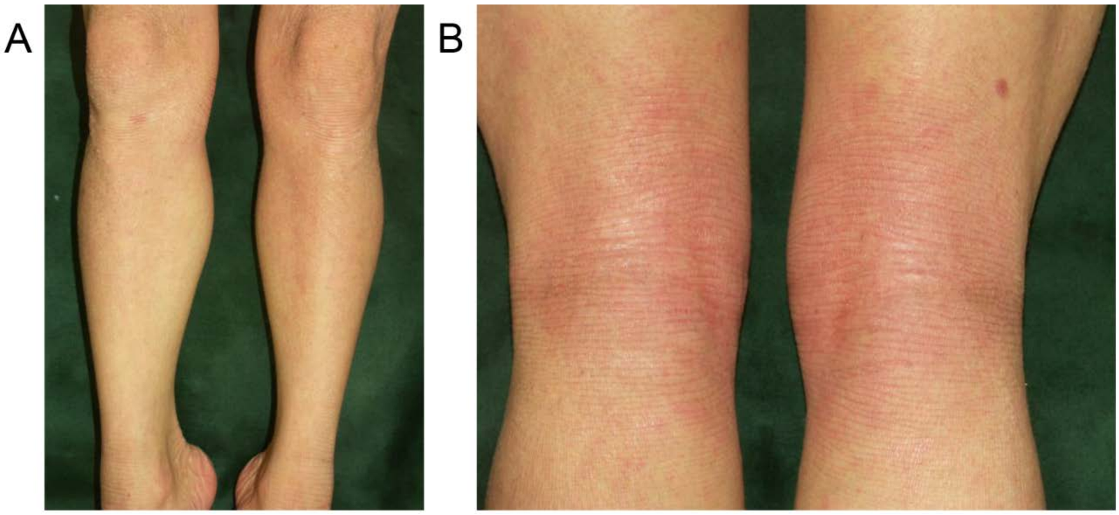

Supplemental Figure 1. Cutaneous photos of the patient.

Hyperkeratotic erythema with fine white scales is seen on the lower legs (A) and the back of the knees (B).

Supplemental Figure 2.

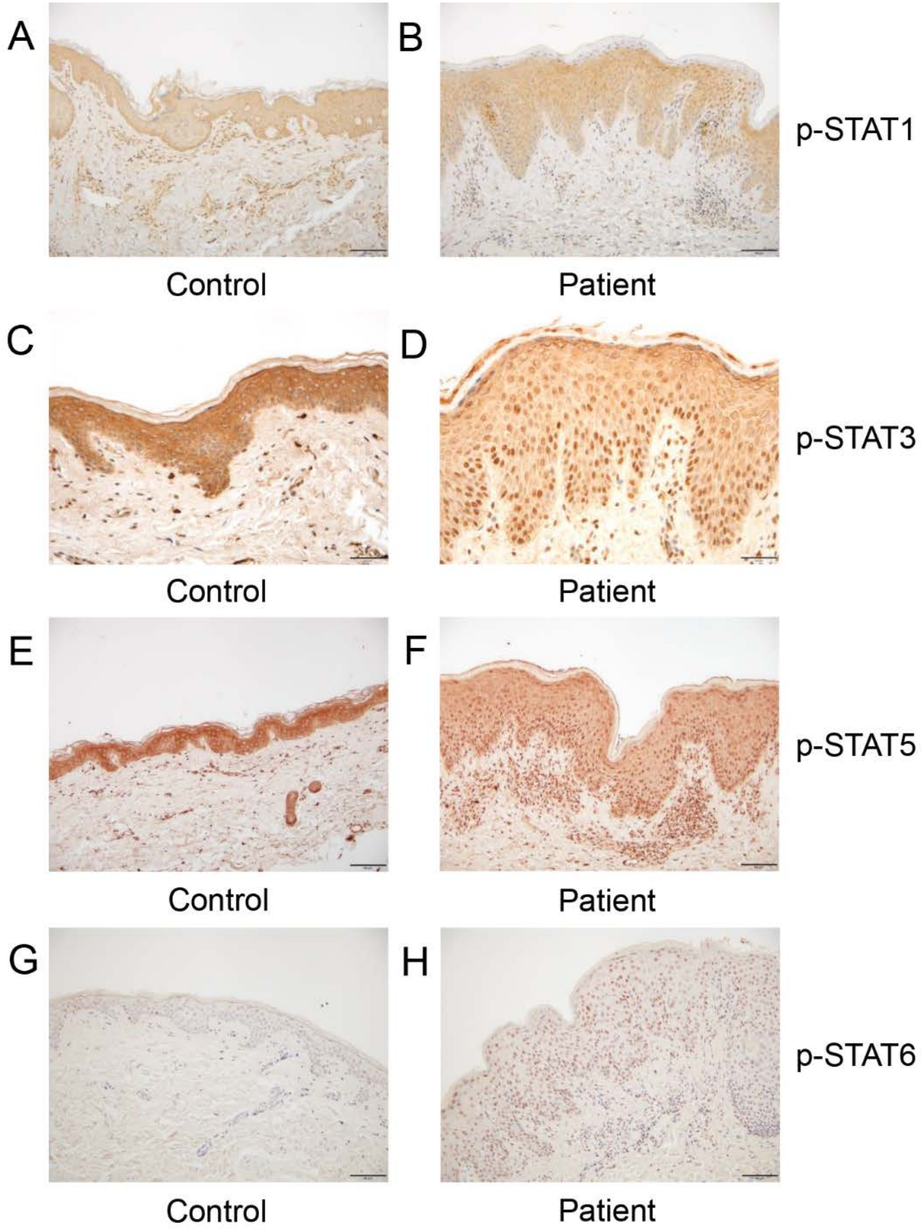

Supplemental Figure 2. Cutaneous hyperactivation of the JAK1-STAT pathway in the patient with *JAK1* mutation.

Skin samples from the patient (B, D, F, H) and normal skin from healthy donors (A, C, E, G) were stained with anti-p-STAT1 (A, B), anti-p-STAT3 (C, D), anti-p-STAT5 (E, F) and anti-p-STAT6 (G, H) antibodies. The nuclear localization of p-STAT3, p-STAT5 and p-STAT6, and the focal nuclear staining of p-STAT1 are seen in the epidermal keratinocytes and dermal infiltrating cells of the patient's epidermis. (A, B, E-H) Scale bars = 100 µm. (C, D) Scale bars = 50 µm.

Supplemental Figure 3.

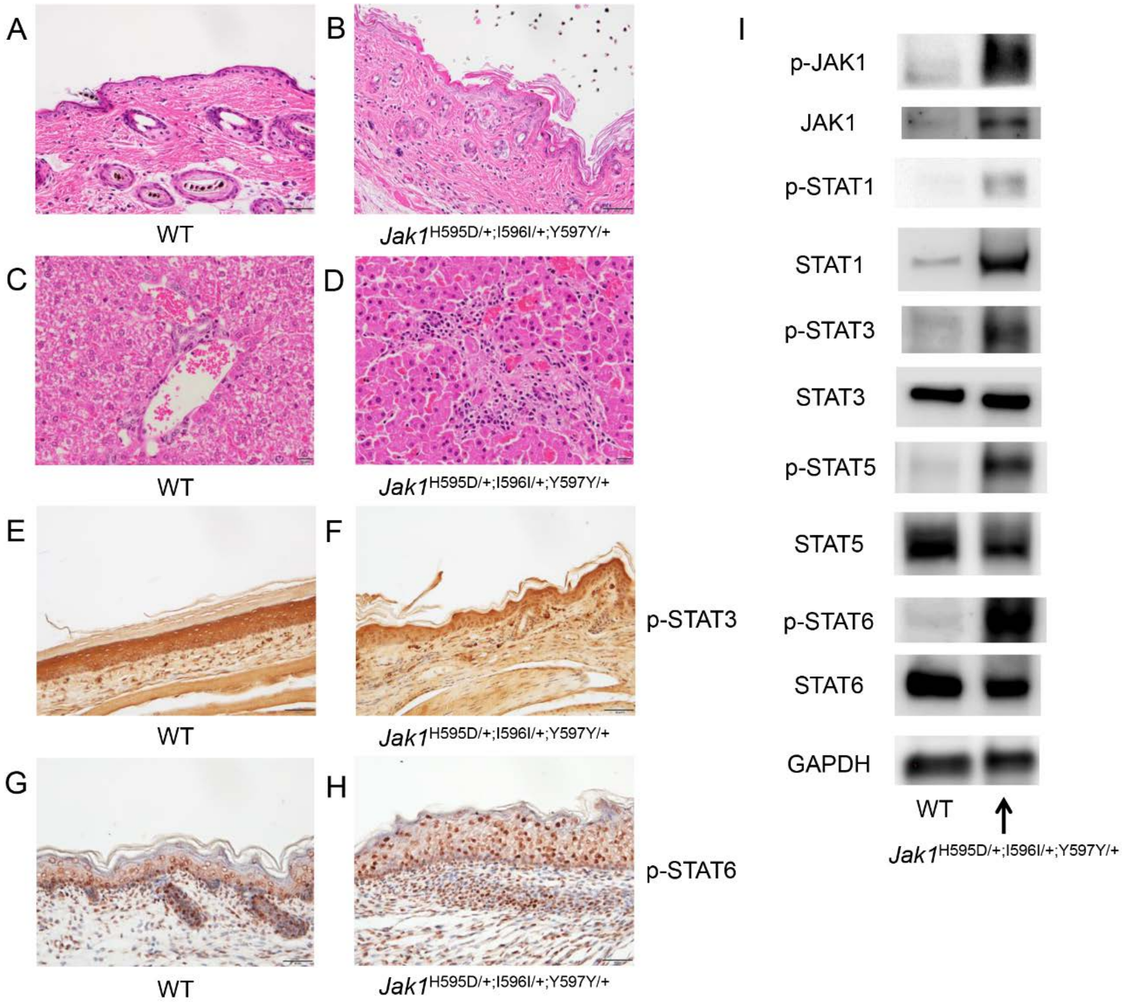

Supplemental Figure 3. Histological features, IHC and WB results of the *Jak1*$^{\mathbf{H595D/+;I596I/+;Y597Y/+}}$.

(A, B) A biopsy sample from the dorsal skin of a *Jak1*$^{H595D/+;I596I/+;Y597Y/+}$ mouse (B) shows marked hyperkeratosis, but the WT mouse shows no hyperkeratosis (A). (C, D) In the liver lobules of the *Jak1*$^{H595D/+;I596I/+;Y597Y/+}$ mice, many lymphocytes are found, with small numbers of neutrophils and plasma cells, and increases in collagen fibres. (E-H) Immunohistochemical analysis by using anti-p-STAT3 (E, F) and anti-p-STAT6 (G, H) antibodies for the palmar skin of *Jak1*$^{H595D/+;I596I/+;Y597Y/+}$ mice (F, H) and WT mice (E, G). (A, B, E-H) Scale bars = 50 µm. (C, D) Scale bars = 20 µm. (n=3) (I) The p-JAK1, JAK1, p-STAT1, STAT1, p-STAT3, STAT3, p-STAT5, STAT5, p-STAT6, and STAT6 protein levels in the liver tissues were evaluated by Western blot analysis. GAPDH was used as an internal loading control. (n=3)

Supplemental Figure 4.

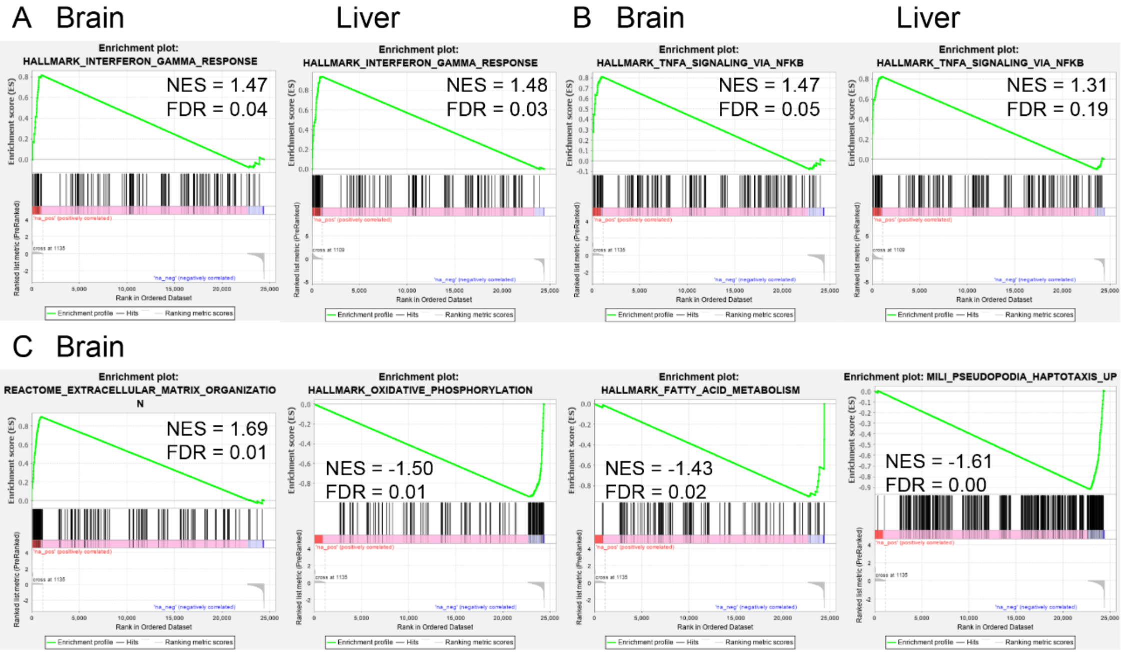

Supplemental Figure 4. Gene set enrichment analysis using the hallmark gene set database of *Jak1*$^{H595D/+;I596I/+;Y597Y/+}$ mice.

Genes associated with HALLMARK_INTERFERON_GAMMA_RESPONSE (A) and HALLMARK_TNFA_SIGNALING_VIA_NFKB (B) are relatively upregulated in the liver and the brain samples from the *Jak1*$^{H595D/+;I596I/+;Y597Y/+}$ mice compared to those of WT mice. (C) Results for brain tissue samples. Genes associated with REACTOME_EXTRACELLULAR_MATRIX_ORGANIZATION are relatively upregulated in *Jak1*$^{H595D/+;I596I/+;Y597Y/+}$ mice compared to those of WT mice. Genes associated with HALLMARK_OXIDATIVE_PHOSPHORYLATION, HALLMARK_FATTY_ACID_METABOLISM and MILI_PSEUDOPODIA_HAPTOTAXIS_UP are relatively downregulated in *Jak1*$^{H595D/+;I596I/+;Y597Y/+}$ mice compared to those of WT mice. NES, normalized enrichment score; FDR, false discovery rate.

Supplemental Figure 5.

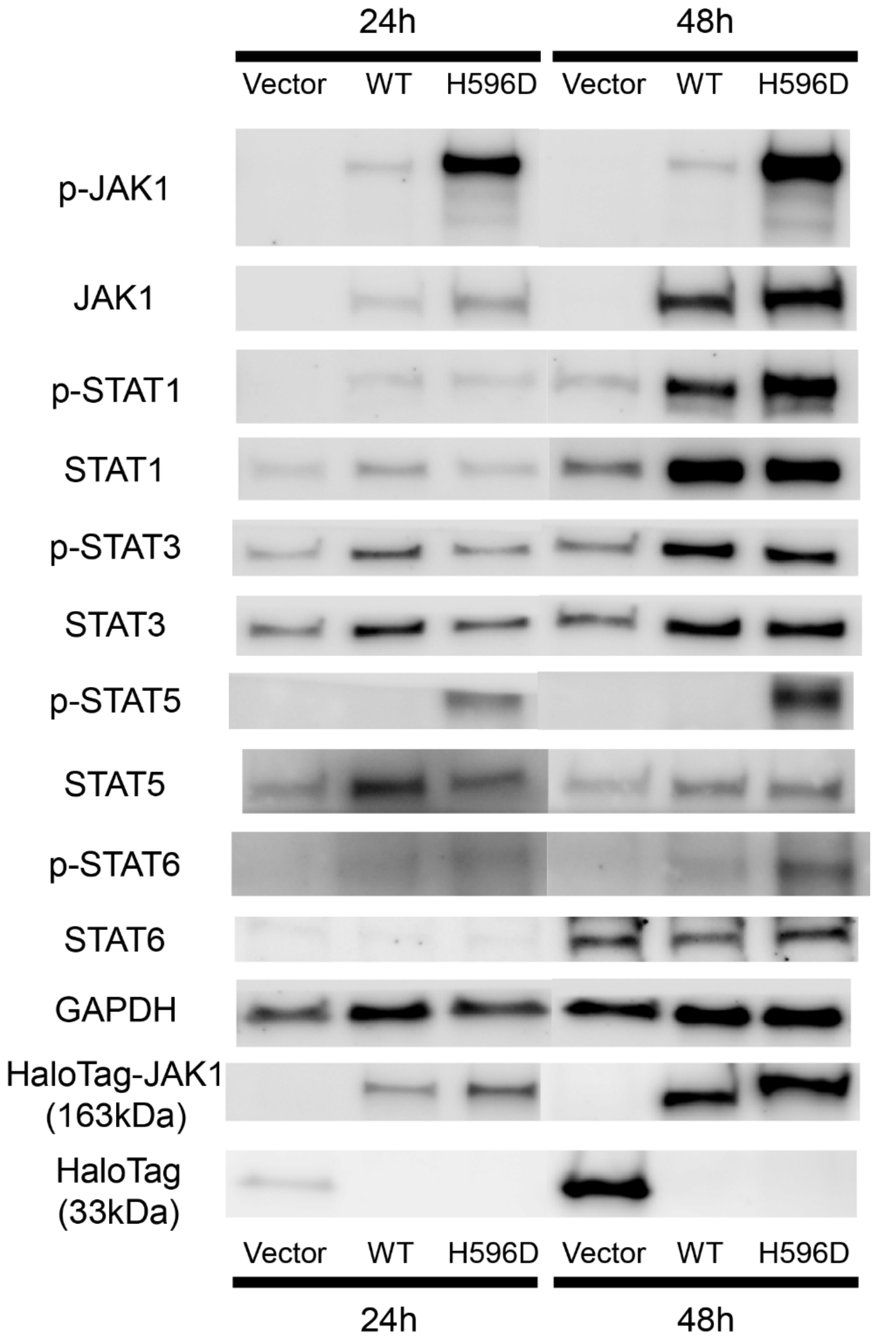

Supplemental Figure 5. Western blotting of HEK293 cell lysates to verify hyperactivation of the JAK1-STAT pathways due to H596D substitution of the patient *in vitro*.

The p-JAK1, JAK1, p-STAT1, STAT1, p-STAT3, STAT3, p-STAT5, STAT5, p-STAT6, and STAT6 protein levels in HEK293 cell lysates were evaluated by Western blot analysis. GAPDH was used as an internal loading control. WT, wild type. (n=3)

Supplemental Tables

Supplemental Table 1. List of loci and primers for potential off-target cleavage sites predicted by CRISPOR

Chrom	Locus	Primer sequence
chr16	intergenic:Impg2-Abi3bp	GAGTGGCAAGGTGAAGCAAT
		TCTTGGGAGAGGACATGTACT
chr9	intron:Lrrc2	AACCAACCCATGTCCTCTTG
		TTAGCCCAAGAAATGCCAAC
chr7	intergenic:Sv2b-Gm24858	AAAGAGGCTTATGGCTGCTG
		ATAATTGGGAAACCGGGAAG
chr8	intron:Vps35	CAGGATGGAATGTTTCAGCA
		TGGAAGCTTACACGGGTTGT
chr12	intergenic:Gm16086-Gm16085	CCTGGGATCTCCACAGAGAG
		CCTGTTGCTCCCAACAGAGT

Supplemental Table 2. Results of blood analysis for the present patient

	15 years old	Normal range
WBC count	9.98	3.3-8.6*10^3/µL
Neutrophil %	34.6	36-74%
Lymphocyte %	48.2	14-55%
Monocyte %	6.7	2-16%
Basophil %	0.6	<4%
Eosinophil %	9.9	<6%
RBC count	496	4.35-5.55*10^6/µL
Haemoglobin	14.9	13.7-16.8 g/dL
Thrombocyte count	49.9	15.8-34.8*10^4/µL
Total protein	6.79	6.6-8.1 g/dL
Albumin	4.33	4.1-5.1 g/dL
AST	28	13-30 U/L
ALT	20	10-42 U/L
Total bilirubin	0.5	0.4-1.5 mg/dL
Direct bilirubin	0.0	0-0.2 mg/dL
g-GTP	19	13-64 U/L
BUN	7.3	8-20 mg/dL
Creatinine	0.37	0.65-1.07 mg/dL
Calcium	9.9	8.8-10.1 mg/dL
TARC	4092	<450 pg/mL
IgE	3352	<233 IU/mL
Anti-cedar pollen antibody	19.50	<0.27 IU/mL
Anti-candida antibody	7.42	<0.27 IU/mL
Anti-house dust antibody	>100.00	<0.27 IU/mL
Anti-milk antibody	0.77	<0.27 IU/mL
Anti-ovalbumin antibody	6.82	<0.27 IU/mL
Anti-soy antibody	8.93	<0.27 IU/mL
Anti-wheat antibody	3.00	<0.27 IU/mL
Anti-chicken antibody	1.65	<0.27 IU/mL
Anti-kiwi fruit antibody	0.70	<0.27 IU/mL

Abbreviations: WBC, white blood cell; RBC, red blood cell; TARC, thymus and activation-regulated chemokine

Supplemental Table 3. List of filtered exome variants

Chr	Start	End	Ref	Alt	Gene	ExonicFunc	AAChange
chr3	27332187	27332187	G	C	NEK10	nonsynonymous SNV	NEK10:NM_152534:exon20:c.C1664G:p.P555R,NEK10:NM_199347:exon20:c.C1664G:p.P555R
chr3	75714819	75714819	G	A	FRG2C	nonsynonymous SNV	FRG2C:NM_001124759:exon4:c.G476A:p.R159Q
chr3	75714831	75714831	G	A	FRG2C	nonsynonymous SNV	FRG2C:NM_001124759:exon4:c.G488A:p.R163Q
chr3	75714835	75714835	C	T	FRG2C	synonymous SNV	FRG2C:NM_001124759:exon4:c.C492T:p.A164A
chr3	75781243	75781243	T	C	ZNF717	nonsynonymous SNV	ZNF717:NM_001290210:exon5:c.A307G:p.I103V
chr3	75786754	75786754	G	T	ZNF717	nonsynonymous SNV	ZNF717:NM_001128223:exon5:c.C2020A:p.R674S,ZNF717:NM_001290208:exon5:c.C2020A:p.R674S,ZNF717:NM_001290209:exon5:c.C1870A:p.R624S
ch	7578	7578	-	T	ZNF717	frameshift	ZNF717:NM_001128223:exon5:c.2009_2010insA:p.T670fs,ZNF717:NM_001290208:exon5:c.2009_2010insA:p.T670fs,

r3	6764	6764				insertion	ZNF717:NM_001290209:exon5:c.1859_1860insA:p.T620fs
chr6	32489933	32489933	T	G	HLA-DRB5	nonsynonymous SNV	HLA-DRB5:NM_002125:exon2:c.A119C:p.D40A
chr11	1018390	1018390	C	A	MUC6	nonsynonymous SNV	MUC6:NM_005961:exon31:c.G4411T:p.A1471S
chr11	55111055	55111055	C	A	OR4A16	nonsynonymous SNV	OR4A16:NM_001005274:exon1:c.C379A:p.P127T
chr11	60718807	60718807	T	G	SLC15A3	nonsynonymous SNV	SLC15A3:NM_016582:exon1:c.A217C:p.T73P
chr13	25671195	25671195	A	G	PABPC3	nonsynonymous SNV	PABPC3:NM_030979:exon1:c.A859G:p.R287G
chr13	25671200	25671200	C	T	PABPC3	synonymous SNV	PABPC3:NM_030979:exon1:c.C864T:p.I288I
chr	25671206	25671206	A	G	PABPC3	synonymous SNV	PABPC3:NM_030979:exon1:c.A870G:p.R290R

13							
chr13	25671210	25671210	C	T	PABPC3	stopgain	PABPC3:NM_030979:exon1:c.C874T:p.Q292X
chr13	25671214	25671214	T	G	PABPC3	nonsynonymous SNV	PABPC3:NM_030979:exon1:c.T878G:p.V293G
chr13	25671369	25671369	G	T	PABPC3	stopgain	PABPC3:NM_030979:exon1:c.G1033T:p.E345X
chr19	9009627	9009627	G	A	MUC16	synonymous SNV	MUC16:NM_024690:exon39:c.C39099T:p.S13033S
chr19	23544782	23544782	A	G	ZNF91	synonymous SNV	ZNF91:NM_001300951:exon3:c.T903C:p.R301R,ZNF91:NM_003430:exon4:c.T999C:p.R333R
chr19	23544789	23544789	A	T	ZNF91	nonsynonymous SNV	ZNF91:NM_001300951:exon3:c.T896A:p.F299Y,ZNF91:NM_003430:exon4:c.T992A:p.F331Y
chr	41522631	41522631	A	G	CYP2B6	nonsynonymo	CYP2B6:NM_000767:exon9:c.A1375G:p.M459V

19						us SNV	
chr19	41522648	41522648	C	T	CYP2B6	synonymous SNV	CYP2B6:NM_000767:exon9:c.C1392T:p.A464A
chr19	55537871	55537871	T	C	KIR3DL2	synonymous SNV	KIR3DL2:NM_001242867:exon7:c.T1101C:p.N367N,KIR3DL2:NM_006737:exon8:c.T1152C:p.N384N
chr19	55537875	55537875	C	G	KIR3DL2	nonsynonymous SNV	KIR3DL2:NM_001242867:exon7:c.C1105G:p.Q369E,KIR3DL2:NM_006737:exon8:c.C1156G:p.Q386E
chr21	47565833	47565833	G	-	FTCD	frameshift deletion	FTCD:NM_006657:exon9:c.997delC:p.R333fs,FTCD:NM_206965:exon9:c.997delC:p.R333fs
chr3	129286359	129286359	G	A	PLXND1	synonymous SNV	PLXND1:NM_015103:exon22:c.C4062T:p.F1354F
chr6	32497901	32497901	C	G	HLA-DRB5	splicing	0
chr6	157100041	157100055	AGGAGGAGCAGGAGC	-	ARID1B	nonframeshift deletion	ARID1B:NM_017519:exon1:c.978_992del:p.326_331del,ARID1B:NM_020732:exon1:c.978_992del:p.326_331del

chr9	40703127	40703127	G	A	SPATA31A3	nonsynonymous SNV	SPATA31A3:NM_001083124:exon4:c.G784A:p.A262T
chr1	26671526	26671526	T	C	AIM1L	synonymous SNV	AIM1L:NM_001039775:exon2:c.A1623G:p.S541S
chr1	65313328	65313328	G	C	JAK1	nonsynonymous SNV	JAK1:NM_002227:exon13:c.C1786G:p.H596D
chr1	145299792	145299792	A	G	NBPF10	nonsynonymous SNV	NBPF10:NM_001039703:exon6:c.A841G:p.S281G,NBPF10:NM_001302371:exon6:c.A841G:p.S281G
chr1	145299906	145299906	T	G	NBPF10	nonsynonymous SNV	NBPF10:NM_001039703:exon6:c.T955G:p.S319A,NBPF10:NM_001302371:exon6:c.T955G:p.S319A
chr1	148024842	148024842	C	A	NBPF8	nonsynonymous SNV	NBPF8:NM_001037501:exon8:c.G1175T:p.R392L
chr1	234561540	234561540	C	T	TARBP1	splicing	0
ch	9812	9812	T	A	ANKR	nonsynon	ANKRD36B:NM_025190:exon39:c.A2872T:p.R958W

r2	8449	8449			D36B	ymous SNV	
chr2	240982047	240982047	A	G	PRR21	nonsynonymous SNV	PRR21:NM_001080835:exon1:c.T353C:p.M118T
chr3	195507077	195507077	G	A	MUC4	nonsynonymous SNV	MUC4:NM_018406:exon2:c.C11374T:p.P3792S
chr3	195513667	195513667	T	G	MUC4	nonsynonymous SNV	MUC4:NM_018406:exon2:c.A4784C:p.K1595T
chr5	146258290	146258290	-	GCTGCTGCTGCTGCTGCT	PPP2R2B	nonframeshift insertion	PPP2R2B:NM_181675:exon1:c.57_58insAGCAGCAGCAGCAGCAGC:p.C20delinsSSSSSSC
chr5	172111131	172111131	G	A	NEURL1B	synonymous SNV	NEURL1B:NM_001142651:exon3:c.G1287A:p.L429L
chr6	16327915	16327915	-	TGC	ATXN1	nonframeshift insertion	ATXN1:NM_001128164:exon7:c.626_627insGCA:p.H209delinsQH,ATXN1:NM_000332:exon8:c.626_627insGCA:p.H209delinsQH

chr7	100551222	100551222	G	C	MUC3A	nonsynonymous SNV	MUC3A:NM_005960:exon2:c.G1803C:p.E601D
chr7	143964267	143964267	A	G	CTAGE4, CTAGE8	nonsynonymous SNV	CTAGE8:NM_001278507:exon1:c.T2077C:p.F693L,CTAGE4:NM_198495:exon1:c.T2077C:p.F693L
chr8	16859310	16859310	G	C	FGF20	nonsynonymous SNV	FGF20:NM_019851:exon1:c.C232G:p.Q78E
chr8	16859332	16859332	A	G	FGF20	synonymous SNV	FGF20:NM_019851:exon1:c.T210C:p.Y70Y
chr8	16859341	16859341	C	G	FGF20	synonymous SNV	FGF20:NM_019851:exon1:c.G201C:p.R67R
chr12	7045892	7045903	CAGCAGCAGCAG	-	ATN1	nonframeshift deletion	ATN1:NM_001007026:exon5:c.1462_1473del:p.488_491del,ATN1:NM_001940:exon5:c.1462_1473del:p.488_491del
chr12	42538349	42538349	T	C	GXYLT1	nonsynonymous SNV	GXYLT1:NM_001099650:exon1:c.A100G:p.T34A,GXYLT1:NM_173601:exon1:c.A100G:p.T34A

chr12	42538352	42538352	C	A	GXYLT1	stopgain	GXYLT1:NM_001099650:exon1:c.G97T:p.G33X,GXYLT1:NM_173601:exon1:c.G97T:p.G33X
chr12	42538356	42538356	T	C	GXYLT1	synonymous SNV	GXYLT1:NM_001099650:exon1:c.A93G:p.E31E,GXYLT1:NM_173601:exon1:c.A93G:p.E31E
chr13	113201864	113201864	C	T	TUBGCP3	nonsynonymous SNV	TUBGCP3:NM_001286279:exon10:c.G1238A:p.R413H
chr17	42462693	42462693	C	T	ITGA2B	synonymous SNV	ITGA2B:NM_000419:exon5:c.G585A:p.K195K

Supplemental Table 4. Frequencies of *Jak1*$^{H595D/+;I596I/+;Y597Y/+}$ genotypes in pups derived from intercrosses between mosaic-*Jak1*$^{H595D/+;I596I/+;Y597Y/+}$ male mouse and wild-type mice

Jak1$^{H595D/+;I596I/+;Y597Y/+}$	WT	Total
10	65	75

Supplemental Table 5. Differentially expressed genes in the brain at q <0.1

Gene	WT RPKM	*Jak1*$^{H595D/+;I596I/+;Y597Y/+}$ RPKM	Log2FC	GFOLD(0.1)	22q11
Crx	0.003	0.478	5.07	4.26	
Gm21677	0.000	0.183	4.03	3.09	
Tph1	0.059	0.759	3.54	2.99	
Gm21693	0.000	0.183	3.80	2.79	
Gm13871	0.000	0.138	3.83	2.78	
Gm11758	0.000	0.135	3.65	2.64	
Foxg1	3.206	9.349	3.17	2.63	
Gm21704	0.000	0.183	3.67	2.62	
Meox2	0.071	1.142	3.19	2.60	
Gm11757	0.000	0.138	3.59	2.48	
Gm21708	0.000	0.108	3.33	2.40	
Spns3	0.017	0.335	3.09	2.39	
Aox3	0.036	0.480	3.02	2.37	
Ttr	219.946	514.210	2.79	2.29	
Fezf2	0.576	2.946	2.71	2.22	
F2rl1	0.051	0.449	2.82	2.22	
Rbmy	0.000	0.098	3.16	2.08	
Cyp3a11	0.005	0.211	2.97	2.05	
Mup3	0.012	0.478	2.82	2.00	
Cd5l	0.080	0.628	2.55	1.92	
Rbp3	0.080	0.440	2.45	1.88	
Marco	0.032	0.281	2.47	1.85	
Aqp1	1.284	4.585	2.31	1.81	
Emx1	0.459	2.005	2.32	1.77	
Olfr922	0.000	0.182	2.66	1.76	
Olfr456	0.000	0.265	2.68	1.73	
Gm3376	0.000	0.124	2.66	1.67	
1700019B21Rik	0.007	0.252	2.63	1.66	
Mcpt9	0.005	0.144	2.52	1.66	
Gm5878	1.423	3.261	2.11	1.61	
Clic6	0.935	3.092	2.09	1.59	
Tfap2c	0.097	0.430	2.15	1.59	

Pdc	0.000	0.157	2.42	1.54
Gpr31b	0.044	0.355	2.34	1.49
LOC100048884	0.082	0.628	2.26	1.49
Cidec	0.012	0.156	2.33	1.47
Cyp3a41a	0.000	0.045	2.36	1.46
Kcne2	1.237	3.020	2.03	1.46
Wnt8b	0.107	0.419	1.95	1.45
Esm1	0.308	1.236	1.98	1.44
Xlr5a	0.010	0.119	2.18	1.43
Cyp2e1	0.024	0.202	2.15	1.41
Gm4064	0.000	0.091	2.38	1.41
Olfr410	0.050	0.359	2.12	1.39
Tbr1	1.241	3.051	1.89	1.38
Olfr458	0.011	0.274	2.45	1.36
Gm10256	0.000	0.091	2.44	1.32
Cd5	0.043	0.244	2.04	1.32
Serpina1d	0.294	1.137	1.87	1.31
Mup10	0.130	0.760	1.98	1.30
Slc17a7	1.052	3.715	1.76	1.25
Gm2083	0.142	0.818	1.91	1.24
Ces2c	0.005	0.110	2.03	1.21
Dlx1as	8.044	11.806	1.69	1.19
Hbq1b	1.139	7.046	1.71	1.19
Kcnj13	0.693	2.133	1.70	1.19
Dlx2	3.470	6.089	1.66	1.18
Slc4a5	0.187	0.608	1.77	1.17
Mir6935	199.887	412.242	1.65	1.17
Mas1	0.008	0.092	2.07	1.17
Capn13	0.030	0.158	1.99	1.16
Tyrp1	0.028	0.153	1.87	1.15
Bsx	0.227	0.860	1.73	1.15
Gm9992	0.039	0.259	1.97	1.14
Mup13	0.069	0.461	1.99	1.14
Fam167b	0.006	0.164	2.10	1.13
Lhx6	4.098	5.920	1.62	1.12

Avp	20.996	23.293	1.64	1.12	
Car12	0.707	1.793	1.60	1.11	
Mup1	0.120	0.530	1.77	1.10	
Pde6g	0.074	0.426	1.82	1.10	
Apob	0.005	0.027	1.88	1.09	
Cfd	0.045	0.401	2.13	1.08	
Defb11	2.235	4.777	1.65	1.08	
Gm10352	0.000	0.091	2.00	1.07	
Mup8	0.090	0.492	1.79	1.05	
Tmem72	0.221	0.557	1.52	1.01	
Sp110	0.265	0.796	1.55	1.01	
Mir124a-1	0.184	1.820	1.95	1.00	
Cyp2d34	0.013	0.114	1.81	0.99	
Kdm5d	1.081	2.074	1.50	0.99	
Mup2	0.118	0.555	1.57	0.98	
Gm18853	0.062	0.180	1.53	0.97	
Dlx1	12.343	16.702	1.48	0.96	
Sytl3	0.215	0.596	1.48	0.96	
Rab19	0.170	0.664	1.56	0.96	
Slc36a3	0.071	0.264	1.61	0.95	
Mup14	0.152	0.646	1.59	0.95	
Krt6a	0.016	0.100	1.65	0.93	
Gabrd	0.125	0.390	1.52	0.92	+
Gm8979	0.211	0.622	1.43	0.92	
Hp	0.117	0.429	1.63	0.92	
Folr1	1.968	3.944	1.39	0.91	
Dsp	0.049	0.145	1.48	0.91	
Cyp2d11	0.030	0.143	1.63	0.89	
Gm11166	0.045	0.207	1.56	0.88	
Mirlet7c-2	0.330	2.280	1.63	0.88	
Steap4	0.050	0.161	1.51	0.87	
Dlx6os1	2.231	2.829	1.38	0.87	
Gm17757	0.062	0.180	1.48	0.87	
Kcnh3	0.459	1.123	1.34	0.85	
Siglec1	0.035	0.115	1.49	0.84	
Serpina1b	0.407	1.115	1.44	0.83	

Gm2176	0.000	0.048	1.70	0.83
Unc93a	0.038	0.152	1.58	0.83
Siglecg	0.004	0.062	1.69	0.81
4930405D11Rik	0.201	0.731	1.49	0.80
Prr32	0.462	0.911	1.33	0.80
Cldn2	0.349	0.807	1.31	0.79
Gsx2	0.135	0.378	1.37	0.79
Crym	5.263	13.164	1.29	0.78
Lbp	0.482	1.099	1.27	0.78
Lhx8	2.680	2.922	1.28	0.77
Gm11487	0.000	0.086	1.77	0.76
Krt6b	0.019	0.101	1.56	0.76
Prss29	0.097	0.306	1.49	0.75
Umodl1	0.022	0.072	1.41	0.75
Nkx2-1	2.709	3.891	1.20	0.74
Mir6993	1.206	4.284	1.44	0.74
Ces2d-ps	0.008	0.074	1.80	0.73
Pira2	0.011	0.060	1.76	0.73
C5ar1	0.124	0.329	1.35	0.73
Serpina1c	0.484	1.123	1.31	0.73
Gbp2	0.264	0.668	1.23	0.72
Krt8	0.330	0.720	1.26	0.72
Phf11b	0.171	0.479	1.31	0.72
Spic	0.000	0.082	1.68	0.72
Col8a2	1.044	2.336	1.17	0.71
Cyp3a41b	0.000	0.045	1.84	0.71
Dmrt3	0.098	0.298	1.33	0.71
Ttll2	0.035	0.159	1.50	0.71
Eif2s3y	5.575	11.908	1.20	0.71
Foxb2	0.228	0.585	1.28	0.70
Cbr2	0.384	0.768	1.28	0.70
Rtn4rl2	2.375	5.676	1.17	0.69
4930519D14Rik	0.216	0.590	1.32	0.69
Fnd3c2	0.014	0.068	1.45	0.69
Egr1	3.186	6.782	1.17	0.69
Wfikkn2	1.330	2.807	1.26	0.68

Chrm1	0.442	0.963	1.22	0.68
Krt15	0.112	0.324	1.29	0.68
Aebp1	2.793	4.877	1.12	0.68
Serpina1a	0.337	0.856	1.23	0.68
Gm11985	0.005	0.103	1.88	0.67
Fezf1	1.734	2.029	1.21	0.67
Apoa5	0.005	0.053	1.75	0.67
Tfap2d	4.191	6.040	1.13	0.67
Ccdc180	0.180	0.402	1.15	0.67
Oxt	8.410	8.767	1.19	0.66
Nr4a3	1.280	3.117	1.14	0.65
Mup12	0.142	0.491	1.43	0.65
Mir6236	732.84<br>3	#######	1.12	0.65
Cd209g	0.024	0.152	1.52	0.65
Cplx4	0.012	0.088	1.70	0.65
Slc6a20b	2.084	3.207	1.13	0.64
Lox	1.129	2.019	1.16	0.64
Klk4	0.101	0.292	1.30	0.64
Gm6588	0.036	0.116	1.31	0.63
Gm12216	0.000	0.081	1.70	0.63
Gm8989	0.202	0.508	1.20	0.63
Col1a1	5.447	7.853	1.17	0.63
Gm4070	0.599	1.288	1.12	0.63
Gm6614	0.086	0.238	1.28	0.62
Rbm47	0.133	0.310	1.17	0.60
Hist1h1d	0.559	1.329	1.16	0.60
Tnip3	0.001	0.016	1.52	0.60
Bhmt2	0.005	0.044	1.69	0.60
Cybb	0.130	0.300	1.15	0.60
Egr4	0.144	0.344	1.18	0.60
Mup11	0.132	0.486	1.31	0.60
Gm29684	0.050	0.146	1.24	0.60
Gvin1	0.599	1.287	1.09	0.60
Tal2	0.265	0.694	1.22	0.60
Smim6	0.016	0.148	1.65	0.59

Vwf	1.775	3.741	1.06	0.59
BB283400	0.000	0.036	1.63	0.59
Dlx5	3.669	4.923	1.08	0.59
Adh1	0.204	0.464	1.19	0.59
Hpd	0.000	0.053	1.60	0.58
Fmod	4.231	6.086	1.05	0.58
Alx3	0.101	0.282	1.21	0.58
H2-M10.2	0.475	1.177	1.19	0.57
Sh3tc1	0.277	0.601	1.09	0.57
Pitx3	0.201	0.472	1.15	0.57
Hist1h1c	5.752	10.129	1.07	0.57
Krt16	0.023	0.119	1.40	0.56
Slc16a3	2.075	4.607	1.05	0.56
Urah	0.031	0.199	1.50	0.56
Pde6c	0.019	0.077	1.37	0.56
Atp2a3	0.546	1.160	1.01	0.56
Osr2	0.000	0.048	1.62	0.55
Slc13a4	1.402	2.420	1.10	0.55
F5	0.057	0.137	1.18	0.55
Vax2os	0.005	0.030	1.43	0.55
Nr2e1	0.947	1.831	1.03	0.55
Tmc8	0.165	0.365	1.09	0.55
1700030M09Rik	0.005	0.107	1.70	0.55
Trim72	0.040	0.121	1.25	0.54
Defb2	0.980	2.070	1.17	0.53
4930526L06Rik	0.031	0.162	1.36	0.53
4930544G11Rik	0.004	0.064	1.52	0.53
Gm14482	0.046	0.122	1.35	0.53
Foxd2	0.172	0.374	1.10	0.53
Gm13286	0.057	0.301	1.51	0.52
Mat1a	0.013	0.050	1.23	0.52
Slco1a6	0.082	0.199	1.12	0.52
Cxcr2	0.014	0.064	1.43	0.51
Acox2	0.020	0.071	1.25	0.51
Sp100	0.220	0.507	1.06	0.51
1700012D01Rik	0.173	0.475	1.17	0.49

Grn	96.405	197.440	0.96	0.49
Tlr4	0.171	0.351	0.98	0.49
Gc	0.049	0.154	1.17	0.49
Lce1m	0.000	0.082	1.77	0.49
Zbtb20	0.289	0.601	0.99	0.49
Lrrc36	0.153	0.318	1.00	0.48
Dmrta2	0.495	0.980	0.97	0.48
Arhgap33os	0.028	0.093	1.25	0.48
Lrg1	0.066	0.191	1.22	0.48
Mup19	0.161	0.482	1.21	0.48
Gm14476	0.035	0.088	1.20	0.48
Loxl2	0.821	1.621	0.98	0.48
1700016L04Rik	0.017	0.136	1.42	0.48
Rgs9bp	0.072	0.161	1.07	0.48
Kcng1	0.711	1.545	0.97	0.47
Pirb	0.061	0.163	1.11	0.47
Pira7	0.031	0.100	1.19	0.47
Tgfbi	0.648	1.248	0.97	0.47
Gm13807	0.020	0.096	1.37	0.47
Irf7	0.841	1.787	1.02	0.47
Smad6	0.728	1.496	0.98	0.47
Smok4a	0.115	0.254	1.08	0.46
Cdh16	0.121	0.239	0.99	0.46
Myof	0.199	0.377	0.97	0.46
Neurog2	0.759	1.628	0.99	0.46
Serpina3k	0.035	0.125	1.22	0.46
Rab20	0.275	0.629	1.10	0.46
Ms4a1	0.008	0.065	1.64	0.46
Rprml	1.692	3.435	0.95	0.46
Spink8	0.046	0.243	1.34	0.46
Klhl30	0.007	0.034	1.31	0.46
Gprc5a	0.083	0.184	1.13	0.46
Lrp5	1.332	2.614	0.91	0.46
Lhx2	7.400	13.449	0.93	0.46
Kcng4	0.161	0.347	1.11	0.45
Pitx2	2.041	3.018	0.95	0.45

Krt19	1.781	2.892	0.93	0.45	
Patl2	0.155	0.331	1.01	0.45	
Slc6a20a	6.677	9.917	0.96	0.44	
Adamtsl4	0.145	0.302	0.98	0.44	
Mpped1	9.233	17.849	0.91	0.44	
Tas2r137	0.000	0.082	1.35	0.44	
Fndc3c1	0.028	0.090	1.18	0.44	
Egr3	0.300	0.592	0.97	0.43	
Krt18	0.956	1.790	0.98	0.43	
Sp9	2.252	4.103	0.91	0.43	
Neurod4	0.275	0.524	0.93	0.43	
Trp73	0.415	0.777	0.96	0.43	
Dio2	0.291	0.557	0.91	0.43	
Ddn	1.516	3.045	0.95	0.43	
Fam83f	0.113	0.241	0.98	0.43	
Tlr9	0.074	0.172	1.10	0.43	
Mixl1	0.018	0.059	1.13	0.43	
9530052E02Rik	0.553	1.157	0.98	0.42	
Fam83b	0.025	0.069	1.12	0.42	
Pax7	2.024	2.847	0.87	0.42	
Cdhr3	0.163	0.360	0.99	0.42	
5430402O13Rik	0.301	0.707	1.00	0.42	
Mmp25	0.253	0.508	0.95	0.41	
Tmprss6	0.085	0.184	1.04	0.41	
Mob3b	0.245	0.487	0.91	0.41	
Filip1l	0.569	1.069	0.89	0.41	
Lmo7	0.921	1.690	0.91	0.40	
2210409E12Rik	0.014	0.192	1.42	0.40	
Olfr257	0.044	0.150	1.33	0.40	
Nfix	11.341	22.331	0.88	0.40	+
Hist1h1e	0.648	1.319	0.96	0.40	
Stra6l	0.080	0.186	0.99	0.40	
Fosb	0.165	0.306	0.90	0.40	
Cd209f	0.033	0.158	1.32	0.40	
Sema3b	0.598	1.154	0.93	0.40	
B3gnt3	0.196	0.397	0.97	0.40	

1700034G24Rik	0.150	0.343	1.04	0.39
Lars2	125.076	255.495	0.92	0.39
Col8a1	1.271	2.495	0.90	0.39
Upk1b	0.071	0.181	1.09	0.39
Oas1a	0.601	1.109	0.90	0.39
Gm11762	0.184	0.369	0.93	0.39
Sag	0.380	0.798	0.97	0.38
Xlr5b	0.014	0.118	1.23	0.38
Hist2h3b	1.509	3.020	0.93	0.38
Wisp1	0.265	0.427	0.92	0.38
Efcab6	0.180	0.345	0.89	0.38
Oas1g	0.364	0.718	0.93	0.38
C4b	0.272	0.537	0.90	0.38
1700097N02Rik	0.291	0.623	0.97	0.38
Col1a2	11.123	14.526	0.90	0.38
Olfr224	0.653	1.037	0.93	0.38
Sned1	0.693	1.261	0.86	0.38
Osr1	0.993	1.451	0.84	0.37
Tcf7l2	36.570	51.405	0.87	0.37
Itih4	0.010	0.036	1.29	0.37
Cyp26a1	0.018	0.087	1.49	0.37
Cd300lg	0.019	0.076	1.22	0.37
AI506816	0.122	0.293	0.97	0.37
Gm14477	0.035	0.088	1.09	0.37
Olfr285	0.044	0.150	1.21	0.37
Wdr63	0.444	0.853	0.90	0.36
Mageb1	0.009	0.090	1.48	0.36
Mageb2	0.016	0.104	1.31	0.36
4933429O19Rik	0.055	0.158	1.03	0.36
Ccdc154	0.007	0.047	1.45	0.36
Olfr574	0.000	0.079	1.36	0.36
Sry	0.946	1.884	0.87	0.36
Irf1	0.336	0.675	0.88	0.35
AI661453	0.276	0.519	0.86	0.35
Irs2	3.336	6.229	0.83	0.35

Aox1	0.060	0.134	1.05	0.35
A630019I02Rik	0.021	0.059	1.10	0.35
Kif26a	2.898	5.518	0.85	0.34
Robo3	0.574	1.039	0.83	0.34
Slc6a12	0.626	0.869	0.87	0.34
LOC102634401	0.006	0.081	1.34	0.34
Armc4	0.098	0.199	0.95	0.34
Gm5415	0.241	0.484	0.89	0.34
Bcl11b	4.720	8.788	0.78	0.34
Rab44	0.047	0.095	0.92	0.34
Islr2	50.700	88.915	0.80	0.34
Inf2	0.526	0.977	0.84	0.34
Arsi	0.689	1.042	0.87	0.34
Sh3bp1	0.872	1.596	0.87	0.33
Cytip	0.024	0.054	0.97	0.33
Neurod2	7.028	12.530	0.80	0.33
Gm12130	0.000	0.025	1.31	0.33
Nlrp3	0.052	0.115	0.93	0.33
Spata31d1a	0.005	0.019	1.17	0.33
Celsr1	1.058	2.030	0.82	0.33
Cldn9	0.080	0.186	1.02	0.33
Gm15441	6.747	11.670	0.81	0.33
Gm11651	0.845	1.701	0.89	0.33
Cngb3	0.030	0.070	0.99	0.33
Txnip	18.664	32.290	0.84	0.33
Itga11	0.188	0.355	0.83	0.33
Olfr286	0.058	0.171	1.26	0.32
Hrg	0.006	0.053	1.33	0.32
Csf2rb2	0.108	0.200	0.86	0.32
Dbx1	0.304	0.601	0.88	0.32
Kcnn4	0.164	0.324	0.91	0.32
4933400F21Rik	0.007	0.037	1.38	0.32
Dlx6	2.991	3.920	0.83	0.32
Gucy2e	0.240	0.443	0.79	0.32
Parp4	0.189	0.341	0.79	0.32
Col4a2	11.458	21.036	0.80	0.32

Mir6996	0.428	1.691	1.14	0.32
Gm20743	0.066	0.156	1.05	0.32
Arx	4.451	7.038	0.82	0.32
Tram2	0.256	0.527	0.90	0.31
Il6ra	0.250	0.469	0.84	0.31
Mpzl2	0.486	0.632	0.82	0.31
Cpz	0.137	0.247	0.92	0.31
Col4a3	0.151	0.265	0.83	0.31
Padi1	0.018	0.055	1.18	0.31
Slc6a3	1.966	2.826	0.77	0.31
Lgals3bp	2.186	3.996	0.83	0.31
A330074K22Rik	0.275	0.518	0.85	0.31
Laptm5	9.377	16.112	0.73	0.31
Plin4	0.110	0.204	0.86	0.31
Oasl1	0.076	0.175	0.97	0.31
Ror2	0.745	1.341	0.78	0.31
Plxnd1	3.227	5.761	0.77	0.31
Uty	0.878	1.198	0.81	0.31
Pde8a	0.699	1.361	0.83	0.30
Tmem52b	0.000	0.022	1.28	0.30
4933432I03Rik	0.094	0.224	0.99	0.30
Cdc20b	0.332	0.635	0.88	0.30
Map3k7cl	0.202	0.362	0.90	0.30
Itprip	0.791	1.512	0.81	0.30
Bmp6	3.333	4.845	0.72	0.30
Lpl	2.211	4.264	0.80	0.30
Scnn1g	0.007	0.038	1.12	0.30
Podxl	3.805	6.887	0.78	0.30
Col7a1	0.122	0.218	0.79	0.30
Foxo6	6.183	10.889	0.79	0.30
BC061212	0.000	0.019	1.34	0.30
Gfra3	0.100	0.217	0.95	0.30
4930594O21Rik	0.000	0.036	1.37	0.29
Tssk3	0.283	0.529	0.86	0.29
Slco1a4	0.722	1.257	0.76	0.29
Actl10	0.077	0.206	1.12	0.29

Usp43	0.369	0.670	0.81	0.29
Mup9	0.154	0.386	1.02	0.29
A230020J21Rik	0.805	1.520	0.79	0.29
Ccno	0.596	1.072	0.80	0.29
Gpr152	0.009	0.029	1.16	0.29
Hk3	0.232	0.461	0.84	0.29
Prelp	1.929	3.503	0.79	0.28
Hist2h3c2	1.973	3.449	0.75	0.28
Plxna4os1	0.022	0.059	1.07	0.28
Lect1	0.579	1.056	0.84	0.28
Epb4.1l4b	0.784	1.419	0.73	0.28
Col5a1	1.488	2.437	0.77	0.28
Ptgds	315.184	259.313	0.78	0.28
Gm4788	2.793	3.505	0.75	0.28
Aox2	0.011	0.035	1.08	0.28
Fbxo24	0.092	0.205	0.95	0.28
Tbc1d2	0.216	0.386	0.79	0.28
Piezo1	0.770	1.393	0.77	0.28
Cplx3	0.115	0.234	0.88	0.28
Atxn7l1os2	0.164	0.323	0.88	0.28
Rspo3	1.306	2.230	0.76	0.28
Cyp4f39	0.168	0.307	0.89	0.27
Msx1os	0.886	1.584	0.80	0.27
Hoxb5	19.611	17.914	0.73	0.27
Erich6	0.355	0.616	0.83	0.27
Bhlhe22	6.022	10.165	0.74	0.27
Mir8101	4.755	8.549	0.77	0.27
Gm14475	0.035	0.088	1.09	0.27
Fn1	4.735	7.509	0.71	0.27
Pm20d1	0.058	0.119	0.95	0.27
Cldn6	0.257	0.506	0.85	0.27
A430088P11Rik	0.016	0.053	1.09	0.27
Creb3l2	0.712	1.237	0.76	0.27
Gm14479	0.035	0.088	1.09	0.27
Col4a1	16.353	29.254	0.77	0.27

4933407K13Rik	2.828	4.977	0.73	0.27
Tac4	0.013	0.083	1.32	0.27
Rorc	0.090	0.178	0.87	0.27
1700119H24Rik	0.000	0.053	1.29	0.27
1700007J10Rik	2.361	4.308	0.79	0.27
Gpr151	0.858	1.415	0.73	0.27
Myo15	0.027	0.052	0.88	0.26
Thbd	2.526	3.199	0.78	0.26
Ddx3y	4.262	6.413	0.69	0.26
Fam89a	0.223	0.471	0.97	0.26
Fam19a3	0.021	0.050	1.04	0.26
Pou3f1	3.674	6.380	0.72	0.26
Serpinc1	0.075	0.162	0.95	0.26
Zfp36l3	0.010	0.039	1.15	0.26
Lepr	0.692	0.981	0.77	0.26
Mir7029	0.343	1.691	1.24	0.26
Nphs2	0.214	0.378	0.82	0.26
Duoxa1	0.071	0.187	1.04	0.26
Tnxb	0.163	0.275	0.73	0.26
Adipoq	0.072	0.176	0.99	0.25
Mcidas	0.235	0.425	0.79	0.25
1700019B03Rik	0.097	0.237	0.94	0.25
Mir6904	2.886	5.542	0.90	0.25
Krt76	0.099	0.210	0.90	0.25
Pou6f2	0.695	1.225	0.75	0.25
Ly6i	0.661	1.245	0.85	0.25
Lef1	3.742	5.564	0.71	0.25
Ly6g6e	0.097	0.236	0.92	0.25
Neurod6	10.561	18.209	0.76	0.25
Kcnj4	0.367	0.685	0.78	0.25
Gm9926	0.011	0.036	1.16	0.25
Pet117	2.272	4.100	0.78	0.25
Zfp42	0.058	0.143	1.09	0.25
Ssmem1	0.029	0.097	1.06	0.25
Cd248	3.268	5.858	0.76	0.25
Emilin1	1.730	2.760	0.75	0.25

C130050O18Rik	0.049	0.113	0.92	0.25
Naip6	0.038	0.071	0.81	0.25
Rspo2	1.091	1.952	0.74	0.25
Mir1903	4.246	8.638	0.84	0.24
Gsx1	0.643	1.141	0.77	0.24
Ehd2	1.900	3.059	0.74	0.24
Olfml2a	0.972	1.430	0.72	0.24
Scn5a	0.526	0.834	0.76	0.24
Nid2	3.748	6.040	0.74	0.24
Gpa33	0.016	0.049	1.04	0.24
1700010B08Rik	0.012	0.058	1.08	0.24
Ppp1r1b	1.572	2.736	0.72	0.24
Triobp	5.295	9.117	0.72	0.24
Muc2	0.040	0.077	0.86	0.24
Eps8l2	0.113	0.219	0.79	0.24
Mir7083	1.132	3.266	1.06	0.24
Ntng2	6.752	11.140	0.73	0.24
Mir7071	0.454	1.943	1.15	0.24
Csf2rb	0.156	0.281	0.81	0.24
Ttc28	5.068	8.519	0.69	0.23
Col3a1	10.986	13.821	0.73	0.23
Apoc3	0.021	0.083	1.01	0.23
Tgm2	2.063	3.092	0.69	0.23
Gipr	0.630	1.059	0.70	0.23
Gpr65	0.095	0.183	0.86	0.23
Six6	0.383	0.435	0.78	0.23
Lrrd1	0.043	0.094	0.97	0.23
Efemp1	1.096	1.535	0.70	0.23
Gbgt1	0.470	0.811	0.80	0.22
D6Ertd527e	0.038	0.088	1.07	0.22
Ppp1r13l	0.611	1.078	0.72	0.22
4930445N18Rik	0.000	0.058	1.21	0.22
Icam1	0.782	1.211	0.71	0.22
Slc16a2	8.643	14.408	0.66	0.22
Nrgn	18.309	29.243	0.66	0.22
Apol9a	0.196	0.367	0.82	0.22

Ldlrap1	0.648	1.201	0.77	0.22
Vmn2r63	0.053	0.108	0.88	0.22
Heg1	2.045	3.517	0.71	0.22
Ephb4	1.974	3.441	0.72	0.22
Notum	1.090	1.762	0.65	0.22
AI606473	1.855	1.387	0.66	0.22
Slc16a10	0.240	0.426	0.74	0.22
Klk12	0.208	0.421	0.85	0.22
Osm	0.019	0.076	1.00	0.22
4930567H17Rik	0.062	0.172	1.05	0.21
Gm26705	0.043	0.120	0.99	0.21
Lrrc15	0.088	0.169	0.81	0.21
Pdgfrb	3.283	5.394	0.69	0.21
Spib	0.015	0.041	1.07	0.21
Tifab	0.276	0.457	0.70	0.21
Tex13	0.002	0.034	1.28	0.21
Krt36	0.000	0.037	1.30	0.21
Apol6	0.008	0.031	1.13	0.21
Adamts14	0.162	0.284	0.73	0.21
Veph1	0.385	0.658	0.70	0.21
Map10	1.851	3.020	0.68	0.21
Cxcl12	7.163	11.468	0.67	0.21
Zfp217	0.727	1.232	0.68	0.21
Tnfsf10	0.115	0.204	0.76	0.21
Plbd1	0.061	0.152	0.93	0.21
She	0.501	0.844	0.70	0.21
Slc38a8	0.044	0.114	1.09	0.21
Ptk2b	0.591	0.989	0.70	0.20
Gm13290	0.238	0.398	0.78	0.20
Col4a4	0.111	0.186	0.68	0.20
4930556M19Rik	0.048	0.097	0.82	0.20
Slit3	0.606	0.999	0.67	0.20
Figla	0.021	0.122	1.21	0.20
Slco1a1	0.050	0.107	0.94	0.20
Cldn1	1.169	1.884	0.69	0.20
Gm16894	0.591	1.058	0.78	0.20

Ifi44	0.482	0.816	0.69	0.20
4833418N02Rik	0.458	0.883	0.81	0.20
C7	0.023	0.061	0.97	0.20
6530402F18Rik	2.498	4.090	0.65	0.20
Pon1	0.069	0.211	1.05	0.20
Gm13287	0.124	0.244	0.81	0.20
Gm7978	0.000	0.019	1.03	0.20
G630093K05Rik	0.037	0.077	0.90	0.20
Egr2	0.190	0.351	0.76	0.20
Hcrt	0.743	1.219	0.76	0.20
Gm11190	0.080	0.157	0.83	0.20
Col22a1	0.550	0.943	0.71	0.19
Mir6989	4.341	8.727	0.82	0.19
Igfbpl1	16.914	27.775	0.67	0.19
Lif	0.028	0.064	0.95	0.19
Gm10415	0.000	0.022	1.31	0.19
Msx1	1.383	2.291	0.67	0.19
Apbb1ip	0.455	0.763	0.67	0.19
Spta1	0.116	0.194	0.75	0.19
Tmem171	0.018	0.080	1.21	0.19
2900026A02Rik	2.564	4.141	0.62	0.19
Cntn2	6.573	11.158	0.67	0.19
Kynu	0.026	0.054	0.94	0.19
Odf3b	0.678	1.240	0.79	0.19
Mir8109	0.134	0.617	1.09	0.19
Slc12a7	1.341	2.256	0.65	0.19
H2-T3	0.218	0.412	0.81	0.19
Sla	2.280	3.895	0.66	0.19
Angptl2	2.003	2.851	0.69	0.19
Gckr	0.060	0.133	0.98	0.19
Flt1	2.308	3.860	0.64	0.19
Anpep	1.157	1.877	0.71	0.19
Gm3230	0.145	0.296	0.82	0.18
Zfp109	1.493	2.459	0.67	0.18
Lrp2bp	0.087	0.160	0.81	0.18
LOC102631757	0.003	0.024	1.22	0.18

Col13a1	0.413	0.544	0.72	0.18
Hkdc1	0.143	0.249	0.77	0.18
2210416O15Rik	0.660	1.152	0.74	0.18
Hist2h3c1	1.944	3.402	0.68	0.18
1700123L14Rik	0.037	0.085	0.98	0.18
Gm10681	0.110	0.201	0.73	0.18
Eng	5.843	9.895	0.66	0.18
Arhgap9	0.139	0.250	0.78	0.18
Xpnpep2	0.061	0.112	0.78	0.18
Clcnka	0.019	0.042	0.94	0.18
Mir7082	5.226	9.193	0.76	0.18
Tbx19	0.012	0.046	1.16	0.18
Adrb1	1.083	1.834	0.65	0.18
Ggt5	0.283	0.494	0.71	0.17
Slfn10-ps	0.032	0.072	0.89	0.17
Fyb	0.260	0.419	0.67	0.17
Bpifb4	0.057	0.146	0.96	0.17
Ipw	44.790	70.021	0.63	0.17
AI839979	0.227	0.422	0.77	0.17
Arid5b	1.720	2.924	0.68	0.17
Ar	0.062	0.113	0.74	0.17
Otx1	1.509	2.092	0.71	0.17
Armc3	0.452	0.704	0.68	0.17
Mir6956	5.394	9.482	0.77	0.17
Wnt3a	0.065	0.150	0.96	0.17
Scarf1	0.696	1.184	0.67	0.17
Cyp2a4	0.402	0.607	0.74	0.17
Pou2f3	0.029	0.078	1.01	0.17
Asb16	0.422	0.719	0.72	0.17
Slc22a6	1.562	1.832	0.67	0.17
Pydc3	0.072	0.131	0.83	0.17
Atoh8	0.551	0.872	0.65	0.17
Irf8	0.476	0.826	0.64	0.17
Vmn2r62	0.049	0.112	0.85	0.17
Lin28a	0.074	0.139	0.79	0.17
Mef2c	4.979	8.113	0.65	0.16

Slc6a13	7.895	9.115	0.65	0.16
Fzd4	1.055	1.744	0.65	0.16
Dnm3os	0.053	0.087	0.75	0.16
Noto	0.000	0.032	1.27	0.16
Gm1604b	3.620	5.679	0.63	0.16
Gm13289	0.238	0.398	0.73	0.16
Podn	0.261	0.452	0.68	0.16
Cyp3a44	0.000	0.021	1.21	0.16
Nr4a2	4.601	7.818	0.69	0.16
Scube1	1.725	2.927	0.63	0.16
Gda	2.893	4.099	0.62	0.16
Insm1	4.271	6.454	0.63	0.16
4933403O08Rik	0.093	0.190	0.85	0.16
Igll1	0.006	0.047	1.04	0.16
Six3	3.993	6.202	0.64	0.16
Anxa11	3.812	6.008	0.64	0.16
Tlr8	0.012	0.036	1.04	0.16
Appbp2os	0.122	0.338	0.98	0.16
Bmp7	4.524	6.520	0.62	0.16
Satb2	0.622	1.036	0.65	0.16
Cldn19	0.276	0.472	0.66	0.16
Scube3	2.459	4.206	0.67	0.15
Ano1	0.827	1.360	0.65	0.15
Arsg	1.184	1.884	0.62	0.15
Irx6	0.879	1.301	0.62	0.15
Ackr2	0.802	1.339	0.66	0.15
Clec4a1	0.245	0.391	0.73	0.15
D330046F09Rik	0.000	0.048	1.12	0.15
Mir6925	3.061	6.188	0.82	0.15
Gm6904	0.122	0.264	0.85	0.15
Unc5b	2.178	3.606	0.64	0.15
Emx2os	0.410	0.548	0.62	0.15
Zbtb18	17.861	26.946	0.63	0.15
Cdh5	4.134	6.391	0.63	0.15
Col10a1	0.042	0.092	0.84	0.15
Nfatc1	0.508	0.866	0.67	0.15

Cdsn	0.039	0.077	0.84	0.15
Rasal1	1.801	2.858	0.61	0.15
Arrdc3	18.212	28.637	0.63	0.15
Npc1l1	0.029	0.061	1.01	0.15
Smagp	0.568	0.952	0.74	0.14
Ubl4b	0.015	0.075	1.16	0.14
Hivep3	1.202	1.969	0.66	0.14
Rnf43	0.171	0.287	0.70	0.14
Cfh	13.193	15.846	0.67	0.14
Sostdc1	2.749	4.329	0.63	0.14
Spz1	0.000	0.026	1.22	0.14
Trim34a	0.634	1.019	0.67	0.14
Slco2a1	1.594	2.639	0.65	0.14
Hsd3b4	0.101	0.185	0.76	0.14
Trabd2b	0.292	0.440	0.61	0.14
Ctf2	0.064	0.149	1.01	0.14
Elovl3	0.053	0.132	0.86	0.14
Mir5617	12.469	22.076	0.69	0.14
Cass4	0.058	0.114	0.81	0.14
Itgb6	0.010	0.030	0.97	0.14
Il17ra	0.715	1.138	0.60	0.14
Sema3g	0.810	1.298	0.61	0.14
Nlrp6	0.150	0.267	0.69	0.14
Foxj1	7.099	11.214	0.61	0.14
Rasal3	0.105	0.182	0.68	0.13
Trim34b	0.409	0.658	0.68	0.13
Col2a1	1.631	2.588	0.58	0.13
Per1	4.170	6.696	0.61	0.13
Cfap70	0.309	0.478	0.62	0.13
Blnk	0.174	0.334	0.80	0.13
Cnga3	0.113	0.191	0.68	0.13
Vav1	0.247	0.389	0.63	0.13
Igf2os	0.199	0.287	0.65	0.13
Il17re	0.031	0.072	0.91	0.13
Otop2	0.058	0.110	0.80	0.13
Cldn15	0.048	0.123	0.99	0.13

Foxn4	0.144	0.247	0.74	0.13
Zbp1	0.186	0.348	0.73	0.13
Fndc1	1.033	1.664	0.64	0.13
Dthd1	0.009	0.038	1.15	0.13
Cma1	0.107	0.240	0.93	0.13
Defb10	0.457	0.868	0.81	0.13
1700030C10Rik	1.385	2.226	0.62	0.13
Hspg2	0.707	1.156	0.63	0.13
Cyp2d26	0.114	0.238	0.80	0.13
Wipf3	1.800	2.851	0.59	0.13
Otx2os1	0.879	1.416	0.67	0.13
Ajuba	1.164	1.843	0.62	0.13
Colec12	3.972	5.362	0.62	0.13
Parp14	0.200	0.331	0.69	0.13
Aldh1a2	4.279	5.604	0.58	0.12
Galnt6	0.110	0.186	0.69	0.12
Olfr1431	0.000	0.055	1.17	0.12
Ly6f	0.501	0.906	0.76	0.12
Cmklr1	0.551	0.895	0.65	0.12
2900092D14Rik	3.046	4.868	0.62	0.12
AI427809	0.122	0.226	0.82	0.12
Oasl2	0.900	1.447	0.62	0.12
Tbx15	0.302	0.451	0.70	0.12
A2m	2.732	4.153	0.65	0.12
Mup15	0.203	0.441	0.67	0.12
Helz2	0.234	0.387	0.64	0.12
Xkrx	0.402	0.653	0.65	0.12
Col6a2	4.716	6.389	0.62	0.12
Pira4	0.032	0.102	1.02	0.12
Pou2f2	3.004	4.788	0.61	0.12
Otud1	1.294	2.035	0.60	0.12
5930430L01Rik	0.198	0.329	0.69	0.12
Ccdc80	1.831	2.980	0.66	0.12
Olfr520	0.000	0.043	1.21	0.12
4921507L20Rik	0.145	0.293	0.81	0.12
Apol10b	0.005	0.047	1.06	0.12

Tmem102	0.652	1.031	0.64	0.12
Tbx21	0.045	0.097	0.86	0.12
Wfs1	3.585	5.351	0.59	0.12
Vmo1	0.117	0.276	0.95	0.12
Cdh1	1.246	1.737	0.62	0.11
Mrvi1	0.195	0.312	0.63	0.11
Ptafr	0.092	0.181	0.80	0.11
Trim25	1.296	2.028	0.58	0.11
BC024386	0.000	0.032	1.06	0.11
Bcl9l	8.388	13.166	0.58	0.11
Pkhd1l1	0.020	0.036	0.77	0.11
Bank1	0.063	0.111	0.76	0.11
Pard3	2.601	3.910	0.56	0.11
Olfr235	0.000	0.055	0.99	0.11
Synpo2	0.438	0.661	0.63	0.11
Cd28	0.036	0.074	0.77	0.11
Lhx9	8.983	11.574	0.59	0.11
Mroh8	0.927	1.439	0.59	0.11
Cbfa2t3	3.195	4.911	0.58	0.11
Ezr	11.422	18.193	0.59	0.11
Lrrk1	0.384	0.576	0.59	0.11
Pyroxd2	0.546	0.883	0.62	0.11
Pcolce	3.205	4.504	0.62	0.11
1700110K17Rik	1.247	2.040	0.63	0.11
Cr2	0.063	0.116	0.74	0.11
Lamc3	0.793	1.181	0.60	0.11
Tead4	0.182	0.302	0.66	0.11
Hpx	0.064	0.139	0.76	0.11
Loxl1	1.339	2.132	0.61	0.11
Cldn22	0.084	0.166	0.88	0.11
Acvrl1	2.508	3.875	0.59	0.10
Maml1	1.818	2.876	0.59	0.10
Dnaic2	0.919	1.369	0.62	0.10
A930011G23Rik	0.380	0.637	0.65	0.10
Gm13285	0.180	0.282	0.64	0.10
Foxo3	4.477	6.919	0.53	0.10

Ccdc60	0.886	1.406	0.58	0.10
Slc26a10	0.201	0.340	0.75	0.10
Kdr	4.665	7.612	0.62	0.10
Mir5129	3.216	6.083	0.75	0.10
Emx2	2.444	3.438	0.62	0.10
Serpina1e	0.381	0.526	0.69	0.10
Ranbp3l	1.079	1.304	0.61	0.10
Ppl	0.030	0.059	0.74	0.10
Gm3716	0.045	0.083	0.78	0.10
Rtn4rl1	5.734	9.008	0.57	0.10
Gm12359	2.924	4.557	0.58	0.10
Itpr3	0.276	0.447	0.58	0.10
Cldn14	0.021	0.052	0.95	0.10
Actl7a	0.000	0.034	1.27	0.10
Scn11a	0.065	0.110	0.73	0.10
Gm13830	0.339	0.553	0.63	0.10
Tap2	0.525	0.845	0.62	0.09
Trim56	0.342	0.564	0.62	0.09
Tiam2	1.096	1.705	0.58	0.09
Phf11c	0.138	0.292	0.74	0.09
C630031E19Rik	0.003	0.017	1.04	0.09
Islr	6.775	8.562	0.54	0.09
Fbn2	0.959	1.425	0.56	0.09
Ttc21a	0.502	0.784	0.58	0.09
Plekhg3	0.524	0.812	0.59	0.09
Vmn2r55	0.002	0.013	1.13	0.09
Hhipl1	0.967	1.477	0.56	0.09
9630028B13Rik	0.497	0.745	0.62	0.09
Klhl35	0.327	0.539	0.64	0.09
Dusp4	11.692	17.862	0.58	0.09
Ccdc88b	0.197	0.327	0.63	0.09
Crtc3	2.142	3.365	0.57	0.09
Eno4	0.576	0.868	0.62	0.09
Olfml2b	0.940	1.500	0.64	0.09
Ston1	1.137	1.774	0.59	0.09
Klf5	0.745	1.130	0.60	0.09

Klf3	3.869	5.857	0.57	0.09
St6galnac5	8.480	13.152	0.56	0.09
Rarres1	0.522	0.855	0.63	0.09
Plekhg2	2.180	3.216	0.55	0.09
Cd33	0.402	0.630	0.65	0.09
Adad1	0.008	0.042	1.04	0.09
Efnb1	5.635	8.824	0.55	0.09
Grin2b	1.530	2.468	0.57	0.09
Ccdc85c	6.493	10.561	0.59	0.09
Ahdc1	5.945	9.244	0.56	0.09
Mfsd7c	1.077	1.645	0.55	0.09
Abhd15	0.264	0.425	0.60	0.09
3110039M20Rik	0.716	0.930	0.66	0.09
Kcp	1.051	1.627	0.57	0.09
Magix	0.034	0.064	0.76	0.09
Sox14	4.413	6.198	0.59	0.09
Ntsr1	2.116	3.166	0.55	0.09
Fosl2	1.429	2.370	0.62	0.09
Mirlet7i	0.061	0.485	1.15	0.09
Col20a1	0.095	0.160	0.62	0.09
F13a1	2.661	3.351	0.59	0.09
Pappa2	0.394	0.633	0.59	0.09
Mir675	41.620	48.992	0.53	0.09
Prrt1	7.569	11.983	0.56	0.09
2310002L09Rik	0.002	0.033	1.27	0.09
Pydc4	0.025	0.062	0.90	0.08
Chst14	1.039	1.651	0.60	0.08
C2cd4d	0.453	0.737	0.64	0.08
Ccdc13	0.942	1.528	0.59	0.08
Mup16	0.142	0.316	0.81	0.08
Grid2ip	0.389	0.606	0.60	0.08
Ssc5d	0.407	0.593	0.59	0.08
Sfrp5	0.630	1.021	0.60	0.08
Mier1	10.026	20.817	0.61	0.08
BC049762	0.059	0.140	0.94	0.08
Paqr6	1.198	1.717	0.58	0.08

Abcc3	0.106	0.184	0.66	0.08
Adgre5	2.145	3.282	0.55	0.08
Col12a1	0.679	1.002	0.54	0.08
Ptrf	7.110	10.702	0.56	0.08
1700101E01Rik	0.702	1.141	0.63	0.08
Slc2a1	23.498	37.026	0.58	0.08
Akr1c6	0.015	0.061	0.87	0.08
Wdr86	1.718	2.535	0.57	0.08
Naip7	0.049	0.092	0.72	0.08
Alx4	0.615	0.957	0.58	0.08
Synpo	0.612	1.003	0.62	0.08
Fbln5	1.231	1.629	0.55	0.08
1700028E10Rik	0.037	0.083	0.87	0.08
2610027K06Rik	0.586	0.936	0.60	0.08
Tgfbr3	0.903	1.246	0.56	0.08
Uaca	3.577	5.565	0.55	0.08
Rimbp3	0.467	0.744	0.59	0.08
Fbln1	3.963	6.000	0.57	0.08
Sult2a8	0.000	0.018	1.01	0.08
D2hgdh	6.827	10.948	0.57	0.08
Nphs1	0.067	0.113	0.69	0.08
Lilra6	0.012	0.048	1.10	0.08
Fut4	0.408	0.653	0.58	0.07
Acer2	2.666	4.148	0.55	0.07
Col6a1	4.319	5.494	0.52	0.07
Chrm5	0.667	1.075	0.60	0.07
Ahnak	0.693	1.062	0.54	0.07
Tgfbr2	2.392	3.701	0.55	0.07
Pate2	0.144	0.242	0.66	0.07
Amhr2	0.054	0.101	0.85	0.07
Kcne1	0.091	0.163	0.75	0.07
Irf2bpl	24.754	38.211	0.54	0.07
Gdf10	8.079	10.351	0.54	0.07
Mir6987	4.653	8.195	0.69	0.07
Gpr3	0.736	1.208	0.65	0.07
Sox4	55.070	85.786	0.55	0.07

Tbx18	0.953	1.272	0.59	0.07
Mir1966	2.710	4.966	0.72	0.07
Igf2	56.979	68.150	0.49	0.07
Irg1	0.000	0.012	1.14	0.07
Adamts18	0.759	1.236	0.58	0.07
Atp8b4	0.033	0.065	0.76	0.07
Lcp1	2.965	4.718	0.58	0.07
Crocc	1.904	2.938	0.55	0.07
Bend3	1.358	2.017	0.53	0.07
Ap5b1	0.788	1.231	0.58	0.07
Csf3r	0.200	0.306	0.50	0.07
Foxp4	13.199	20.131	0.51	0.07
Lsr	4.242	6.415	0.54	0.06
D8Ertd82e	4.748	7.157	0.53	0.06
Lce1g	0.017	0.089	1.00	0.06
Tspan11	0.756	1.186	0.57	0.06
Gltscr1	2.615	4.235	0.57	0.06
9030612E09Rik	0.591	0.917	0.54	0.06
Maff	1.704	2.514	0.53	0.06
Mn1	5.932	9.247	0.56	0.06
Atp13a5	1.152	1.647	0.56	0.06
Disc1	0.491	0.778	0.56	0.06
En1	4.012	5.949	0.55	0.06
Adrb3	0.185	0.314	0.63	0.06
Tgtp1	0.102	0.173	0.67	0.06
Grm2	4.202	6.126	0.53	0.06
Prox1	3.273	4.759	0.55	0.06
1700049L16Rik	0.182	0.346	0.75	0.06
Gm853	0.031	0.065	0.87	0.06
Spen	3.814	6.093	0.53	0.06
Mir1224	3.013	5.218	0.68	0.06
Mansc1	0.501	0.764	0.57	0.06
Dlx6os2	0.367	0.548	0.64	0.06
Fbln2	2.225	3.400	0.51	0.06
Gal3st3	8.393	13.270	0.58	0.06
Slc7a5	26.641	40.326	0.51	0.06

Hunk	3.714	5.669	0.51	0.06
Apol9b	0.216	0.369	0.68	0.06
Cyp2d22	0.833	1.317	0.59	0.06
Mir5131	0.843	1.885	0.79	0.06
Muc5b	0.034	0.051	0.62	0.06
5830432E09Rik	0.185	0.338	0.71	0.06
Zfhx2os	2.767	4.077	0.51	0.06
Mef2d	7.687	12.051	0.54	0.06
Lamb2	2.764	4.478	0.58	0.06
Maml3	2.420	3.638	0.53	0.06
Adamts12	0.825	1.158	0.57	0.06
Gpr20	0.061	0.111	0.83	0.06
Rara	2.610	3.930	0.50	0.06
Ccdc33	0.089	0.155	0.62	0.06
Cdh3	0.489	0.789	0.61	0.06
Itga5	0.972	1.462	0.54	0.06
Wnt6	0.675	0.713	0.58	0.05
Aldh1a1	8.510	9.669	0.55	0.05
Zc3h12a	0.101	0.188	0.70	0.05
Mvp	0.627	0.958	0.55	0.05
Tspan18	6.584	9.390	0.48	0.05
Ermap	0.123	0.209	0.60	0.05
Zfp366	0.604	0.916	0.57	0.05
Xkr7	4.534	7.057	0.51	0.05
Slc12a8	0.221	0.337	0.62	0.05
Hdgfl1	0.183	0.300	0.61	0.05
Tgtp2	0.108	0.170	0.61	0.05
Tlr12	0.323	0.500	0.59	0.05
Npr3	0.515	0.743	0.50	0.05
Rfx2	0.935	1.397	0.50	0.05
Cyp2d12	0.010	0.057	1.06	0.05
Tnfrsf14	0.047	0.139	0.97	0.05
Klhdc7a	0.250	0.356	0.58	0.05
Scara3	1.770	2.741	0.57	0.05
Olfr90	0.000	0.049	1.07	0.05
Syde2	0.508	0.753	0.51	0.05

Glp1r	0.272	0.474	0.65	0.05
Mpo	0.016	0.049	1.01	0.05
Plod1	3.521	5.335	0.54	0.05
1700125H20Rik	0.177	0.286	0.71	0.05
Hoxa5	23.587	18.447	0.51	0.05
Lnx2	0.997	1.517	0.54	0.05
Sowahd	0.046	0.111	0.79	0.05
Npr1	0.546	0.832	0.56	0.05
Wasf2	2.810	4.335	0.53	0.05
Slc7a8	3.732	5.515	0.51	0.05
Thsd4	0.922	1.239	0.50	0.05
Soga1	11.176	16.686	0.50	0.05
Arhgap31	1.745	2.556	0.50	0.05
Lpin3	0.064	0.129	0.70	0.05
Robo4	1.478	2.242	0.55	0.05
Glis3	0.508	0.796	0.56	0.05
Adora2a	1.634	2.590	0.60	0.05
Tjp3	0.984	1.472	0.52	0.05
Bmp5	0.841	1.187	0.55	0.05
Krt85	0.159	0.286	0.69	0.05
Cgnl1	1.455	2.153	0.53	0.04
Cstl1	0.026	0.152	1.12	0.04
Slc52a3	0.586	0.820	0.50	0.04
Gm10532	4.431	6.957	0.52	0.04
Htr3a	0.685	0.961	0.51	0.04
Abca8a	0.290	0.465	0.60	0.04
Ankrd63	0.080	0.119	0.65	0.04
Slc11a1	0.397	0.640	0.57	0.04
Serpinf1	3.799	4.853	0.51	0.04
Draxin	10.633	15.789	0.50	0.04
Defb1	0.000	0.045	1.18	0.04
Tns2	1.348	2.044	0.51	0.04
Cx3cr1	2.932	4.447	0.50	0.04
Dscaml1	3.407	4.990	0.52	0.04
Prdm9	1.630	2.516	0.57	0.04
Pde2a	8.567	12.179	0.45	0.04

Slc38a11	0.309	0.514	0.64	0.04
Slc26a2	0.686	1.015	0.56	0.04
Stra6	1.167	1.804	0.56	0.04
C230072F16Rik	0.682	1.032	0.57	0.04
6430584L05Rik	0.202	0.327	0.65	0.04
Myh9	4.108	6.170	0.50	0.04
5430416O09Rik	0.022	0.045	0.73	0.04
Slc22a12	0.155	0.247	0.63	0.04
Bcorl1	2.752	4.045	0.50	0.04
Flrt1	1.347	2.004	0.52	0.04
Socs3	1.504	2.253	0.52	0.04
Zfp516	1.487	2.328	0.55	0.04
Gpr25	2.149	3.352	0.56	0.04
Gm4984	0.017	0.088	1.15	0.04
Pecam1	5.216	7.801	0.50	0.04
Espnl	0.060	0.106	0.72	0.04
Itih2	0.969	1.008	0.53	0.04
Cyp1b1	1.449	1.965	0.54	0.04
Ucn2	0.000	0.041	1.09	0.04
Krtap6-1	0.000	0.035	1.13	0.04
Zdhhc23	1.268	2.042	0.60	0.04
Birc3	0.439	0.660	0.56	0.04
Mef2b	0.142	0.250	0.71	0.04
D830031N03Rik	0.677	1.005	0.50	0.04
Dmbt1	0.004	0.013	1.11	0.04
Rel	0.182	0.291	0.65	0.04
Mir7073	5.144	9.025	0.66	0.04
Heyl	1.425	2.085	0.52	0.03
D930015M05Rik	1.221	1.889	0.55	0.03
Gabrr2	0.063	0.125	0.80	0.03
Sprr2a3	0.000	0.024	1.09	0.03
Arhgap30	0.196	0.306	0.58	0.03
Auts2	9.444	14.044	0.50	0.03
Tor3a	0.697	1.013	0.48	0.03
Fcgr2b	1.402	1.933	0.52	0.03
Foxa2	0.728	0.966	0.57	0.03

Slfn8	0.066	0.131	0.64	0.03
1700001C19Rik	0.229	0.372	0.59	0.03
C1s1	0.277	0.425	0.57	0.03
Pira11	0.045	0.103	0.85	0.03
Ccdc146	0.319	0.469	0.56	0.03
Bst2	5.328	7.662	0.54	0.03
Gramd2	0.814	1.244	0.56	0.03
Prok2	0.173	0.300	0.62	0.03
Vasn	1.812	2.756	0.52	0.03
Camk2a	9.136	13.155	0.45	0.03
Map3k19	0.499	0.755	0.55	0.03
Cyp3a25	0.000	0.026	1.15	0.03
Tinagl1	1.163	1.693	0.54	0.03
Slain1	8.565	12.821	0.51	0.03
Apc2	31.432	46.209	0.49	0.03
2010300C02Rik	1.863	2.651	0.49	0.03
Sox1	5.556	7.810	0.50	0.03
Lrguk	0.282	0.445	0.60	0.03
Ifnk	0.045	0.126	0.97	0.03
Atn1	27.783	41.416	0.50	0.03
Best3	0.023	0.057	0.88	0.03
C530008M17Rik	16.786	25.030	0.47	0.03
Cldn3	1.039	1.494	0.57	0.02
Car9	0.243	0.379	0.59	0.02
Mir6921	10.386	16.245	0.58	0.02
Nes	7.430	10.813	0.48	0.02
Sox11	18.988	27.333	0.47	0.02
Prr12	7.088	10.389	0.48	0.02
4930512B01Rik	0.566	0.907	0.57	0.02
Spag16	0.297	0.434	0.51	0.02
Brwd3	1.157	1.661	0.49	0.02
Snai1	0.409	0.637	0.59	0.02
E130012A19Rik	1.478	2.275	0.52	0.02
Mir6944	50.353	76.281	0.49	0.02
Slfn5	0.761	1.138	0.52	0.02
Npy6r	0.010	0.040	1.16	0.02

5930403L14Rik	1.827	2.714	0.49	0.02
Scarf2	1.162	1.734	0.54	0.02
Gpc2	37.325	55.039	0.51	0.02
Rab42	0.839	1.249	0.55	0.02
Mirlet7f-1	0.059	0.464	1.20	0.02
Lzts1	2.645	3.971	0.53	0.02
AI607873	0.152	0.219	0.53	0.02
Gm28979	0.007	0.040	1.15	0.02
2610015P09Rik	0.680	0.985	0.51	0.02
Fzd8	1.488	2.272	0.54	0.02
Hfe	1.029	1.557	0.51	0.02
Dsc3	0.264	0.426	0.61	0.02
Ribc1	0.257	0.411	0.68	0.02
Igf2bp2	2.002	3.053	0.53	0.02
Aox4	0.103	0.170	0.64	0.02
Col4a6	0.784	1.012	0.47	0.02
Ccdc177	2.883	4.293	0.51	0.02
4930523C07Rik	0.288	0.442	0.54	0.02
Prrc2a	35.803	54.667	0.50	0.02
Trpm3	2.959	4.329	0.49	0.02
Wnt9a	0.758	1.150	0.52	0.02
Spef2	0.247	0.360	0.54	0.02
4931431C16Rik	0.329	0.475	0.56	0.02
4930429B21Rik	0.876	1.248	0.50	0.02
Igsf8	19.419	28.530	0.50	0.02
Slc37a2	0.343	0.525	0.50	0.02
Synb	0.002	0.021	1.18	0.02
Vmn2r18	0.083	0.151	0.70	0.02
Ankfn1	1.014	1.572	0.54	0.02
Prdm13	0.486	0.611	0.52	0.01
Zbtb7a	2.476	3.697	0.50	0.01
Fam163a	1.121	1.680	0.50	0.01
Capn9	0.200	0.332	0.62	0.01
H2-Q4	4.832	7.219	0.52	0.01
Shisa3	1.963	2.193	0.52	0.01
Ly9	0.077	0.139	0.77	0.01

Car14	2.094	2.977	0.49	0.01	
Ltbp4	2.967	4.168	0.52	0.01	
Sptbn2	20.539	29.707	0.46	0.01	
Chrnb1	0.263	0.403	0.58	0.01	
Bcor	3.334	4.955	0.51	0.01	
Pck1	0.026	0.055	0.80	0.01	
Gpr83	0.620	0.930	0.52	0.01	+
Tmem200c	1.698	2.507	0.48	0.01	
St8sia2	32.898	48.122	0.48	0.01	
Zc3hav1	0.314	0.465	0.54	0.01	
Elk3	2.201	3.140	0.43	0.01	
Sh3bp4	5.707	8.523	0.52	0.01	
Inhba	0.260	0.411	0.56	0.01	
Jup	8.334	12.742	0.53	0.01	
Crispld2	0.710	1.046	0.54	0.01	
Zfp319	6.596	9.679	0.47	0.01	
Arhgap33	46.144	69.019	0.48	0.01	
Zfp628	3.071	4.440	0.49	0.01	
E230013L22Rik	0.073	0.128	0.66	0.01	
Foxa1	1.208	1.534	0.49	0.01	
Gm10324	0.014	0.048	0.82	0.01	
Pou4f1	3.680	5.175	0.46	0.01	
Cobll1	0.871	1.319	0.51	0.01	
Wnt9b	0.509	0.762	0.53	0.01	
Irgm2	0.610	0.857	0.46	0.01	
H2-M2	0.547	0.884	0.57	0.01	
Osmr	0.565	0.826	0.49	0.01	
Cnih3	5.122	7.496	0.48	0.01	
Kcnk15	0.132	0.250	0.75	0.01	
Dlec1	0.278	0.406	0.50	0.01	
L1td1	0.028	0.056	0.72	0.01	
Tlr2	0.289	0.434	0.53	0.01	
Dtx3l	0.372	0.547	0.51	0.01	
Tnrc18	9.473	15.031	0.52	0.01	
Fbrs	8.422	12.069	0.47	0.01	
Ccr5	0.289	0.451	0.56	0.01	

Nfatc2	0.594	0.890	0.48	0.00
Snx29	3.148	4.592	0.50	0.00
Bcl9	9.276	13.335	0.44	0.00
H19	69.080	76.716	0.52	0.00
Gm13497	0.000	0.020	1.10	0.00
2900005J15Rik	0.234	0.381	0.57	0.00
Serpine1	0.548	0.809	0.53	0.00
Kctd19	0.026	0.051	0.69	0.00
Ltb4r2	0.211	0.366	0.64	0.00
Azgp1	0.004	0.039	0.95	0.00
Ceacam19	0.006	0.025	1.03	0.00
Akna	0.939	1.362	0.50	0.00
Krt81	0.051	0.106	0.76	0.00
Bicc1	0.997	1.415	0.46	0.00
Usp35	2.055	3.023	0.47	0.00
Sec31a	10.532	15.596	0.49	0.00
Hic2	1.798	2.558	0.47	0.00
Dbil5	0.273	0.502	0.74	0.00
Gapt	0.043	0.090	0.87	0.00
Sec14l5	0.078	0.128	0.71	0.00
Prlh	0.040	0.316	0.97	0.00
Cfap53	0.248	0.362	0.51	0.00
Mir6908	9.954	16.044	0.62	0.00
Scaf1	23.404	34.039	0.46	0.00
Cnn2	6.971	9.758	0.47	0.00
6430531B16Rik	0.401	0.622	0.56	0.00
St14	0.229	0.341	0.56	0.00
Dydc1	0.089	0.031	-1.00	0.00
Ublcp1	17.269	13.127	-0.50	0.00
1500009L16Rik	31.587	24.144	-0.46	0.00
Gbp8	0.803	0.503	-0.52	0.00
Krt1	7.477	5.568	-0.52	0.00
Defb29	0.077	0.000	-1.34	0.00
Zfp133-ps	3.821	2.801	-0.51	0.00
2210013O21Rik	42.441	30.653	-0.52	0.00
Klhl4	3.970	3.043	-0.45	0.00

2010012O05Rik	10.543	8.200	-0.48	0.00	
Cwc15	67.709	50.738	-0.48	0.00	
Gm17745	0.171	0.084	-0.78	0.00	
2810408I11Rik	0.836	0.579	-0.59	0.00	
Hibch	12.249	9.291	-0.44	0.00	+
Clec2i	0.093	0.043	-0.87	0.00	
Cmbl	6.792	4.845	-0.46	0.00	
Ap3s1	75.397	57.392	-0.50	0.00	
Gm8817	0.046	0.000	-1.36	0.00	
Slc5a9	0.013	0.002	-1.22	0.00	
Pdzd11	30.356	22.234	-0.49	0.00	
U2af1	62.613	48.669	-0.48	0.00	
Nmd3	8.888	6.403	-0.50	0.00	
Nme6	13.750	10.616	-0.48	0.00	
Qpct	10.137	7.430	-0.49	0.00	
1700024F13Rik	1.984	1.465	-0.54	0.00	
Olfr558	0.029	0.007	-1.25	0.00	
Ndufs8	60.888	45.314	-0.54	0.00	+
Ppp2r3c	73.643	55.274	-0.49	0.00	
Fra10ac1	9.113	6.832	-0.47	-0.01	
Mitd1	4.609	3.290	-0.51	-0.01	
Mrpl36	19.240	15.151	-0.43	-0.01	
Pigx	24.234	17.899	-0.50	-0.01	
Cox4i1	473.140	354.730	-0.52	-0.01	
Spc25	4.631	3.352	-0.49	-0.01	
Rln1	2.969	2.087	-0.54	-0.01	
Gm13212	3.771	2.773	-0.54	-0.01	
Agr3	0.134	0.050	-0.94	-0.01	
2700069I18Rik	5.540	3.983	-0.52	-0.01	
2610318N02Rik	1.218	0.904	-0.56	-0.01	
6720483E21Rik	0.303	0.184	-0.66	-0.01	
Snord99	3.079	1.289	-0.87	-0.01	
Gm10635	0.065	0.033	-0.81	-0.01	
N6amt1	10.904	8.529	-0.47	-0.01	
Scnm1	21.862	16.181	-0.49	-0.01	

Fastkd2	4.817	3.730	-0.46	-0.01
Mir7084	61.291	46.414	-0.49	-0.01
Fcgbp	0.146	0.099	-0.57	-0.01
Ppp1r11	31.246	23.534	-0.47	-0.01
Bex2	608.996	458.223	-0.51	-0.01
Gm15107	0.021	0.000	-1.08	-0.01
Acnat1	0.014	0.000	-1.35	-0.01
Nmrk2	0.045	0.011	-0.98	-0.01
Zfp101	3.574	2.625	-0.50	-0.01
Glrx	37.254	27.282	-0.51	-0.01
Ssna1	45.726	35.622	-0.46	-0.01
Olfr1318	0.061	0.009	-1.61	-0.01
Myh2	0.363	0.237	-0.52	-0.01
Gm11974	6.488	4.721	-0.51	-0.01
Ccdc59	15.871	12.352	-0.47	-0.01
Sirt3	18.574	14.636	-0.46	-0.01
Eif4a2	252.960	193.656	-0.47	-0.01
Xlr3a	20.691	14.754	-0.50	-0.01
Glis1	0.394	0.273	-0.55	-0.01
Triqk	11.211	8.411	-0.48	-0.01
Nae1	23.895	18.130	-0.47	-0.01
Gm5434	3.731	2.658	-0.53	-0.01
Psma7	85.613	66.436	-0.49	-0.01
Nxt2	7.444	5.571	-0.47	-0.01
Tbce	16.376	12.488	-0.51	-0.01
Psenen	76.843	56.886	-0.49	-0.01
Srsf7	63.877	48.891	-0.48	-0.01
Gm13139	18.730	14.024	-0.47	-0.01
Setmar	1.819	1.288	-0.53	-0.01
Chchd3	38.245	28.436	-0.49	-0.01
Glipr1l1	0.111	0.058	-0.79	-0.01
Fam132a	3.675	2.780	-0.49	-0.01
Serpinb6c	2.353	1.779	-0.51	-0.01
Chchd7	3.356	2.422	-0.54	-0.01

Fis1	108.480	82.663	-0.48	-0.01	
Use1	47.114	35.486	-0.48	-0.01	
Ankrd37	12.556	8.648	-0.47	-0.01	
1700084E18Rik	1.312	0.713	-0.70	-0.01	
Eif1ax	141.712	106.215	-0.51	-0.01	
Snx22	50.609	38.923	-0.47	-0.02	
Capza2	89.673	67.873	-0.51	-0.02	
Eif3k	96.661	71.213	-0.52	-0.02	+
Rps13	509.749	385.698	-0.49	-0.02	
Cyp7b1	2.415	1.709	-0.51	-0.02	
Oas1d	0.362	0.244	-0.60	-0.02	
Nudt7	0.655	0.435	-0.58	-0.02	
Tm6sf2	0.282	0.164	-0.71	-0.02	
Tmem81	0.741	0.527	-0.52	-0.02	
Hist1h3a	1.093	0.719	-0.66	-0.02	
Glra4	0.285	0.212	-0.63	-0.02	
Lin7b	16.546	12.000	-0.54	-0.02	
H2-Oa	0.055	0.000	-1.37	-0.02	
Atoh1	1.335	0.780	-0.56	-0.02	
Hist1h4a	2.768	1.881	-0.55	-0.02	
Znf41-ps	12.630	9.620	-0.50	-0.02	
1700021F05Rik	22.923	17.194	-0.51	-0.02	
Rplp2	196.104	145.263	-0.51	-0.02	
Hcrtr2	1.538	1.151	-0.49	-0.02	
Fuom	3.076	2.254	-0.50	-0.02	
BB287469	1.619	1.209	-0.53	-0.02	
Cd1d1	9.023	6.167	-0.54	-0.02	
Mkx	0.371	0.261	-0.60	-0.02	
Mir1892	65.780	47.058	-0.54	-0.02	
Tex11	0.038	0.014	-0.97	-0.02	
Zfp949	5.982	4.589	-0.48	-0.02	
Olfm3	11.560	8.724	-0.49	-0.02	

Hsd17b10	45.701	34.667	-0.48	-0.02	
Poln	0.132	0.081	-0.71	-0.02	
Slc27a5	0.153	0.093	-0.60	-0.02	
Htr4	0.542	0.403	-0.52	-0.02	
Slc5a7	2.755	1.927	-0.53	-0.02	
Sepp1	89.230	63.495	-0.54	-0.02	
6530411M01Rik	2.058	1.515	-0.50	-0.02	
Klkb1	0.036	0.013	-1.08	-0.02	
Gm3558	1.625	1.164	-0.54	-0.02	
Gkn3	0.415	0.234	-0.79	-0.02	
Ndufab1	79.478	58.906	-0.54	-0.02	
Denr	29.277	22.653	-0.47	-0.02	+
Ndufaf6	6.677	4.966	-0.53	-0.02	
Ift20	51.984	36.035	-0.53	-0.02	
Hpgd	1.705	1.236	-0.54	-0.02	
Rnf183	0.093	0.037	-0.97	-0.02	
Gm4305	1.322	0.972	-0.51	-0.02	
Zfp277	12.030	9.020	-0.49	-0.02	
Gm14326	17.087	12.444	-0.52	-0.03	
Nhp2l1	233.199	174.216	-0.51	-0.03	
Gm10516	2.043	1.465	-0.52	-0.03	
Mybl1	0.732	0.534	-0.53	-0.03	
Rpf1	6.222	4.569	-0.50	-0.03	
Mphosph6	12.792	8.964	-0.55	-0.03	
Rnf7	90.025	65.866	-0.49	-0.03	
Gm4302	1.299	0.948	-0.54	-0.03	
Serpinb9b	0.184	0.112	-0.73	-0.03	
Pcna	54.630	39.145	-0.51	-0.03	
Kcnj14	0.808	0.571	-0.56	-0.03	
9930111J21Rik1	0.341	0.222	-0.58	-0.03	
Zcchc16	0.574	0.412	-0.52	-0.03	
Mtf2	7.873	5.922	-0.47	-0.03	
Ikbip	3.775	2.735	-0.53	-0.03	
1700123M08Rik	1.397	0.968	-0.59	-0.03	

Rpl18a	281.359	214.199	-0.47	-0.03	
Psmb2	101.431	74.744	-0.49	-0.03	
Tmem54	3.114	2.349	-0.48	-0.03	
Zfp943	6.959	5.176	-0.48	-0.03	
Phf5a	34.830	26.242	-0.50	-0.03	
4933411E08Rik	0.018	0.000	-0.97	-0.03	
Ppib	204.073	154.572	-0.51	-0.03	
Serpini1	81.049	62.566	-0.48	-0.03	
Nxt1	9.844	6.998	-0.50	-0.03	
Nupr1l	0.807	0.476	-0.71	-0.03	
Eif4e	86.126	61.917	-0.56	-0.03	+
Snca	120.950	88.227	-0.53	-0.03	
Dkk4	0.084	0.024	-0.96	-0.03	
Stmn3	697.506	525.357	-0.52	-0.03	+
Gjb1	0.641	0.433	-0.58	-0.03	
1700048M11Rik	0.357	0.171	-0.95	-0.03	
Ovol3	1.168	0.797	-0.57	-0.03	
Nppa	1.860	1.356	-0.54	-0.03	
Zufsp	3.035	2.216	-0.51	-0.03	
Apip	11.735	8.729	-0.52	-0.03	
Commd3	54.417	40.706	-0.53	-0.03	
Glrx3	43.066	31.773	-0.56	-0.03	
Ndufs3	66.846	48.762	-0.49	-0.03	+
Hebp1	9.023	6.630	-0.52	-0.03	
Cth	0.876	0.619	-0.56	-0.03	
B2m	56.314	42.495	-0.52	-0.03	
Sepw1	659.962	517.197	-0.45	-0.03	
Mob4	37.061	27.519	-0.48	-0.03	
Hoxd10	0.040	0.000	-1.32	-0.03	
Crip1	4.653	3.438	-0.57	-0.03	

Hes1	4.048	3.057	-0.52	-0.03
Exosc8	17.006	12.317	-0.50	-0.03
Pla2g16	2.106	1.478	-0.53	-0.03
Tspan8	0.422	0.251	-0.61	-0.03
Taf9b	6.659	4.849	-0.52	-0.03
Tnnt2	0.305	0.165	-0.76	-0.03
Nudt12	1.565	1.126	-0.52	-0.03
Mtx2	48.960	36.829	-0.51	-0.03
Gm14322	4.097	2.956	-0.52	-0.03
Mrpl46	25.322	18.203	-0.55	-0.04
Faim	32.620	24.441	-0.52	-0.04
5830411N06Rik	0.012	0.000	-1.16	-0.04
Ttc1	28.260	20.715	-0.51	-0.04
2700094K13Rik	85.406	63.753	-0.51	-0.04
Coa4	4.862	3.722	-0.48	-0.04
Arid3c	0.233	0.153	-0.63	-0.04
Zwint	232.814	174.817	-0.50	-0.04
Nms	0.198	0.093	-0.82	-0.04
Sdf2	53.956	40.166	-0.53	-0.04
Phlda2	0.220	0.098	-0.91	-0.04
Ube2f	15.217	11.131	-0.54	-0.04
Nlrp4f	0.050	0.026	-0.86	-0.04
Tyw5	8.304	5.838	-0.54	-0.04
D5Ertd615e	0.023	0.005	-1.04	-0.04
Rec114	1.124	0.724	-0.68	-0.04
Nuf2	4.374	2.998	-0.54	-0.04
2610316D01Rik	7.793	5.381	-0.57	-0.04
Vsnl1	195.228	146.736	-0.51	-0.04
Brcc3	6.742	4.963	-0.52	-0.04
Nanp	19.961	14.957	-0.53	-0.04
Mir5113	7.902	4.905	-0.61	-0.04
Ube2w	13.421	9.682	-0.49	-0.04
Eif3m	36.428	27.451	-0.51	-0.04
9230112J17Rik	0.066	0.029	-1.02	-0.04

Pir	1.332	0.992	-0.50	-0.04	
Acot13	31.964	23.380	-0.50	-0.04	+
Ccdc28b	53.303	38.997	-0.53	-0.04	
Gm4307	1.322	0.972	-0.56	-0.04	
Haus7	20.896	15.792	-0.49	-0.04	
Zfp942	7.500	5.376	-0.53	-0.04	
Lamtor2	67.652	51.209	-0.46	-0.04	+
Hoxa2	3.197	2.260	-0.53	-0.04	
Bola3	25.488	18.254	-0.55	-0.04	
Rbpjl	0.045	0.017	-0.91	-0.04	
Tnfaip6	0.937	0.696	-0.52	-0.04	
Fate1	0.033	0.000	-1.03	-0.04	
Emg1	33.545	25.159	-0.48	-0.04	
Mdh1	177.661	130.301	-0.54	-0.04	
Stfa2l1	0.808	0.523	-0.68	-0.04	
Lrrc51	3.662	2.597	-0.56	-0.05	
4930563D23Rik	0.020	0.000	-1.28	-0.05	
AY074887	3.100	2.128	-0.56	-0.05	
Shcbp1	2.231	1.538	-0.54	-0.05	
Il21r	0.158	0.087	-0.74	-0.05	
Gm15133	0.113	0.037	-0.92	-0.05	
Rap1a	21.384	15.578	-0.52	-0.05	
Acer3	3.114	2.211	-0.53	-0.05	
Ckmt2	0.056	0.014	-0.92	-0.05	
Gm8773	5.626	3.904	-0.57	-0.05	
Ssu72	56.832	41.655	-0.54	-0.05	
Nup43	8.341	6.005	-0.54	-0.05	
Gng10	61.464	43.107	-0.57	-0.05	
Hsdl2	16.276	11.713	-0.50	-0.05	
Cbwd1	4.961	3.477	-0.52	-0.05	
Gm4303	1.322	0.972	-0.52	-0.05	
Vimp	28.542	21.494	-0.51	-0.05	
Sumo1	103.251	77.436	-0.52	-0.05	+
2810001G20Rik	18.142	12.581	-0.59	-0.05	

1700063D05Rik	0.162	0.073	-0.92	-0.05	
Hapln1	1.061	0.726	-0.54	-0.05	
Fam187b	0.155	0.104	-0.65	-0.05	
Mrpl14	31.109	23.592	-0.50	-0.05	
Scin	0.033	0.007	-1.21	-0.05	
Ube2t	2.048	1.402	-0.62	-0.05	
Rps8	1679.5<br>80	#######	-0.53	-0.05	
Timm21	13.655	9.334	-0.56	-0.05	
Nqo1	3.478	2.645	-0.53	-0.05	
Fhit	3.399	2.436	-0.52	-0.05	
B9d2	12.381	8.738	-0.58	-0.05	
Nsa2	73.033	55.787	-0.49	-0.05	
Gm21951	0.009	0.000	-1.21	-0.05	
Speer8-ps1	0.051	0.000	-1.59	-0.05	
Cdc42	226.69<br>6	170.021	-0.52	-0.05	
Gcsh	60.271	44.388	-0.52	-0.05	+
Sult2b1	1.099	0.726	-0.61	-0.05	
Gm38402	0.034	0.000	-1.00	-0.05	
Trhr	1.548	1.126	-0.51	-0.05	
Ska2	16.929	11.996	-0.56	-0.05	
Ostc	61.858	45.083	-0.55	-0.05	
Sh2d7	1.601	1.193	-0.55	-0.05	
5033406O09Rik	0.187	0.107	-0.77	-0.05	
Sh2d1a	0.191	0.076	-0.90	-0.05	
Timm9	28.482	20.010	-0.54	-0.05	
Npm1	158.07<br>3	120.305	-0.49	-0.05	
U2af1l4	22.984	17.107	-0.50	-0.05	+
Rnaseh2b	8.579	6.272	-0.50	-0.06	
C230004F18Rik	4.642	3.418	-0.52	-0.06	
Mrpl18	48.999	35.416	-0.57	-0.06	
Mt3	120.36<br>8	89.376	-0.53	-0.06	
AA543186	0.176	0.097	-0.73	-0.06	

Tmem251	10.287	7.404	-0.54	-0.06	
Ndufb7	145.94<br>0	107.514	-0.53	-0.06	
Zcchc9	6.502	4.780	-0.55	-0.06	
Tgm3	0.076	0.045	-0.74	-0.06	
Mylk4	0.092	0.055	-0.69	-0.06	
Gpx8	18.517	12.913	-0.56	-0.06	
Mien1	77.902	56.811	-0.53	-0.06	
Pkib	0.594	0.405	-0.60	-0.06	+
Serf2	54.107	40.415	-0.51	-0.06	+
Tcf24	1.003	0.661	-0.58	-0.06	
Tcte3	1.790	1.108	-0.63	-0.06	
1700028B04Rik	24.179	17.620	-0.55	-0.06	
l7Rn6	11.696	8.949	-0.50	-0.06	
Ptn	279.39<br>8	211.340	-0.52	-0.06	
Fancd2os	0.119	0.046	-0.99	-0.06	
Ccdc23	57.628	40.804	-0.59	-0.06	
D3Ertd751e	1.761	1.283	-0.54	-0.06	
Dytn	0.030	0.013	-0.89	-0.06	
Smc2os	1.094	0.676	-0.65	-0.06	
Hes5	20.628	14.706	-0.56	-0.06	
Mterf1a	4.014	2.901	-0.55	-0.06	
Slc24a1	0.064	0.036	-0.77	-0.06	
AK010878	19.010	12.959	-0.62	-0.06	
Gpr174	0.082	0.048	-0.71	-0.06	
Synpr	21.835	15.324	-0.58	-0.06	
Smim15	20.511	14.719	-0.56	-0.06	
Rpl19	889.15<br>8	621.498	-0.58	-0.06	
Tmsb10	1807.2<br>30	#######	-0.51	-0.06	+
Mir485	0.573	0.000	-1.32	-0.06	
Cox7a2l	264.48<br>3	195.286	-0.56	-0.06	
2310009B15Rik	24.225	17.684	-0.52	-0.06	

Pdzph1	0.353	0.247	-0.56	-0.06	
Gm16062	21.986	15.359	-0.56	-0.06	
Bcap29	11.253	7.802	-0.53	-0.06	+
Muc20	0.018	0.000	-1.13	-0.06	
Ssx9	0.087	0.000	-1.31	-0.06	
Mrpl51	41.230	28.178	-0.59	-0.06	+
Hacd1	15.501	11.153	-0.55	-0.06	
Sdf2l1	4.702	3.333	-0.52	-0.06	
Lyrm4	16.367	12.404	-0.51	-0.06	
Ndufb9	160.555	115.150	-0.55	-0.06	
S100a11	51.957	36.161	-0.60	-0.06	
Sra1	36.179	26.677	-0.55	-0.06	+
Chmp5	65.121	46.609	-0.56	-0.06	
4930520O04Rik	0.808	0.551	-0.60	-0.06	
Hmgb2	63.731	45.264	-0.51	-0.06	
Hist1h4h	5.094	3.272	-0.60	-0.06	
Prdx3	37.767	28.280	-0.51	-0.06	+
5430416N02Rik	18.340	13.478	-0.55	-0.06	
0610037L13Rik	18.386	12.628	-0.56	-0.07	
Nck1	16.406	11.857	-0.59	-0.07	
Txndc9	14.213	10.108	-0.56	-0.07	
Chrac1	17.053	11.841	-0.55	-0.07	
Tmprss11d	0.018	0.000	-1.45	-0.07	
Zcrb1	69.321	49.797	-0.57	-0.07	
Btf3	162.037	115.090	-0.54	-0.07	
Cfdp1	105.578	77.878	-0.55	-0.07	+
B230214G05Rik	0.085	0.045	-0.86	-0.07	
Ccdc173	1.256	0.828	-0.59	-0.07	
Mrpl40	29.591	22.075	-0.51	-0.07	+
Rpf2	15.628	10.822	-0.58	-0.07	
Npy5r	0.795	0.566	-0.58	-0.07	
Vmn2r78	0.086	0.040	-0.80	-0.07	
Timm10b	31.302	23.111	-0.52	-0.07	

Nkiras1	20.584	15.117	-0.54	-0.07	
D930007P13Rik	0.043	0.008	-1.13	-0.07	
Gm14440	12.942	9.122	-0.53	-0.07	
Olfr1393	0.061	0.011	-1.23	-0.07	
Vmn1r198	0.943	0.662	-0.59	-0.07	
Cndp1	0.046	0.018	-0.99	-0.07	
Ptges3	103.871	77.298	-0.52	-0.07	
Vmn1r158	0.209	0.112	-0.78	-0.07	
Ormdl1	23.358	16.980	-0.55	-0.07	
Aimp2	26.658	19.915	-0.55	-0.07	
Esd	93.426	65.765	-0.59	-0.07	
Fam96a	20.778	15.377	-0.53	-0.07	
Atp5f1	160.080	117.842	-0.55	-0.07	
Mterf1b	3.308	2.390	-0.57	-0.07	
Gm10377	0.302	0.183	-0.72	-0.07	
Tmem216	9.470	6.643	-0.56	-0.07	
Lyrm5	7.934	5.713	-0.56	-0.07	
Cpvl	0.043	0.012	-1.15	-0.07	
Cd9	50.965	37.596	-0.52	-0.07	
Dad1	20.428	14.473	-0.60	-0.07	
Dph5	5.339	3.837	-0.56	-0.07	
Pcbd2	6.537	4.741	-0.55	-0.07	
Rpl14-ps1	552.812	399.397	-0.58	-0.07	
Zfp788	9.690	6.984	-0.53	-0.07	
Tmem100	8.185	5.923	-0.57	-0.07	
9330175M20Rik	0.078	0.044	-0.74	-0.07	
Ndufb11	109.252	74.242	-0.60	-0.07	+
4933428C19Rik	0.051	0.000	-1.23	-0.07	
Snora81	21.541	15.157	-0.62	-0.07	
Mnat1	6.261	4.546	-0.58	-0.07	
Mir208a	0.441	0.000	-1.34	-0.07	
Commd9	40.005	29.889	-0.49	-0.07	

Ndufaf4	11.725	8.619	-0.56	-0.07	
Dscc1	0.918	0.630	-0.65	-0.07	
Sctr	0.215	0.118	-0.76	-0.07	
Rsl24d1	31.897	23.583	-0.53	-0.07	
2310001H17Rik	0.258	0.134	-0.96	-0.07	
Gm21319	1.614	1.145	-0.55	-0.07	
Htr3b	0.048	0.017	-1.09	-0.08	
5930438M14Rik	0.150	0.076	-0.80	-0.08	
Rnase6	0.722	0.453	-0.67	-0.08	
Cops2	33.443	25.010	-0.56	-0.08	
Fopnl	43.545	30.921	-0.54	-0.08	
Dyrk4	0.113	0.060	-0.83	-0.08	
4931402G19Rik	0.139	0.082	-0.84	-0.08	
1110008P14Rik	63.298	45.024	-0.53	-0.08	
Mthfd2l	14.674	10.755	-0.55	-0.08	+
1810024B03Rik	0.202	0.098	-0.82	-0.08	
1810009A15Rik	22.060	15.655	-0.55	-0.08	
Slc22a14	0.045	0.005	-1.25	-0.08	
Ptpmt1	16.686	12.165	-0.54	-0.08	
Brix1	10.298	7.054	-0.60	-0.08	
1700109H08Rik	1.411	0.995	-0.65	-0.08	
1700064M15Rik	0.211	0.064	-1.11	-0.08	
BC048502	0.390	0.185	-0.74	-0.08	
Ssb	86.559	63.021	-0.60	-0.08	
Oard1	13.344	9.106	-0.58	-0.08	
4933400C23Rik	0.061	0.024	-1.01	-0.08	
Creb3l3	0.208	0.113	-0.72	-0.08	
Nudt2	33.635	24.289	-0.52	-0.08	
Arl6	20.061	13.961	-0.56	-0.08	
Zfp493	1.881	1.343	-0.57	-0.08	
Gm1564	0.120	0.074	-0.74	-0.08	
Gm5347	0.031	0.000	-1.28	-0.08	
Nexn	2.054	1.098	-0.57	-0.08	
Mir3063	0.909	0.336	-1.06	-0.08	
Hist1h4n	3.727	2.311	-0.61	-0.08	
Ccdc122	0.413	0.276	-0.67	-0.08	

Gm4371	2.204	1.576	-0.57	-0.08
Zfp773	2.994	2.165	-0.58	-0.08
Mlf1	9.776	6.858	-0.57	-0.08
Dnajc12	9.778	7.065	-0.58	-0.08
Emc4	83.382	61.419	-0.55	-0.08
Pigf	16.627	11.777	-0.51	-0.08
Gm14379	0.370	0.253	-0.65	-0.08
Tm2d1	25.392	17.929	-0.58	-0.08
Hist1h4m	3.445	2.088	-0.64	-0.08
Coa6	15.856	10.652	-0.60	-0.08
Crhbp	3.162	2.264	-0.58	-0.08
Mir684-2	192.018	135.043	-0.59	-0.08
Exosc7	18.414	13.318	-0.56	-0.09
1700001L19Rik	7.573	5.111	-0.65	-0.09
Ccdc169	0.049	0.019	-0.93	-0.09
Hist1h3d	1.503	1.003	-0.66	-0.09
Gm10406	0.305	0.200	-0.64	-0.09
Ttn	0.023	0.014	-0.67	-0.09
Ggct	5.748	3.900	-0.60	-0.09
Pbk	8.563	5.840	-0.57	-0.09
Fundc2	18.262	13.085	-0.59	-0.09
Llph	51.633	35.469	-0.63	-0.09
Atg10	3.417	2.308	-0.60	-0.09
Snord22	18.912	12.197	-0.58	-0.09
Cryba4	0.757	0.494	-0.69	-0.09
Cetn1	0.815	0.530	-0.66	-0.09
Cenpq	5.273	3.533	-0.63	-0.09
Tbx6	0.305	0.186	-0.70	-0.09
Tyrobp	11.624	8.146	-0.58	-0.09
Rps3	298.026	213.548	-0.59	-0.09
Tspan6	87.810	64.716	-0.56	-0.09
Lage3	22.574	15.688	-0.57	-0.09
Spin2c	10.224	7.629	-0.53	-0.09
Ap4s1	25.251	17.830	-0.58	-0.09

AI662270	0.241	0.132	-0.78	-0.09	
Fam227b	0.315	0.189	-0.74	-0.09	
Mocs2	38.758	26.820	-0.60	-0.09	
Zfp958	6.213	4.229	-0.61	-0.09	
Myl4	1.290	0.864	-0.64	-0.09	+
Xlr4a	2.940	1.758	-0.59	-0.09	
4922502N22Rik	0.050	0.009	-1.30	-0.09	
Zfp708	5.186	3.579	-0.57	-0.09	
Trnt1	15.460	11.392	-0.54	-0.09	
Dtymk	24.621	17.038	-0.59	-0.09	
Tsen15	16.227	11.362	-0.58	-0.09	
Lamp5	22.691	14.962	-0.55	-0.09	
Fbxl22	1.716	1.180	-0.60	-0.09	
1700007L15Rik	0.378	0.207	-0.82	-0.09	
Xlr4b	2.173	1.219	-0.66	-0.09	
Hist1h2bh	9.701	6.698	-0.61	-0.09	
4930465K10Rik	0.170	0.082	-0.82	-0.10	
Gm10440	0.099	0.052	-0.83	-0.10	
Rabggtb	32.917	23.143	-0.61	-0.10	
Mrpl35	8.344	5.722	-0.59	-0.10	
Gm14391	16.085	11.337	-0.56	-0.10	
Ndufb8	363.401	265.592	-0.58	-0.10	
Chek1	1.031	0.717	-0.58	-0.10	
Il2	0.044	0.000	-1.16	-0.10	
Rhbg	0.194	0.102	-0.87	-0.10	
Gm9199	4.889	3.588	-0.54	-0.10	
Gm5086	0.210	0.103	-1.02	-0.10	
Polr2d	56.768	41.002	-0.58	-0.10	
Tipin	16.523	11.413	-0.57	-0.10	
Med30	32.855	23.648	-0.56	-0.10	
Mrpl13	38.881	26.271	-0.61	-0.10	
Serpinb2	0.094	0.040	-0.95	-0.10	
Snhg14	13.955	9.764	-0.57	-0.10	
Commd2	8.328	5.768	-0.60	-0.10	
A930005H10Rik	2.637	1.850	-0.61	-0.10	

Zfp418	1.101	0.745	-0.58	-0.10	
Sec11a	41.492	27.856	-0.63	-0.10	
Spata4	0.252	0.156	-0.74	-0.10	
Thyn1	41.931	28.854	-0.60	-0.10	
Tmem223	70.478	50.012	-0.57	-0.10	
Gabrg1	13.318	9.134	-0.64	-0.10	
5430421F17Rik	0.032	0.004	-0.92	-0.10	
Utp11l	17.794	12.381	-0.60	-0.10	
Tecrl	0.081	0.039	-0.86	-0.10	
Cst3	396.618	287.182	-0.56	-0.10	
Cbx3	417.081	302.707	-0.56	-0.11	+
Taf9	166.532	115.489	-0.60	-0.11	+
Gpr84	0.155	0.073	-0.94	-0.11	
Snord88a	0.435	0.143	-1.17	-0.11	
1810043H04Rik	14.598	10.419	-0.57	-0.11	
Stra13	20.698	14.271	-0.59	-0.11	
Calcb	2.455	1.447	-0.68	-0.11	
Gm13152	4.691	3.280	-0.59	-0.11	
Gal3st2	0.024	0.000	-1.38	-0.11	
Gm6787	18.966	12.889	-0.59	-0.11	
Igip	5.472	3.735	-0.58	-0.11	
Mypn	0.169	0.110	-0.70	-0.11	
Mterf3	9.194	6.405	-0.62	-0.11	
Rps5	696.478	492.526	-0.54	-0.11	
Trappc1	73.259	52.041	-0.60	-0.11	
Gm4532	2.104	1.316	-0.64	-0.11	
Mb	0.409	0.247	-0.71	-0.11	
C030023E24Rik	1.199	0.797	-0.63	-0.11	
1810062G17Rik	0.115	0.034	-1.16	-0.11	
Apol7a	0.024	0.000	-1.23	-0.11	
Rpl10a	366.146	255.678	-0.59	-0.11	

Tlx1	0.291	0.168	-0.75	-0.11	
Atp6v0e	27.869	18.866	-0.63	-0.11	
Paip2	137.441	97.897	-0.61	-0.11	
Rpp38	6.503	4.369	-0.62	-0.11	
Fbxl17	5.038	3.476	-0.58	-0.11	
Fam178b	0.020	0.000	-1.21	-0.11	
Gm14430	18.605	12.953	-0.63	-0.11	
Gabra6	0.061	0.017	-1.13	-0.11	
Dynlt1a	137.976	99.285	-0.59	-0.11	
Msrb2	3.741	2.626	-0.58	-0.11	
S100a6	10.989	7.617	-0.62	-0.11	+
Hspb7	0.083	0.041	-0.89	-0.11	
Cers3	0.109	0.054	-0.79	-0.11	
Uqcc3	25.148	17.227	-0.62	-0.11	
Dnase1l2	1.856	1.310	-0.60	-0.11	
3110009E18Rik	1.895	1.288	-0.58	-0.11	
Erh	399.831	271.698	-0.61	-0.11	
Mettl6	9.895	6.844	-0.59	-0.11	
Gm6460	0.083	0.025	-1.29	-0.11	
Pantr1	50.803	35.363	-0.62	-0.11	
Gm14139	0.094	0.030	-1.10	-0.11	
Gm14434	18.038	12.259	-0.61	-0.11	
2810403D21Rik	0.983	0.616	-0.66	-0.11	
Mterf2	8.751	6.108	-0.60	-0.11	
5530601H04Rik	2.289	1.644	-0.54	-0.12	
Gm10408	4.342	3.092	-0.58	-0.12	
Rd3l	0.151	0.041	-1.04	-0.12	
Dpcd	22.985	15.559	-0.63	-0.12	
Mgst3	165.234	113.618	-0.61	-0.12	+
Timp4	16.430	11.013	-0.58	-0.12	
Rab9	34.076	22.708	-0.63	-0.12	
H2-Eb2	0.100	0.061	-0.75	-0.12	

Dynlt3	29.180	20.355	-0.61	-0.12	
Coq10b	12.180	8.600	-0.58	-0.12	
1110034G24Rik	4.899	3.155	-0.67	-0.12	
Zswim7	5.261	3.566	-0.64	-0.12	+
Akp3	0.031	0.006	-1.10	-0.12	
Mettl21c	0.967	0.608	-0.69	-0.12	
Them4	31.708	23.401	-0.57	-0.12	
2010204K13Rik	5.336	3.598	-0.64	-0.12	
Hist1h4b	3.902	2.544	-0.66	-0.12	
Gm12657	604.557	427.888	-0.62	-0.12	
Sumo2	1110.850	755.152	-0.62	-0.12	
Ngdn	21.034	14.981	-0.56	-0.12	
Itgb3bp	2.212	1.405	-0.65	-0.12	
Ccnc	8.837	5.940	-0.60	-0.12	
Wdr76	1.290	0.836	-0.63	-0.12	
Polr2g	43.944	29.742	-0.59	-0.12	
2210015D19Rik	7.515	5.174	-0.62	-0.12	
Gm3701	0.007	0.000	-1.46	-0.12	
Mycs	0.027	0.000	-1.39	-0.12	
Dctpp1	48.780	34.620	-0.60	-0.12	
Cenpa	5.299	3.666	-0.54	-0.12	
Gm4461	0.077	0.028	-1.09	-0.12	
Ndufa7	86.202	59.729	-0.64	-0.12	+
Gm14351	0.006	0.000	-1.20	-0.12	
Fbxo43	0.013	0.000	-1.32	-0.12	
15-Sep	160.576	106.846	-0.64	-0.12	
Krtcap2	75.015	52.450	-0.57	-0.12	+
Lypla1	13.283	9.216	-0.63	-0.12	
Timm8a1	28.841	20.663	-0.57	-0.12	
Aimp1	68.122	49.971	-0.57	-0.12	+
H3f3a	1460.300	#######	-0.63	-0.12	
Cyb5a	89.690	59.929	-0.64	-0.12	

Tac1	34.559	24.329	-0.60	-0.12	
Gm3002	0.318	0.218	-0.63	-0.13	
Lyrm2	41.425	28.073	-0.64	-0.13	
Bex6	0.638	0.388	-0.70	-0.13	
Klrg1	0.561	0.356	-0.70	-0.13	
Cd1d2	5.881	3.963	-0.65	-0.13	
Olfr329-ps	0.040	0.000	-1.10	-0.13	
Zfp455	1.227	0.840	-0.57	-0.13	
A230072C01Rik	3.440	2.381	-0.59	-0.13	
5330411J11Rik	0.083	0.025	-1.10	-0.13	
Gm4724	18.038	12.259	-0.61	-0.13	
Il12b	0.025	0.000	-1.39	-0.13	
Pth2	0.156	0.036	-1.10	-0.13	
Adam32	0.092	0.045	-0.88	-0.13	
Rps19	1526.6<br>40	#######	-0.65	-0.13	
Sptssa	56.548	38.994	-0.60	-0.13	
Stfa3	0.533	0.200	-1.06	-0.13	
Txk	0.086	0.042	-0.85	-0.13	
Vmn1r177	0.264	0.144	-0.87	-0.13	
Ppwd1	3.158	2.201	-0.63	-0.13	
Fam159b	1.125	0.709	-0.69	-0.13	
Sod1	225.88<br>7	156.638	-0.60	-0.13	
Eqtn	0.191	0.096	-0.85	-0.13	
Psmb7	208.08<br>3	145.781	-0.59	-0.13	
Psmg4	21.450	14.820	-0.60	-0.13	
2810417H13Rik	8.026	5.254	-0.58	-0.13	
Klrc3	0.086	0.000	-1.28	-0.13	
Eif3e	125.24<br>3	87.974	-0.62	-0.13	
Nme2	215.92<br>6	148.989	-0.63	-0.13	
9530062K07Rik	0.359	0.197	-0.76	-0.13	
Impact	27.203	18.867	-0.63	-0.13	+

Cox5b	192.115	135.932	-0.57	-0.13	+
Hsd3b3	1.671	1.073	-0.66	-0.13	
4632427E13Rik	0.562	0.293	-0.79	-0.13	
Crh	2.379	1.586	-0.61	-0.13	
Hmgn3	61.614	42.140	-0.64	-0.13	
Fam92a	44.415	30.686	-0.63	-0.13	
Ndufa12	97.362	66.058	-0.61	-0.13	+
Lipo1	12.222	8.358	-0.64	-0.13	
Tas2r135	0.027	0.000	-1.18	-0.13	
Platr10	0.066	0.010	-1.06	-0.13	
Psmd14	66.599	47.160	-0.57	-0.13	+
Selk	97.139	67.964	-0.62	-0.13	
Cwh43	0.358	0.213	-0.73	-0.13	
Spata9	1.538	0.960	-0.70	-0.13	
Atp5c1	219.481	155.400	-0.62	-0.13	
Gm12060	26.076	17.678	-0.60	-0.14	
Hbb-y	0.279	0.100	-1.01	-0.14	
Zfp472	3.450	2.401	-0.62	-0.14	
Dnajc24	7.477	5.011	-0.62	-0.14	
Pfn3	0.451	0.227	-0.87	-0.14	
Snord49b	28.866	19.810	-0.66	-0.14	
Cldn10	6.487	4.337	-0.63	-0.14	
Vmn2r84	0.189	0.109	-0.75	-0.14	
Rps19-ps3	476.553	322.594	-0.64	-0.14	
2300002M23Rik	0.033	0.000	-1.29	-0.14	
Cutc	2.553	1.640	-0.70	-0.14	
Myh13	0.221	0.149	-0.66	-0.14	
4930429D17Rik	0.035	0.006	-1.06	-0.14	
Rdm1	3.746	2.555	-0.63	-0.14	
Mrpl41	21.511	14.328	-0.64	-0.14	+
Prorsd1	2.070	1.343	-0.69	-0.14	
Psma2	230.382	159.400	-0.61	-0.14	+

Psmc6	86.383	60.117	-0.62	-0.14	
Proca1	1.527	0.948	-0.67	-0.14	
Clec2f	0.072	0.000	-1.61	-0.14	
Srsf3	149.159	105.817	-0.57	-0.14	
A330050F15Rik	0.446	0.270	-0.70	-0.14	
A930001A20Rik	0.129	0.065	-0.91	-0.14	
Psmb11	0.017	0.005	-1.14	-0.14	
6720468P15Rik	0.726	0.468	-0.72	-0.14	
Ssxb9	0.054	0.000	-1.41	-0.14	
Gpx4	113.946	82.479	-0.59	-0.14	
Slc22a13b-ps	0.370	0.220	-0.75	-0.14	
Vdac3	209.146	149.986	-0.60	-0.14	+
Ccdc153	1.997	1.206	-0.66	-0.14	
Ier3ip1	70.016	46.140	-0.63	-0.14	
Il18	10.826	7.158	-0.69	-0.14	
Gm10637	0.100	0.025	-1.33	-0.14	
Naa20	39.002	26.076	-0.64	-0.14	
5730457N03Rik	0.676	0.439	-0.68	-0.14	
Tmem208	36.574	24.807	-0.64	-0.14	+
Gm8267	0.046	0.008	-1.04	-0.14	
Mrpl22	20.643	14.210	-0.61	-0.14	
Gabarapl2	229.690	165.499	-0.59	-0.14	
Rpl23a	1984.320	#######	-0.59	-0.14	
Cgrrf1	6.708	4.668	-0.60	-0.14	
Cacybp	142.811	102.824	-0.61	-0.14	+
Mir31	0.641	0.097	-1.41	-0.14	
Gin1	2.412	1.607	-0.66	-0.15	
Clec2g	0.120	0.059	-0.91	-0.15	
Calm2	1508.190	#######	-0.62	-0.15	

Rad51b	0.461	0.298	-0.67	-0.15
Supt4a	37.614	26.269	-0.64	-0.15
Rpl12	462.122	319.301	-0.61	-0.15
Nedd8	177.538	120.240	-0.63	-0.15
Rpl36	379.685	254.431	-0.64	-0.15
6430550D23Rik	0.262	0.167	-0.70	-0.15
B230217O12Rik	1.693	1.135	-0.62	-0.15
Gm20300	9.323	6.491	-0.64	-0.15
Mir6902	0.898	0.161	-1.03	-0.15
Gipc2	3.072	2.130	-0.60	-0.15
Pldi	0.061	0.012	-1.37	-0.15
Syf2	28.672	18.801	-0.62	-0.15
Aldh3b3	0.256	0.144	-0.73	-0.15
Tusc5	0.173	0.102	-0.76	-0.15
Tgm5	0.076	0.041	-0.92	-0.15
Mthfsl	39.764	27.491	-0.60	-0.15
Crabp1	33.720	20.860	-0.64	-0.15
Tph2	4.798	3.189	-0.64	-0.15
1300002E11Rik	2.285	1.532	-0.64	-0.15
Ces1b	0.021	0.000	-1.24	-0.15
Etohi1	8.000	5.095	-0.67	-0.15
A330023F24Rik	1.500	1.015	-0.65	-0.15
Ccdc53	8.227	5.565	-0.65	-0.15
AI413582	37.893	25.826	-0.62	-0.15
Ccdc90b	18.703	12.524	-0.64	-0.15
Mrpl30	127.499	88.663	-0.63	-0.15
Nup35	10.663	7.141	-0.64	-0.15
Mrpl54	57.804	39.183	-0.63	-0.15
Lbhd1	13.401	9.220	-0.62	-0.15
Dmgdh	0.147	0.080	-0.80	-0.16
Iapp	0.120	0.030	-1.28	-0.16
Osgepl1	5.161	3.427	-0.67	-0.16

Ces2b	0.116	0.065	-0.84	-0.16	
Xrcc4	8.253	5.420	-0.69	-0.16	
Cox5a	304.161	209.263	-0.63	-0.16	
Nme1	253.187	172.600	-0.64	-0.16	+
Snrpd3	137.956	91.171	-0.61	-0.16	
1110012L19Rik	30.267	20.433	-0.64	-0.16	
Rpl14	223.267	157.767	-0.61	-0.16	
Dupd1	0.128	0.065	-0.98	-0.16	
Ifitm6	0.396	0.210	-0.86	-0.16	
Gpr1	0.221	0.109	-0.87	-0.16	
Atp5g3	379.859	250.516	-0.65	-0.16	
Plekhg6	0.180	0.106	-0.77	-0.16	
Rpl22	168.381	114.678	-0.64	-0.16	
Bloc1s2	61.684	41.279	-0.64	-0.16	
Jsrp1	0.908	0.545	-0.78	-0.16	
Ccdc160	4.226	2.706	-0.67	-0.16	
Mettl5	18.557	12.555	-0.65	-0.16	
Bpifa1	0.047	0.000	-1.51	-0.16	
Kbtbd3	3.391	2.224	-0.65	-0.16	
1700102H20Rik	0.863	0.495	-0.75	-0.16	
Grxcr1	0.088	0.029	-1.03	-0.16	
Snora16a	1.691	0.834	-0.88	-0.16	
Gm20257	1.580	0.992	-0.71	-0.16	
Rhox4d	0.069	0.000	-1.32	-0.16	
Hist1h4f	1.752	1.008	-0.78	-0.16	
Cnga4	0.086	0.042	-0.90	-0.16	
2610528J11Rik	0.069	0.011	-1.00	-0.16	
Rhd	0.252	0.123	-0.98	-0.16	
Atp5g1	147.472	101.751	-0.64	-0.16	

Il17b	0.408	0.194	-0.91	-0.16	
Bud31	71.946	49.771	-0.64	-0.16	+
F630206G17Rik	0.024	0.000	-1.28	-0.16	
Cript	58.381	39.574	-0.65	-0.17	
Cstb	34.254	24.021	-0.60	-0.17	
Mir7049	2.787	1.031	-1.16	-0.17	
Fa2h	0.499	0.306	-0.75	-0.17	
Mrpl23	41.214	27.221	-0.68	-0.17	
Hprt	54.179	37.128	-0.64	-0.17	
Tspan12	10.865	7.506	-0.63	-0.17	
Obox7	0.066	0.016	-1.24	-0.17	
Cd59a	2.933	1.967	-0.67	-0.17	
Wfdc12	0.072	0.000	-1.38	-0.17	
Mt1	109.057	70.086	-0.66	-0.17	
4933416C03Rik	0.035	0.009	-1.15	-0.17	
Gm13582	0.061	0.023	-1.07	-0.17	
Spink10	1.644	1.063	-0.64	-0.17	
2010320M18Rik	19.984	13.014	-0.69	-0.17	
Mrps22	22.357	14.627	-0.67	-0.17	+
Cox14	55.568	38.346	-0.61	-0.17	+
Gm14308	18.038	12.259	-0.63	-0.17	
2700029M09Rik	31.509	21.579	-0.65	-0.17	
1110059E24Rik	33.444	22.383	-0.66	-0.17	
Smco3	1.100	0.739	-0.72	-0.17	
Lamtor5	59.490	37.916	-0.70	-0.17	
Cisd3	16.023	10.439	-0.63	-0.17	
Gm5662	2.433	1.597	-0.71	-0.17	
Nme3	33.222	23.400	-0.61	-0.17	
Fkbp7	3.608	2.205	-0.66	-0.17	
AA465934	5.078	3.155	-0.76	-0.18	
Pop5	19.292	12.786	-0.67	-0.18	
Rpl7	935.921	605.319	-0.64	-0.18	
Rpl9	715.869	455.670	-0.70	-0.18	

Gm3258	32.724	22.139	-0.67	-0.18	
Gm14325	18.336	12.435	-0.67	-0.18	
Ptpn20	0.222	0.117	-0.81	-0.18	
Trim43a	0.199	0.101	-0.89	-0.18	
Tbx20	0.265	0.165	-0.73	-0.18	
Pts	28.979	19.981	-0.67	-0.18	
0610010B08Rik	18.033	12.253	-0.65	-0.18	
Mrpl21	19.570	13.298	-0.68	-0.18	
Cetn2	42.278	27.794	-0.65	-0.18	
Cyp4a32	0.134	0.048	-1.10	-0.18	
Uba52	2220.5<br>90	#######	-0.68	-0.18	
Tepp	0.490	0.291	-0.84	-0.18	
Slco4c1	0.432	0.258	-0.76	-0.18	
Tigit	0.049	0.000	-1.12	-0.18	
D330023K18Rik	8.870	6.124	-0.64	-0.18	
Lamtor4	50.190	32.165	-0.71	-0.18	
Unc5cl	0.068	0.022	-1.02	-0.18	
Psma3	74.100	48.353	-0.69	-0.18	+
Swi5	242.50<br>4	162.989	-0.66	-0.18	
4930474N09Rik	0.196	0.070	-0.99	-0.18	
Rps15	821.09<br>1	557.890	-0.64	-0.18	
BC048546	0.362	0.239	-0.71	-0.18	
Mthfs	34.893	22.602	-0.69	-0.18	
Abhd3	5.143	3.334	-0.70	-0.18	
Fxyd2	5.154	3.215	-0.71	-0.18	
5730405O15Rik	0.560	0.314	-0.85	-0.18	
Ccpg1os	11.043	7.075	-0.68	-0.18	
Zfp934	3.030	2.026	-0.67	-0.18	
Mss51	0.254	0.124	-0.91	-0.18	
Nup37	4.684	3.021	-0.67	-0.18	
Tmem161b	7.852	5.427	-0.63	-0.18	
Taf13	30.349	20.316	-0.65	-0.19	
Higd1a	40.107	26.642	-0.64	-0.19	

Gm6880	0.142	0.000	-1.37	-0.19	
B230118H07Rik	10.608	6.809	-0.67	-0.19	
1700108F19Rik	0.078	0.000	-1.56	-0.19	
Rpp21	27.655	18.318	-0.66	-0.19	
Zfp930	4.240	2.833	-0.69	-0.19	
Olfr55	0.055	0.011	-1.41	-0.19	
Snhg12	37.331	24.781	-0.69	-0.19	
Pnrc2	41.056	27.503	-0.66	-0.19	
Clec4a2	0.300	0.166	-0.79	-0.19	
Clec1b	0.255	0.132	-0.89	-0.19	
Srp19	72.338	47.120	-0.66	-0.19	
Txndc17	52.431	34.616	-0.67	-0.19	+
5830454E08Rik	1.632	0.944	-0.78	-0.19	
Vmn2r11	0.028	0.000	-1.45	-0.19	
Fkbp6	0.066	0.018	-1.33	-0.19	
Gtf2h5	102.584	68.077	-0.71	-0.19	
Dynll1	271.806	177.085	-0.68	-0.19	+
Nmb	4.342	2.728	-0.70	-0.19	
Pus3	6.792	4.557	-0.72	-0.19	
Ptchd3	0.032	0.006	-1.48	-0.19	
Rgn	0.259	0.138	-0.87	-0.20	
Psma6	137.600	94.844	-0.61	-0.20	
Eef1b2	238.775	155.757	-0.69	-0.20	
Ndufs6	101.060	65.377	-0.70	-0.20	+
2700097O09Rik	9.527	6.421	-0.66	-0.20	
9230105E05Rik	0.074	0.028	-0.98	-0.20	
Tmbim4	23.063	15.663	-0.66	-0.20	
Gulp1	2.502	1.619	-0.70	-0.20	
2810429I04Rik	2.555	1.534	-0.79	-0.20	
Glyatl3	0.227	0.071	-1.11	-0.20	
Zgrf1	0.469	0.296	-0.74	-0.20	

Trappc2	43.603	28.448	-0.71	-0.20	
Pde6h	0.103	0.000	-1.31	-0.20	
Sftpb	0.094	0.040	-1.04	-0.20	
Pbdc1	6.643	4.499	-0.64	-0.20	
Clec7a	0.232	0.118	-0.89	-0.20	
Isca2	25.972	17.527	-0.65	-0.20	
Tmsb15a	2.761	1.483	-0.86	-0.20	
Cartpt	8.098	5.198	-0.72	-0.20	
BC039966	0.641	0.316	-0.93	-0.20	
Sub1	203.030	128.176	-0.76	-0.20	
Cd79a	0.201	0.093	-0.92	-0.20	
Vps29	106.194	69.364	-0.70	-0.20	
Uqcc2	148.300	96.403	-0.71	-0.21	+
Olfr324	0.154	0.046	-1.15	-0.21	
Rps18	1039.790	690.986	-0.71	-0.21	
Tmem14a	9.744	5.985	-0.72	-0.21	
Gm694	1.230	0.752	-0.78	-0.21	
Pla2g2c	0.132	0.057	-1.10	-0.21	
Krt71	0.341	0.199	-0.79	-0.21	
1700109G15Rik	0.191	0.054	-1.22	-0.21	
2410006H16Rik	67.561	43.449	-0.71	-0.21	
1700071K01Rik	0.060	0.000	-1.50	-0.21	
1810044D09Rik	2.742	1.570	-0.81	-0.21	
Ano5	0.582	0.388	-0.68	-0.21	
Gm10941	1.235	0.783	-0.79	-0.21	
Snrpd2	231.252	154.078	-0.66	-0.21	+
Fpr2	0.165	0.072	-1.00	-0.21	
1600020E01Rik	15.539	9.935	-0.73	-0.21	
Snhg18	3.083	1.927	-0.71	-0.21	
Tmc2	0.015	0.000	-1.27	-0.21	
Bbs5	6.018	3.990	-0.64	-0.21	

4930455G09Rik	1.454	0.820	-0.85	-0.21	
Acyp1	17.097	10.903	-0.71	-0.21	
Skap2	6.881	4.411	-0.65	-0.21	
Gtf2b	35.520	23.718	-0.66	-0.21	
Cox8a	583.14<br>1	387.602	-0.69	-0.21	
Pvalb	2.080	1.272	-0.77	-0.21	
1110059G10Rik	5.981	3.750	-0.71	-0.21	
Gnpnat1	13.864	9.537	-0.69	-0.21	
Cd48	0.403	0.199	-0.90	-0.21	
Gm4832	7.370	4.785	-0.70	-0.22	
Mybpc1	0.452	0.254	-0.75	-0.22	
Sat1	41.410	27.278	-0.71	-0.22	
Ndufaf5	12.368	8.279	-0.69	-0.22	
Mad2l1	8.240	5.256	-0.68	-0.22	
Dpm3	31.712	19.674	-0.71	-0.22	
Hist1h4k	3.309	1.917	-0.79	-0.22	
Vsig1	0.034	0.004	-1.23	-0.22	
Txn1	153.76<br>6	101.620	-0.70	-0.22	
4930558J18Rik	0.191	0.121	-0.85	-0.22	
Cox6b1	810.65<br>0	538.784	-0.72	-0.22	
3110045C21Rik	1.031	0.609	-0.80	-0.22	
Cetn4	2.660	1.656	-0.76	-0.22	
Cks1b	36.001	24.075	-0.71	-0.22	
C1galt1c1	14.266	9.199	-0.69	-0.22	+
Dynlrb1	225.11<br>3	143.488	-0.72	-0.22	+
4930529K09Rik	0.084	0.000	-1.42	-0.22	
Raet1e	0.595	0.348	-0.80	-0.22	
Ren1	0.415	0.189	-0.95	-0.22	
Lpcat2b	0.021	0.000	-1.73	-0.22	
Terf1	2.906	1.860	-0.71	-0.22	
Ndufaf2	34.168	22.342	-0.70	-0.22	
Polr2h	28.666	18.374	-0.74	-0.22	

Taf1d	17.978	11.613	-0.71	-0.22	
Mrpl47	23.043	14.643	-0.73	-0.22	
Rps16	465.26<br>6	297.818	-0.73	-0.22	
Hotairm1	5.019	3.017	-0.79	-0.22	
Snord34	57.325	35.943	-0.73	-0.22	
Zfp300	1.094	0.751	-0.69	-0.22	
Gm14124	0.171	0.092	-0.82	-0.23	
Tnfsf13b	0.274	0.140	-0.86	-0.23	
Fau	230.15<br>3	152.092	-0.70	-0.23	
Med31	15.061	9.692	-0.68	-0.23	
C030034L19Rik	0.050	0.016	-1.13	-0.23	
Psmd10	13.352	8.600	-0.73	-0.23	
Rps6	877.15<br>0	593.283	-0.68	-0.23	
Cutal	0.211	0.118	-0.91	-0.23	
D8Ertd738e	104.42<br>1	66.472	-0.69	-0.23	
Epb4.2	0.033	0.009	-1.26	-0.23	
A930016O22Rik	0.361	0.165	-0.78	-0.23	
Commd6	22.922	14.204	-0.74	-0.23	
Mrpl52	99.595	62.786	-0.71	-0.23	
Polr2i	37.542	24.903	-0.67	-0.23	
Gemin6	8.315	5.357	-0.71	-0.23	
Coq7	17.579	11.899	-0.65	-0.23	
Resp18	133.28<br>7	85.128	-0.70	-0.23	+
Gm19757	0.060	0.021	-1.07	-0.23	
4932435O22Rik	0.037	0.012	-1.08	-0.23	
Clec2d	2.587	1.555	-0.75	-0.23	
Slc15a2	4.074	2.663	-0.69	-0.23	
Cryba2	0.319	0.129	-1.11	-0.23	
Cyp11a1	0.524	0.308	-0.78	-0.23	
Ndufv2	70.397	45.182	-0.74	-0.23	+

Vbp1	112.637	70.369	-0.76	-0.23	
Bet1	13.118	8.056	-0.78	-0.23	
Sds	0.296	0.151	-0.86	-0.23	
Zbbx	0.270	0.154	-0.80	-0.24	
Ppp1r17	1.314	0.760	-0.79	-0.24	
Galr2	0.399	0.223	-0.92	-0.24	
Naca	30.877	19.918	-0.73	-0.24	+
Mrps28	16.358	10.314	-0.73	-0.24	+
Bloc1s1	96.886	58.790	-0.73	-0.24	
Fabp3	138.271	89.249	-0.71	-0.24	+
AU041133	3.706	2.420	-0.72	-0.24	
Ccdc38	1.476	0.934	-0.71	-0.24	
Commd1	67.412	41.017	-0.75	-0.24	
Snord57	3.733	1.637	-1.05	-0.24	
Hddc2	37.010	24.062	-0.71	-0.24	
Ndufa11	37.549	24.376	-0.74	-0.24	+
Pet100	10.907	6.718	-0.71	-0.24	+
Zfp938	15.363	10.086	-0.71	-0.24	
Plgrkt	25.230	15.673	-0.76	-0.24	
Rbm3	164.111	108.776	-0.69	-0.25	
Gm2694	10.471	6.031	-0.74	-0.25	
Churc1	30.995	19.966	-0.69	-0.25	
Ndufv3	55.750	36.292	-0.69	-0.25	
5033403F01Rik	0.252	0.128	-0.95	-0.25	
Bbip1	30.977	19.924	-0.72	-0.25	
BC048507	100.382	63.920	-0.73	-0.25	
Vmn1r101	0.209	0.112	-0.96	-0.25	
Gm11744	1.292	0.824	-0.76	-0.25	
Scrg1	24.412	14.366	-0.76	-0.25	
Capsl	2.685	1.483	-0.77	-0.25	
Gtf2a2	40.394	25.610	-0.74	-0.25	
Dfnb59	0.485	0.229	-0.91	-0.25	

Atp2a1	0.132	0.057	-0.93	-0.25	
Eaf2	0.195	0.100	-0.89	-0.25	
Timm10	48.343	30.741	-0.75	-0.25	
Mir24-1	1.537	0.455	-1.23	-0.25	
Ccdc58	6.885	4.126	-0.78	-0.25	
Snhg9	3.284	1.634	-0.92	-0.26	
Dpy30	57.639	36.807	-0.71	-0.26	
Mei1	0.059	0.023	-1.05	-0.26	
Ssu2	0.075	0.023	-1.11	-0.26	
Ppil3	10.569	6.608	-0.78	-0.26	+
AF251705	1.023	0.589	-0.76	-0.26	
Ttc32	2.090	1.207	-0.79	-0.26	
Mir6913	0.713	0.000	-1.33	-0.26	
Snrpf	35.597	22.024	-0.75	-0.26	
Rpl5	427.964	273.234	-0.76	-0.26	+
Snhg20	20.685	12.944	-0.73	-0.26	
Myh15	0.076	0.041	-0.92	-0.26	
Ms4a7	0.378	0.192	-0.83	-0.26	
Psmb1	176.083	114.620	-0.73	-0.26	
9430065F17Rik	0.521	0.297	-0.84	-0.26	
Lsm6	29.284	18.428	-0.76	-0.26	
Chac2	14.428	8.670	-0.76	-0.26	
Duox1	0.092	0.051	-0.85	-0.26	
4933422A05Rik	0.362	0.205	-0.84	-0.26	
Tma7	304.647	189.394	-0.73	-0.26	+
Psma4	153.789	100.252	-0.71	-0.26	
Gm21859	0.070	0.000	-1.54	-0.26	
Slc25a43	0.125	0.049	-1.07	-0.26	
Zfp931	4.086	2.568	-0.72	-0.26	
Gm10409	0.849	0.536	-0.74	-0.26	
Lipi	0.025	0.000	-1.54	-0.26	
Polr2l	34.657	21.789	-0.74	-0.26	

Gnpda2	9.084	5.685	-0.76	-0.26	
Tmem126a	63.883	39.129	-0.79	-0.27	+
Zbtb8os	7.348	4.188	-0.80	-0.27	
Ak6	37.853	24.691	-0.71	-0.27	
Mrpl27	50.612	32.539	-0.75	-0.27	
Gm6416	0.045	0.000	-1.43	-0.27	
Gdf6	0.067	0.029	-1.07	-0.27	
Mkks	8.641	5.236	-0.76	-0.27	+
Tceal1	33.958	21.326	-0.74	-0.27	
Gm6710	23.013	14.110	-0.77	-0.27	
Serf1	226.759	142.296	-0.73	-0.27	
Ssbp1	9.794	5.965	-0.76	-0.27	
Rps14	1279.230	760.739	-0.81	-0.27	
Ost4	67.634	41.723	-0.77	-0.27	
N6amt2	25.691	15.965	-0.76	-0.27	
Atp5h	146.113	89.341	-0.76	-0.27	
Itgae	0.109	0.054	-0.95	-0.27	
Tceb1	152.013	97.255	-0.77	-0.27	
Orc6	23.036	14.910	-0.71	-0.27	
Trappc2l	64.800	39.464	-0.78	-0.27	+
Skint6	0.044	0.011	-1.21	-0.27	
Gm14346	0.007	0.000	-1.49	-0.28	
Gjc2	0.403	0.229	-0.87	-0.28	
8430408G22Rik	2.873	1.339	-0.81	-0.28	
Spata31d1c	0.017	0.000	-1.59	-0.28	
BC021785	0.084	0.047	-0.94	-0.28	
Trim30e-ps1	0.138	0.071	-0.94	-0.28	
B930018H19Rik	0.042	0.005	-1.34	-0.28	
Hoxd8	0.092	0.033	-1.06	-0.28	
Cstad	0.237	0.101	-1.11	-0.28	
Rplp1	1421.670	901.867	-0.74	-0.28	

Otor	0.097	0.011	-1.28	-0.28	
Timm17a	26.716	16.557	-0.76	-0.28	+
Asb5	0.566	0.327	-0.82	-0.28	
Olfr111	0.055	0.000	-1.47	-0.28	
Ooep	4.988	3.110	-0.80	-0.28	
Gm5544	0.158	0.035	-1.32	-0.28	
BC002163	213.808	132.516	-0.75	-0.28	
Adamdec1	0.041	0.009	-1.14	-0.28	
OTTMUSG00000016609	24.105	15.105	-0.75	-0.28	
Myoz1	1.987	1.212	-0.86	-0.28	
2610001J05Rik	27.109	17.322	-0.72	-0.28	
Slc28a1	0.039	0.007	-1.29	-0.28	
Mylk3	0.053	0.019	-1.09	-0.28	
Rpp30	13.729	8.369	-0.79	-0.28	
Fgl1	0.401	0.184	-1.02	-0.28	
Mir1982	29.872	17.423	-0.83	-0.28	
Cd302	4.382	2.643	-0.78	-0.28	
Rps15a	81.058	51.352	-0.75	-0.29	
Slc25a31	0.176	0.088	-0.91	-0.29	
Tceb2	533.648	333.625	-0.76	-0.29	
4921504A21Rik	1.287	0.756	-0.81	-0.29	
Sar1b	64.490	41.166	-0.71	-0.29	+
Angptl1	0.177	0.080	-1.04	-0.29	
Ms4a6b	1.032	0.599	-0.79	-0.29	
Uxt	2.497	1.436	-0.80	-0.29	
Gm16677	3.531	2.019	-0.83	-0.29	
Fabp5	784.127	491.711	-0.77	-0.29	
Rwdd2a	21.780	13.491	-0.79	-0.29	
B930059L03Rik	0.744	0.426	-0.83	-0.29	
Rps4x	1010.990	661.786	-0.79	-0.29	
Serpina3h	0.138	0.060	-1.07	-0.29	

Riiad1	5.600	3.203	-0.81	-0.29	
Mycbp	6.950	4.133	-0.81	-0.29	
Dynlt1f	463.231	291.759	-0.73	-0.29	
Mcee	22.825	13.945	-0.77	-0.29	
1700121N20Rik	0.061	0.000	-1.46	-0.29	
Il25	0.442	0.243	-0.89	-0.29	
Dnajc19	5.422	3.290	-0.79	-0.29	
Gm6578	3.673	2.082	-0.80	-0.29	
Atp5o	374.051	228.099	-0.78	-0.29	
Tceal8	37.259	23.476	-0.75	-0.29	+
Ppih	8.466	5.217	-0.76	-0.29	
Tmem29	17.327	11.195	-0.75	-0.29	
Crisp3	0.038	0.000	-1.92	-0.29	
Speer4e	0.127	0.042	-1.18	-0.30	
Mc3r	0.385	0.220	-0.85	-0.30	
Dynlt1c	636.899	393.923	-0.73	-0.30	
Ndufa13	81.441	52.281	-0.78	-0.30	
Pfdn5	310.411	197.763	-0.72	-0.30	+
Tnnc1	1.791	1.056	-0.86	-0.30	
Cyp4a10	0.115	0.045	-1.08	-0.30	
Ndufs5	173.714	108.491	-0.76	-0.30	
Gpr34	2.183	1.310	-0.81	-0.30	
2900009J06Rik	6.149	3.623	-0.79	-0.30	
Atox1	65.895	42.363	-0.72	-0.30	+
Apold1	4.538	2.506	-0.78	-0.30	
9030619P08Rik	0.116	0.012	-1.47	-0.30	
Slc4a1	0.117	0.061	-0.90	-0.30	
Clk4	20.922	12.662	-0.76	-0.30	
1700001O22Rik	0.381	0.220	-0.92	-0.30	
2410015M20Rik	60.128	38.114	-0.73	-0.30	
Rn4.5s	8.689	5.459	-0.77	-0.30	

Gadl1	0.122	0.071	-0.88	-0.30	
1700007G11Rik	1.997	1.174	-0.82	-0.30	
Mettl10	27.546	17.401	-0.72	-0.30	
Rps3a1	1194.950	758.486	-0.73	-0.30	
D830026I12Rik	0.051	0.000	-1.38	-0.30	
Gm833	0.093	0.016	-1.43	-0.30	
Pomp	270.866	174.862	-0.76	-0.30	+
Arhgap15	0.531	0.290	-0.86	-0.30	+
Cdkn3	2.995	1.775	-0.83	-0.30	
Cenpp	2.747	1.624	-0.81	-0.30	
4930500L23Rik	0.673	0.388	-0.88	-0.31	
Hspb11	12.649	7.380	-0.78	-0.31	
Fam72a	0.429	0.235	-0.84	-0.31	
Pilra	0.741	0.434	-0.79	-0.31	
Tmem60	26.666	16.211	-0.79	-0.31	
Cd6	0.017	0.000	-1.23	-0.31	
Ascl4	0.113	0.045	-1.10	-0.31	
Smim11	18.175	10.397	-0.82	-0.31	
Tctex1d2	31.015	18.176	-0.81	-0.31	
Cphx1	0.386	0.212	-0.88	-0.31	
Uchl4	66.721	41.505	-0.76	-0.31	
Hist1h4j	4.522	2.479	-0.85	-0.31	
Airn	0.900	0.542	-0.83	-0.31	
Uchl5	16.606	10.687	-0.77	-0.31	
Skp1a	399.223	243.070	-0.78	-0.32	
Rpl27a	778.611	476.341	-0.78	-0.32	
Mrps16	52.794	32.637	-0.79	-0.32	
Romo1	137.795	85.312	-0.79	-0.32	+
Gm13247	0.472	0.272	-0.89	-0.32	
Hist1h4i	12.591	7.422	-0.81	-0.32	
Gm15694	4.416	2.576	-0.81	-0.32	

Mpc1	170.175	102.265	-0.80	-0.32	
Rpl27	736.317	443.521	-0.79	-0.32	
Wbp5	152.806	94.215	-0.77	-0.32	
Dynlt1b	756.315	470.189	-0.77	-0.32	
Eldr	0.551	0.313	-0.93	-0.32	
Meig1	4.190	2.279	-0.85	-0.32	
Fsd2	0.181	0.087	-0.95	-0.32	
Gm5148	1458.820	836.122	-0.84	-0.32	
Minos1	31.686	19.229	-0.80	-0.32	
Lgals1	36.025	21.801	-0.80	-0.32	
Tatdn1	7.475	4.523	-0.83	-0.32	
Plac1	0.044	0.000	-1.51	-0.32	
Il22	1.788	0.996	-0.88	-0.32	
Snhg5	41.620	24.795	-0.79	-0.32	
Cav3	0.045	0.000	-1.72	-0.32	
Ttpa	3.779	2.216	-0.82	-0.32	
Wdr38	12.341	7.244	-0.81	-0.32	
4930545L23Rik	0.071	0.021	-1.20	-0.33	
Tmem40	0.375	0.179	-0.97	-0.33	
Cphx2	0.384	0.211	-0.87	-0.33	
Toporsos	7.927	4.380	-0.86	-0.33	
4930481A15Rik	4.367	2.611	-0.79	-0.33	
Prpf39	29.965	18.022	-0.81	-0.33	
Sec61b	132.131	81.803	-0.78	-0.33	+
Myl6	1053.110	650.192	-0.80	-0.33	
Vmn1r149	0.209	0.112	-1.02	-0.33	
C730002L08Rik	0.052	0.021	-1.15	-0.33	
Gchfr	3.456	1.804	-0.93	-0.33	
Hrsp12	17.980	9.951	-0.86	-0.33	

Tsks	0.039	0.006	-1.38	-0.33	
1110001J03Rik	71.716	42.473	-0.82	-0.33	
Snord104	15.320	7.488	-0.93	-0.33	
Mir5136	5.088	2.307	-1.01	-0.33	
Scgb3a2	0.147	0.000	-1.40	-0.34	
Atp5e	350.696	201.050	-0.85	-0.34	
4631405J19Rik	0.200	0.084	-1.16	-0.34	
Npff	0.777	0.401	-0.94	-0.34	
Olfr654	0.102	0.010	-1.65	-0.34	
Mnd1-ps	0.486	0.242	-0.91	-0.34	
BC028528	6.699	3.946	-0.87	-0.34	
Rpl11	1034.520	619.853	-0.81	-0.34	
Srp14	149.034	86.900	-0.85	-0.34	
Eef1e1	76.747	47.818	-0.79	-0.34	
Krtap7-1	0.353	0.133	-1.21	-0.34	
Vip	0.917	0.466	-0.91	-0.34	
P2rx3	0.816	0.482	-0.85	-0.34	
Hsf5	0.073	0.030	-1.12	-0.34	
Rpl17	345.206	212.031	-0.82	-0.34	
4933440M02Rik	0.045	0.006	-1.32	-0.34	
Rps26	1011.210	576.922	-0.89	-0.34	
0610040B10Rik	7.209	4.008	-0.89	-0.34	
Aspa	1.622	0.939	-0.87	-0.34	
Gstm5	106.254	63.741	-0.82	-0.35	
Ndufb4	248.973	149.804	-0.83	-0.35	+
4933408J17Rik	5.586	3.246	-0.84	-0.35	
Rpl38	542.647	320.324	-0.83	-0.35	
Thoc7	66.444	38.204	-0.87	-0.35	+

S100b	7.828	4.167	-0.87	-0.35	
Sdhaf3	11.962	7.314	-0.80	-0.35	+
Agtr2	1.709	0.966	-0.85	-0.35	
Cysrt1	0.127	0.026	-1.41	-0.35	
Rps25	562.300	328.755	-0.86	-0.35	
Apool	5.928	3.223	-0.89	-0.35	
Uchl3	85.046	52.025	-0.81	-0.35	
Emc2	56.460	31.903	-0.87	-0.35	+
1600002K03Rik	3.147	1.727	-0.88	-0.35	
Gm32014	0.023	0.002	-1.44	-0.35	
Vmn1r168	0.242	0.111	-1.01	-0.35	
Smim8	16.957	9.980	-0.84	-0.35	
H2afz	297.479	186.599	-0.80	-0.35	
Tmem256	99.302	56.620	-0.86	-0.36	+
Pdx1	0.028	0.000	-1.43	-0.36	
Med9os	1.689	0.897	-0.95	-0.36	
Milr1	0.498	0.267	-0.89	-0.36	
Pf4	7.545	3.596	-0.86	-0.36	
Gnat1	0.476	0.238	-0.97	-0.36	
Tsix	4.364	5.133	-0.81	-0.36	
Tcp10b	0.443	0.165	-1.07	-0.36	
Tex30	7.105	4.028	-0.83	-0.36	
Clec12b	0.152	0.057	-1.15	-0.36	
Il11	0.328	0.164	-1.02	-0.36	
Psma1	101.022	62.600	-0.81	-0.36	
S100a10	99.403	60.409	-0.82	-0.36	
Gng5	124.306	70.175	-0.85	-0.36	
AA467197	0.261	0.081	-1.30	-0.36	
LOC100504039	0.197	0.059	-1.29	-0.36	
Clec12a	0.151	0.060	-1.05	-0.36	
3110056K07Rik	2.985	1.669	-0.87	-0.36	
Rgs13	0.471	0.234	-0.99	-0.36	

Gm13544	0.036	0.007	-1.29	-0.37	
Ndufb6	155.34<br>5	90.780	-0.84	-0.37	
Ren2	0.387	0.176	-0.95	-0.37	
Fam229b	16.085	9.226	-0.83	-0.37	
Anapc10	9.461	5.212	-0.88	-0.37	
Zcchc10	6.427	3.815	-0.86	-0.37	
Bola2	78.402	44.409	-0.85	-0.37	
Med6	14.324	8.249	-0.83	-0.37	
6230400D17Rik	9.063	5.422	-0.83	-0.37	
AV051173	1.306	0.678	-0.95	-0.37	
Gm10375	0.375	0.148	-1.11	-0.38	
Abracl	21.355	12.316	-0.81	-0.38	
Cebpzos	32.426	18.072	-0.88	-0.38	
Krt26	0.201	0.091	-1.08	-0.38	
Gm16381	46.530	26.186	-0.87	-0.38	
Mrpl42	81.031	44.633	-0.89	-0.38	+
Snrpg	105.71<br>3	61.019	-0.85	-0.38	
Btn2a2	0.054	0.010	-1.40	-0.38	
A930003A15Rik	0.214	0.055	-1.39	-0.38	
Gfy	0.039	0.000	-1.44	-0.38	
Actr6	20.808	11.846	-0.88	-0.38	
Rpl24	326.45<br>4	191.487	-0.89	-0.38	
Spdef	1.292	0.684	-0.89	-0.38	
Cox20	61.566	33.820	-0.89	-0.38	
LOC100504703	9.943	5.486	-0.90	-0.38	
Apoo-ps	16.411	9.442	-0.88	-0.39	
1700028P14Rik	1.993	1.010	-0.96	-0.39	
Snrpb2	50.788	29.165	-0.87	-0.39	
Anapc13	88.326	50.913	-0.87	-0.39	
Dmrtc2	0.065	0.006	-1.55	-0.39	
Gm11681	0.090	0.012	-1.26	-0.39	
Apobec2	0.485	0.203	-1.00	-0.39	
Sox15	0.216	0.082	-1.21	-0.39	

Vmn1r85	0.101	0.022	-1.37	-0.39	
Mrpl1	4.639	2.677	-0.85	-0.39	
1500026H17Rik	1.655	0.955	-0.93	-0.39	
Mrpl32	38.642	21.233	-0.92	-0.39	
Snord12	6.176	2.657	-1.11	-0.39	
C1d	62.662	35.540	-0.88	-0.39	
Mir377	2.382	0.607	-1.33	-0.39	
Irf4	0.032	0.009	-1.32	-0.39	
Magohb	16.980	9.385	-0.90	-0.39	
Gm14305	28.334	16.658	-0.88	-0.39	
1500012F01Rik	39.441	22.962	-0.88	-0.39	
3110040N11Rik	13.726	7.895	-0.86	-0.39	
Snord4a	5.898	2.800	-1.14	-0.40	
Apoo	19.283	10.936	-0.88	-0.40	
Gm6654	387.026	221.078	-0.91	-0.40	
Uqcrq	328.188	184.972	-0.90	-0.40	
BC089491	2.280	1.239	-0.96	-0.40	
1700028I16Rik	0.049	0.000	-1.49	-0.40	
Uqcr11	143.781	83.199	-0.88	-0.40	+
Rpl41	3958.490	#######	-0.87	-0.40	
Prdx1	157.600	89.981	-0.89	-0.41	+
Lrif1	4.732	2.650	-0.90	-0.41	
Sarnp	29.997	16.967	-0.87	-0.41	
Dnajc15	34.331	19.976	-0.92	-0.41	+
Serpinb12	0.093	0.024	-1.37	-0.41	
A630033H20Rik	0.173	0.073	-1.14	-0.41	
2310068J16Rik	1.343	0.690	-0.99	-0.41	
Synj2bp-cox16	25.747	14.524	-0.93	-0.41	
E130114P18Rik	12.167	6.351	-0.91	-0.41	
A330102I10Rik	8.474	4.746	-0.88	-0.41	
Barx2	0.980	0.501	-0.94	-0.41	

Myh1	0.357	0.168	-0.90	-0.41	
Klrb1	0.559	0.224	-1.19	-0.41	
Rps12	1286.870	741.222	-0.89	-0.41	
Fam183b	11.354	5.781	-0.93	-0.41	
Nup62cl	0.649	0.293	-1.05	-0.42	
Dppa2	0.040	0.000	-1.69	-0.42	
Spcs1	136.297	79.294	-0.89	-0.42	
Mcts2	26.629	14.544	-0.87	-0.42	
Chrna1	0.146	0.067	-1.06	-0.42	
Tmem128	43.632	24.763	-0.89	-0.42	
Zfp433	12.058	6.944	-0.88	-0.42	
Amn1	28.454	15.918	-0.91	-0.42	
Chchd1	114.836	63.878	-0.92	-0.42	+
Gm21283	0.136	0.000	-1.68	-0.42	
Mrps14	18.940	10.147	-0.95	-0.42	
Snhg10	6.362	3.451	-0.93	-0.42	+
Snora69	1.242	0.169	-1.40	-0.42	
Gm6251	282.758	159.677	-0.94	-0.42	
Fcer1g	6.671	3.746	-0.91	-0.42	
Acmsd	0.399	0.206	-1.01	-0.42	
C920021L13Rik	2.131	1.195	-0.96	-0.42	
Brs3	0.402	0.197	-1.01	-0.42	
1700034I23Rik	0.579	0.290	-0.99	-0.43	
Mrpl20	133.914	75.906	-0.89	-0.43	
Gm20594	2.394	1.143	-1.06	-0.43	
Stfa1	3.551	2.073	-1.03	-0.43	
Acsm4	0.027	0.005	-1.49	-0.43	
Ubl5	175.316	101.689	-0.89	-0.43	
Hsd3b2	1.280	0.682	-0.93	-0.43	
Ssr4	67.298	37.523	-0.91	-0.43	+

Ndufb5	112.802	63.289	-0.91	-0.43
Gm10714	0.173	0.047	-1.32	-0.43
Magoh	68.124	37.083	-0.94	-0.43
Rpl37a	520.269	285.567	-0.92	-0.43
C330021F23Rik	609.239	349.051	-0.90	-0.43
Pfdn1	111.222	62.593	-0.92	-0.44
Mir682	1684.340	930.848	-0.92	-0.44
Sapcd1	1.011	0.482	-1.03	-0.44
Lce1h	0.118	0.000	-1.72	-0.44
Etaa1os	0.898	0.407	-1.09	-0.44
Ly96	1.378	0.616	-1.07	-0.44
Deb1	70.452	37.885	-0.93	-0.44
Ssxb2	0.107	0.000	-1.68	-0.44
Atp5j	171.820	94.986	-0.93	-0.44
Mrps36	58.316	32.426	-0.91	-0.44
Mc1r	0.101	0.045	-1.16	-0.44
Platr14	1.123	0.613	-1.00	-0.44
Snapc5	29.542	15.735	-0.95	-0.44
Rpl32	724.301	405.647	-0.95	-0.44
Rps7	620.575	354.500	-0.92	-0.44
Lce1e	0.110	0.000	-1.72	-0.44
Krt10	5.857	3.188	-0.94	-0.44
Cxcl3	0.277	0.111	-1.16	-0.45
A930009A15Rik	0.695	0.348	-1.05	-0.45
Vmn1r63	0.234	0.105	-1.14	-0.45
Gm2016	3.291	1.781	-0.95	-0.45
Gm648	0.070	0.000	-1.85	-0.45
Gt(ROSA)26Sor	12.830	7.298	-0.91	-0.45

Myeov2	178.978	93.132	-0.99	-0.45	
Hint1	653.415	361.853	-0.93	-0.45	
Gm3279	0.062	0.000	-1.52	-0.45	
Klrc2	0.074	0.000	-1.60	-0.45	
Pdcd10	23.099	12.106	-0.96	-0.45	
Mrps21	149.853	84.692	-0.90	-0.45	
Taf5	7.189	3.986	-0.95	-0.45	
Ndufs4	89.479	48.370	-0.92	-0.45	+
4930503E14Rik	0.049	0.000	-1.74	-0.46	
D930028M14Rik	11.371	6.252	-0.93	-0.46	
Gm6607	14.411	7.684	-0.95	-0.46	
Kif16bos	0.274	0.042	-1.64	-0.46	
Ppia	6262.150	#######	-0.93	-0.46	
Skint5	0.031	0.007	-1.39	-0.46	
Gm6402	129.304	68.867	-0.97	-0.46	
Krt75	0.048	0.013	-1.44	-0.46	
Mrpl33	142.184	79.636	-0.95	-0.46	
Myl1	1.365	0.455	-1.07	-0.46	
Atad3aos	0.783	0.364	-1.06	-0.46	
Gjc3	1.588	0.874	-0.93	-0.47	
Rps11	969.194	548.138	-0.91	-0.47	
Rbm3os	14.439	9.013	-0.93	-0.47	
Gm10069	7.948	4.448	-0.94	-0.47	
Vmn1r62	0.254	0.114	-1.11	-0.47	
Klhl41	0.109	0.033	-1.29	-0.47	
Slc12a3	0.029	0.003	-1.55	-0.47	
Gm6642	25.540	13.371	-1.01	-0.47	
Cml5	0.138	0.021	-1.72	-0.47	
4930506C21Rik	25.427	13.763	-0.99	-0.47	

Ppp1r1c	0.665	0.350	-0.97	-0.47	
Rpl10l	0.858	0.423	-1.07	-0.47	
Gp5	0.183	0.070	-1.23	-0.47	
Nop10	135.658	72.464	-0.95	-0.47	+
Atpif1	313.534	164.728	-1.01	-0.47	
Sdhaf4	36.957	20.087	-0.95	-0.47	+
S100a1	21.710	11.924	-0.95	-0.47	
4933416I08Rik	0.195	0.065	-1.30	-0.47	
Snrpd1	123.415	65.273	-0.98	-0.47	
Gm5925	0.053	0.000	-1.83	-0.47	
2810405F15Rik	0.455	0.213	-1.05	-0.47	
Tomm5	77.899	42.474	-0.95	-0.47	
Hoxb8	6.852	2.948	-0.99	-0.48	
S100a8	2.186	1.000	-1.12	-0.48	
Lst1	2.927	1.463	-0.99	-0.48	
Dancr	7.455	4.032	-0.97	-0.48	
Ndufc2	227.418	125.602	-0.94	-0.48	
Cd59b	0.742	0.337	-1.10	-0.48	
Hnmt	11.383	6.142	-0.94	-0.49	
Lsm8	35.466	19.349	-0.96	-0.49	
Mir7067	10.452	4.257	-1.15	-0.49	
Lsm3	74.776	39.426	-0.99	-0.49	
Rassf6	1.100	0.574	-1.01	-0.49	
Ehf	0.057	0.016	-1.41	-0.49	
Rps27l	131.478	70.332	-0.96	-0.49	
Gm2022	2.880	1.472	-0.99	-0.49	
Wdr72	0.125	0.047	-1.17	-0.49	
Rps15a-ps4	417.171	219.156	-0.96	-0.49	
Snora17	1.623	0.547	-1.35	-0.49	
Dpt	0.521	0.195	-1.15	-0.49	

Gm6639	0.018	0.000	-1.85	-0.49	
Rps20	698.258	370.096	-1.00	-0.49	
9630013A20Rik	0.623	0.291	-1.04	-0.49	
Prss3	0.058	0.000	-1.80	-0.49	
Rhox2h	0.154	0.026	-1.54	-0.50	
Hypk	127.418	70.389	-0.93	-0.50	
5730408K05Rik	3.987	1.800	-1.09	-0.50	
Rps23	1938.480	#######	-0.98	-0.50	
Clcnkb	0.042	0.004	-1.60	-0.50	
Ndufa6	196.285	99.347	-0.99	-0.50	
Fcf1	19.779	10.314	-0.99	-0.50	
Cycs	208.704	111.344	-0.99	-0.50	+
Gm14295	66.795	34.003	-1.05	-0.50	
9330188P03Rik	2.391	1.106	-1.06	-0.50	
Lsm7	139.768	73.653	-1.00	-0.50	
Tdh	0.161	0.049	-1.40	-0.50	
Camp	0.122	0.000	-1.72	-0.51	
Smim4	6.406	3.433	-0.97	-0.51	+
Pyy	0.598	0.207	-1.29	-0.51	
0610009B22Rik	35.286	18.150	-1.02	-0.51	
Adm	1.328	0.575	-1.09	-0.51	
Mgst1	29.787	16.089	-0.97	-0.51	
Vmn1r187	0.561	0.251	-1.19	-0.51	
Gm14635	0.063	0.000	-1.73	-0.51	
Cenpw	7.382	3.725	-1.02	-0.51	
Gm10731	0.751	0.376	-1.03	-0.51	
Macc1	0.055	0.016	-1.38	-0.52	
Mcts1	71.556	37.913	-0.99	-0.52	+
Cox16	24.202	12.984	-0.98	-0.52	

E030024N20Rik	273.003	147.584	-1.02	-0.52	
C530044C16Rik	0.536	0.250	-1.15	-0.52	
Zfp534	0.371	0.166	-1.15	-0.52	
Immp1l	25.801	13.122	-1.03	-0.52	+
Sncg	72.012	35.987	-1.01	-0.52	
2900076A07Rik	8.806	4.263	-1.07	-0.52	
Tmem258	104.936	55.615	-0.99	-0.52	
BC064078	6.557	3.291	-1.02	-0.52	
Pdcd5	48.856	25.416	-1.03	-0.53	+
Znhit3	22.429	11.909	-1.00	-0.53	
Gmnn	8.291	4.244	-0.97	-0.53	
Ssxb10	0.173	0.024	-1.58	-0.53	
Snhg1	48.492	24.269	-1.06	-0.53	
Gng13	4.898	2.491	-1.06	-0.53	+
Mcemp1	0.245	0.091	-1.33	-0.53	
Immp2l	1.581	0.755	-1.09	-0.53	
Hist1h3b	1.474	0.671	-1.08	-0.53	
Bpifb6	0.097	0.025	-1.40	-0.53	
Ube2v2	93.920	51.667	-0.98	-0.53	
Snhg4	14.697	7.724	-1.02	-0.53	
Mrps33	114.033	57.518	-1.04	-0.53	
Dapl1	0.774	0.288	-1.23	-0.54	
Rps15a-ps6	400.262	212.203	-1.01	-0.54	
Akr1c13	0.933	0.410	-1.13	-0.54	
Rps24	1182.490	632.649	-1.01	-0.54	
Sirpb1b	0.042	0.000	-1.89	-0.54	
Rps17	626.015	314.428	-1.01	-0.54	+
Sf3b6	86.474	44.963	-1.03	-0.54	
1810037I17Rik	66.377	32.993	-1.01	-0.54	
S100a13	10.897	5.150	-1.10	-0.54	

Rpl34	427.56<br>0	222.599	-1.03	-0.54	+
Prss44	0.034	0.000	-1.79	-0.54	
Il5ra	0.026	0.000	-1.75	-0.54	
Epb4.1l4aos	24.403	12.482	-1.04	-0.55	
1110019D14Rik	0.942	0.427	-1.12	-0.55	
Rpl37	206.04<br>8	102.665	-1.06	-0.55	
Ndufa4l2	39.471	20.317	-1.02	-0.55	
4933427E11Rik	0.207	0.076	-1.31	-0.55	
Cox17	30.384	15.897	-1.03	-0.55	
Uqcrh	573.63<br>2	291.968	-1.02	-0.55	
Uqcr10	342.49<br>7	183.993	-0.98	-0.55	+
4831407H17Rik	0.044	0.000	-1.47	-0.55	
Ccdc150	0.074	0.027	-1.23	-0.55	
Tomm7	78.043	39.834	-1.04	-0.55	
Rpl35	1221.9<br>70	633.222	-1.04	-0.55	
Fbxw13	0.179	0.056	-1.33	-0.55	
Lce1i	0.122	0.000	-1.53	-0.55	
2900057B20Rik	0.262	0.081	-1.44	-0.55	
Gm6083	1.585	0.748	-1.15	-0.55	
Tmsb15b1	45.735	22.179	-1.06	-0.55	
Atp5k	541.01<br>3	270.604	-1.03	-0.55	
Ndufb2	203.55<br>3	102.166	-1.02	-0.55	+
Gm10451	0.234	0.069	-1.46	-0.55	
Rpl31-ps12	1878.2<br>00	948.557	-1.05	-0.56	
Rps21	1398.9<br>40	687.695	-1.05	-0.56	
Snora68	7.945	2.631	-1.17	-0.56	

Tmsb4x	3700.0<br>30	#######	-1.03	-0.56	
Cetn3	124.46<br>0	64.349	-1.03	-0.56	
Cenpk	2.630	1.294	-1.07	-0.57	
Krt25	1.102	0.475	-1.14	-0.57	
Chst9	0.366	0.151	-1.21	-0.57	
Ndufb3	282.29<br>1	139.143	-1.13	-0.57	
Rps28	861.77<br>7	433.571	-1.07	-0.57	
Vmn1r58	0.189	0.089	-1.28	-0.57	
4930425O10Rik	0.118	0.015	-1.62	-0.57	
Fam162b	0.669	0.279	-1.27	-0.57	
Rpl34-ps1	615.11<br>3	312.245	-1.08	-0.57	
Gm15772	1708.7<br>30	863.826	-1.06	-0.57	
Hspe1	201.76<br>5	100.657	-1.08	-0.57	+
Gm12191	1305.8<br>10	657.319	-1.02	-0.57	
Vmn1r59	0.543	0.243	-1.18	-0.58	
Apoa1	1.931	0.929	-1.14	-0.58	
Slc30a8	0.093	0.027	-1.33	-0.58	
Atp5j2	514.96<br>5	256.988	-1.07	-0.58	
Rpl30	1023.0<br>00	514.074	-1.08	-0.58	
Iltifb	2.208	1.022	-1.16	-0.58	
Rpl31	579.61<br>3	301.381	-1.05	-0.59	
Snord42a	9.290	3.438	-1.20	-0.59	
Gm17750	40.330	20.415	-1.04	-0.59	
Rps27a	965.43<br>5	461.308	-1.10	-0.59	

Rps29	983.066	481.034	-1.07	-0.59	
Dbi	832.199	387.866	-1.12	-0.59	
Snord35b	80.757	37.778	-1.11	-0.59	
Gm16294	0.133	0.028	-1.70	-0.59	
Ndufc1	79.548	40.385	-1.05	-0.59	
1500011K16Rik	28.394	14.121	-1.06	-0.60	
Ndufa1	189.676	89.040	-1.15	-0.60	
Gm3020	0.627	0.276	-1.15	-0.60	
Shfm1	216.855	107.342	-1.07	-0.60	
Lsmem1	0.043	0.000	-1.77	-0.60	
Gm16796	2.593	1.275	-1.10	-0.60	
Cox7a2	546.983	252.682	-1.15	-0.60	+
Rpa3	25.203	11.624	-1.08	-0.60	
Timm8b	240.605	117.800	-1.11	-0.60	+
1700001F09Rik	0.870	0.350	-1.17	-0.61	
2610037D02Rik	0.089	0.026	-1.54	-0.61	
Rpl26	1636.580	827.427	-1.09	-0.61	+
Smpx	0.153	0.018	-1.62	-0.61	
Tmsb15b2	46.740	23.089	-1.12	-0.61	
Ndufa2	143.822	70.160	-1.10	-0.62	+
Cks2	28.087	12.335	-1.11	-0.62	
Gm14327	7.181	3.232	-1.15	-0.62	
Clhc1	0.237	0.076	-1.45	-0.62	
Vmn1r56	0.277	0.124	-1.22	-0.62	
Hoxc6	1.224	0.421	-1.17	-0.63	
Krt28	0.537	0.152	-1.27	-0.63	
1110020A21Rik	0.651	0.279	-1.23	-0.63	
Gm21950	0.009	0.000	-1.68	-0.63	

Gm13154	0.444	0.190	-1.25	-0.63	
Try5	0.144	0.013	-1.84	-0.63	
Rpl23	393.662	192.511	-1.08	-0.63	
Ndufa3	371.196	178.768	-1.11	-0.63	
Rpl35a	1069.580	531.669	-1.10	-0.63	
Vmn1r186	0.561	0.251	-1.21	-0.64	
Rpl37rt	728.648	362.624	-1.11	-0.64	
Rpl36al	387.133	193.514	-1.09	-0.64	
Krt32	0.072	0.000	-1.85	-0.64	
Kbtbd12	0.095	0.030	-1.49	-0.64	
Snora65	31.399	14.073	-1.20	-0.64	
R3hdml	0.201	0.057	-1.61	-0.64	
Hist1h3h	1.853	0.803	-1.20	-0.65	
Ahsg	0.249	0.068	-1.38	-0.65	
Fkbp3	275.928	132.119	-1.12	-0.65	+
Ckm	1.252	0.364	-1.28	-0.65	
Vmn1r61	0.561	0.251	-1.36	-0.66	
Mir29c	6.354	2.461	-1.29	-0.66	
2310009A05Rik	17.727	8.364	-1.13	-0.66	
Ccl24	0.227	0.064	-1.51	-0.67	
Gm8300	1.851	0.837	-1.23	-0.67	
Naa38	63.789	29.489	-1.18	-0.67	+
Nrp	18.415	8.542	-1.15	-0.67	
1810022K09Rik	74.204	34.312	-1.18	-0.67	
Rpl36a	296.962	141.191	-1.19	-0.67	+
Tmsb15l	47.575	22.933	-1.13	-0.67	
2310040G24Rik	2.488	1.078	-1.16	-0.67	
Vmn2r3	0.078	0.023	-1.67	-0.67	
AW495222	0.413	0.109	-1.49	-0.68	

Gm20754	1.660	0.768	-1.20	-0.68
Rps27	357.135	170.730	-1.14	-0.68
Hist1h3i	1.580	0.534	-1.39	-0.68
Akr1c12	0.526	0.193	-1.28	-0.68
Capn11	0.361	0.132	-1.28	-0.68
Rps27rt	581.457	276.657	-1.16	-0.68
Rslcan18	0.358	0.145	-1.22	-0.68
Hist1h3c	1.350	0.473	-1.36	-0.69
Atp5l	897.125	416.384	-1.21	-0.69
Ctxn2	29.305	13.809	-1.16	-0.69
Glrp1	0.072	0.005	-1.85	-0.70
Pin4	45.459	19.868	-1.21	-0.70
Tnfsf18	0.061	0.010	-1.74	-0.70
Tcp10c	0.337	0.110	-1.37	-0.70
Mrps18c	96.761	42.818	-1.20	-0.70
Cpa2	3.341	1.448	-1.26	-0.70
Snora41	3.422	1.278	-1.42	-0.71
Myot	0.057	0.009	-1.79	-0.71
AF357426	1.638	0.154	-1.75	-0.71
Snora78	1.065	0.131	-1.54	-0.71
Mrln	0.691	0.185	-1.52	-0.71
Gas5	392.575	174.540	-1.23	-0.72
Zfp750	0.040	0.006	-1.64	-0.72
Mir135a-2	4.599	1.444	-1.53	-0.72
Gm5346	0.054	0.000	-2.09	-0.72
Chil3	0.065	0.007	-1.81	-0.73
BC042761	0.163	0.000	-1.78	-0.73
Reg3g	0.108	0.000	-1.98	-0.73
Alas2	3.480	1.480	-1.24	-0.73
Gm10012	467.357	202.511	-1.26	-0.73

Pcp4	421.357	200.425	-1.21	-0.73	
Smim18	54.030	23.088	-1.27	-0.74	
Pfdn4	64.644	28.264	-1.24	-0.74	
Polr2k	50.346	23.446	-1.23	-0.74	
Rhox4f	0.090	0.000	-1.72	-0.74	
2500002B13Rik	1.686	0.698	-1.30	-0.74	
Cyp4a31	0.098	0.016	-1.72	-0.74	
Gm9758	0.176	0.045	-1.52	-0.74	
Gm561	45.222	19.797	-1.25	-0.75	
Gpr132	0.034	0.000	-1.61	-0.75	
Slco1b2	0.024	0.000	-1.82	-0.76	
Snhg6	67.948	29.954	-1.27	-0.76	
Rhox2c	0.134	0.000	-1.95	-0.76	
4933431G14Rik	0.097	0.012	-1.71	-0.76	
Vmn2r5	0.067	0.008	-1.74	-0.76	
Trim29	0.083	0.029	-1.52	-0.77	
Ccl3	0.814	0.274	-1.40	-0.77	
Acyp2	20.615	8.516	-1.29	-0.78	
Myl2	0.809	0.261	-1.50	-0.78	
Snhg8	49.602	21.456	-1.24	-0.78	
Snhg3	28.126	12.132	-1.27	-0.79	
Lsm5	47.439	21.122	-1.25	-0.79	
Gm5111	0.157	0.011	-1.81	-0.79	
D630010B17Rik	0.137	0.010	-1.65	-0.79	
Gm13051	0.546	0.215	-1.31	-0.79	
B230312C02Rik	0.422	0.139	-1.47	-0.79	
Csrp3	0.166	0.021	-1.62	-0.80	
Cox7c	869.333	376.086	-1.24	-0.81	+
Hoxb7	4.859	1.707	-1.29	-0.81	
Cfc1	0.433	0.119	-1.63	-0.82	
Ndufa5	210.802	91.869	-1.26	-0.82	
DQ267102	2.723	0.436	-1.86	-0.82	

Cox6c	607.62 6	256.663	-1.31	-0.82	
Mep1a	0.050	0.007	-1.79	-0.82	
Gm15023	0.058	0.000	-1.94	-0.82	
Atoh7	0.941	0.252	-1.67	-0.82	
Mpz	0.745	0.197	-1.48	-0.82	
Sec61g	176.04 0	74.569	-1.29	-0.83	
Ccdc69	0.188	0.051	-1.60	-0.83	
Snora33	6.470	2.063	-1.49	-0.83	
5730422E09Rik	0.061	0.012	-1.93	-0.83	
Gm12338	1098.2 40	474.782	-1.30	-0.84	
Clk1	27.221	10.147	-1.37	-0.84	
Snrpe	154.88 3	62.346	-1.34	-0.84	
Ssxb3	0.346	0.051	-2.07	-0.84	
1700011H14Rik	0.121	0.018	-1.76	-0.85	
Gfi1	0.020	0.000	-2.26	-0.85	
Slirp	111.27 6	45.835	-1.33	-0.85	+
4931429P17Rik	0.042	0.000	-2.05	-0.85	
Snora3	3.832	1.031	-1.54	-0.85	
Med21	47.407	19.875	-1.34	-0.85	
Tcp10a	0.295	0.075	-1.56	-0.85	
Tbca	221.19 8	94.037	-1.32	-0.86	
C330024C12Rik	0.094	0.000	-2.15	-0.86	
Myoz2	0.186	0.018	-1.92	-0.87	
Ndufa4	1658.3 50	660.836	-1.36	-0.87	
Rpl39	1444.1 20	600.522	-1.31	-0.88	
Nr1i2	0.041	0.000	-1.98	-0.88	
9530026F06Rik	0.193	0.023	-2.13	-0.88	
Uts2b	1.346	0.445	-1.50	-0.88	

Ssxb5	0.278	0.051	-1.82	-0.88	
3830403N18Rik	0.605	0.172	-1.62	-0.88	
Gypa	0.277	0.068	-1.65	-0.89	
Mir3091	22.966	8.821	-1.42	-0.89	
Gm15421	303.67<br>0	121.897	-1.38	-0.89	
Mnd1	1.106	0.366	-1.47	-0.89	
Fabp7	2651.4<br>40	#######	-1.37	-0.89	
2010107E04Rik	490.56<br>6	195.572	-1.37	-0.89	
Snora62	1.225	0.081	-1.83	-0.89	
Cnga2	0.094	0.024	-1.60	-0.89	
Olfr731	0.149	0.000	-2.25	-0.89	
Mir330	0.907	0.000	-1.89	-0.89	
Xlr	0.505	0.185	-1.44	-0.90	
Snord47	529.97<br>6	213.740	-1.39	-0.90	
2010005H15Rik	1.089	0.313	-1.58	-0.90	
Rp1	0.012	0.000	-1.86	-0.91	
Stfa2	0.889	0.171	-1.75	-0.91	
Cox7b	305.29<br>4	127.540	-1.30	-0.91	
Rpl22l1	418.83<br>1	154.841	-1.42	-0.91	+
Dppa5a	7.884	3.035	-1.41	-0.91	
Uqcrb	238.41<br>3	94.037	-1.41	-0.92	
Akr1c19	2.861	1.088	-1.48	-0.93	
Usmg5	150.95<br>0	55.049	-1.43	-0.93	
BB031773	1.019	0.347	-1.46	-0.93	
1810019D21Rik	0.070	0.000	-2.22	-0.94	
Gm5483	1.797	0.574	-1.56	-0.94	
Reg3b	1.749	0.557	-1.51	-0.95	
Scgb3a1	0.273	0.000	-2.24	-0.95	

Cox8b	0.216	0.000	-1.87	-0.96
Mpl	0.067	0.010	-1.99	-0.96
Hoxa7	0.271	0.069	-1.74	-0.97
4930528D03Rik	0.118	0.000	-2.46	-0.98
H2-Ob	0.088	0.015	-2.02	-0.99
Rhox2f	0.195	0.000	-2.46	-0.99
Speer4d	0.122	0.018	-1.96	-1.00
Efcab10	2.632	0.840	-1.55	-1.01
Psapl1	0.039	0.000	-2.14	-1.01
Rab26os	18.777	6.538	-1.53	-1.02
Rhox2a	0.254	0.000	-2.27	-1.03
Atp1b4	0.053	0.005	-1.92	-1.03
Acbd7	4.776	1.523	-1.59	-1.04
Gm5431	0.290	0.076	-1.65	-1.05
Slc22a29	0.080	0.000	-2.09	-1.06
Gmfg	0.503	0.130	-1.82	-1.08
Vmn2r4	0.086	0.004	-2.17	-1.08
1810041H14Rik	1.200	0.288	-1.87	-1.09
Tubb1	0.592	0.118	-1.86	-1.11
Olfr1260	0.129	0.000	-2.27	-1.12
Hoxb9	0.283	0.038	-1.92	-1.13
Ssxb1	0.197	0.000	-2.45	-1.14
Tnnt3	1.697	0.451	-1.77	-1.15
Lcn8	0.297	0.000	-2.44	-1.16
Mroh5	0.093	0.014	-1.94	-1.17
Tff3	0.217	0.000	-2.03	-1.18
Zfp572	0.190	0.019	-2.17	-1.18
Tctex1d1	1.368	0.319	-1.76	-1.19
Olfr330	0.126	0.000	-2.42	-1.19
Slc22a27	0.053	0.000	-2.60	-1.21
1600014C23Rik	0.414	0.060	-2.03	-1.22
Hba-x	50.124	15.437	-1.71	-1.22
S100g	0.425	0.021	-2.25	-1.22
Tubal3	0.037	0.000	-2.20	-1.23
Hba-a1	3605.450	#######	-1.73	-1.24

Hba-a2	3580.3<br>80	#######	-1.75	-1.25
3110079O15Rik	0.645	0.112	-2.03	-1.27
Gm5128	0.083	0.013	-2.05	-1.27
5830473C10Rik	0.103	0.014	-2.29	-1.31
Ngp	0.185	0.018	-2.33	-1.32
4930486L24Rik	0.316	0.038	-2.09	-1.33
Xist	23.754	24.118	-1.87	-1.38
Allc	0.111	0.007	-2.36	-1.38
Gm7903	0.083	0.013	-2.22	-1.39
BC061195	0.236	0.000	-2.62	-1.41
Rhox2b	0.199	0.000	-2.69	-1.44
1700017N19Rik	0.114	0.012	-2.58	-1.50
Hbb-bs	2740.2<br>80	707.493	-2.02	-1.51
Hbb-b1	2774.9<br>80	716.446	-2.05	-1.54
Pramel3	0.053	0.000	-2.64	-1.56
Tnni2	1.183	0.139	-2.44	-1.66
Ppara	3.453	0.316	-2.14	-1.67
Muc6	0.051	0.003	-2.62	-1.67
S100a9	4.358	0.984	-2.21	-1.69
Krt27	0.533	0.040	-2.54	-1.70
1700003E24Rik	0.267	0.000	-2.83	-1.71
Hbb-bt	2931.1<br>10	636.691	-2.28	-1.76
Mira	1.107	0.116	-2.59	-1.80
Hbb-b2	2931.1<br>10	636.691	-2.27	-1.80
Hsd17b2	0.208	0.000	-2.84	-1.84
Myoc	0.475	0.069	-2.62	-1.92
AV320801	0.081	0.000	-3.39	-1.98
Mylpf	2.990	0.287	-2.75	-2.05
Rhox2e	0.268	0.000	-3.67	-2.10
Tnnc2	1.261	0.075	-3.09	-2.15
Afp	0.278	0.015	-3.07	-2.18

1700018B08Rik	1.911	0.000	-4.37	-3.00

Log2FC: log2-fold change

GFOLD (0.1): conservatively estimated log2-fold change at q = 0.1 confidence level

22q11: STARK_PREFRONTAL_CORTEX_22Q11_DELETION_DN

Supplemental Table 6. Differentially expressed genes in the liver at q <0.1

Gene	WT RPKM	*Jak1*$^{H595D/+;I596I/+;Y597Y/+}$ RPKM	Log2FC	GFOLD(0.1)	Inflammatory
Hbb-bh2	700.253	#######	9.35	8.71	
Fgf23	0.077	24.141	8.17	7.46	
Car4	0.002	5.071	8.19	7.28	
Sprr2a1	0.119	23.236	7.64	7.08	
Sprr2a2	0.119	23.236	7.57	6.88	
Sprr2a3	0.178	33.098	7.37	6.65	
Sprr2b	0.195	25.902	6.83	6.08	
Tnfrsf9	0.101	8.017	6.24	5.50	+
Tfap2b	0.000	0.838	6.22	5.14	
Magel2	0.005	0.526	5.77	4.99	
Plac1	0.096	4.181	5.13	4.45	
Ctsj	0.050	1.491	4.64	3.86	
Rgs4	0.127	2.408	4.23	3.65	
Cntnap1	1.773	6.002	4.16	3.45	
Esyt3	0.528	2.182	4.03	3.33	
Rtl1	0.061	1.019	4.05	3.30	
Dppa2	0.000	0.179	4.16	3.13	
Gm16223	0.066	2.180	3.62	2.89	
Sele	0.055	0.566	3.26	2.55	+
Car7	0.131	1.227	3.19	2.51	
Gpr37	0.017	0.217	3.30	2.47	
Tmem158	0.601	20.120	3.08	2.44	
Disp2	0.035	0.327	3.10	2.42	
Gm24148	0.103	3.564	3.21	2.30	
Il13ra2	0.039	0.325	3.00	2.28	
6430411K18Rik	0.081	0.783	2.96	2.27	
Ccr3	0.053	0.478	3.03	2.26	
Ch25h	0.098	0.888	2.99	2.25	
Gm5771	0.118	1.622	2.92	2.14	
Gm6034	0.017	0.205	2.89	2.10	
Sirpb1a	0.528	3.239	2.75	2.09	
Prss1	0.142	1.634	2.78	2.06	

Selp	0.540	3.178	2.61	2.03	
Hoxa13	0.007	0.140	2.97	1.98	
Mir432	0.000	1.648	2.90	1.94	
Fam180a	0.297	5.470	2.55	1.93	
Qrfpr	0.003	0.137	2.79	1.92	
Twist1	0.206	1.184	2.55	1.91	
Grrp1	0.355	1.911	2.50	1.82	
Sirpb1b	0.945	5.056	2.39	1.79	
Cxcl1	0.832	4.261	2.43	1.77	
LOC100038947	0.823	4.637	2.41	1.77	
Irg1	0.043	0.252	2.47	1.75	
Htr2b	0.020	0.173	2.45	1.72	
2610528J11Rik	2.600	24.959	2.37	1.72	
A730018C14Rik	0.007	0.114	2.74	1.70	
Il1b	1.418	4.994	2.33	1.69	+
Cpa1	0.142	0.690	2.34	1.69	
Kcnk2	0.115	0.812	2.34	1.66	
Loxl4	0.107	0.520	2.28	1.64	
4930412O13Rik	0.015	0.105	2.34	1.63	
Mir7676-2	3.954	18.797	2.27	1.61	
Dsg1b	0.014	0.104	2.48	1.60	
Csf2rb2	3.171	14.813	2.20	1.59	
Hoxb8	0.011	0.085	2.47	1.54	
Gm10791	0.066	0.356	2.25	1.53	
Krt75	0.005	0.067	2.48	1.53	
Dnaic1	0.469	2.075	2.16	1.52	
Sycp2	0.013	0.073	2.26	1.52	
Il6	0.092	0.487	2.20	1.51	+
Dsg1c	0.059	0.286	2.16	1.50	
Slc35f2	0.071	0.348	2.20	1.49	
4921517D22Rik	0.007	0.075	2.37	1.49	

Klk13	0.199	1.157	2.21	1.48
Gbx1	0.002	0.077	2.51	1.46
Car11	0.025	0.164	2.26	1.46
Mir433	0.073	1.044	2.41	1.45
Morc1	0.001	0.041	2.41	1.45
Slc4a9	1.213	4.911	2.03	1.42
Dnajc12	2.807	11.832	2.11	1.42
Asb4	0.800	4.109	2.03	1.41
Nxpe5	0.055	0.260	2.15	1.41
Htr7	0.000	0.038	2.45	1.40
Chrm2	0.092	0.399	2.05	1.40
Taar6	13.152	19.807	2.05	1.39
Krt4	0.007	0.077	2.36	1.38
Atp6v0d2	0.132	0.581	2.11	1.38
Il1r2	2.188	7.578	2.01	1.37
Msx2	0.003	0.049	2.46	1.36
Mir7676-1	4.637	18.323	1.99	1.35
4930502E18Rik	0.018	0.152	2.20	1.34
Mchr1	0.803	3.004	1.93	1.33
Psg22	0.086	0.428	2.00	1.33
Olfr824	0.000	0.087	2.33	1.32
Gje1	0.002	0.080	2.39	1.30
Socs3	3.133	11.433	1.96	1.30
Pcdh11x	0.010	0.048	2.06	1.30
Serpina3j	1.640	6.984	1.92	1.30
Nol4	0.000	0.026	2.29	1.28
Gm5150	0.424	1.676	1.96	1.28
Foxd4	0.031	0.156	2.08	1.26
4930405J17Rik	0.000	0.140	2.29	1.24
Asic2	0.003	0.031	2.23	1.23
Flrt3	0.141	0.472	1.90	1.23
Tmem35	0.169	0.648	1.96	1.22
2210408F21Rik	0.076	0.345	2.00	1.22

Sec1	0.006	0.048	2.19	1.22	
Clec4d	1.395	4.974	1.83	1.21	
Oas1e	0.104	0.419	1.80	1.20	
Ntng1	0.001	0.019	2.38	1.19	
Adora2b	0.089	0.329	1.83	1.19	+
Ipw	0.000	0.096	2.38	1.17	
Tmem212	0.017	0.164	2.10	1.15	
Tmprss9	0.097	0.474	1.88	1.15	
Slc22a28	0.008	0.065	2.17	1.15	
Fmr1nb	0.033	0.177	2.07	1.13	
Mir431	0.000	0.776	2.07	1.12	
Sbk3	0.000	0.022	2.25	1.11	
Slc25a22	8.153	45.035	1.77	1.10	
Capn13	0.000	0.023	2.28	1.10	
Timp1	0.649	2.169	1.73	1.09	+
Ms4a4c	1.061	3.560	1.83	1.08	
Pglyrp4	0.004	0.048	2.05	1.08	
Gsg1l	0.024	0.090	1.77	1.07	
Prss34	1.015	3.262	1.67	1.06	
Tarm1	0.231	0.806	1.75	1.05	
Dsg1a	0.009	0.041	1.86	1.05	
Wfdc17	2.839	9.024	1.65	1.04	
Pgbd5	0.054	0.223	1.75	1.04	
Arg2	0.491	1.520	1.67	1.04	
Tshr	0.007	0.031	1.85	1.04	
Nrbp1	9.588	57.842	1.73	1.04	
Slc41a2	0.067	0.223	1.73	1.03	
Cts6	0.002	0.058	1.95	1.03	
Mcpt8	0.927	2.968	1.67	1.03	
Cxcl11	0.011	0.077	1.97	1.03	+
Gm5416	102.696	322.489	1.60	1.02	
Gm11128	0.020	0.133	1.99	1.02	
6030498E09Rik	0.010	0.099	2.05	1.00	
A4galt	0.035	0.124	1.70	0.99	
Adamts4	0.264	0.776	1.58	0.99	

Lrg1	26.147	81.042	1.63	0.99
Kcnk16	1.071	3.576	1.71	0.99
Il1f9	0.698	2.137	1.60	0.99
Rubie	0.045	0.197	1.87	0.99
Ttbk1	0.003	0.019	1.96	0.98
Ifi27l2b	0.291	0.937	1.68	0.98
Gpx2	0.048	0.215	1.76	0.98
Speer4e	0.000	0.052	2.05	0.97
Ifi27l2a	6.920	20.124	1.56	0.97
Mir434	0.032	0.939	2.23	0.96
Trim30b	0.332	0.969	1.62	0.96
Slfn4	4.307	13.217	1.59	0.96
Cyp11a1	0.092	0.292	1.65	0.95
Sh3gl3	0.004	0.052	1.88	0.95
Fam13c	0.073	0.230	1.62	0.93
Skap1	0.039	0.129	1.72	0.93
Ubd	0.346	1.055	1.62	0.92
Gm13283	0.101	0.327	1.62	0.91
Fcrlb	0.028	0.138	1.77	0.91
Klrc1	0.260	0.686	1.53	0.90
Gm15348	0.000	0.010	1.94	0.90
Serpinb9b	0.055	0.171	1.61	0.90
Gpr34	0.027	0.105	1.69	0.90
Klrb1b	0.268	0.777	1.55	0.90
Abo	0.000	0.039	2.00	0.89
Fbxo16	0.199	0.594	1.57	0.88
Myo7b	0.017	0.059	1.61	0.88
Entpd3	0.023	0.073	1.63	0.88
Lincppara	0.006	0.047	1.88	0.88
Gm12295	0.004	0.030	1.85	0.87
Mamdc4	0.139	0.578	1.57	0.87
Serpinb11	0.000	0.031	1.94	0.86
Ptgs2os2	0.007	0.057	1.94	0.86
4930405D11Rik	0.014	0.118	1.87	0.86
Zbp1	1.174	3.293	1.45	0.86

C9	0.553	1.479	1.45	0.86	
Pth2r	0.066	0.217	1.65	0.85	
Gm4925	0.130	0.373	1.57	0.85	
Klrb1c	0.217	0.595	1.46	0.85	
Klra17	0.255	0.785	1.53	0.85	
D030045P18Rik	0.083	0.255	1.58	0.84	
Art3	0.093	0.275	1.54	0.83	
Nlrc5	0.257	0.664	1.48	0.82	
Sectm1a	0.423	1.170	1.43	0.81	
Opcml	0.014	0.050	1.60	0.81	
Csf3r	2.049	5.405	1.43	0.81	+
Gm10872	0.558	1.509	1.53	0.81	
Mfsd2a	0.004	0.036	1.88	0.81	
Zscan10	0.002	0.024	1.97	0.81	
Wfdc18	0.056	0.227	1.62	0.80	
Cyb561	0.250	0.633	1.48	0.80	
Lilr4b	4.589	11.471	1.43	0.80	
Cd300ld	1.988	5.178	1.37	0.79	
Rasd1	1.264	3.150	1.41	0.78	
Amica1	0.335	0.893	1.46	0.77	
Cacna1h	0.095	0.223	1.42	0.77	
Bcl2a1d	2.140	5.553	1.41	0.76	
Bcl2a1a	2.030	5.117	1.35	0.76	
Gpr15	0.047	0.137	1.51	0.76	
Adam2	0.008	0.037	1.69	0.76	
Tescl	0.000	0.066	2.05	0.76	
Il10	0.046	0.158	1.59	0.76	+
Ctsw	0.142	0.349	1.48	0.76	
Ms4a6d	3.413	8.947	1.37	0.76	
Ppp2r2b	0.068	0.180	1.44	0.75	
Mir6949	0.623	2.164	1.55	0.75	
Pdzd3	0.011	0.054	1.77	0.75	
Rptoros	0.460	1.132	1.40	0.74	
Hbb-bh1	824.481	#######	1.36	0.74	
Zmat4	0.000	0.008	1.84	0.74	

Wfdc12	0.013	0.089	1.56	0.74
Slc22a20	0.026	0.102	1.56	0.74
Clec4a3	2.011	5.041	1.33	0.73
Sema3a	0.194	0.510	1.38	0.73
Bcl2a1b	3.601	8.837	1.36	0.73
Tacstd2	0.225	0.537	1.36	0.73
Gm11992	0.023	0.078	1.53	0.72
Elovl3	0.016	0.069	1.65	0.72
5430425K12Rik	0.012	0.075	1.79	0.72
Vmn2r78	0.005	0.030	1.75	0.72
Gm8989	1.520	3.757	1.38	0.71
Mroh4	0.073	0.211	1.47	0.71
Psd2	0.013	0.055	1.47	0.71
Rai2	0.156	0.401	1.37	0.71
Dgkk	0.030	0.081	1.41	0.71
Serpina7	13.143	34.487	1.39	0.71
Fcgr2b	8.081	20.508	1.32	0.70
Tmigd3	0.096	0.254	1.43	0.70
Phyhipl	0.026	0.088	1.45	0.70
Syt12	0.074	0.192	1.36	0.70
Gpnmb	0.100	0.263	1.36	0.69
4933433G15Rik	0.010	0.066	1.73	0.69
Tll1	0.026	0.068	1.40	0.69
Slfn1	2.583	6.701	1.34	0.68
Gm17757	0.339	0.786	1.27	0.68
Susd3	0.323	0.792	1.32	0.68
Mir6935	7.707	29.062	1.40	0.68
Gm5622	0.040	0.112	1.40	0.67
Astn1	0.017	0.054	1.48	0.67
Gm18853	0.338	0.784	1.31	0.67
Apol9b	0.698	1.642	1.28	0.67
Btbd3	1.551	3.750	1.31	0.67
Adgrf4	0.000	0.012	1.91	0.67
Ak7	0.146	0.331	1.28	0.66

Hs3st1	0.167	0.402	1.40	0.66	
Gm5464	0.006	0.030	1.63	0.66	
Neurog1	0.000	0.039	1.88	0.66	
Phactr1	0.096	0.233	1.31	0.65	
3300002I08Rik	0.177	0.481	1.39	0.65	
Dner	0.012	0.045	1.50	0.65	
Gm10684	0.087	0.230	1.35	0.65	
Csf2rb	5.681	14.197	1.29	0.65	
1700016L21Rik	0.307	0.852	1.34	0.65	
Serpinb9c	0.026	0.090	1.55	0.65	
Gm16677	0.006	0.102	1.76	0.64	
Cd14	4.185	8.466	1.30	0.63	+
Gucy2c	0.302	0.722	1.29	0.63	
Gm9758	0.000	0.041	1.87	0.63	
Ms4a4b	0.522	1.235	1.29	0.63	
Fgl1	210.584	514.416	1.24	0.63	
Fabp4	46.290	114.020	1.27	0.63	
Txk	0.031	0.084	1.42	0.63	
9330158H04Rik	0.004	0.021	1.59	0.62	
Cd200r3	0.080	0.189	1.31	0.62	
Gm21119	0.001	0.021	1.68	0.62	
1700071K01Rik	0.012	0.068	1.60	0.62	
Hip1r	6.059	19.247	1.24	0.62	
Mir7050	9.028	21.778	1.30	0.62	
Fam78b	0.031	0.081	1.29	0.62	
Lingo3	0.000	0.014	1.80	0.61	
Lilrb4a	5.459	12.498	1.23	0.61	
Ccdc36	0.008	0.037	1.49	0.61	
Tex14	0.011	0.033	1.40	0.61	
Gm11110	0.077	0.224	1.42	0.61	
Lrrc7	0.004	0.015	1.55	0.61	
Jakmip2	0.001	0.013	1.76	0.61	
Eif2s3y	2.999	6.379	1.23	0.61	

Oscar	0.015	0.061	1.52	0.61	
Slc2a6	0.169	0.422	1.26	0.60	
Pcdh19	0.065	0.160	1.28	0.60	
Lgals2	0.348	0.746	1.24	0.60	
Stk32b	0.725	1.018	1.25	0.60	
Gpr35	0.352	0.741	1.16	0.60	
Mettl24	0.081	0.200	1.37	0.60	
Ly6k	0.012	0.091	1.60	0.60	
Gm8979	1.581	3.677	1.25	0.59	
Hottip	0.000	0.016	1.77	0.59	
Gm29684	0.014	0.059	1.51	0.59	
S100a4	1.474	3.433	1.25	0.59	
Klrc2	0.236	0.557	1.29	0.59	
Rlbp1	0.102	0.214	1.33	0.59	
Oas1g	6.077	14.241	1.17	0.59	
Adrb1	0.379	0.955	1.29	0.59	
Klre1	0.099	0.258	1.35	0.59	
Edil3	0.010	0.032	1.44	0.58	
Rgs1	4.501	8.771	1.21	0.58	+
C430049B03Rik	0.509	1.123	1.18	0.58	
Ubxn10	0.022	0.049	1.31	0.58	
Grik2	0.001	0.012	1.79	0.58	
1700028M03Rik	0.000	0.101	1.67	0.58	
Oas1a	8.691	20.389	1.25	0.58	
Ccl3	0.211	0.499	1.28	0.57	
Mir127	0.043	0.925	1.85	0.57	
Spatc1	0.087	0.213	1.23	0.57	
Oxgr1	0.000	0.009	1.71	0.56	
Cd2	0.288	0.653	1.29	0.55	
Cd244	0.137	0.334	1.23	0.55	
Fam174b	1.205	2.679	1.16	0.55	
Gm8369	0.145	0.360	1.25	0.55	
Mira	0.076	0.199	1.37	0.55	
Lrrc2	0.018	0.052	1.36	0.55	

Hapln4	0.004	0.023	1.53	0.54	
Ctla2a	8.996	19.541	1.12	0.54	
Fxyd5	4.050	9.092	1.18	0.54	
Klrc3	0.055	0.159	1.40	0.54	
Gm11978	0.007	0.054	1.48	0.54	
Hcar2	0.491	1.046	1.19	0.54	
AU022793	0.062	0.164	1.30	0.54	
Ccl6	4.957	10.510	1.12	0.54	
Sytl3	0.074	0.180	1.31	0.54	
Kcns3	0.022	0.062	1.31	0.53	
Ccl5	0.975	2.317	1.21	0.53	+
Apol9a	0.564	1.336	1.19	0.53	
Ccno	0.290	0.684	1.18	0.53	
Oasl1	1.476	2.890	1.11	0.53	
AI197445	0.007	0.023	1.52	0.53	
Platr17	0.146	0.379	1.28	0.53	
Adora3	0.145	0.337	1.25	0.53	
Klra2	0.658	1.529	1.18	0.53	
Trem1	1.927	3.910	1.12	0.53	
Ms4a6b	2.759	6.134	1.14	0.52	
Ccna1	0.019	0.074	1.41	0.52	
Klf13	5.531	18.098	1.19	0.52	
Alpk2	0.008	0.027	1.32	0.52	
Selplg	5.146	11.711	1.13	0.52	
Lrrc10b	0.076	0.173	1.29	0.52	
Gm6093	0.044	0.109	1.34	0.52	
Lipg	0.052	0.124	1.21	0.51	
Enho	3.842	9.157	1.19	0.51	
5430403N17Rik	0.007	0.035	1.56	0.51	
F630042J09Rik	0.029	0.073	1.34	0.50	
Ang3	0.960	2.170	1.20	0.50	
1810063I02Rik	0.022	0.172	1.50	0.50	
Clec4e	0.599	1.323	1.15	0.50	

4930542D17Rik	0.004	0.039	1.70	0.50
Tnnc1	1.068	2.143	1.09	0.50
B3gnt7	3.901	7.506	1.11	0.50
Olfr1141	0.000	0.044	1.66	0.50
Dnah2	1.287	2.115	1.10	0.50
Lrrc9	0.006	0.021	1.41	0.50
Lmtk3	0.025	0.062	1.29	0.50
Spred3	0.140	0.322	1.20	0.49
Bhlhe22	0.041	0.100	1.23	0.49
2210417A02Rik	0.160	0.417	1.26	0.49
Phf11a	2.990	5.786	1.08	0.49
9230105E05Rik	0.016	0.060	1.43	0.49
Cutc	2.536	7.668	1.19	0.49
Clec2i	0.196	0.443	1.19	0.49
Gm10024	0.000	0.083	1.53	0.49
Ccr1	2.155	4.772	1.16	0.49
Ccl8	0.059	0.230	1.44	0.49
E230029C05Rik	0.499	1.047	1.12	0.49
Ang	7.512	16.356	1.14	0.48
Skor2	0.002	0.015	1.34	0.48
9230102K24Rik	0.072	0.194	1.23	0.48
Ackr2	0.685	1.472	1.12	0.48
Mta1	7.401	24.819	1.15	0.48
Cxcl3	0.048	0.139	1.33	0.48
Ang6	0.513	1.047	1.13	0.47
Fam43a	2.986	6.483	1.11	0.47
Il4ra	4.210	8.752	1.06	0.47
4933411E08Rik	0.005	0.035	1.46	0.47
2200002D01Rik	1.259	2.577	1.13	0.47

Podn	0.196	0.521	1.14	0.47	
Ang2	0.187	0.551	1.30	0.47	
Scn1b	0.753	1.620	1.11	0.47	+
Scin	0.018	0.045	1.28	0.47	
Alox8	0.001	0.013	1.60	0.47	
Gm6904	2.521	5.217	1.10	0.47	
Trim69	0.007	0.040	1.56	0.47	
Lmcd1	0.886	1.679	1.07	0.47	
Serpina3a	0.370	0.701	1.07	0.47	
Btnl6	0.004	0.029	1.53	0.47	
Ly6a	6.019	11.091	1.07	0.47	
Tmem108	0.496	0.995	1.08	0.46	
Steap1	0.299	0.649	1.18	0.46	
Plac8	52.150	110.405	1.05	0.46	
Klrd1	0.238	0.564	1.18	0.46	
Hamp	149.544	299.753	0.99	0.46	
Cyp4x1os	0.033	0.091	1.32	0.46	
Stmnd1	0.000	0.026	1.70	0.45	
Gm4477	0.040	0.129	1.25	0.45	
Rnf39	0.029	0.090	1.37	0.45	
Clec7a	1.870	3.998	1.11	0.45	
2010204K13Rik	0.988	2.601	1.08	0.45	
Krt82	0.027	0.079	1.30	0.45	
Tlr2	1.159	2.396	1.03	0.45	+
Il2rb	0.471	0.910	1.03	0.44	+
Insl3	0.995	2.015	1.10	0.44	
A930024E05Rik	0.045	0.115	1.18	0.44	
5830454E08Rik	0.122	0.309	1.23	0.44	
Ccr2	1.337	2.932	1.09	0.44	
Gm5878	0.029	0.071	1.15	0.44	
Sh2d1b2	0.061	0.251	1.45	0.44	
Ptpn13	2.485	3.539	1.05	0.44	
Emb	4.358	8.887	1.02	0.44	

A530016L24Rik	0.015	0.042	1.39	0.44	
C4b	132.824	289.119	1.03	0.43	
Casp1	1.314	2.622	1.00	0.43	
Cpa3	0.442	0.956	1.04	0.43	
4933427E11Rik	0.012	0.068	1.53	0.43	
Batf	0.907	1.901	1.16	0.43	
Prok2	0.076	0.206	1.34	0.43	+
Crtac1	0.031	0.090	1.21	0.43	
Plekha4	0.299	0.617	1.08	0.43	
Oas3	2.539	5.381	1.08	0.43	
Ccl2	0.409	0.811	1.06	0.42	+
Gm10125	0.002	0.020	1.54	0.42	
Gm13285	0.160	0.225	1.05	0.42	
Ifitm1	7.861	16.215	1.04	0.42	+
Slc22a14	0.012	0.043	1.26	0.42	
Klrg2	0.017	0.059	1.39	0.42	
Gpr65	2.165	4.148	1.04	0.42	
Scn7a	0.011	0.031	1.19	0.42	
Tph1	0.001	0.011	1.60	0.42	
Slc12a8	0.024	0.058	1.12	0.42	
Mmp27	0.086	0.206	1.17	0.41	
Abcc12	0.000	0.009	1.54	0.41	
Spaca1	0.020	0.096	1.40	0.41	
Mir6381	1.704	3.856	1.20	0.41	
Fam189a1	0.039	0.082	1.12	0.41	
Slc22a30	0.014	0.047	1.32	0.41	
Cyp21a1	0.088	0.208	1.13	0.41	
Ifit1bl2	0.260	0.520	1.04	0.41	
Ifit1	3.168	6.529	1.05	0.41	
Bmp3	0.008	0.026	1.33	0.40	
Apol11a	0.192	0.395	1.08	0.40	
Kcnj2	0.265	0.547	1.03	0.40	+
Krt85	0.040	0.106	1.20	0.40	
Gm5086	0.007	0.036	1.44	0.40	

Cfap69	0.248	0.486	1.08	0.40	
BC048546	1.784	3.661	1.03	0.40	
C4a	58.160	118.355	1.02	0.40	
Spink10	0.000	0.026	1.32	0.39	
Cd200r2	0.052	0.138	1.25	0.39	
Dnaaf3	0.061	0.140	1.15	0.39	
Ctla2b	6.862	13.856	1.06	0.39	
Fosb	0.362	0.519	1.04	0.39	
1700001G17Rik	0.099	0.218	1.18	0.39	
Irf7	15.950	33.147	1.06	0.39	+
Casp4	1.097	2.333	1.07	0.39	
Terc	0.038	0.178	1.33	0.39	
Ang4	0.549	1.126	1.06	0.39	
Gm11711	2.931	5.237	1.01	0.38	
Oas1c	0.413	0.839	0.99	0.38	
E230016K23Rik	0.008	0.034	1.37	0.38	
Ang5	0.945	2.137	1.12	0.38	
Slco1c1	0.004	0.017	1.42	0.38	
Asprv1	5.857	12.664	1.02	0.38	
Kcnj15	0.067	0.144	1.06	0.38	
Adam8	1.757	3.361	1.07	0.38	
Ugt3a1	0.603	1.197	1.00	0.37	
Gem	0.276	0.467	0.94	0.37	
Gm15319	0.001	0.021	1.47	0.37	
Ccr8	0.000	0.031	1.77	0.37	
Gm11710	2.931	5.237	1.03	0.37	
Tcte2	0.037	0.109	1.20	0.36	
Tspan17	0.998	1.835	1.02	0.36	
Bst1	1.209	2.459	1.02	0.36	
Cxcl5	0.073	0.156	1.14	0.36	
Rasal1	0.063	0.133	1.05	0.36	
Mir7062	0.688	1.694	1.23	0.36	
9030619P08Rik	1.148	2.328	1.04	0.36	

Retnlg	47.549	95.193	0.98	0.36
Rbm44	0.085	0.179	1.03	0.36
4930539E08Rik	0.042	0.088	1.04	0.36
Tmem139	0.045	0.133	1.26	0.36
Ifit1bl1	0.194	0.387	1.05	0.36
Ddx60	0.377	0.713	0.95	0.35
Mgarp	0.299	0.630	1.01	0.35
Oas2	1.671	3.197	0.98	0.35
Galnt18	1.096	2.176	1.00	0.35
Gm38415	0.000	0.012	1.63	0.35
4931402G19Rik	0.259	0.527	1.10	0.35
Clec4a4	2.630	5.024	1.02	0.35
Il12rb1	0.055	0.111	1.02	0.35
Gm4841	0.049	0.102	1.08	0.35
Arid5a	0.624	1.131	0.97	0.35
Retnla	4.221	8.012	0.95	0.35
Cd300lf	4.408	8.603	0.96	0.35
Actbl2	0.000	0.015	1.41	0.35
Tcaf2	0.078	0.149	1.03	0.34
Synpo	0.512	0.979	1.04	0.34
Grm6	0.009	0.023	1.24	0.34
Lgals3	8.943	17.683	0.98	0.34
Fam50b	0.064	0.139	1.19	0.34
Cd300lh	3.408	5.575	1.02	0.34
Pcsk1n	0.001	0.019	1.47	0.34
Ascl3	0.004	0.067	1.45	0.34
Apol6	0.002	0.014	1.40	0.34
Lbx1	0.004	0.068	1.46	0.34
9530036O11Rik	0.005	0.020	1.26	0.33
Card11	0.106	0.209	1.01	0.33
Tnfsf11	0.011	0.037	1.30	0.33
Smtnl1	0.043	0.090	1.09	0.33
Hdc	3.188	6.252	1.04	0.33

Gbp2	2.331	4.193	0.97	0.33
Gm13289	0.121	0.195	0.98	0.32
Pinlyp	0.000	0.040	1.34	0.32
Slc35g2	0.359	0.739	1.02	0.32
Adrb3	0.128	0.221	0.99	0.32
Ccl12	0.231	0.516	1.10	0.32
Myl4	1.950	3.433	0.95	0.32
Gvin1	4.011	7.756	0.96	0.32
Snord100	6.052	10.820	1.00	0.32
Derl3	0.070	0.167	1.12	0.32
Milr1	1.176	2.175	0.93	0.32
Cpa2	0.041	0.098	1.11	0.32
Gnat1	0.655	1.368	1.00	0.32
Sh2d1b1	0.211	0.452	1.08	0.32
Mtl5	0.033	0.060	1.03	0.32
Crtam	0.027	0.073	1.24	0.31
Gm2560	0.003	0.039	1.46	0.31
Serpina3n	153.397	318.609	0.96	0.31
Syt13	0.057	0.111	1.04	0.31
4930509K18Rik	0.074	0.150	1.06	0.31
Hbb-y	1.809	3.243	0.95	0.31
Fgf11	0.155	0.304	0.95	0.31
2810047C21Rik1	0.589	1.146	1.02	0.31
Gm1141	0.003	0.019	1.42	0.31
Hoxa1	0.100	0.214	1.03	0.31
Eqtn	0.014	0.059	1.33	0.30
Mir7027	0.084	0.572	1.34	0.30
5430416O09Rik	0.028	0.058	1.10	0.30
Trem2	0.658	1.207	0.93	0.30
Mir1247	0.369	1.148	1.27	0.30
Msl3l2	0.352	0.666	0.96	0.30
2010003K11Rik	0.454	0.807	0.91	0.30

Pcdh10	0.001	0.005	1.26	0.30	
Serpina3g	11.795	22.845	0.91	0.30	
Cabp1	0.074	0.194	1.10	0.30	
Cd3g	0.246	0.512	1.01	0.30	
Rnf222	0.049	0.096	1.04	0.30	
Lppr5	0.006	0.023	1.23	0.29	
Ccdc160	0.083	0.176	1.05	0.29	
H2-Oa	0.205	0.382	0.98	0.29	
Rtp4	6.031	11.413	0.95	0.29	+
Gm996	0.021	0.049	1.08	0.29	
Lpo	0.189	0.388	1.05	0.29	
Vmo1	0.158	0.342	1.05	0.29	
Ctsq	0.000	0.031	1.56	0.29	
A2m	76.930	140.913	0.89	0.28	
Clec4a1	3.034	5.448	0.89	0.28	
Prdm8	0.015	0.045	1.16	0.28	
Gm15326	0.049	0.099	1.14	0.28	
Tmem171	0.073	0.153	1.11	0.28	
Cd83	1.770	3.035	0.92	0.28	
Il18	2.907	5.295	0.92	0.28	+
Olfm1	1.280	2.428	0.87	0.28	
Ccr5	1.061	1.802	0.88	0.28	
1700061I17Rik	0.049	0.111	1.05	0.28	
Ncr1	0.095	0.197	0.93	0.28	
Hamp2	48.205	92.977	0.89	0.27	
Nlrc4	0.229	0.425	0.92	0.27	
Armc3	0.037	0.078	1.04	0.27	
Mir6537	0.165	0.535	1.23	0.27	
Serpina10	27.704	52.707	0.88	0.27	
Il27	0.099	0.259	1.07	0.27	
Lpxn	0.752	1.326	0.85	0.27	
Tpd52	4.804	8.754	0.86	0.27	
Xlr	0.320	0.582	0.90	0.27	
B930025P03Rik	0.544	1.002	0.87	0.27	
Fgr	1.856	3.521	0.94	0.27	

Mx1	0.654	1.112	0.91	0.27	
AU040972	0.006	0.034	1.48	0.27	
Gm5766	0.115	0.214	0.92	0.27	
4930405A21Rik	0.055	0.136	1.19	0.27	
Slc6a3	0.109	0.201	0.94	0.26	
Ms4a2	0.202	0.378	0.89	0.26	
Maf	5.014	13.616	0.91	0.26	
Fcgr4	4.706	9.215	0.95	0.26	
Slfn2	10.332	18.963	0.87	0.26	
Gja1	1.944	3.297	0.85	0.26	
4921518K17Rik	0.003	0.014	1.25	0.26	
Dhrs9	0.377	0.673	0.87	0.26	
Hyou1	6.713	13.594	0.89	0.26	
Ms4a6c	5.107	9.733	0.94	0.26	
Brinp1	0.126	0.219	0.93	0.26	
Serpine1	34.476	28.883	0.87	0.26	+
Bend4	0.121	0.218	0.91	0.26	
A430105I19Rik	0.327	0.580	0.89	0.26	
Orm3	0.341	0.632	0.88	0.26	
Zfp458	0.107	0.208	0.89	0.26	
Lipm	0.006	0.026	1.38	0.26	
Myrfl	0.001	0.017	1.50	0.25	
Celsr3	0.069	0.124	0.88	0.25	
Ly6g6d	0.120	0.292	1.08	0.25	
Zkscan5	1.405	3.006	0.90	0.25	
Ifi44	1.138	2.097	0.90	0.25	
Cytip	1.605	2.999	0.93	0.25	
Pabpc1l	0.072	0.137	0.96	0.25	
Eya2	0.034	0.079	1.03	0.25	
Aif1	4.098	7.332	0.86	0.25	
Apol10b	0.107	0.219	1.04	0.25	
Tmem154	1.206	2.184	0.92	0.25	
Kcng1	0.038	0.081	1.10	0.25	

4930593A02Rik	0.026	0.082	1.25	0.25	
Adgre4	0.697	1.266	0.93	0.25	
Dio3os	1.327	2.039	0.88	0.25	
Olfr54	0.292	0.544	0.96	0.25	
2610037D02Rik	0.012	0.032	1.16	0.25	
Psmb9	4.235	7.916	0.91	0.25	
Ptrh1	0.308	0.580	0.92	0.25	
Lst1	6.343	11.263	0.93	0.25	
AW112010	10.348	18.440	0.88	0.25	
Prss3	1.938	3.740	0.86	0.24	
Cxcr2	2.162	4.304	0.91	0.24	
Rnase4	17.604	33.618	0.88	0.24	
Klhl32	0.010	0.035	1.23	0.24	
Phf11b	6.029	11.448	0.95	0.24	
Emid1	0.898	1.602	0.90	0.24	
Mcf2l	4.348	5.538	0.92	0.24	
Ly6d	2.816	4.847	0.85	0.24	
Upk1a	0.021	0.059	1.13	0.23	
Apold1	1.433	2.723	0.92	0.23	
4930467E23Rik	0.001	0.020	1.31	0.23	
S100a6	9.125	17.445	0.88	0.23	
Sh2d2a	0.126	0.218	0.90	0.23	
Gm11651	0.010	0.047	1.40	0.23	
Tnfrsf10b	0.254	0.411	0.87	0.23	
Bcl2l14	0.041	0.093	1.02	0.23	
Hpse	1.168	2.131	0.89	0.23	
Socs1	0.678	1.191	0.87	0.23	
Ramp3	0.159	0.328	1.00	0.23	
Ppfia4	0.280	0.485	0.86	0.23	
Gda	2.930	5.330	0.84	0.23	
Gm10334	1.892	3.709	0.85	0.23	
Ifi205	14.405	25.774	0.83	0.23	
C3ar1	0.463	0.795	0.88	0.23	+

Slc2a3	5.132	8.846	0.88	0.22
Isg15	53.107	97.578	0.92	0.22
Krt6b	0.016	0.047	1.15	0.22
Mir7067	6.869	10.838	0.94	0.22
Fosl2	2.748	4.680	0.84	0.22
Ctsc	11.532	21.137	0.83	0.22
Mir6954	0.000	0.589	1.51	0.22
Slco4a1	0.387	0.681	0.84	0.22
Cyp7b1	0.131	0.226	0.89	0.22
Batf2	0.172	0.318	0.89	0.22
Jdp2	2.534	4.274	0.86	0.22
Slc22a3	0.136	0.242	0.89	0.22
Tmem74	0.004	0.024	1.34	0.22
Cnih2	0.060	0.120	1.04	0.22
Ppp1r3d	0.333	0.600	0.91	0.22
Cp	49.779	95.220	0.83	0.22
Frmd3	0.024	0.043	0.98	0.22
Oasl2	3.704	6.198	0.80	0.22
Rsph1	0.432	0.845	0.85	0.22
Mnda	20.734	36.343	0.82	0.22
Sorcs1	0.053	0.097	0.89	0.22
B3galt4	0.509	0.900	0.83	0.22
Cd52	28.289	51.593	0.86	0.22
Mir7674	0.144	0.841	1.28	0.22
Car13	0.835	1.488	0.84	0.22
Dcstamp	0.006	0.026	1.24	0.22
Ppp1r3f	0.081	0.150	0.88	0.21
Krt77	0.002	0.027	1.26	0.21
4930552P12Rik	0.021	0.050	1.07	0.21
1810034E14Rik	0.793	1.405	0.85	0.21
Gpr179	0.001	0.005	1.23	0.21
Limch1	0.142	0.255	0.89	0.21
Ubap1l	0.018	0.047	1.16	0.21
Gzma	6.249	10.827	0.85	0.21

Pilrb1	3.409	6.042	0.84	0.21	
Gm4070	4.013	7.759	0.85	0.21	
Prdm14	0.008	0.023	1.13	0.21	
Lsmem1	0.010	0.044	1.35	0.20	
Vpreb3	9.586	17.013	0.82	0.20	
Ly6g6c	0.082	0.178	0.99	0.20	
Adam1b	0.045	0.096	0.99	0.20	
Epsti1	1.675	3.057	0.85	0.20	
Gipr	0.084	0.204	0.93	0.20	
4933412E12Rik	0.239	0.417	0.89	0.20	
5730507C01Rik	0.049	0.095	0.95	0.20	
Sp100	7.444	12.967	0.86	0.20	
Il7r	1.317	2.116	0.81	0.20	+
Mir6989	2.514	4.437	0.91	0.20	
Ccl22	0.070	0.130	0.99	0.20	+
Gpr18	0.095	0.180	0.92	0.20	
Tlr13	1.417	2.417	0.83	0.20	
Lilra6	3.652	6.244	0.80	0.20	
Il6st	9.864	16.241	0.74	0.20	
Ncf2	4.640	7.929	0.79	0.20	
Psmb8	7.916	13.996	0.81	0.20	
Ly6f	3.268	5.524	0.79	0.20	
Mei4	0.024	0.060	1.07	0.20	
Slc7a9	0.078	0.160	0.97	0.20	
Blk	1.360	2.378	0.85	0.20	
4930426D05Rik	0.067	0.120	0.93	0.20	
Ccser1	0.004	0.017	1.23	0.19	
Tnfaip8l3	0.030	0.069	1.06	0.19	
Casq1	0.060	0.122	1.01	0.19	
Itga2b	7.913	13.493	0.83	0.19	
Stfa3	199.451	354.589	0.80	0.19	
Clec4b1	1.903	3.266	0.81	0.19	

5430421N21Rik	0.456	0.744	0.85	0.19
Snord89	0.419	1.002	1.04	0.19
A530064D06Rik	1.201	2.187	0.79	0.19
Pde6h	0.000	0.053	1.30	0.19
Ntn4	1.128	1.973	0.79	0.19
C130050O18Rik	0.441	0.734	0.77	0.19
Gm13290	0.121	0.195	0.89	0.19
Thbd	1.630	2.723	0.78	0.19
BC094916	1.948	3.375	0.86	0.19
Pira7	8.314	14.691	0.80	0.19
Clec4a2	4.956	8.831	0.83	0.19
Cib3	0.009	0.064	1.38	0.19
Snord34	29.666	39.954	0.81	0.19
Fcrla	3.109	5.474	0.81	0.19
Pdgfb	0.685	1.170	0.77	0.19
Cxcl16	1.985	3.619	0.86	0.18
Trim6	0.215	0.351	0.86	0.18
Gnb5	0.294	0.465	0.76	0.18
Dpep2	0.060	0.122	0.95	0.18
Gm6377	0.292	0.485	0.78	0.18
Arl5c	2.526	4.578	0.82	0.18
I830077J02Rik	2.041	3.699	0.86	0.18
Spib	3.355	5.607	0.77	0.18
Pyhin1	3.827	6.574	0.81	0.18
Arntl2	0.112	0.193	0.89	0.18
Adam21	0.002	0.012	1.41	0.18
Gm12250	0.701	1.170	0.81	0.18
Ctss	28.213	48.702	0.79	0.18
H2-DMb2	1.636	2.711	0.81	0.18
Pstpip1	1.862	3.253	0.80	0.18
Cldnd2	0.442	0.841	0.94	0.18
Mmp8	12.331	21.773	0.77	0.18
Fcgr1	2.416	4.167	0.80	0.18

1110058D11Rik	0.018	0.071	1.13	0.17
Hap1	0.264	0.432	0.75	0.17
Gm7609	1.518	2.689	0.86	0.17
Fam131b	0.033	0.060	0.94	0.17
Ablim2	0.046	0.086	0.89	0.17
Stc2	0.532	0.854	0.81	0.17
Evi2a	1.509	2.664	0.84	0.17
Cd7	0.509	0.883	0.83	0.17
Dusp5	1.032	1.743	0.88	0.17
Mcemp1	6.068	10.618	0.79	0.17
AI607873	3.828	6.702	0.79	0.17
Serpina11	64.282	122.345	0.81	0.17
Gm38407	0.099	0.162	0.81	0.17
Gm5382	0.018	0.104	1.42	0.17
Gm15350	0.216	0.489	0.94	0.17
Pcp4	0.047	0.101	0.96	0.17
Fasl	0.034	0.076	0.97	0.17
Khdrbs3	1.233	2.086	0.77	0.17
Tnni2	0.272	0.494	0.90	0.17
Ifitm3	109.196	188.025	0.73	0.17
1700123M08Rik	0.085	0.160	0.99	0.17
Slc15a3	3.395	5.680	0.81	0.17
9330175E14Rik	0.014	0.046	1.22	0.17
Pirb	13.499	23.587	0.74	0.16
Ell3	0.918	1.466	0.79	0.16
Stmn1-rs1	0.124	0.259	0.96	0.16
Gm13271	0.099	0.225	1.00	0.16
Entpd2	1.925	3.209	0.75	0.16
9830107B12Rik	0.454	0.838	0.83	0.16
Pilrb2	2.517	4.336	0.81	0.16
Tubb3	0.804	1.356	0.83	0.16
Ptprcap	6.811	12.078	0.84	0.16

1700125G22Rik	0.000	0.027	1.14	0.16	
Fpr2	6.886	11.958	0.78	0.16	
Slc35e4	0.173	0.275	0.82	0.16	
Thbs1	22.819	37.008	0.77	0.15	
Mndal	13.442	22.424	0.78	0.15	
Ifi204	15.634	27.360	0.80	0.15	
Zfp273	0.349	0.588	0.83	0.15	
LOC547349	199.353	372.211	0.83	0.15	
H2-D1	142.401	247.846	0.74	0.15	
Sncb	0.005	0.034	1.28	0.15	
2210407C18Rik	0.007	0.054	1.28	0.15	
Prdm6	0.101	0.177	0.84	0.15	
Gal3st4	0.118	0.199	0.83	0.15	
Klrk1	0.230	0.385	0.84	0.15	
Acpp	0.464	0.750	0.77	0.15	
Rbfox3	0.065	0.114	0.79	0.15	
BC053393	0.000	0.012	1.11	0.15	
Gpsm3	6.535	11.316	0.80	0.15	
Parp8	0.827	1.336	0.75	0.15	
H2-Ob	0.428	0.688	0.78	0.15	
Pira4	8.404	14.855	0.79	0.15	
Slc5a11	0.035	0.070	1.00	0.15	
Pld4	6.141	10.394	0.78	0.14	
Ifi27	11.001	18.217	0.75	0.14	
Gfra4	0.026	0.067	1.08	0.14	
H2-Eb1	1.527	2.698	0.84	0.14	
Tcp11l1	0.331	0.555	0.76	0.14	
Gm7008	0.038	0.132	1.21	0.14	
Gm1968	0.148	0.288	1.01	0.14	
Trim30d	1.530	2.665	0.79	0.14	
Alox12e	0.024	0.048	0.99	0.14	
Bst2	28.287	46.673	0.71	0.14	+
Stat4	0.246	0.400	0.75	0.14	
Irf4	1.235	1.936	0.77	0.14	

Dpysl3	0.833	1.381	0.81	0.14	
Fam84a	0.289	0.474	0.76	0.14	
D830046C22Rik	0.370	0.612	0.75	0.14	
Ctnnd2	0.025	0.043	0.83	0.14	
Guca1a	0.399	0.728	0.86	0.14	
C5ar1	1.638	2.803	0.77	0.14	+
Kdm5d	0.325	0.636	0.82	0.14	
5830416P10Rik	0.264	0.445	0.77	0.13	
A630001G21Rik	1.265	1.897	0.72	0.13	
Nlrp3	0.348	0.561	0.82	0.13	+
Fcgr3	19.804	32.919	0.72	0.13	
1810053B23Rik	0.093	0.146	0.80	0.13	
Vegfc	1.696	2.960	0.78	0.13	
Ms4a7	0.363	0.614	0.81	0.13	
Dusp2	1.653	2.681	0.79	0.13	
A930001C03Rik	0.218	0.375	0.78	0.13	
Xaf1	1.483	2.404	0.80	0.13	
Vmn2r1	0.000	0.004	1.32	0.13	
Wfdc21	23.657	41.417	0.76	0.13	
Pira2	6.228	10.685	0.77	0.13	
BC028528	1.577	2.581	0.74	0.13	
Atp6v1c2	0.032	0.088	1.02	0.13	
Il2rg	1.865	3.129	0.74	0.13	
Slfn5	2.211	3.666	0.74	0.13	
Pparg	0.858	1.411	0.77	0.13	
Olfr823	0.000	0.037	1.38	0.13	
Slc6a4	1.153	1.853	0.78	0.13	
Il20rb	0.281	0.413	0.75	0.13	
3110009F21Rik	0.080	0.134	0.88	0.13	
Micalcl	0.054	0.106	0.88	0.12	

Tmem173	1.379	2.605	0.82	0.12	
Ccr4	0.000	0.008	1.30	0.12	
Lat2	0.478	0.792	0.80	0.12	
Il18r1	0.139	0.219	0.74	0.12	+
Themis2	2.119	3.332	0.73	0.12	
1700124L16Rik	0.026	0.102	1.29	0.12	
Krt35	0.002	0.017	1.18	0.12	
Spa17	0.570	0.913	0.80	0.12	
Aldh3b2	0.314	0.493	0.76	0.12	
Ifitm6	38.815	67.477	0.76	0.12	
Sell	9.341	16.583	0.74	0.12	+
1700016H13Rik	0.006	0.044	1.26	0.12	
Cplx1	0.015	0.032	1.04	0.12	
Ccdc87	0.002	0.007	1.32	0.12	
Alox5ap	15.340	26.313	0.76	0.12	
Gm20743	0.091	0.163	0.86	0.12	
Slco5a1	0.007	0.016	0.98	0.12	
Khdc3	0.048	0.094	0.86	0.12	
Ddx3y	1.836	3.400	0.76	0.12	
Gpx3	7.178	12.808	0.78	0.12	
Cabp4	0.074	0.152	0.85	0.12	
Mir692-2	18.657	30.780	0.72	0.11	
Flrt2	0.351	0.591	0.82	0.11	
Lyz2	285.959	500.890	0.76	0.11	
Mir703	95.701	190.080	0.71	0.11	
Chst3	1.050	1.594	0.69	0.11	
C4bp	5.869	10.678	0.75	0.11	
Mybpc2	0.048	0.081	0.81	0.11	
Ralgds	0.732	1.218	0.81	0.11	
Cd79a	8.426	13.568	0.71	0.11	
Ecscr	2.313	3.889	0.78	0.11	
AB124611	7.067	12.231	0.75	0.11	
H2-DMb1	1.631	2.526	0.69	0.11	
Fpr3	0.203	0.338	0.80	0.11	

Slc6a11	0.047	0.094	0.90	0.11
Mro	0.001	0.013	1.32	0.11
Pira1	6.258	10.804	0.75	0.11
6530402F18Rik	0.286	0.494	0.78	0.11
Sfxn4	0.070	0.116	0.87	0.11
Mir3077	0.993	2.483	0.92	0.11
Batf3	0.446	0.736	0.74	0.11
2310075C17Rik	0.074	0.210	1.03	0.11
Tyrobp	42.971	72.722	0.75	0.10
Gm14548	8.338	14.113	0.71	0.10
1700073E17Rik	0.012	0.028	0.91	0.10
Scn4a	0.022	0.043	0.92	0.10
S1pr1	3.787	6.321	0.73	0.10
Endou	0.195	0.338	0.75	0.10
Chil1	6.429	10.343	0.75	0.10
AF357399	0.515	1.127	0.93	0.10
Sp110	8.446	13.974	0.72	0.10
H2-L	191.027	331.801	0.75	0.10
1110046J04Rik	0.039	0.114	1.07	0.10
Gbp2b	0.091	0.161	0.89	0.10
Tekt4	0.142	0.231	0.76	0.10
Trim72	0.011	0.031	1.03	0.10
Gm14327	0.456	0.732	0.73	0.10
Slc47a2	0.015	0.035	1.06	0.10
Zfp521	0.176	0.290	0.81	0.10
Gp6	1.177	1.824	0.76	0.10
Treml4	1.682	2.823	0.75	0.10
Ugt3a2	3.910	5.806	0.61	0.10
Zfp105	0.563	0.890	0.75	0.09
Samd3	0.003	0.013	1.30	0.09
Trh	0.009	0.034	1.13	0.09
Klra14-ps	0.019	0.066	1.23	0.09

Plekhf1	0.590	0.906	0.74	0.09	
Strip2	0.046	0.081	0.81	0.09	
Clec4n	5.090	8.850	0.78	0.09	
Slpi	4.529	7.720	0.75	0.09	
Ofcc1	0.000	0.010	1.50	0.09	
Xcl1	0.102	0.235	0.92	0.09	
Lsp1	6.114	9.839	0.67	0.09	
Mmp13	1.481	2.354	0.66	0.09	
Apobec2	0.119	0.237	0.86	0.09	
4833422M21Rik	0.000	0.017	1.24	0.09	
Gm15446	0.211	0.323	0.75	0.09	
Cds1	0.174	0.288	0.77	0.09	
Pik3r5	0.597	0.983	0.71	0.08	+
Mir5116	1.105	2.336	0.99	0.08	
Cxcl2	0.341	0.533	0.88	0.08	
Nfkbie	0.663	1.053	0.73	0.08	
Gpr171	0.437	0.672	0.72	0.08	
AI839979	0.591	0.973	0.73	0.08	
Dok2	3.929	6.287	0.71	0.08	
Lcn2	126.126	206.528	0.72	0.08	
Pira11	8.706	14.946	0.69	0.08	
AA467197	1.948	3.145	0.75	0.08	
Lyz1	30.906	52.751	0.75	0.08	
Myh7	0.023	0.040	0.89	0.08	
Kcng2	0.118	0.217	0.79	0.08	
4933433H22Rik	0.000	0.013	1.30	0.08	
Aplp1	0.596	0.906	0.73	0.08	
Slfnl1	0.002	0.013	1.16	0.08	
S100a8	1329.520	#######	0.72	0.08	
Bcas3os1	0.004	0.017	0.98	0.08	
Sit1	0.169	0.270	0.77	0.08	
Tnfsf14	0.787	1.389	0.84	0.08	
Rn4.5s	0.936	1.346	0.70	0.08	

Trem3	4.337	7.094	0.69	0.07	
Gbp3	1.798	2.745	0.65	0.07	
Ebi3	3.641	5.600	0.69	0.07	+
Rac3	0.775	1.256	0.73	0.07	
Thnsl1	0.655	1.075	0.72	0.07	
Pira6	5.546	9.378	0.67	0.07	
Gzmb	2.082	3.309	0.69	0.07	
Cyth4	2.900	4.526	0.68	0.07	
Mybphl	1.027	1.522	0.69	0.07	
Gm6634	0.072	0.134	0.84	0.07	
Gapt	1.021	1.627	0.70	0.07	
Snn	3.069	4.921	0.73	0.07	
Mzb1	3.484	5.535	0.68	0.07	
5730416F02Rik	1.420	2.262	0.70	0.07	
Slc22a29	0.032	0.064	0.91	0.07	
Il13ra1	3.556	5.761	0.68	0.07	
1810043G02Rik	0.828	1.371	0.80	0.07	
Rdh12	0.900	1.445	0.73	0.07	
Gm16894	0.018	0.069	1.15	0.07	
Mest	33.066	53.139	0.66	0.07	
Wdr93	0.033	0.067	0.89	0.07	
Aspg	3.901	5.968	0.71	0.07	
Slamf6	0.705	1.120	0.72	0.07	
Lrrc27	0.109	0.182	0.73	0.07	
2310043L19Rik	0.000	0.034	1.11	0.07	
Klf4	0.935	1.383	0.79	0.07	
Gpr137c	0.050	0.087	0.80	0.06	
BC100530	1872.910	#######	0.66	0.06	
Orm2	0.665	1.057	0.73	0.06	
Higd1c	0.042	0.105	1.04	0.06	
Dnah6	0.012	0.021	0.91	0.06	
Apcdd1	0.199	0.347	0.79	0.06	

Tnfrsf13b	0.460	0.824	0.75	0.06
Snora20	0.744	1.737	0.90	0.06
Mir6981	1.447	2.406	0.79	0.06
Ltb	1.581	2.564	0.74	0.06
AF251705	6.408	10.361	0.71	0.06
Steap4	6.526	10.936	0.69	0.06
F730035M05Rik	0.037	0.073	0.85	0.06
Zfp85	0.561	0.865	0.67	0.06
Mmp12	0.018	0.038	0.89	0.06
Actl6b	0.016	0.046	1.04	0.06
Camk2a	0.143	0.228	0.70	0.06
Fndc8	0.097	0.164	0.76	0.06
Inhbb	1.131	1.757	0.71	0.06
Sftpb	0.023	0.053	1.03	0.06
Naaladl1	0.416	0.629	0.68	0.06
Tdgf1	0.000	0.006	1.36	0.06
Cd300a	2.031	3.252	0.68	0.05
Tdrp	0.222	0.387	0.72	0.05
Olfr1265	0.029	0.082	0.97	0.05
Blnk	0.977	1.530	0.76	0.05
Rhoh	0.740	1.155	0.70	0.05
Treml1	5.747	8.622	0.66	0.05
Ly6c1	14.491	25.519	0.74	0.05
Lpcat2	1.201	1.926	0.75	0.05
Comp	1.160	1.866	0.70	0.05
P2ry10	0.093	0.171	0.78	0.05
Bmx	0.479	0.746	0.66	0.05
Mir692-3	18.657	30.780	0.74	0.05
Sfxn5	0.677	1.086	0.69	0.05
Fkbp1b	1.587	2.426	0.69	0.05
Dgkh	0.124	0.207	0.70	0.05
H2-M5	2.442	3.919	0.67	0.05
Gm17745	0.138	0.255	0.85	0.05
Gpat2	0.199	0.320	0.69	0.05
H2-Aa	3.507	5.368	0.67	0.05

Kcne3	0.352	0.555	0.75	0.04
Smim3	3.858	5.957	0.67	0.04
Kng2	448.586	703.959	0.64	0.04
Tmsb4x	524.846	843.350	0.66	0.04
Tnnt3	0.076	0.142	0.81	0.04
Gm5136	0.368	0.595	0.72	0.04
Gm13023	0.000	0.013	1.42	0.04
Ggct	1.069	1.685	0.70	0.04
Ovol1	0.061	0.110	0.80	0.04
Faiml	0.146	0.247	0.73	0.04
Fcer1g	29.697	47.410	0.65	0.04
Gm15987	0.201	0.332	0.76	0.04
Atp2c2	0.022	0.042	0.85	0.04
Pilra	2.471	3.966	0.67	0.04
Slc11a1	3.558	5.743	0.68	0.04
Adam1a	0.127	0.198	0.68	0.04
Cage1	0.102	0.152	0.70	0.04
Cd79b	7.571	11.777	0.65	0.04
Tmem184a	0.676	1.053	0.69	0.04
Grin3a	0.008	0.015	0.88	0.04
Mt1	395.915	711.609	0.61	0.04
Cd72	2.660	4.241	0.73	0.04
Ticam2	0.368	0.518	0.62	0.04
Ifnz	0.093	0.157	0.83	0.03
Tmprss3	0.710	1.094	0.69	0.03
Gm13308	0.859	1.157	0.67	0.03
1810062O18Rik	0.226	0.353	0.75	0.03
Klhl36	1.269	1.973	0.68	0.03
Slc7a8	2.272	3.467	0.61	0.03
Traf1	0.043	0.078	0.75	0.03
Nr4a1	2.639	3.380	0.68	0.03
Gm3696	0.016	0.033	0.86	0.03
Uxt	1.620	2.438	0.68	0.03
C030013G03Rik	0.018	0.058	1.00	0.03

Mir6961	9.033	14.056	0.70	0.03	
Hspb11	2.041	3.138	0.68	0.03	
Cxcl9	0.400	0.675	0.71	0.03	+
Zfp641	0.079	0.117	0.66	0.03	
Tap1	3.374	5.323	0.66	0.03	
Stk17b	7.808	11.928	0.62	0.03	
Fam26f	0.515	0.789	0.67	0.03	
B630019K06Rik	0.120	0.192	0.70	0.03	
Map3k14	0.308	0.474	0.71	0.03	
F2rl3	1.249	1.904	0.63	0.03	
Bcl3	1.956	3.002	0.64	0.03	
Tstd1	5.284	8.076	0.65	0.03	
Ly6c2	61.004	98.306	0.68	0.03	
B4galt4	1.036	1.567	0.69	0.03	
5033406O09Rik	0.614	0.936	0.67	0.03	
Crybb3	0.106	0.220	0.84	0.03	
Gm16287	0.007	0.028	1.09	0.03	
Park2	0.061	0.101	0.75	0.03	
C1qtnf1	3.205	5.056	0.65	0.03	
DXBay18	1.029	1.545	0.63	0.03	
Ryr1	0.027	0.040	0.65	0.03	
Pycard	1.916	3.024	0.71	0.03	
Cysltr1	0.239	0.387	0.71	0.03	
Ttc39b	0.390	0.606	0.64	0.03	
Hmgcll1	0.101	0.158	0.69	0.03	
Robo3	0.055	0.082	0.69	0.03	
Pde6b	0.045	0.075	0.78	0.03	
Ntpcr	2.455	3.720	0.66	0.03	
Cd53	6.781	10.531	0.67	0.03	
Defb30	0.000	0.023	1.37	0.02	
Apol10a	0.146	0.237	0.70	0.02	
Nek5	0.038	0.069	0.84	0.02	
Mgat5	0.420	0.599	0.63	0.02	
Gadd45g	12.312	17.151	0.65	0.02	

Pydc3	0.990	1.537	0.68	0.02
Gm19705	1.510	2.323	0.64	0.02
Samhd1	6.098	9.512	0.65	0.02
Ace	0.092	0.143	0.69	0.02
Ganc	0.991	1.459	0.62	0.02
4930486L24Rik	0.095	0.167	0.74	0.02
Gm5477	0.650	0.903	0.71	0.02
Serpina3b	0.709	1.018	0.67	0.02
Lat	2.308	3.534	0.65	0.02
Aldh3b3	0.536	0.813	0.59	0.02
Siglece	2.213	3.448	0.66	0.02
Foxq1	10.180	13.415	0.72	0.02
Trpc7	0.002	0.010	1.14	0.02
H2-Q5	10.854	16.233	0.59	0.02
Pla2g15	2.291	3.341	0.60	0.02
Cmtm2a	0.246	0.380	0.71	0.02
Dgat1	4.337	6.154	0.61	0.01
Ciita	0.042	0.072	0.78	0.01
Cd300lb	1.008	1.519	0.62	0.01
Tsga13	0.013	0.046	1.03	0.01
Slamf7	0.871	1.258	0.62	0.01
Ifit3	7.923	11.651	0.64	0.01
Pydc4	0.600	0.969	0.68	0.01
Creld2	4.976	7.742	0.68	0.01
Ush1g	0.071	0.098	0.68	0.01
Nupr1	3.075	4.523	0.65	0.01
Mlkl	1.668	2.414	0.63	0.01
Nod1	1.143	1.682	0.62	0.01
Cib2	0.625	0.952	0.71	0.01
Gm6642	5.879	8.579	0.66	0.01
Pcbp4	1.575	2.322	0.63	0.01
Cacna1b	0.248	0.402	0.72	0.01
Tgfbi	6.591	10.449	0.61	0.01
Rtn4rl2	0.144	0.219	0.69	0.01
Pde10a	0.236	0.372	0.71	0.01

Lcp1	21.165	31.185	0.58	0.01	
Rufy4	0.087	0.141	0.73	0.00	
Mir7041	1.688	3.283	0.82	0.00	
Cd22	0.833	1.189	0.62	0.00	
Cd5l	36.267	55.442	0.62	0.00	
Apol11b	1.165	1.697	0.61	0.00	
Gfi1	1.143	1.582	0.56	0.00	
Pafah1b3	4.769	7.374	0.63	0.00	
Copg2	12.928	19.327	0.59	0.00	
Olfr56	0.364	0.543	0.65	0.00	
Ifng	0.025	0.063	0.94	0.00	
Gh	0.000	0.039	1.50	0.00	
S100a11	64.782	97.271	0.60	0.00	
Pde1b	0.469	0.689	0.59	0.00	
Upp1	0.460	0.676	0.66	0.00	
Slc3a1	8.926	5.799	-0.59	0.00	
Sall4	0.565	0.343	-0.69	0.00	
Adm2	2.959	1.848	-0.65	0.00	
Sdpr	10.043	6.661	-0.63	0.00	
D5Ertd615e	0.012	0.000	-1.35	0.00	
Gli2	0.109	0.062	-0.70	0.00	
Ddn	0.011	0.000	-1.43	0.00	
P2ry4	0.194	0.110	-0.69	0.00	
Hsd17b14	0.155	0.078	-0.80	0.00	
Tmem252	0.519	0.253	-0.59	0.00	
Adgrv1	0.024	0.013	-0.73	0.00	
Lrrc63	0.017	0.002	-1.07	0.00	
Wfdc6a	0.056	0.000	-1.32	0.00	
Gm16596	0.281	0.167	-0.67	0.00	
Tnfsf15	0.009	0.002	-1.00	0.00	+
Sowaha	4.791	3.229	-0.62	-0.01	
6030468B19Rik	8.062	4.827	-0.65	-0.01	
Pcdha3	0.168	0.100	-0.72	-0.01	
Lrfn4	0.922	0.524	-0.68	-0.01	
Srsf2	77.608	37.685	-0.70	-0.01	

Tecta	0.008	0.003	-0.98	-0.01
Fam47e	2.308	1.392	-0.62	-0.01
Pcdha4	0.166	0.098	-0.75	-0.01
Ehhadh	33.514	20.658	-0.73	-0.01
Olfr10	0.162	0.082	-0.85	-0.01
Aass	7.411	4.366	-0.60	-0.01
Gm15401	0.483	0.258	-0.69	-0.01
Mn1	0.312	0.195	-0.60	-0.01
Enox1	0.023	0.007	-1.08	-0.01
Atg9b	0.688	0.403	-0.69	-0.01
Xrcc2	0.794	0.485	-0.63	-0.01
Itga2	0.661	0.359	-0.63	-0.01
Lincred1	1.467	0.868	-0.66	-0.01
Cldn13	28.698	18.038	-0.67	-0.01
Hipk2	3.994	2.506	-0.67	-0.01
Hspb2	0.209	0.091	-0.89	-0.01
Krt10	0.286	0.169	-0.72	-0.01
Impg1	0.013	0.002	-1.13	-0.01
Slco4c1	0.900	0.425	-0.66	-0.02
Mup4	0.062	0.000	-1.40	-0.02
Pdgfc	0.513	0.307	-0.65	-0.02
A630019I02Rik	0.012	0.000	-1.32	-0.02
Uprt	0.414	0.249	-0.63	-0.02
Ppp2r3a	1.630	1.012	-0.64	-0.02
Ppp2r2a	8.250	3.655	-0.63	-0.02
E230025N22Rik	0.018	0.000	-1.30	-0.02
Clec11a	0.430	0.238	-0.68	-0.02
Cep128	1.274	0.835	-0.62	-0.02
A930018P22Rik	0.115	0.029	-1.21	-0.02
Fhl5	0.030	0.000	-1.24	-0.02
Gpam	17.871	12.009	-0.60	-0.02
Cps1	193.826	123.398	-0.65	-0.02
Cdh11	1.091	0.616	-0.67	-0.02
Paqr6	0.237	0.127	-0.75	-0.02

Slc5a9	0.026	0.011	-0.99	-0.02	
Rab42	0.107	0.040	-0.95	-0.02	
Ankrd37	7.321	3.028	-0.64	-0.02	
Ntsr1	0.012	0.003	-1.07	-0.02	
Trib3	2.376	1.452	-0.64	-0.02	
Slc16a4	0.130	0.067	-0.85	-0.03	
Sh3pxd2a	1.958	1.263	-0.68	-0.03	
Inhba	2.068	1.255	-0.64	-0.03	+
Hecw2	0.300	0.183	-0.67	-0.03	
Hemgn	50.539	33.079	-0.64	-0.03	
Sptbn4	0.082	0.047	-0.73	-0.03	
Tspan33	24.193	15.723	-0.60	-0.03	
Bicc1	1.516	0.910	-0.58	-0.03	
Gnmt	78.652	51.457	-0.64	-0.03	
Cyp4a29	2.241	1.300	-0.67	-0.03	
Slc4a3	0.226	0.119	-0.77	-0.03	
Gpr153	0.635	0.360	-0.66	-0.03	
Tinag	0.078	0.034	-0.89	-0.03	
Cyp27b1	0.020	0.002	-1.36	-0.03	
Obscn	0.096	0.057	-0.75	-0.03	
Ndrg1	53.469	33.220	-0.71	-0.03	
Dusp18	0.086	0.043	-0.76	-0.03	
Prr22	0.139	0.070	-0.82	-0.03	
Mall	0.035	0.012	-1.04	-0.03	
Acot1	13.923	7.567	-0.67	-0.03	
Spire1	5.568	3.570	-0.67	-0.03	
9930014A18Rik	0.634	0.384	-0.68	-0.03	
Gm14405	0.356	0.201	-0.74	-0.03	
Pdk4	12.253	6.362	-0.66	-0.03	
Gfra3	0.014	0.000	-1.40	-0.03	
Gm7444	0.377	0.232	-0.71	-0.03	
Mir6995	1.108	0.249	-1.15	-0.03	
Srpx	0.294	0.153	-0.70	-0.04	
Adam22	0.049	0.027	-0.71	-0.04	
Apoa5	90.116	61.574	-0.59	-0.04	

B4galnt2	0.135	0.075	-0.72	-0.04
Klhl35	0.045	0.015	-0.95	-0.04
Foxo3	7.675	4.633	-0.66	-0.04
Onecut1	3.493	2.005	-0.64	-0.04
Tns1	3.970	2.431	-0.70	-0.04
Unc5cl	0.325	0.191	-0.71	-0.04
Ltbp3	0.685	0.379	-0.69	-0.04
Il1rl2	0.490	0.272	-0.76	-0.04
9530052E02Rik	1.269	0.728	-0.77	-0.04
Dcc	0.004	0.000	-1.28	-0.04
B130024G19Rik	0.259	0.140	-0.68	-0.04
Egr1	11.883	4.656	-0.66	-0.04
Prn	0.336	0.173	-0.72	-0.04
Zbed6	2.933	1.944	-0.66	-0.04
Mug2	6.439	4.071	-0.67	-0.04
A730056A06Rik	0.010	0.000	-1.32	-0.04
Scrn1	0.068	0.023	-0.92	-0.04
2310007B03Rik	0.345	0.189	-0.73	-0.04
Dkk3	0.819	0.440	-0.69	-0.04
Hif3a	6.548	4.243	-0.72	-0.04
3632451O06Rik	0.160	0.086	-0.76	-0.04
Snord2	1.424	0.433	-0.99	-0.04
Sctr	0.025	0.000	-1.32	-0.04
Sh2d4a	2.858	1.736	-0.65	-0.04
Grem1	0.028	0.000	-1.41	-0.04
Epcam	1.523	0.888	-0.70	-0.04
Fap	0.062	0.030	-0.82	-0.04
St8sia1	0.024	0.014	-0.78	-0.04
Serpina4-ps1	6.287	4.215	-0.66	-0.05
Ryr3	0.024	0.015	-0.85	-0.05
Radil	0.021	0.005	-1.12	-0.05

Podxl2	0.281	0.138	-0.83	-0.05
Fam205a1	0.188	0.104	-0.74	-0.05
Klhl11	0.565	0.326	-0.73	-0.05
Ehd3	15.517	10.147	-0.63	-0.05
Yod1	2.088	1.295	-0.66	-0.05
Cpox	76.390	50.334	-0.68	-0.05
Znrf3	1.154	0.733	-0.67	-0.05
Necab1	1.094	0.694	-0.66	-0.05
Col24a1	0.112	0.066	-0.74	-0.05
Plin5	8.601	5.358	-0.67	-0.05
Spta1	10.182	6.027	-0.69	-0.05
Map1a	0.079	0.042	-0.73	-0.05
Gm19461	0.041	0.019	-0.86	-0.05
Btn1a1	0.325	0.181	-0.77	-0.05
Glb1l2	0.012	0.000	-1.33	-0.05
Gm24572	0.997	0.401	-0.92	-0.06
Gpihbp1	7.867	4.741	-0.70	-0.06
Tfdp2	7.706	4.957	-0.66	-0.06
Pcdha11	0.167	0.100	-0.80	-0.06
Cacna2d3	0.009	0.000	-1.23	-0.06
Ankrd55	0.693	0.408	-0.72	-0.06
Olfr1094	0.034	0.000	-1.21	-0.06
Col4a4	0.110	0.056	-0.74	-0.06
Plch1	0.034	0.015	-0.89	-0.06
Tox2	0.125	0.056	-0.91	-0.06
Rhbg	1.122	0.686	-0.71	-0.06
Wnt7a	0.013	0.000	-1.47	-0.06
Pcdha5	0.169	0.101	-0.79	-0.06
Pcdha7	0.168	0.100	-0.73	-0.06
Fam151a	0.684	0.431	-0.65	-0.06
Lrrd1	0.017	0.004	-1.34	-0.06
Chd9	1.773	0.842	-0.68	-0.06
Nrp1	2.462	1.535	-0.67	-0.06
A930006K02Rik	2.135	1.241	-0.76	-0.07
Cyp39a1	23.831	14.512	-0.72	-0.07

Ephb3	0.156	0.078	-0.78	-0.07
Rnf151	0.036	0.000	-1.33	-0.07
Gm1564	0.010	0.001	-1.22	-0.07
Slc17a8	1.912	1.145	-0.70	-0.07
Acacb	2.817	1.647	-0.76	-0.07
Spink4	0.123	0.000	-1.45	-0.07
Cyp3a11	172.472	102.545	-0.72	-0.07
Ctse	13.979	8.215	-0.69	-0.07
Ogn	0.875	0.452	-0.76	-0.07
Ctgf	5.784	3.230	-0.67	-0.07
Mcmdc2	0.115	0.060	-0.84	-0.07
Fam84b	2.506	1.546	-0.67	-0.07
Scn9a	0.006	0.002	-0.99	-0.07
Plekhs1	0.037	0.012	-1.04	-0.07
Lrrc3b	0.075	0.025	-1.00	-0.07
Gm14207	0.346	0.174	-0.83	-0.07
Cd163	0.453	0.250	-0.71	-0.07
Prss50	0.901	0.526	-0.75	-0.07
Pak3	0.009	0.003	-1.06	-0.07
Ggt1	11.740	7.286	-0.68	-0.07
Akr1b7	204.922	130.997	-0.71	-0.08
Npc1l1	0.012	0.003	-1.11	-0.08
Ell2	16.443	10.267	-0.61	-0.08
Acot2	6.343	3.463	-0.69	-0.08
Tnfrsf14	12.674	7.672	-0.69	-0.08
Lox	2.383	1.303	-0.74	-0.08
Nnat	2.027	1.088	-0.68	-0.08
Rab3b	0.200	0.101	-0.84	-0.08
Col4a5	0.552	0.326	-0.70	-0.08
Thy1	0.572	0.278	-0.75	-0.08
Gprc5b	0.412	0.226	-0.75	-0.08
Gpr162	0.103	0.046	-0.89	-0.08
Mup3	0.077	0.013	-1.24	-0.08
2610027K06Rik	0.327	0.182	-0.78	-0.08
Gen1	1.302	0.839	-0.67	-0.08

Lect1	0.303	0.148	-0.84	-0.08
4930513N10Rik	0.249	0.141	-0.78	-0.08
Rnase2a	0.044	0.000	-1.46	-0.08
Brca2	1.057	0.649	-0.71	-0.08
Mir6918	0.574	0.000	-1.20	-0.08
Mier1	11.061	5.028	-0.74	-0.08
Elavl3	0.007	0.000	-1.41	-0.08
Aifm3	2.303	1.446	-0.66	-0.08
Gm19345	0.076	0.018	-1.17	-0.09
Insm2	0.009	0.000	-1.19	-0.09
Clvs2	0.011	0.000	-1.49	-0.09
Lhpp	4.446	2.557	-0.72	-0.09
Klf11	3.519	2.106	-0.72	-0.09
5330439B14Rik	0.670	0.364	-0.79	-0.09
Ildr2	0.113	0.060	-0.79	-0.09
Cntfr	0.276	0.143	-0.78	-0.09
Snord38a	0.872	0.100	-1.18	-0.09
Erich5	0.236	0.116	-0.85	-0.09
Dsc3	0.011	0.002	-1.26	-0.09
Nes	1.550	0.879	-0.80	-0.09
Kctd15	0.277	0.142	-0.78	-0.09
Cep85l	2.669	1.387	-0.75	-0.09
Cyp4a12a	8.104	4.727	-0.77	-0.09
4921511C10Rik	0.033	0.000	-1.30	-0.09
Ebf3	0.015	0.002	-1.24	-0.09
Rnf123	9.467	5.887	-0.71	-0.10
Spaca6	0.486	0.267	-0.81	-0.10
Zfp174	0.283	0.163	-0.73	-0.10
Atad5	2.091	1.234	-0.72	-0.10
Pdpr	0.875	0.539	-0.70	-0.10
Maml2	0.577	0.317	-0.75	-0.10
Sox8	0.019	0.004	-1.11	-0.10
Pcdha2	0.164	0.098	-0.81	-0.10

Igfbp5	2.811	1.492	-0.75	-0.10
Ltbp4	4.621	2.611	-0.76	-0.10
Gm9895	2.425	1.401	-0.74	-0.10
Ube2u	0.824	0.469	-0.75	-0.10
Arhgef10	0.413	0.230	-0.75	-0.10
Dll4	1.013	0.582	-0.73	-0.10
Sftpc	0.049	0.000	-1.17	-0.10
Megf6	0.662	0.351	-0.72	-0.10
Ccnb3	0.005	0.000	-1.41	-0.10
Stx5a	20.772	8.301	-0.75	-0.10
D630033O11Rik	0.200	0.072	-1.07	-0.10
Igsf9	0.211	0.109	-0.78	-0.10
Arhgap32	0.499	0.305	-0.68	-0.11
Gdf5	0.017	0.000	-1.47	-0.11
Lvrn	0.034	0.009	-1.18	-0.11
Col17a1	0.035	0.017	-0.87	-0.11
Slc7a3	0.068	0.029	-0.88	-0.11
Ptpn14	0.358	0.199	-0.76	-0.11
Pcdha6	0.167	0.100	-0.76	-0.11
Mns1	10.005	5.795	-0.71	-0.11
Pkhd1l1	0.918	0.540	-0.73	-0.11
Hapln3	0.025	0.000	-1.53	-0.11
Sox6	3.259	1.971	-0.66	-0.11
Ltb4r2	0.345	0.184	-0.86	-0.11
Lct	0.034	0.016	-0.85	-0.11
Rgs7bp	0.212	0.124	-0.72	-0.11
Pcdha1	0.167	0.100	-0.73	-0.11
Col6a3	2.024	1.102	-0.73	-0.11
Bmp2	2.183	1.137	-0.78	-0.11
Bmp8b	0.077	0.038	-0.94	-0.11
4930558J18Rik	1.125	0.648	-0.75	-0.11
Rhd	36.563	20.671	-0.76	-0.11
Foxj1	0.166	0.078	-0.84	-0.11
Lect2	13.313	7.946	-0.80	-0.11

Sdk2	0.062	0.025	-0.93	-0.11	
Frem1	0.275	0.143	-0.83	-0.11	
5830473C10Rik	6.698	3.278	-0.74	-0.11	
Adamts17	0.064	0.033	-0.83	-0.11	
Pla2g5	0.015	0.000	-1.28	-0.12	
Efnb1	3.039	1.698	-0.78	-0.12	
Fgf12	0.015	0.006	-0.98	-0.12	
Mir877	4.664	2.285	-0.83	-0.12	
4933407K13Rik	0.965	0.534	-0.77	-0.12	
Pianp	0.053	0.015	-1.08	-0.12	
Syngap1	0.427	0.249	-0.76	-0.12	
Scube3	0.063	0.026	-0.95	-0.12	
2610305D13Rik	0.552	0.322	-0.77	-0.12	
Pcdhac2	0.165	0.098	-0.82	-0.12	
Cth	31.988	19.224	-0.71	-0.12	
C030034I22Rik	0.280	0.137	-0.82	-0.12	
Pcdhac1	0.166	0.099	-0.79	-0.12	
Vipr2	0.099	0.049	-0.89	-0.12	
Wscd2	0.012	0.000	-1.60	-0.12	
Slc25a21	2.108	1.258	-0.74	-0.12	
Glod5	0.766	0.359	-0.90	-0.12	
Syt4	0.046	0.018	-1.02	-0.12	
Mmp2	3.242	1.707	-0.76	-0.12	
Asb13	14.877	3.353	-0.77	-0.12	
Syt15	0.123	0.063	-0.85	-0.12	
Cpeb3	0.725	0.428	-0.73	-0.13	
Hoxa2	0.239	0.126	-0.86	-0.13	
Ascl1	0.019	0.000	-1.50	-0.13	
Hmbox1	1.323	0.737	-0.78	-0.13	
Sphk1	2.349	1.387	-0.72	-0.13	+
Mir6934	0.927	0.095	-1.30	-0.13	

9030625G05Rik	0.133	0.034	-1.21	-0.13
Tet1	0.106	0.055	-0.82	-0.13
Gm609	0.140	0.067	-0.94	-0.13
Pcca	6.710	3.964	-0.78	-0.13
Snord53	0.534	0.000	-1.40	-0.13
Scx	0.136	0.047	-1.11	-0.13
Ass1	137.341	79.391	-0.76	-0.13
4930519D14Rik	0.162	0.088	-0.90	-0.13
Rasl12	0.145	0.059	-0.88	-0.13
Medag	0.269	0.156	-0.82	-0.13
4833415N18Rik	0.024	0.000	-1.23	-0.13
Myl1	0.095	0.037	-0.99	-0.13
Hsd3b6	10.676	6.347	-0.73	-0.13
Gpr156	0.014	0.003	-1.34	-0.13
Fastk	27.815	9.811	-0.75	-0.13
Epha4	0.148	0.079	-0.77	-0.13
Cntn3	0.222	0.115	-0.87	-0.13
Zfp872	0.087	0.044	-0.88	-0.13
Atp2b4	8.730	4.999	-0.79	-0.13
Abi3bp	0.089	0.045	-0.79	-0.13
Fibin	0.712	0.245	-0.83	-0.14
Slc25a41	0.017	0.000	-1.47	-0.14
Slc26a9	0.010	0.000	-1.33	-0.14
Adarb1	0.767	0.409	-0.79	-0.14
Mageb16	0.040	0.012	-1.07	-0.14
2210416O15Rik	0.038	0.008	-1.37	-0.14
Npnt	2.728	1.512	-0.78	-0.14
Col8a2	0.344	0.171	-0.73	-0.14
Ppap2b	12.628	7.649	-0.70	-0.14
Pah	39.349	21.229	-0.79	-0.14
Nol3	0.201	0.103	-0.83	-0.14
Vmn1r14	0.040	0.000	-1.40	-0.14

Rab15	0.082	0.030	-0.96	-0.14	
Bmper	0.394	0.230	-0.67	-0.14	
D830031N03Rik	0.193	0.112	-0.72	-0.14	
Phf24	0.021	0.005	-1.27	-0.14	
Aim1l	0.133	0.073	-0.82	-0.14	
Ammecr1	2.213	1.316	-0.74	-0.14	
Etnk2	15.500	9.512	-0.74	-0.14	
Cldn18	0.014	0.000	-1.56	-0.15	
Gpt	1.058	0.612	-0.83	-0.15	
Ppp1r1b	0.598	0.325	-0.82	-0.15	
Slco1b2	36.207	21.055	-0.81	-0.15	
Plau	1.100	0.638	-0.77	-0.15	
Col12a1	0.490	0.276	-0.80	-0.15	
Cacna1g	0.072	0.031	-0.88	-0.15	
Sez6l2	0.072	0.031	-0.96	-0.15	
Rgs16	39.004	20.297	-0.79	-0.15	+
2310068J16Rik	1.374	0.772	-0.83	-0.15	
Dnah10	0.018	0.008	-0.96	-0.15	
Pdgfd	0.270	0.121	-0.98	-0.15	
Myzap	0.901	0.490	-0.84	-0.15	
Efnb3	0.173	0.086	-0.83	-0.15	
Klhl38	0.100	0.051	-0.89	-0.15	
Aqp3	0.963	0.514	-0.82	-0.16	
Ntf3	0.109	0.042	-1.03	-0.16	
Glrp1	0.148	0.066	-0.88	-0.16	
Mapt	0.045	0.020	-0.92	-0.16	
Cd164l2	0.206	0.090	-0.94	-0.16	
Rcor1	5.984	2.835	-0.81	-0.16	
Dclk2	0.721	0.397	-0.79	-0.16	
Cela1	5.992	3.230	-0.82	-0.16	
Mab21l2	0.037	0.004	-1.24	-0.16	
1700109K24Rik	0.175	0.065	-1.19	-0.16	
Ppp1r3c	14.788	6.357	-0.74	-0.16	

G0s2	19.500	8.777	-0.80	-0.16
Slc18b1	0.399	0.205	-0.86	-0.16
Csrnp3	0.006	0.001	-1.22	-0.16
Sap30l	8.399	2.500	-0.74	-0.16
Taf7l	0.014	0.000	-1.34	-0.16
Adamtsl1	0.051	0.023	-0.93	-0.17
Gria1	0.029	0.011	-1.00	-0.17
Pcdha8	0.167	0.102	-0.82	-0.17
Gm5424	414.461	241.854	-0.76	-0.17
Mrc2	0.430	0.209	-0.80	-0.17
Dpy19l2	0.013	0.000	-1.47	-0.17
Cyp3a41a	126.794	73.826	-0.80	-0.17
Lama3	0.070	0.034	-0.83	-0.17
Itgb4	0.134	0.063	-0.90	-0.17
Gm4759	0.080	0.038	-0.93	-0.17
Grid1	0.041	0.015	-1.04	-0.17
Ryr2	0.025	0.012	-0.85	-0.17
Gm4489	0.145	0.034	-1.25	-0.17
Scnn1b	0.022	0.004	-1.19	-0.17
Apol7e	0.813	0.399	-0.83	-0.17
Vmn2r57	0.009	0.000	-1.23	-0.17
Fbln1	0.704	0.305	-0.87	-0.17
Pcdh18	0.319	0.178	-0.83	-0.18
Acsbg1	0.035	0.011	-1.15	-0.18
Smtnl2	1.103	0.594	-0.83	-0.18
Fasn	14.154	8.198	-0.77	-0.18
Sec14l4	7.571	4.045	-0.82	-0.18
N4bp2l1	5.767	3.317	-0.83	-0.18
Apol7b	0.813	0.399	-0.87	-0.18
Asic5	0.124	0.053	-0.97	-0.18
Lama2	0.311	0.144	-0.86	-0.18
Hymai	0.186	0.090	-0.88	-0.18
Pcdhb15	0.022	0.004	-1.32	-0.18
Bloodlinc	0.362	0.139	-1.01	-0.18
Cldn10	0.130	0.061	-0.98	-0.18
Olfr420	0.211	0.090	-1.01	-0.18

Mia	0.155	0.031	-1.31	-0.18	
Kcnmb2	0.037	0.010	-1.23	-0.19	+
AI506816	0.347	0.142	-0.95	-0.19	
Sptb	14.742	8.958	-0.74	-0.19	
Gramd1c	3.287	1.681	-0.85	-0.19	
Wnt2b	0.106	0.048	-0.94	-0.19	
Tmeff1	0.187	0.080	-0.99	-0.20	
En1	0.014	0.000	-1.48	-0.20	
Srrm3	0.012	0.000	-1.39	-0.20	
Aqp1	43.892	24.841	-0.85	-0.20	
Ldb2	1.713	0.905	-0.83	-0.20	
Fcgbp	0.015	0.004	-1.18	-0.20	
Ptprb	1.643	0.927	-0.83	-0.20	
2810029C07Rik	0.133	0.054	-1.05	-0.20	
Gm16617	0.310	0.124	-1.04	-0.20	
Col16a1	1.138	0.563	-0.85	-0.20	
Zfp109	0.607	0.326	-0.82	-0.20	
Umod	0.011	0.000	-1.20	-0.20	
Ccr6	0.240	0.125	-0.90	-0.20	
2900009J06Rik	0.528	0.216	-1.03	-0.20	
Insl6	0.312	0.134	-1.08	-0.20	
Slc25a29	0.932	0.500	-0.83	-0.20	
Ugt2a1	0.065	0.023	-1.11	-0.20	
Faim2	0.012	0.003	-1.30	-0.20	
Fmo2	0.276	0.138	-0.88	-0.20	
Tmem117	0.017	0.000	-1.38	-0.20	
Gm14393	0.192	0.056	-1.13	-0.21	
B4galnt3	0.031	0.008	-1.23	-0.21	
Smok3a	0.016	0.000	-1.42	-0.21	
Gm13889	3.174	1.719	-0.84	-0.21	
Esrp1	0.124	0.058	-0.89	-0.21	
Slc6a9	6.027	3.486	-0.81	-0.21	
Sobp	0.032	0.013	-1.11	-0.21	
Col3a1	21.983	10.194	-0.84	-0.21	

Hsd3b5	2.874	1.527	-0.89	-0.21
Nav3	0.014	0.005	-1.11	-0.21
Cav1	2.869	1.349	-0.89	-0.22
Tnxb	0.815	0.380	-0.90	-0.22
Cmya5	0.008	0.002	-1.06	-0.22
Anxa13	0.225	0.100	-0.95	-0.22
Sh3tc2	1.566	0.875	-0.82	-0.22
Acot6	2.398	1.160	-0.92	-0.22
Ebf2	0.013	0.003	-1.17	-0.22
Wnt9b	0.052	0.023	-1.10	-0.22
Ank1	10.913	6.312	-0.84	-0.22
Krt42	0.102	0.027	-1.24	-0.22
6330403K07Rik	0.220	0.088	-1.02	-0.22
Egfbp2	0.525	0.147	-1.06	-0.22
Trpc1	0.479	0.256	-0.85	-0.23
Scn3b	0.034	0.010	-1.28	-0.23
Trim43c	0.081	0.035	-0.99	-0.23
Aldh1a1	1.668	0.834	-0.87	-0.23
Slc22a1	0.583	0.307	-0.91	-0.23
2900079G21Rik	0.022	0.009	-1.13	-0.23
Srpx2	0.318	0.141	-0.93	-0.23
Esrrb	0.062	0.027	-1.03	-0.23
Ppp1r26	0.065	0.028	-1.02	-0.23
Papln	0.427	0.211	-0.87	-0.23
Actl10	0.030	0.000	-1.51	-0.23
Cyp3a41b	121.615	69.879	-0.88	-0.23
Bhlhe40	20.489	10.254	-0.91	-0.23
Lgi3	0.052	0.019	-1.13	-0.23
Fbxw22	0.018	0.000	-1.64	-0.23
Sox18	2.637	1.432	-0.79	-0.23
Olfr159	0.258	0.123	-0.97	-0.24
Sox10	0.116	0.056	-0.93	-0.24
Pcdh17	0.568	0.292	-0.88	-0.24
Twist2	0.099	0.027	-1.13	-0.24

Col27a1	1.872	0.998	-0.88	-0.24	
Mir6240	1.086	0.000	-1.49	-0.24	
Pitpnm3	0.013	0.004	-1.23	-0.24	
Chst7	0.437	0.220	-0.91	-0.24	
Tmod2	0.012	0.003	-1.31	-0.24	
9530053A07Rik	0.004	0.000	-1.43	-0.24	
9030624G23Rik	0.044	0.013	-1.20	-0.24	
Zfp939	0.322	0.149	-0.97	-0.24	
Col6a1	5.883	2.819	-0.83	-0.24	
Wisp1	0.227	0.110	-0.89	-0.24	
Fitm1	0.226	0.090	-1.09	-0.25	
Itgb8	0.031	0.008	-1.35	-0.25	+
Zfp354c	0.273	0.134	-0.88	-0.25	
Vwa2	0.009	0.000	-1.45	-0.25	
Wipf3	0.235	0.115	-0.93	-0.25	
Mfap2	3.693	1.798	-0.86	-0.25	
Cyp3a44	68.327	37.758	-0.91	-0.25	
Wnt2	1.022	0.484	-0.95	-0.25	
Sult1c2	0.083	0.024	-1.23	-0.25	
Zscan4f	0.030	0.003	-1.42	-0.25	
Vsig8	0.113	0.038	-1.20	-0.25	
Hist1h2ak	13.996	5.359	-0.91	-0.25	
Klk1	0.238	0.087	-1.13	-0.25	
Lypd8	0.041	0.000	-1.49	-0.25	
4931406C07Rik	8.773	4.640	-0.91	-0.26	
Iglon5	0.021	0.002	-1.55	-0.26	
Krt16	0.075	0.019	-1.20	-0.26	
Cep192	3.114	1.231	-0.83	-0.26	
Xrra1	0.015	0.000	-1.50	-0.26	
Postn	9.893	4.921	-0.88	-0.26	
Efcab5	0.054	0.020	-1.14	-0.26	
Nefh	0.086	0.034	-1.12	-0.26	
Slc36a3	0.041	0.007	-1.37	-0.26	

Lyve1	17.857	9.785	-0.88	-0.26
Foxd3	0.019	0.000	-1.79	-0.26
Asxl3	0.031	0.014	-0.96	-0.26
Trpv1	0.046	0.016	-1.17	-0.26
Lamc2	0.289	0.134	-0.97	-0.27
Col15a1	0.366	0.146	-0.88	-0.27
Dpysl4	0.018	0.000	-1.62	-0.27
Nmnat2	0.031	0.010	-1.21	-0.27
Heyl	0.685	0.331	-0.88	-0.27
Pklr	54.805	31.518	-0.85	-0.27
2810410L24Rik	0.284	0.128	-1.05	-0.27
Wscd1	0.054	0.021	-1.11	-0.28
Adamts5	0.288	0.137	-1.00	-0.28
Tsku	3.798	1.919	-0.92	-0.28
Tfap2e	0.030	0.000	-1.62	-0.28
Abcg4	2.541	1.297	-0.93	-0.28
Adgrb1	0.020	0.006	-1.23	-0.28
9330175M20Rik	0.058	0.021	-1.20	-0.28
Duxbl3	0.462	0.197	-0.94	-0.28
Flrt1	0.052	0.019	-1.14	-0.28
Syndig1l	0.051	0.016	-1.34	-0.28
Mrgpra1	0.111	0.022	-1.47	-0.28
Dnase1l3	4.740	2.521	-0.88	-0.28
Fbxo43	0.013	0.000	-1.52	-0.29
Sult5a1	0.548	0.284	-0.97	-0.29
Slitrk6	0.023	0.006	-1.38	-0.29
Vmn2r46	0.017	0.002	-1.44	-0.29
Ldhb	1.738	0.898	-0.92	-0.29
Nyx	0.032	0.010	-1.24	-0.29
Ybx2	0.031	0.004	-1.45	-0.29
Tcf15	0.055	0.000	-1.55	-0.29
Smco3	0.053	0.017	-1.15	-0.29
Btnl10	6.141	3.161	-0.94	-0.30
Mc5r	0.078	0.015	-1.47	-0.30

Cma1	0.247	0.088	-1.12	-0.30
Apba2	0.012	0.000	-1.64	-0.30
Duxbl2	0.462	0.197	-0.97	-0.30
Sox9	0.523	0.253	-0.90	-0.30
Ranbp17	0.157	0.074	-1.01	-0.30
Csdc2	0.344	0.156	-0.99	-0.30
Gm6225	0.080	0.031	-1.14	-0.30
Sema5a	0.343	0.150	-0.97	-0.30
Jph4	0.017	0.003	-1.47	-0.30
Pitx1	0.017	0.000	-1.62	-0.31
Sulf1	0.578	0.288	-0.88	-0.31
Sult2a6	0.768	0.383	-1.04	-0.31
Map6	0.024	0.008	-1.21	-0.31
Gm21284	0.039	0.013	-1.24	-0.31
Fbxo47	0.083	0.025	-1.26	-0.31
Psg16	0.207	0.098	-0.99	-0.31
Cfap46	0.013	0.003	-1.23	-0.32
Gm27775	9.287	3.958	-1.07	-0.32
4930567H17Rik	0.083	0.007	-1.58	-0.32
2310002F09Rik	0.012	0.000	-1.52	-0.33
Serpina3k	361.008	213.877	-1.02	-0.33
Olfr421-ps1	0.086	0.022	-1.28	-0.33
Thsd7b	0.033	0.009	-1.24	-0.33
1700022A21Rik	0.075	0.020	-1.17	-0.33
Igsf3	0.535	0.259	-0.96	-0.33
Penk	0.279	0.113	-1.09	-0.33
Cyp1a2	0.446	0.233	-0.98	-0.33
Gm27740	9.287	3.958	-1.09	-0.34
Esrrg	0.115	0.047	-1.08	-0.34
Ptpru	0.351	0.147	-1.08	-0.34
Serinc2	0.566	0.281	-0.96	-0.34
Gria4	0.035	0.012	-1.30	-0.34
Ngf	0.382	0.157	-1.07	-0.34

Fxyd6	0.436	0.174	-1.10	-0.34
Gm5346	0.021	0.000	-1.44	-0.34
Casq2	0.262	0.115	-1.06	-0.35
Sspn	0.270	0.116	-1.03	-0.35
Hoxa10	0.030	0.004	-1.43	-0.35
Klf10	4.939	2.372	-1.00	-0.35
Nkain1	0.371	0.106	-1.04	-0.35
Dhtkd1	1.722	0.859	-0.98	-0.35
Elfn1	0.024	0.005	-1.53	-0.35
Spint1	0.331	0.114	-1.18	-0.35
Ptch2	0.180	0.088	-1.03	-0.36
Corin	0.038	0.012	-1.20	-0.36
Col4a6	0.063	0.025	-1.11	-0.36
Fzd2	0.276	0.122	-0.98	-0.36
Cdkl1	0.566	0.310	-1.01	-0.36
Dkkl1	0.241	0.098	-1.06	-0.36
Rpp25	0.225	0.087	-1.08	-0.36
Slc28a1	0.070	0.025	-1.30	-0.36
Cyp2g1	0.088	0.032	-1.24	-0.36
Cfap44	0.316	0.149	-1.02	-0.36
Dpep1	0.512	0.215	-1.03	-0.36
Ugt2a2	0.066	0.023	-1.27	-0.36
Smpdl3b	0.248	0.107	-1.04	-0.37
Ces1d	96.103	48.597	-0.97	-0.37
D630045M09Rik	0.090	0.023	-1.29	-0.37
Col6a2	5.623	2.393	-1.01	-0.37
Col1a2	15.684	6.894	-0.96	-0.37
Rbp2	99.523	54.726	-1.00	-0.37
Rarb	0.842	0.422	-0.98	-0.37
Ankrd61	0.415	0.173	-1.08	-0.37
Mir483	0.953	0.161	-1.46	-0.37
Tex40	0.077	0.015	-1.44	-0.38
Ndst3	0.023	0.007	-1.27	-0.38
Pdgfrl	0.407	0.177	-1.17	-0.39
Slc6a17	0.023	0.007	-1.33	-0.39

Mup12	0.364	0.073	-1.27	-0.39
Ins1	0.059	0.000	-1.60	-0.39
4931428F04Rik	0.307	0.136	-1.07	-0.39
1700011H14Rik	0.526	0.182	-1.14	-0.39
Ildr1	0.164	0.068	-1.12	-0.39
Fbn2	0.555	0.226	-0.99	-0.39
Krt19	3.419	1.333	-1.07	-0.39
Plekhg4	0.017	0.001	-1.56	-0.39
Npr3	0.989	0.486	-0.99	-0.39
Ppargc1b	0.534	0.254	-1.09	-0.39
Sntg1	0.022	0.007	-1.32	-0.39
Fabp3	0.322	0.101	-1.19	-0.40
Klk1b26	0.506	0.142	-1.21	-0.40
Mlph	0.027	0.009	-1.34	-0.40
Ptprt	0.094	0.042	-1.07	-0.40
Wnk2	0.119	0.051	-1.06	-0.40
Gm14827	0.014	0.000	-1.52	-0.40
3110039I08Rik	0.113	0.046	-1.04	-0.41
Stox2	0.145	0.065	-1.07	-0.41
Itgb6	0.012	0.000	-1.76	-0.41
Hspa12a	0.122	0.054	-1.05	-0.41
Wnt7b	0.066	0.025	-1.17	-0.41
Arx	0.457	0.300	-1.11	-0.41
Itga8	0.878	0.396	-1.04	-0.41
Ssc5d	0.143	0.050	-1.28	-0.41
Adamts8	0.085	0.029	-1.18	-0.42
Col9a3	0.162	0.066	-1.14	-0.42
Tpsb2	0.150	0.043	-1.32	-0.42
Nell2	0.038	0.007	-1.55	-0.42
Foxe1	0.057	0.005	-1.51	-0.42
Zscan4b	0.033	0.000	-1.78	-0.42
Xirp1	0.100	0.043	-1.09	-0.42
Tmem27	1.285	0.592	-1.07	-0.42
Gm4980	0.297	0.130	-1.08	-0.42

Epha3	0.391	0.151	-1.11	-0.42
Cd8b1	0.032	0.000	-1.63	-0.43
Syt14	1.294	0.597	-1.05	-0.43
Snord19	0.953	0.000	-1.74	-0.43
Fgfr3	1.380	0.650	-1.02	-0.43
Sulf2	0.481	0.175	-1.16	-0.43
Dclk1	0.021	0.006	-1.29	-0.44
Gm14725	0.175	0.043	-1.48	-0.44
Col1a1	17.449	6.931	-1.08	-0.44
Rbfox1	0.053	0.020	-1.18	-0.44
Gli1	0.150	0.061	-1.16	-0.44
Slc38a11	0.072	0.018	-1.25	-0.44
Apol7a	7.989	3.620	-1.09	-0.44
4930565N06Rik	0.108	0.037	-1.27	-0.45
Cacng7	0.245	0.078	-1.30	-0.45
Phyhip	1.119	0.485	-1.14	-0.45
Nkx2-3	0.072	0.022	-1.36	-0.45
Usp43	0.028	0.007	-1.42	-0.45
Sprr1a	0.337	0.104	-1.20	-0.45
Rec8	0.642	0.306	-1.06	-0.45
Cspg4	0.092	0.033	-1.22	-0.45
Pla2g4c	0.426	0.186	-1.14	-0.45
Serpina12	0.127	0.053	-1.18	-0.46
Zfp454	0.078	0.020	-1.42	-0.46
Hes1	3.352	1.440	-1.11	-0.46
Acot3	1.990	0.915	-1.00	-0.46
Efs	0.117	0.038	-1.35	-0.47
Kcnma1	0.025	0.006	-1.33	-0.47
Cyp17a1	1.770	0.818	-1.10	-0.47
Colgalt2	0.064	0.022	-1.30	-0.47
Hoxa5	0.387	0.154	-1.26	-0.47
Aff2	0.096	0.035	-1.17	-0.47
Emilin3	0.028	0.003	-1.56	-0.47
Sfrp1	2.441	0.934	-1.11	-0.47
Col6a6	0.161	0.054	-1.19	-0.47

Smcp	1.031	0.371	-1.22	-0.48
Islr	1.662	0.670	-1.13	-0.48
Acot5	1.142	0.469	-1.16	-0.48
Macc1	0.017	0.000	-1.68	-0.48
0610040F04Rik	0.898	0.401	-1.08	-0.48
Adamts15	0.262	0.112	-1.09	-0.48
Fat4	0.202	0.086	-1.10	-0.49
Adamtsl2	0.684	0.252	-1.18	-0.49
Nipal2	0.053	0.015	-1.36	-0.49
Slc26a10	0.638	0.268	-1.15	-0.49
Ccdc7a	0.027	0.000	-1.79	-0.49
Mir5132	2.898	0.912	-1.37	-0.49
Clic6	0.167	0.064	-1.18	-0.50
Aqp12	0.070	0.000	-1.77	-0.50
Itgae	0.169	0.048	-1.25	-0.50
Mme	0.029	0.008	-1.34	-0.50
Tceal7	0.078	0.012	-1.65	-0.50
Hoxd8	0.025	0.000	-1.95	-0.50
Slc27a3	0.354	0.148	-1.19	-0.50
Fgf21	0.585	0.267	-1.24	-0.50
Celsr1	0.115	0.049	-1.15	-0.50
Zfp287	0.104	0.042	-1.24	-0.50
Tmem132c	0.023	0.003	-1.71	-0.50
Col26a1	0.093	0.026	-1.44	-0.51
Pmepa1	0.293	0.105	-1.20	-0.51
Gstt3	5.555	2.511	-1.16	-0.51
Fras1	0.224	0.091	-1.16	-0.51
Meox2	0.085	0.023	-1.44	-0.51
Try5	0.072	0.000	-1.85	-0.52
Tcfl5	0.047	0.008	-1.52	-0.52
Ptges	0.919	0.400	-1.11	-0.52
Kcnb1	0.080	0.031	-1.24	-0.52
Gdf15	2.222	0.836	-1.15	-0.52
Epb4.1l4a	0.074	0.024	-1.34	-0.52
Crnde	0.254	0.090	-1.34	-0.53

Lrrc74b	0.034	0.005	-1.56	-0.53
Loxl1	1.300	0.446	-1.15	-0.53
Gpbar1	0.159	0.048	-1.45	-0.53
Rab25	0.280	0.089	-1.33	-0.53
Muc6	0.288	0.115	-1.21	-0.53
Sult2a1	14.746	6.883	-1.20	-0.53
Nrxn2	0.078	0.027	-1.31	-0.53
Ppp1r1a	0.750	0.305	-1.25	-0.53
Cd300lg	0.652	0.263	-1.21	-0.54
Tacr2	0.028	0.000	-1.69	-0.54
Slc8a3	0.069	0.022	-1.34	-0.54
Aldh1a7	0.917	0.363	-1.20	-0.54
4933431G14Rik	0.136	0.042	-1.30	-0.54
Fxyd2	0.179	0.041	-1.60	-0.54
Ccdc103	0.227	0.056	-1.44	-0.54
Cttnbp2	0.101	0.038	-1.21	-0.55
Cpz	0.687	0.263	-1.27	-0.55
Spock3	0.045	0.011	-1.43	-0.55
Trnp1	0.210	0.071	-1.29	-0.55
Mdk	1.801	0.596	-1.21	-0.55
Cyp2f2	8.813	3.926	-1.15	-0.55
Hspa1l	1.505	0.563	-1.20	-0.56
Krt17	0.116	0.027	-1.54	-0.56
Rnf112	0.047	0.008	-1.63	-0.56
Hsd11b2	0.105	0.029	-1.52	-0.56
Spag4	0.105	0.024	-1.51	-0.57
Gm6484	53.419	19.189	-1.16	-0.58
Has3	0.035	0.009	-1.60	-0.58
Mamdc2	0.175	0.063	-1.30	-0.58
Ntrk3	0.093	0.035	-1.29	-0.58
Muc5b	0.016	0.005	-1.46	-0.59
D130017N08Rik	0.117	0.035	-1.42	-0.60
Boc	0.291	0.105	-1.24	-0.60
Pdlim3	0.905	0.380	-1.19	-0.60

Gm8221	0.121	0.036	-1.39	-0.61
Cnr1	0.018	0.003	-1.61	-0.61
1500015O10Rik	0.167	0.040	-1.49	-0.61
1700001J03Rik	0.070	0.000	-2.18	-0.61
Shh	0.018	0.000	-2.01	-0.62
Adgrd1	0.212	0.073	-1.28	-0.62
Snora69	0.719	0.048	-1.84	-0.62
Ntm	0.110	0.033	-1.40	-0.62
Slc28a3	0.013	0.000	-1.99	-0.62
Hcn3	1.092	0.442	-1.28	-0.63
Apol7c	0.899	0.337	-1.28	-0.63
Gabra3	0.140	0.041	-1.42	-0.63
Fbp2	0.332	0.077	-1.43	-0.64
Spata21	0.048	0.005	-1.80	-0.64
Piezo2	0.082	0.030	-1.35	-0.64
Zfp931	0.777	0.314	-1.35	-0.64
Nov	0.508	0.137	-1.41	-0.64
Lrrc75b	0.058	0.014	-1.54	-0.65
Dnah17	1.019	0.401	-1.30	-0.65
Ptgis	0.712	0.229	-1.33	-0.65
Ntn1	0.570	0.217	-1.32	-0.65
Gm8580	2.605	1.464	-1.36	-0.66
Trabd2b	0.200	0.067	-1.38	-0.67
E030013I19Rik	0.058	0.008	-1.72	-0.67
Krt15	0.331	0.092	-1.48	-0.67
Gpr161	0.075	0.015	-1.55	-0.68
Fermt1	0.071	0.021	-1.39	-0.68
Cldn6	1.052	0.336	-1.41	-0.68
Cfap74	0.032	0.006	-1.60	-0.68
Fhad1	0.496	0.217	-1.30	-0.68
Tulp1	0.216	0.070	-1.52	-0.68
Ccr10	0.037	0.000	-2.00	-0.68
Itga7	0.087	0.025	-1.44	-0.69
Mup21	0.156	0.023	-1.62	-0.69

Cdh17	0.031	0.002	-1.97	-0.70
Myo16	0.094	0.024	-1.52	-0.70
Begain	0.166	0.050	-1.53	-0.70
Anks6	0.084	0.027	-1.51	-0.70
Crisp2	0.052	0.004	-1.91	-0.71
Mrap	4.762	1.845	-1.31	-0.71
Btnl9	0.659	0.249	-1.41	-0.71
Cdh6	0.146	0.042	-1.44	-0.71
Fjx1	0.473	0.158	-1.42	-0.71
Sry	0.074	0.000	-1.97	-0.71
Sema3c	0.247	0.083	-1.35	-0.72
Olfml2a	0.137	0.037	-1.59	-0.72
Npas1	0.094	0.014	-1.68	-0.72
Trp53cor1	0.130	0.043	-1.46	-0.72
Lgi2	0.443	0.134	-1.45	-0.72
Eps8l1	0.386	0.171	-1.40	-0.72
Osbpl6	0.035	0.009	-1.49	-0.73
Vgll3	0.099	0.027	-1.50	-0.73
Dnah7a	0.012	0.001	-1.96	-0.73
Arnt2	0.062	0.016	-1.44	-0.73
Xlr5c	0.157	0.000	-1.85	-0.73
Ror1	0.111	0.030	-1.43	-0.73
Ccl19	0.223	0.025	-1.74	-0.74
Gm10591	2.498	0.604	-1.40	-0.74
Sfrp2	0.280	0.076	-1.46	-0.74
Ccl21c	2.498	0.604	-1.39	-0.75
Ccl21b	2.531	0.630	-1.40	-0.75
4930444P10Rik	0.041	0.000	-1.91	-0.75
Dct	0.746	0.239	-1.41	-0.75
Gjc2	0.231	0.065	-1.49	-0.75
Gm21541	2.498	0.604	-1.39	-0.75
Col9a2	0.380	0.124	-1.39	-0.76
Ror2	0.152	0.044	-1.51	-0.76
Nrsn1	0.091	0.006	-1.81	-0.76
Vipr1	0.843	0.311	-1.37	-0.76

Trpc3	0.076	0.018	-1.62	-0.77
Kcnk1	0.147	0.041	-1.60	-0.77
Asb11	0.094	0.009	-1.95	-0.78
Shisa7	0.018	0.002	-1.96	-0.78
Dcdc2a	0.315	0.097	-1.42	-0.78
Duxbl1	0.503	0.144	-1.51	-0.78
Gm16157	0.438	0.119	-1.49	-0.78
Cyp4a14	54.876	21.270	-1.36	-0.79
2410017I17Rik	1.468	0.826	-1.40	-0.79
Ugt8a	0.192	0.062	-1.51	-0.80
Ano2	0.077	0.016	-1.65	-0.80
Stc1	0.308	0.109	-1.42	-0.80
Tril	0.137	0.037	-1.63	-0.80
Grhl2	0.040	0.008	-1.74	-0.80
Pkd2l1	0.021	0.000	-2.00	-0.81
Sost	0.107	0.031	-1.64	-0.81
Inhbe	4.617	1.726	-1.41	-0.81
Creb5	0.071	0.014	-1.89	-0.81
Prr18	0.048	0.010	-1.83	-0.82
Gdpd2	0.040	0.004	-1.82	-0.82
Htra3	0.698	0.208	-1.48	-0.82
Usp36	13.388	1.286	-1.46	-0.83
Cyp2e1	134.956	48.553	-1.56	-0.83
6030443J06Rik	0.046	0.008	-1.80	-0.83
Mup15	0.727	0.083	-1.59	-0.83
Kcne4	0.215	0.065	-1.64	-0.84
Clasp2	9.721	1.024	-1.51	-0.85
Kcnq1	0.234	0.072	-1.61	-0.85
Zfp503	0.623	0.197	-1.52	-0.85
Calr4	0.029	0.000	-1.93	-0.86
Myoz1	1.069	0.473	-1.51	-0.86
Slc5a1	0.025	0.000	-2.30	-0.86
Tdo2	0.637	0.205	-1.60	-0.86
Prmt8	0.059	0.007	-1.78	-0.86
Gm13304	2.498	0.604	-1.45	-0.86

Lrrc55	0.030	0.000	-2.13	-0.86
1600015I10Rik	0.050	0.002	-1.97	-0.87
Muc1	0.263	0.058	-1.63	-0.88
Ankrd29	0.061	0.013	-1.82	-0.90
Mtcl1	0.060	0.013	-1.70	-0.90
Rgs5	0.403	0.120	-1.62	-0.90
Ptn	0.892	0.224	-1.58	-0.90
Tppp3	1.014	0.287	-1.66	-0.90
D030025P21Rik	0.037	0.006	-1.91	-0.91
Mc2r	0.111	0.022	-1.76	-0.91
Cxxc4	0.043	0.009	-1.71	-0.92
Ecm2	0.292	0.088	-1.59	-0.92
Mup1	0.308	0.043	-1.76	-0.92
Sncaip	0.250	0.065	-1.65	-0.92
Chgb	0.032	0.000	-2.13	-0.93
Vmn2r29	0.153	0.045	-1.72	-0.93
Col6a5	0.022	0.003	-1.87	-0.94
Figf	0.718	0.184	-1.62	-0.95
Adcyap1r1	0.017	0.000	-2.06	-0.95
Map3k7cl	0.200	0.039	-1.81	-0.95
Axin2	0.260	0.075	-1.61	-0.95
Lrrc17	0.611	0.148	-1.61	-0.95
Cyp1a1	0.050	0.004	-2.02	-0.95
Fam198a	0.227	0.058	-1.71	-0.96
Pax1	0.039	0.000	-2.51	-0.96
Xlr5a	0.101	0.000	-2.27	-0.96
Kcnt1	0.009	0.000	-2.11	-0.96
Cdcp1	0.411	0.130	-1.60	-0.97
Cyp3a57	16.430	5.123	-1.59	-0.97
Adra1a	0.027	0.002	-2.12	-0.97
Bmp6	0.472	0.132	-1.71	-0.98
Treh	0.108	0.020	-1.85	-0.99
Hspa1a	5.269	1.207	-1.58	-0.99
Tnnt2	0.839	0.216	-1.66	-0.99
Serpina9	0.045	0.000	-2.15	-1.00

Pgm5	0.135	0.034	-1.69	-1.00
Chodl	0.039	0.000	-2.13	-1.00
Spock2	0.099	0.018	-1.83	-1.01
Cdh13	0.168	0.039	-1.81	-1.01
Prrx1	0.118	0.030	-1.77	-1.01
Nell1	0.038	0.002	-2.21	-1.01
Dnah7b	0.031	0.006	-1.83	-1.01
Gm6086	0.059	0.007	-1.93	-1.02
Neto1	0.024	0.000	-2.13	-1.03
Serpina5	1.235	0.384	-1.65	-1.03
Dkk2	0.096	0.022	-1.77	-1.03
Apoa4	18.328	6.480	-1.66	-1.03
D630041G03Rik	0.089	0.019	-1.89	-1.03
Olfr78	0.055	0.000	-2.60	-1.03
Synpo2	0.028	0.005	-1.89	-1.04
Lrrn1	0.114	0.022	-1.86	-1.04
Stmn2	0.129	0.019	-2.05	-1.04
BC061212	0.038	0.000	-2.16	-1.04
Cldn8	0.065	0.005	-2.04	-1.05
Scml2	0.435	0.107	-1.80	-1.05
1700067K01Rik	0.120	0.011	-2.11	-1.05
Pnmal1	0.088	0.010	-2.12	-1.06
Cyp3a59	24.416	6.894	-1.80	-1.06
Megf10	0.014	0.000	-2.43	-1.06
Sema5b	0.080	0.016	-1.98	-1.07
Cyp3a25	69.469	21.519	-1.69	-1.07
Prss35	0.954	0.222	-1.78	-1.07
H2-M10.1	0.120	0.000	-2.43	-1.08
Mgp	2.299	0.501	-1.71	-1.09
Ptgfr	0.061	0.009	-2.07	-1.10
Klk14	0.138	0.014	-2.13	-1.11
Egflam	0.348	0.093	-1.81	-1.11
E030003E18Rik	0.162	0.011	-2.16	-1.11

Pcdh20	0.107	0.022	-1.96	-1.11
Mup14	0.399	0.032	-2.12	-1.12
4930554I06Rik	0.185	0.000	-2.32	-1.12
Thbs3	0.441	0.111	-1.86	-1.14
Mup13	0.335	0.024	-2.24	-1.14
Gm9994	0.161	0.010	-2.34	-1.15
Ace2	0.188	0.036	-1.94	-1.15
Mup7	1.002	0.103	-1.92	-1.15
Thsd7a	0.145	0.039	-1.82	-1.18
Mrgprf	0.106	0.018	-2.11	-1.18
Mfap4	3.984	0.945	-1.78	-1.18
Mup2	0.353	0.028	-2.06	-1.18
Hspa1b	5.739	1.102	-1.82	-1.18
Slc6a20a	0.853	0.233	-1.84	-1.19
Ano9	1.184	0.358	-1.82	-1.19
Dcx	0.061	0.012	-1.98	-1.20
Pnmal2	0.050	0.003	-2.37	-1.21
Kcnk3	0.091	0.014	-2.07	-1.21
Gm7978	0.038	0.000	-2.53	-1.21
Faxc	0.027	0.004	-2.19	-1.22
Tdpoz5	0.056	0.000	-2.46	-1.22
Id4	0.301	0.057	-2.11	-1.22
Ddi1	0.092	0.004	-2.34	-1.23
Pnpla5	0.138	0.017	-2.22	-1.23
Gm11762	0.106	0.000	-2.55	-1.25
Col2a1	0.259	0.054	-1.95	-1.25
Eln	11.679	2.234	-1.89	-1.26
Fbln2	1.444	0.354	-1.86	-1.26
H2-M10.4	0.246	0.000	-2.65	-1.27
Dnaic2	0.877	0.234	-1.94	-1.28
Gm10697	0.056	0.000	-2.69	-1.30
Fam196b	0.030	0.002	-2.39	-1.30
Gm1987	2.713	0.413	-1.98	-1.30
Fam189a2	0.138	0.021	-2.12	-1.31
2310081J21Rik	0.196	0.000	-2.50	-1.31

Mup9	0.282	0.020	-2.35	-1.31
Spp1	27.419	6.358	-1.97	-1.34
Ccl21a	2.646	0.380	-2.10	-1.35
Rab30	9.985	1.766	-1.99	-1.37
Htra1	0.318	0.043	-2.21	-1.38
Slc6a20b	0.248	0.054	-2.06	-1.39
Snord68	5.674	0.736	-2.27	-1.39
Mecom	0.297	0.064	-2.03	-1.39
Ltbp2	0.483	0.094	-2.04	-1.39
Osr2	0.076	0.000	-2.44	-1.41
Ptprz1	0.017	0.000	-2.59	-1.42
Fut9	0.010	0.000	-2.52	-1.43
Gal3st2	0.139	0.014	-2.42	-1.43
2810002D19Rik	4.498	0.786	-2.11	-1.44
Krtap7-1	0.297	0.000	-2.80	-1.44
Myocd	0.031	0.000	-2.68	-1.45
G6pc2	0.142	0.005	-2.44	-1.50
Barx1	0.140	0.000	-2.68	-1.53
Erich6	0.103	0.003	-2.73	-1.53
Aspn	0.574	0.078	-2.32	-1.56
Mup17	0.273	0.007	-2.60	-1.58
Krt25	0.132	0.000	-2.66	-1.61
Apoc3	64.776	13.303	-2.26	-1.61
Adamts19	0.021	0.000	-2.66	-1.62
Mup8	0.368	0.012	-2.73	-1.62
Prnd	0.096	0.007	-2.51	-1.62
Mup16	0.240	0.000	-2.78	-1.62
S100b	0.142	0.007	-2.55	-1.64
Scara3	0.534	0.092	-2.40	-1.67
Shisa9	0.111	0.015	-2.54	-1.68
Adgrb2	0.042	0.002	-2.70	-1.68
Osr1	0.144	0.006	-2.76	-1.68
Cilp	0.036	0.000	-3.03	-1.69
Agtr2	0.123	0.008	-2.55	-1.70
Hspb7	0.122	0.009	-2.80	-1.71

Spon1	0.340	0.041	-2.43	-1.72
Xlr5b	0.128	0.000	-2.89	-1.73
Unc5c	0.024	0.001	-2.90	-1.74
Cthrc1	0.439	0.040	-2.69	-1.75
Gabrp	0.074	0.000	-3.17	-1.77
Kcne1l	0.176	0.008	-2.74	-1.79
Cyp26b1	0.286	0.044	-2.60	-1.81
Myh3	0.107	0.012	-2.69	-1.81
Wnt5a	0.119	0.011	-2.70	-1.81
Mup10	0.453	0.007	-2.99	-1.81
Fndc1	0.157	0.014	-2.77	-1.87
Ctrb1	0.266	0.000	-3.05	-1.87
Gm4567	0.050	0.000	-3.26	-1.89
Lamc3	0.034	0.000	-3.09	-1.90
Clec3b	0.552	0.036	-2.87	-1.90
Ehf	0.271	0.027	-2.76	-1.93
Col13a1	0.886	0.128	-2.70	-1.94
Lcn4	0.305	0.000	-3.23	-2.00
Cd209f	4.200	0.568	-2.67	-2.00
LOC100048884	0.345	0.000	-3.26	-2.01
Mup19	0.424	0.007	-3.22	-2.02
Mfap5	0.270	0.013	-2.98	-2.02
Tsix	2.917	0.608	-2.67	-2.05
Aoc3	0.209	0.017	-2.98	-2.07
Nrcam	0.049	0.004	-3.04	-2.09
Pi15	0.153	0.012	-2.89	-2.12
Mup11	0.361	0.000	-3.36	-2.12
Gck	3.796	0.494	-2.82	-2.13
Gm2083	0.483	0.007	-3.32	-2.16
Adamts16	0.201	0.018	-2.97	-2.16
Extl1	0.190	0.016	-3.00	-2.17
Ntrk2	0.049	0.002	-3.18	-2.22
Cd209g	3.082	0.358	-2.85	-2.22
Gm6902	0.051	0.000	-3.22	-2.23
Dpt	1.262	0.083	-3.07	-2.23

Xist	16.349	7.161	-2.93	-2.29
Hcrtr1	0.602	0.005	-3.37	-2.30
Thbs4	0.107	0.002	-3.55	-2.38
Lrtm1	0.668	0.077	-3.06	-2.41
Ccdc74a	3.235	0.216	-3.35	-2.58
Ube2ql1	4.046	0.042	-3.42	-2.69
9230112J17Rik	0.911	0.005	-3.81	-2.74
Gm13293	2.268	0.007	-3.88	-2.76
Dmbt1	0.087	0.000	-4.08	-2.78
Gja5	0.985	0.078	-3.47	-2.84
Klk1b5	30.800	0.109	-3.75	-2.93
Slc15a2	0.942	0.058	-3.77	-3.00
Thrsp	5.656	0.403	-3.66	-3.00
Olfr77	1.250	0.000	-4.28	-3.01
Galnt16	0.154	0.000	-4.38	-3.34
Ar	5.615	0.000	-6.10	-4.73

Log2FC: log2-fold change

GFOLD (0.1): conservatively estimated log2-fold change at q = 0.1 confidence level

Inflammatory: HALLMARK_INFLAMMATORY_RESPONSE

Supplemental Table 7. Differentially expressed genes in the skin at q <0.1

Gene	WT RPKM	*Jak1*$^{H595D/+;I596I/+;Y597Y/+}$ RPKM	Log2FC	GFOLD(0.1)	IFNg	NFKB
Sprr2a3	53.161	943.241	5.16	4.71		
Sprr2a2	42.946	711.922	5.02	4.55		
Sprr2a1	42.946	711.922	5.06	4.55		
Sprr2b	28.814	470.914	5.00	4.55		
Gm5476	0.695	15.513	4.51	3.96		
Fetub	0.081	1.816	4.29	3.72		
Gm5878	1.054	2.425	4.20	3.72		
Glycam1	0.258	5.014	4.22	3.64		
1300017J02Rik	0.080	1.288	3.69	3.12		
Frem3	0.006	0.133	3.46	2.70		
Cxcl1	1.433	8.150	3.13	2.64		+
4930502E18Rik	0.049	0.806	3.34	2.62		
Sprr2k	0.034	0.966	3.50	2.59		
Tacr1	0.087	0.732	3.12	2.58		
Gm5477	0.922	6.208	2.96	2.43		
Chil1	2.814	20.575	2.87	2.40		
Defa24	0.051	1.315	3.31	2.39		
Serpinb3c	7.652	46.436	2.81	2.39		
Krt16	52.014	313.445	2.83	2.38		
Il6	0.183	1.460	2.90	2.35	+	+
Krt33a	0.849	4.147	2.76	2.29		
Fosb	9.284	31.011	2.70	2.25		+
Tnfrsf9	1.898	10.239	2.62	2.23		+
Snora30	0.251	5.149	3.14	2.21		
1700001J03Rik	0.083	0.963	2.85	2.20		
Otx2os1	0.420	1.720	2.80	2.17		
Sprr2i	0.078	1.071	2.97	2.09		
Xist	22.675	30.540	2.50	2.03		
Sele	0.733	3.632	2.46	2.02		
Krt6b	79.466	368.716	2.41	1.99		

Chit1	2.638	11.929	2.34	1.89		
Fgf23	0.440	2.283	2.38	1.89		
Spp1	0.555	2.738	2.32	1.86		
Il1b	0.575	2.308	2.31	1.84		+
Lrg1	3.728	17.247	2.27	1.82		
Mir7092	0.870	7.777	2.58	1.81		
Gm20757	0.000	0.165	3.03	1.77		
Krt74	1.323	4.244	2.25	1.76		
Gm6377	0.154	0.802	2.30	1.74		
4930432K21Rik	0.384	1.838	2.25	1.74		
Snord33	85.372	178.704	2.19	1.72		
4930429F11Rik	0.000	0.136	2.73	1.66		
Irg1	0.002	0.085	2.69	1.65		
Serpinb13	0.164	0.795	2.20	1.62		
Atf3	12.756	42.321	2.05	1.61		+
Sectm1b	0.049	0.256	2.36	1.60		
Egr1	46.736	153.461	2.01	1.58		+
Gm11567	0.000	0.190	2.66	1.57		
Defa6	0.038	0.938	2.61	1.55		
Krtap6-1	0.000	0.297	2.61	1.55		
Ctsj	2.043	6.276	2.04	1.54		
Cyr61	95.270	312.498	2.00	1.51		
Foxg1	0.019	0.127	2.30	1.51		
Wbp2nl	0.008	0.167	2.43	1.50		
Gm11562	0.000	0.218	2.70	1.50		
Fos	77.948	201.701	1.91	1.48		+
Ltb4r2	0.280	1.118	2.00	1.47		
Gsdmc	5.029	16.929	1.93	1.47		
Zfp36	39.867	129.817	1.96	1.46		+
Socs3	9.721	32.932	1.90	1.46	+	+
Sprr2j-ps	0.032	0.393	2.42	1.45		
Ankrd1	1.362	4.182	1.93	1.45		
Akr1d1	0.241	0.710	1.96	1.45		
Cd69	0.690	2.503	1.91	1.44	+	+

Hbq1b	0.093	0.648	2.27	1.44
Gm3285	0.032	0.365	2.31	1.44
C4b	5.374	13.741	1.86	1.42
Ros1	0.007	0.054	2.28	1.41
Has1	1.000	3.845	1.92	1.41
Dsg1c	4.128	9.990	1.86	1.38
7420461P10Rik	0.000	0.208	2.61	1.38
1700063D05Rik	0.000	0.218	2.40	1.38
Gm19434	0.036	0.206	2.13	1.37
Oasl1	1.132	3.984	1.79	1.37
Tex21	0.010	0.098	2.21	1.37
Dnase1l3	0.781	2.425	1.86	1.36
Krtap17-1	1.783	4.939	1.78	1.34
Fam26d	0.100	0.314	1.92	1.33
Tgtp1	0.668	2.149	1.84	1.33
Tgtp2	0.664	2.022	1.78	1.32
Sprr2e	0.025	0.341	2.25	1.31
Cxcl13	1.578	4.381	1.76	1.31
Ccr8	0.456	1.601	1.87	1.31
Ereg	0.256	0.719	1.79	1.29
Gm10229	0.000	0.309	2.32	1.29
Krt31	0.707	2.119	1.80	1.29
2610316D01Rik	1.878	5.640	1.73	1.28
Klrb1f	0.242	0.890	1.83	1.28
Mmp12	0.052	0.195	1.92	1.28
Ifitm7	4.575	11.591	1.72	1.27
Treml2	0.063	0.239	1.85	1.27
Gm10228	0.000	0.280	2.42	1.27
Borg	0.010	0.088	2.13	1.27
Apol11b	0.000	0.064	2.40	1.27
Ugt3a1	0.117	0.422	1.88	1.26
Bpifb5	0.007	0.135	2.25	1.26
Lair1	0.050	0.200	1.85	1.25

Trem1	0.076	0.284	1.83	1.25		
Egr2	2.292	6.466	1.68	1.25		+
Krt6a	77.557	217.156	1.67	1.23		
Nfkbiz	1.947	5.500	1.69	1.22		
F630042J09Rik	0.027	0.139	2.03	1.22		
Clec4e	0.075	0.289	1.87	1.22		
Cxcl10	1.198	3.746	1.71	1.22	+	+
Gm7609	0.848	2.572	1.75	1.22		
Serpina3n	2.236	5.517	1.63	1.22		
Serpinb3a	16.530	48.711	1.70	1.21		
Naa11	0.013	0.085	2.16	1.21		
Gm14685	0.382	1.160	1.74	1.20		
Foxi2	0.029	0.145	1.93	1.20		
C4a	2.467	5.927	1.70	1.20		
Ifit1	1.722	5.446	1.69	1.20	+	
9130230L23Rik	2.174	3.061	1.69	1.20		
Scgb1a1	0.701	2.590	1.83	1.19		
Ifit3b	2.636	8.111	1.66	1.19		
Aqp2	0.015	0.140	2.10	1.18		
Rsad2	2.253	6.736	1.64	1.18	+	
Dok2	4.791	14.939	1.62	1.17		
Saa3	0.142	0.705	1.93	1.17		
Tmc7	1.241	3.718	1.62	1.16		
Tnfsf11	0.065	0.276	1.82	1.15		
Ccl2	3.742	11.264	1.67	1.15	+	+
Fam186b	0.013	0.083	2.05	1.15		
Serpinb2	2.674	6.857	1.62	1.15		+
Stfa2	83.472	238.954	1.61	1.15		
Gm15880	0.047	0.183	1.86	1.14		
Serpinb3b	5.611	14.407	1.55	1.14		
Krtap7-1	0.173	0.745	1.93	1.14		
Cxcr2	0.074	0.290	1.81	1.14		
Sectm1a	0.519	1.665	1.64	1.14		
Lhx9	0.009	0.058	2.06	1.13		

4933402N22Rik	0.000	0.119	2.27	1.13	
Snora68	19.203	45.196	1.62	1.13	
Clec4d	0.258	0.841	1.69	1.13	
DXBay18	0.379	1.063	1.57	1.11	
Wfdc5	2.543	6.230	1.55	1.11	
BC018473	0.002	0.041	2.13	1.10	
Serpine1	3.562	10.325	1.55	1.10	+
Fam46b	2.714	6.193	1.54	1.10	
Mcpt8	0.410	1.339	1.69	1.10	
Krtap3-1	0.089	0.511	1.90	1.09	
Pydc3	0.551	1.512	1.58	1.09	
Saa1	4.952	2.505	1.68	1.09	
Proc	0.085	0.271	1.78	1.09	
Slfn4	0.548	1.551	1.57	1.08	
Ptchd3	0.012	0.068	1.98	1.08	
Cabp4	0.743	2.014	1.66	1.08	
Nhlh2	0.003	0.053	2.24	1.08	
Nr2e3	0.070	0.254	1.74	1.07	
Tsix	0.875	1.617	1.50	1.07	
Klrb1b	0.413	1.215	1.60	1.07	
Gm12185	0.055	0.192	1.70	1.07	
Ccr6	0.016	0.106	2.04	1.06	
Hspa1a	20.437	52.963	1.51	1.06	
Junb	35.738	90.861	1.49	1.06	+
Krt73	21.332	35.765	1.55	1.06	
Taf7l	0.019	0.137	2.01	1.06	
Slc22a13	0.070	0.275	1.78	1.05	
Slc16a14	0.043	0.188	1.81	1.05	
Krt10	690.530	#######	1.54	1.05	
Gadl1	1.259	3.174	1.53	1.05	
Serpina3i	1.150	3.155	1.55	1.04	
Sohlh2	0.011	0.094	2.02	1.04	
Zfp457	0.127	0.417	1.69	1.04	
Sytl3	1.887	5.410	1.52	1.04	
Ctsq	0.069	0.283	1.72	1.04	

St6galnac5	0.880	2.469	1.53	1.03		
Gm4489	0.000	0.254	2.16	1.03		
Ifit3	5.164	14.757	1.50	1.03	+	
Selp	1.261	3.602	1.52	1.03	+	
Pglyrp2	0.047	0.169	1.72	1.02		
0610043K17Rik	0.087	0.464	1.90	1.02		
Atcayos	8.877	21.135	1.46	1.02		
Gm436	0.066	0.288	1.80	1.02		
Tchh	4.466	11.069	1.50	1.02		
1700012D14Rik	0.239	0.876	1.74	1.01		
Gm11665	0.040	0.225	1.92	1.01		
Prr9	3.181	6.315	1.46	1.01		
Ttll8	0.000	0.044	2.05	1.00		
Dsg1b	6.571	13.232	1.48	1.00		
Cd22	0.040	0.150	1.73	1.00		
Gm29684	0.016	0.122	1.99	1.00		
Alox8	0.212	0.613	1.51	1.00		
Gm5409	0.014	0.207	2.15	0.99		
Ccl7	3.378	9.239	1.45	0.99	+	
Gsdmc2	1.477	3.193	1.42	0.99		
Rln1	0.247	0.790	1.67	0.99		
Gm11938	0.049	0.299	1.89	0.98		
Cd300ld	0.342	0.950	1.49	0.97		
Cd83	3.857	9.500	1.43	0.97		+
D730001G18Rik	0.563	1.313	1.49	0.97		
Pou2f2	0.191	0.526	1.52	0.97		
Ccl4	0.538	1.569	1.55	0.97		+
Gm15910	0.027	0.119	1.73	0.96		
Il22ra2	0.224	0.545	1.55	0.96		
Vwc2l	0.000	0.024	2.11	0.96		
Epx	0.064	0.213	1.63	0.96		
Thbs1	21.004	55.492	1.42	0.95		
Zic1	2.735	3.184	1.40	0.94		

Hmx1	9.199	17.550	1.37	0.94		
Bsnd	0.131	0.398	1.51	0.94		
Ccl19	3.435	8.355	1.42	0.93		
Hspa1b	22.414	54.062	1.40	0.93		
Gm6455	0.000	0.103	2.06	0.93		
Krt71	21.309	36.946	1.36	0.92		
Klrb1a	0.084	0.311	1.63	0.91		
Drd1	0.092	0.271	1.51	0.90		
Npy2r	0.557	1.069	1.41	0.90		
Krtap9-1	0.000	0.180	2.09	0.90		
Grm5	0.002	0.021	2.00	0.90		
Krt26	1.092	2.026	1.41	0.89		
Krt28	31.354	53.350	1.33	0.89		
Pkd1l2	0.049	0.131	1.51	0.89		
Grin2a	0.004	0.039	1.95	0.89		
Krt1	852.233	#######	1.31	0.88		
Krt80	4.458	10.459	1.34	0.88		
Gm29683	0.013	0.088	1.84	0.88		
Pydc4	0.253	0.658	1.43	0.87		
Apold1	8.550	19.325	1.29	0.87		
Col10a1	0.934	1.830	1.29	0.87		
Il1f9	3.016	6.315	1.34	0.87		
Zfp831	0.022	0.067	1.52	0.87		
Maff	4.952	11.947	1.30	0.87		+
Gprin2	0.087	0.303	1.59	0.87		
Gm5294	0.132	0.508	1.71	0.87		
Olfr378	0.080	0.326	1.67	0.87		
1110025L11Rik	0.000	0.223	2.12	0.87		
Abra	3.393	6.703	1.32	0.86		
Ccl8	0.337	1.035	1.51	0.86		
Usp18	1.904	4.405	1.35	0.86	+	
Slamf6	0.007	0.063	1.87	0.85		
Fam179a	0.427	1.063	1.38	0.85		
Stfa2l1	18.377	43.965	1.30	0.85		
Tbrg3	0.003	0.022	1.90	0.85		

2010109I03Rik	3.368	7.637	1.37	0.85		
Gm5936	0.240	0.599	1.39	0.84		
Fcgr4	1.338	3.420	1.36	0.84		
Htr1f	0.255	0.615	1.39	0.83		
Ranbp3l	0.022	0.078	1.60	0.83		
Krt25	34.813	55.777	1.30	0.83		
Tlr12	0.090	0.264	1.45	0.82		
Tesc	3.299	7.579	1.30	0.81		
Tgm6	0.143	0.348	1.37	0.81		
Sh2d2a	0.045	0.120	1.44	0.80		
Helt	0.018	0.101	1.77	0.80		
Cebpd	12.904	30.684	1.25	0.80		+
Nr4a1	20.076	44.436	1.22	0.80		+
Apol9b	0.334	0.884	1.38	0.80		
Rnf222	0.689	1.371	1.26	0.80		
Mir322	0.906	3.247	1.65	0.80		
Psors1c2	1.728	4.078	1.30	0.80		
Trim6	0.375	0.829	1.26	0.80		
Gm1966	0.185	0.402	1.28	0.79		
Krt83	14.434	26.658	1.23	0.79		
Jun	67.824	141.693	1.22	0.79		+
Mir215	0.000	0.794	2.02	0.79		
Cysltr1	0.549	1.339	1.32	0.79		
Gpr75	0.033	0.121	1.58	0.78		
Cyp2b10	1.600	3.262	1.28	0.78		
Tmem28	0.494	1.089	1.33	0.78		
Ier2	47.945	102.114	1.21	0.78		+
Apol10b	0.119	0.300	1.51	0.78		
Irf7	7.341	16.642	1.20	0.78	+	
Il1f5	8.924	17.089	1.23	0.78		
Csf2rb2	6.305	14.047	1.19	0.77		
B3gnt6	0.039	0.130	1.62	0.77		
Nr5a1	0.073	0.224	1.46	0.77		
9830166K06Rik	0.012	0.064	1.67	0.77		
S100a3	7.234	12.999	1.25	0.77		

Pou3f2	0.023	0.070	1.44	0.77	
5430427M07Rik	0.095	0.323	1.63	0.76	
Eppk1	4.138	7.641	1.18	0.76	
Mir432	9.750	18.216	1.32	0.76	
Gm8221	0.001	0.016	1.87	0.76	
Skint10	0.992	2.039	1.22	0.76	
Rgs9bp	0.207	0.468	1.24	0.76	
Tdh	1.927	3.678	1.21	0.75	
Car6	0.915	2.189	1.27	0.75	
Tyrp1	3.837	5.935	1.27	0.75	
Hist1h2ak	24.195	43.208	1.21	0.75	
Rit2	0.027	0.135	1.69	0.75	
Zbp1	1.294	2.876	1.22	0.75	+
Rbp2	13.106	26.299	1.16	0.74	
Hbq1a	0.080	0.365	1.63	0.74	
Gm438	0.045	0.185	1.66	0.74	
Rasa4	5.000	11.211	1.23	0.74	
Gbp5	0.457	1.123	1.30	0.74	
Lypd5	4.359	7.212	1.19	0.74	
Hal	15.278	29.330	1.19	0.74	
Apol9a	0.332	0.813	1.26	0.73	
Slc5a9	0.476	1.016	1.19	0.73	
Cd84	0.769	1.699	1.21	0.73	
Trim60	0.007	0.049	1.78	0.73	
Gfap	0.442	0.812	1.24	0.73	
Serpinb12	19.288	34.876	1.20	0.73	
Gm5414	40.703	84.131	1.17	0.73	
Acer2	6.405	14.091	1.16	0.73	
Gm15408	0.441	1.055	1.27	0.72	
Gm19299	0.075	0.230	1.45	0.72	
Mnda	20.873	43.185	1.15	0.72	
Clu	11.384	25.656	1.20	0.72	
Skint9	6.086	10.343	1.13	0.72	
Gm6277	0.510	1.271	1.30	0.72	
Dkkl1	8.765	17.131	1.16	0.72	

1700007K09Rik	0.056	0.293	1.62	0.71	
Foxi1	0.057	0.178	1.49	0.71	
Serpinb3d	1.113	2.234	1.22	0.71	
Diras1	0.099	0.234	1.35	0.71	
Krtap2-4	0.000	0.133	1.81	0.71	
Gsdmc4	1.486	2.940	1.16	0.71	
4930429D17Rik	0.007	0.081	1.82	0.71	
Gas2l2	0.018	0.075	1.60	0.71	
Oca2	0.155	0.290	1.24	0.71	
2610037D02Rik	0.016	0.061	1.52	0.70	
Abhd12b	0.891	1.938	1.26	0.70	
Krtap22-2	0.028	0.348	1.75	0.70	
Itk	0.239	0.543	1.20	0.70	
Akr1c18	29.411	57.364	1.13	0.70	
F830016B08Rik	0.139	0.321	1.32	0.70	
Hspa1l	5.281	11.451	1.14	0.70	
2900052N01Rik	0.012	0.060	1.55	0.70	
Apol10a	0.002	0.036	1.83	0.69	
A930003O13Rik	0.039	0.136	1.47	0.69	
Galnt15	2.460	5.384	1.17	0.69	
Padi2	1.715	3.552	1.13	0.69	
Myl10	2.488	2.598	1.22	0.69	
Try10	0.115	0.371	1.51	0.69	
Areg	0.946	1.801	1.21	0.69	+
Nccrp1	28.107	56.947	1.16	0.69	
Mir155hg	0.051	0.192	1.60	0.69	
Krt85	25.903	47.296	1.16	0.69	
Filip1l	17.920	35.402	1.11	0.68	
Dusp1	47.662	93.158	1.13	0.68	+
Klk6	0.511	1.141	1.23	0.67	

2810442N19Rik	0.152	0.456	1.42	0.67	
Gm4984	0.006	0.094	1.98	0.67	
Gm9696	0.677	1.315	1.16	0.67	
Gm8369	0.125	0.337	1.36	0.67	
Gm9733	0.052	0.285	1.59	0.67	
Il18rap	0.206	0.473	1.18	0.67	
Isg15	9.701	21.016	1.17	0.67	+
Spata21	0.009	0.061	1.65	0.67	
Etv2	0.021	0.126	1.62	0.67	
Tmem74b	0.105	0.307	1.43	0.67	
Gm11568	0.009	0.096	1.72	0.66	
Gm16548	0.784	1.793	1.20	0.66	
Gjd3	0.469	1.053	1.24	0.66	
Arl4d	3.933	8.100	1.08	0.66	
Pou4f3	0.178	0.461	1.34	0.66	
5730522E02Rik	0.046	0.174	1.52	0.66	
Sp100	4.903	10.007	1.14	0.66	
Phf11a	4.408	9.488	1.15	0.66	
Tdgf1	0.017	0.103	1.71	0.66	
Actl7b	0.019	0.124	1.73	0.65	
1810008I18Rik	0.016	0.191	1.83	0.65	
Cdk6	1.058	2.150	1.11	0.65	
Urah	20.862	35.616	1.10	0.65	
Mx1	0.674	1.481	1.14	0.65	+
Slc10a6	4.567	9.451	1.11	0.64	
A2m	0.032	0.085	1.34	0.64	
H2-K2	25.219	50.962	1.06	0.64	
Cd28	0.443	0.914	1.14	0.64	
Fam50b	0.012	0.101	1.76	0.64	
1700003E24Rik	0.000	0.087	1.92	0.64	
Icam5	0.108	0.266	1.30	0.64	
Il6ra	1.909	4.186	1.10	0.64	
Adamts1	28.211	55.069	1.10	0.64	

Trim71	0.005	0.030	1.72	0.63		
Grm3	0.114	0.287	1.26	0.63		
Fgl1	0.734	1.653	1.17	0.63		
Mx2	0.728	1.600	1.18	0.63	+	
Phf11d	6.500	12.962	1.07	0.63		
LOC547349	206.613	407.856	1.06	0.63		
Cyp2c55	0.003	0.044	1.74	0.63		
Lce3b	0.268	0.668	1.40	0.62		
Egr3	6.045	10.913	1.06	0.62		+
Dsg4	0.172	0.355	1.20	0.62		
4933433G15Rik	0.014	0.134	1.69	0.62		
Trim34b	1.075	2.311	1.12	0.62		
Krtap26-1	0.044	0.226	1.73	0.62		
Mrgpra4	0.043	0.147	1.45	0.61		
Ecel1	0.090	0.229	1.28	0.61		
BC117090	1201.550	#######	1.03	0.61		
Tmem74	0.007	0.075	1.72	0.61		
Hoxd1	0.234	0.497	1.16	0.61		
Clec4n	1.810	3.788	1.07	0.61		
Edn1	4.824	9.749	1.06	0.60		+
Rgs20	0.319	0.666	1.13	0.60		
Prl8a9	0.023	0.142	1.64	0.60		
LOC100503496	3.742	7.767	1.09	0.60		
Lmod2	13.820	24.422	1.08	0.60		
Skint4	0.417	0.805	1.11	0.60		
Krt27	26.580	41.652	1.03	0.60		
Timd4	0.655	1.125	1.13	0.60		
Krtap1-3	0.059	0.265	1.52	0.60		
Dear1	0.942	2.141	1.15	0.60		
Foxi3	2.136	3.928	1.06	0.59		
Gm5538	0.070	0.203	1.45	0.59		
E130112N10Rik	0.099	0.277	1.44	0.59		

BC061195	0.000	0.077	1.81	0.59	
Rtn4rl2	0.502	1.117	1.19	0.59	
9930111J21Rik2	0.461	0.931	1.06	0.59	
Cass4	0.503	1.087	1.12	0.59	
Cd244	0.066	0.164	1.29	0.59	
Oasl2	4.775	8.903	1.00	0.59	
Fcrlb	0.013	0.103	1.78	0.59	
Rnase1	2.633	4.543	1.05	0.59	
Arl14epl	0.010	0.059	1.59	0.59	
Lypd3	26.857	46.182	1.04	0.58	
Dnajb13	0.273	0.636	1.23	0.58	
Sprr2d	3.806	4.465	1.10	0.58	
Mt2	144.678	238.984	1.04	0.58	
2210011C24Rik	0.273	0.656	1.28	0.58	
Mir8101	2.955	6.750	1.12	0.58	
Csf3r	0.349	0.637	1.06	0.58	
Try4	0.128	0.414	1.34	0.58	
Gm12250	0.515	1.036	1.08	0.58	
Krt81	12.531	21.668	1.07	0.58	
Mir145a	0.384	1.889	1.57	0.57	
Pdzk1ip1	6.191	12.257	1.04	0.57	
Elane	0.520	1.054	1.18	0.57	
Ifi204	13.963	27.566	1.00	0.57	
Flt3	0.215	0.440	1.09	0.56	
Krt35	20.508	31.518	0.96	0.56	
Btg2	26.840	51.126	1.05	0.56	+
Ltb	1.046	2.113	1.03	0.56	
Pax1	0.693	1.128	1.10	0.56	
Lce1g	31.502	54.850	0.97	0.56	
Gpnmb	1.947	3.319	1.06	0.56	
Sell	0.496	0.940	1.08	0.56	
Otud1	5.098	10.073	1.02	0.56	
Aqp3	15.505	26.960	1.01	0.56	
Rhox3h	0.189	0.460	1.28	0.55	

Pax5	0.009	0.109	1.69	0.55		
Cfc1	0.000	0.085	1.87	0.55		
Tnfrsf14	0.674	1.333	1.09	0.55		
Scimp	0.122	0.376	1.36	0.55		
Arg2	5.631	9.082	0.99	0.55		
Gm5478	21.467	35.503	0.99	0.55		
Fbxo32	2.770	5.016	0.98	0.55		
Gabre	0.769	1.571	1.01	0.55		
Gm6904	2.335	4.687	1.04	0.55		
Ikzf3	0.085	0.183	1.15	0.55		
Ctxn3	0.577	1.153	1.06	0.54		
Dsg1a	15.547	26.614	1.04	0.54		
Rnf39	2.666	4.953	0.99	0.54		
Ren2	0.116	0.289	1.22	0.54		
E030044B06Rik	0.049	0.200	1.48	0.54		
Ackr1	3.714	7.440	1.01	0.54		
Rgs16	4.334	8.286	0.99	0.54		
Ly6g6c	46.397	78.204	0.96	0.54		
Arid5b	4.871	9.169	0.96	0.54	+	
Mir7020	0.461	2.204	1.65	0.54		
Osm	1.198	1.941	1.02	0.54		
Vmn1r195	0.074	0.223	1.40	0.54		
Def6	7.098	14.090	1.08	0.53		
Krt82	0.211	0.453	1.09	0.53		
Slc16a6	3.154	6.227	1.04	0.53		+
Nyap2	0.003	0.027	1.66	0.53		
Dmkn	111.798	179.187	0.97	0.53		
Ccr4	0.149	0.300	1.13	0.53		
Lce1f	39.962	71.147	0.96	0.53		
Hist1h1c	94.912	165.070	0.95	0.53		
Akr1c20	1.050	2.025	1.03	0.53		
C1ra	26.759	49.465	0.99	0.53		
H2-Oa	0.190	0.462	1.20	0.52		
Spink5	4.504	7.593	0.95	0.52		
Ifi203	3.266	6.085	0.95	0.52		

Dct	30.338	45.931	1.00	0.52		
Serpina9	1.419	2.719	0.94	0.52		
Ren1	0.125	0.310	1.26	0.52		
Rn4.5s	1.038	2.001	1.01	0.52		
Scarna10	0.227	1.552	1.52	0.52		
Ifi44	1.938	3.690	0.96	0.52	+	
Otos	0.076	0.282	1.47	0.52		
Ifit1bl2	0.105	0.223	1.20	0.52		
Phf11b	6.768	13.434	1.02	0.52		
Gm12505	0.014	0.146	1.53	0.52		
Gast	0.272	0.775	1.37	0.52		
Atcay	0.209	0.425	1.10	0.52		
Wdr54	1.023	2.017	1.00	0.51		
Klk4	0.048	0.160	1.44	0.51		
Phf11c	4.660	8.565	0.92	0.51		
Trim63	4.881	8.097	0.97	0.51		
Gsdma3	1.216	1.984	1.00	0.51		
Calca	4.431	8.185	1.00	0.51		
Slc18a3	0.102	0.201	1.17	0.51		
Gm694	0.065	0.243	1.44	0.51		
C330022C24Rik	2.162	3.703	1.01	0.51		
Gbp6	4.688	7.837	1.01	0.51	+	
Slc4a10	0.025	0.068	1.26	0.51		
Sgol2b	0.158	0.318	1.06	0.51		
Mgarp	1.328	2.652	1.02	0.50		
Ccl12	4.012	7.400	0.98	0.50		
Gm8633	0.137	0.440	1.41	0.50		
Olfr222	0.000	0.092	1.69	0.50		
Gm572	0.307	0.605	1.04	0.50		
Tmprss4	5.602	9.710	0.92	0.50		
Fcgr2b	13.760	26.751	0.97	0.50		
Xdh	4.577	8.716	0.97	0.50		
Gm15284	0.000	0.226	1.72	0.50		
Gfpt2	9.714	18.095	0.93	0.50		+
Adm	6.179	11.552	0.94	0.50		

C1rb	15.862	29.320	0.98	0.49		
Gch1	0.943	1.762	0.91	0.49	+	+
Kcnv2	0.005	0.026	1.53	0.49		
H2-T3	1.308	2.469	0.96	0.49		
Clec7a	0.262	0.541	1.03	0.49		
Ddx60	0.388	0.721	0.95	0.49	+	
Tacstd2	25.582	45.495	0.95	0.49		
Gm20750	0.000	0.025	1.61	0.49		
Nkx2-2os	0.004	0.030	1.63	0.49		
Sh2d1b1	0.090	0.246	1.35	0.49		
Sult2a6	0.006	0.091	1.61	0.49		
Tnfaip6	21.896	39.777	0.86	0.49	+	+
Klf4	53.580	97.459	0.95	0.48		+
Slc16a9	0.598	1.088	0.96	0.48		
Sult2a4	0.006	0.091	1.67	0.48		
Ifi205	17.254	33.552	1.03	0.48		
Lrat	1.377	2.406	0.94	0.48		
Vwf	5.069	9.546	0.92	0.48		
F630111L10Rik	0.001	0.012	1.65	0.48		
Mt1	117.514	191.572	0.88	0.48		
8430436N08Rik	0.160	0.389	1.17	0.48		
Amdhd1	0.049	0.137	1.21	0.48		
Csf2rb	10.171	18.674	0.91	0.48	+	
Irs2	3.852	7.316	0.94	0.48		+
Trim29	37.206	59.380	0.89	0.48		
Htr5b	0.046	0.132	1.30	0.48		
Vmn2r1	0.022	0.080	1.40	0.47		
Dusp10	6.455	11.904	0.96	0.47		
Fam166b	0.195	0.441	1.10	0.47		
Klk5	17.619	27.496	0.93	0.47		
Cysltr2	0.155	0.318	1.07	0.47		
Lgals3bp	29.840	51.888	0.89	0.47	+	
Cdsn	29.843	46.384	0.91	0.47		
Sbk3	0.032	0.084	1.24	0.47		

Gm20337	0.259	0.540	1.05	0.47		
Gbp10	3.208	5.353	0.92	0.47		
Cfb	3.570	6.482	0.92	0.47	+	
Serpina3h	6.412	10.765	0.90	0.47		
Xylt1	0.538	0.969	0.91	0.46		
Tnfrsf17	0.344	0.760	1.16	0.46		
Dnah6	0.033	0.068	1.10	0.46		
Gem	10.615	19.507	0.91	0.46		+
P2rx7	2.365	4.392	0.91	0.46		
Ubd	0.597	1.105	1.05	0.46		
Irf8	2.133	3.755	0.88	0.46	+	
Mir6983	1.008	3.099	1.35	0.46		
Fcer1a	1.860	2.835	0.94	0.46		
Padi3	0.843	1.350	0.93	0.46		
Olfr109	0.000	0.093	1.71	0.46		
Pkdrej	0.113	0.178	1.00	0.45		
Slamf7	0.061	0.127	1.10	0.45	+	
Dnase2a	15.931	26.569	0.88	0.45		
Krt75	18.234	28.448	0.90	0.45		
4930546K05Rik	0.014	0.056	1.42	0.45		
Mybpc2	21.207	28.001	0.90	0.45		
Tyr	1.793	2.532	0.92	0.45		
Dsp	24.372	39.174	0.88	0.45		
Tap1	7.289	13.341	0.93	0.45	+	+
Skint6	0.835	1.423	0.93	0.45		
Amy1	3.199	4.281	0.92	0.45		
Il1r2	1.653	3.101	0.96	0.45		
Nefh	0.059	0.138	1.08	0.45		
Cldn13	0.010	0.082	1.67	0.45		
Cldn1	13.905	22.750	0.91	0.45		
Klf6	36.097	66.374	0.94	0.45		+
Cts6	0.049	0.150	1.39	0.45		
Gm5105	0.549	0.993	0.95	0.45		
Dscam	0.163	0.277	0.93	0.44		
Speer1	0.000	0.070	1.59	0.44		

Fam83g	2.870	4.388	0.88	0.44		
Zcchc16	0.077	0.158	1.14	0.44		
Prss28	0.086	0.185	1.28	0.44		
Ccl6	3.639	6.414	0.87	0.44		
Adap2	0.731	1.407	0.97	0.44		
Dsc1	4.255	6.510	0.88	0.43		
Psmb9	4.376	8.257	0.92	0.43	+	
Rnase2b	1.141	1.771	0.97	0.43		
4933402P03Rik	0.007	0.055	1.53	0.43		
Gm648	0.010	0.106	1.59	0.43		
Cldn19	0.265	0.411	0.90	0.43		
Oas1f	2.710	4.422	0.93	0.43		
S100g	1.344	1.882	1.02	0.43		
Nceh1	1.856	3.455	0.97	0.43		
A330069E16Rik	0.663	1.383	1.04	0.43		
Fgl2	7.236	13.687	0.94	0.43	+	
Gnao1	1.270	2.237	0.88	0.43		
Tns4	2.451	4.009	0.83	0.43		
Cd4	0.709	1.317	0.93	0.42		
Prmt8	0.468	0.749	0.98	0.42		
Rasgef1a	0.299	0.565	0.99	0.42		
Zc3h12a	1.487	2.702	0.87	0.42		+
Krtap24-1	0.021	0.084	1.40	0.42		
Adrb2	3.998	7.041	0.89	0.42		
Slitrk3	0.288	0.602	1.00	0.42		
4930486L24Rik	0.349	0.690	0.99	0.42		
Oas3	0.323	0.612	1.02	0.42	+	
Pyhin1	0.746	1.374	0.92	0.42		
Ccdc155	0.202	0.379	1.01	0.42		
4833422M21Rik	0.055	0.161	1.27	0.42		
Csrnp1	6.407	11.155	0.88	0.42		
Tox2	0.806	1.526	0.92	0.42		

Kcnc1	0.308	0.440	0.90	0.42
Jund	114.275	194.003	0.89	0.42
Adamtsl3	0.523	0.917	0.91	0.42
Aqp9	0.510	0.963	0.92	0.41
Tmprss3	0.017	0.069	1.41	0.41
Galr3	0.017	0.106	1.45	0.41
Tuba8	12.698	21.205	0.89	0.41
Hmgcs2	3.710	6.389	0.86	0.41
Ctgf	12.144	21.449	0.88	0.41
2900092D14Rik	0.038	0.093	1.30	0.41
Slc2a1	31.644	55.004	0.87	0.41
Efcab10	0.024	0.196	1.53	0.41
Tlr5	1.183	2.184	0.89	0.41
Aph1c	1.459	2.635	0.85	0.41
Sun3	0.070	0.186	1.16	0.41
Lce1l	15.681	27.068	0.88	0.41
Olfr543	0.038	0.117	1.32	0.41
Raet1d	2.504	4.681	0.86	0.41
Krt2	2.671	4.351	0.88	0.41
Cytip	0.713	1.249	0.89	0.41
Lce1k	11.127	18.147	0.80	0.41
Dusp8	3.931	7.083	0.87	0.40
AI115009	0.026	0.083	1.40	0.40
AI607873	6.323	11.034	0.84	0.40
Gm10354	0.602	1.109	1.05	0.40
Celrr	0.020	0.084	1.44	0.40
Cyp1b1	1.294	2.376	0.88	0.40
Adora2b	1.268	2.342	0.90	0.40
Trim30d	0.912	1.589	0.88	0.40
Ly6d	93.738	143.088	0.84	0.40
Gbp8	1.601	2.653	0.86	0.40
Rhox3c	0.220	0.451	1.22	0.40
4930447M23Rik	0.212	0.396	1.03	0.40
Ang5	0.235	0.560	1.11	0.40

Gpr21	0.288	0.566	0.94	0.39
Skint3	0.515	0.866	0.90	0.39
Gm5640	0.370	0.555	0.93	0.39
Fbxo43	0.069	0.147	1.07	0.39
Mc3r	0.117	0.244	1.05	0.39
Nt5c1b	0.020	0.083	1.41	0.39
H2-Q6	28.610	50.743	0.89	0.39
Kcnj4	0.150	0.317	1.06	0.39
Them5	25.482	41.006	0.90	0.39
Errfi1	16.460	28.381	0.85	0.39
Cyp2d22	6.475	11.248	0.86	0.39
4833423E24Rik	1.583	2.720	0.87	0.39
Zfp750	9.193	15.472	0.85	0.39
Rab20	1.378	2.533	0.92	0.39
1700120E14Rik	0.000	0.046	1.70	0.39
Fam83c	1.111	1.858	0.86	0.39
Krt86	10.902	17.111	0.86	0.39
Ankrd22	1.373	2.224	0.85	0.39
H2-Q8	27.777	49.259	0.88	0.39
Slco1a4	0.008	0.035	1.39	0.39
Akr1c6	1.092	1.946	0.95	0.38
Blnk	1.041	1.839	0.85	0.38
Fgf21	0.182	0.372	1.19	0.38
Acpp	2.540	4.143	0.85	0.38
Fam71a	0.010	0.059	1.47	0.38
Tprg	4.142	6.505	0.84	0.38
Scn9a	0.057	0.113	1.00	0.38
Pvrl4	6.557	10.829	0.85	0.38
Oas1e	0.085	0.206	1.18	0.38
Art4	1.367	2.320	0.88	0.38
1700034H15Rik	0.301	0.541	0.89	0.38
Siglec1	1.810	2.884	0.80	0.38
Acot12	0.018	0.058	1.32	0.38

Pou3f3	0.281	0.460	0.97	0.38	
Slc6a20a	0.102	0.209	1.14	0.38	
Gapt	0.078	0.182	1.11	0.38	
Dnah7b	0.180	0.286	0.88	0.38	
Rab11fip4	0.258	0.462	0.91	0.38	
1700025N23Rik	0.012	0.098	1.48	0.38	
Mndal	19.624	31.737	0.81	0.38	
Folh1	0.159	0.289	0.99	0.38	
Bdkrb2	2.880	5.142	0.86	0.37	
Sncb	0.270	0.516	0.98	0.37	
Plag1	1.096	1.978	0.86	0.37	
4933417O13Rik	0.166	0.332	0.98	0.37	
Gm10318	0.087	0.261	1.29	0.37	
Stx19	0.905	1.669	0.90	0.37	
Dnajb8	0.033	0.156	1.43	0.37	
Il4i1	0.570	1.120	1.02	0.37	
Rhbg	2.573	4.103	0.80	0.37	
Egln3	21.370	36.767	0.85	0.37	
Nlrc5	0.396	0.631	0.83	0.37	+
Ankrd55	0.592	1.066	0.86	0.37	
Kcne1	0.070	0.140	1.04	0.37	
Olfr1033	0.484	0.789	0.89	0.37	
Gm17019	0.519	0.884	0.96	0.37	
Ankrd23	5.957	9.721	0.81	0.37	
Ccr9	0.058	0.110	1.12	0.37	
Ifi44l	0.008	0.068	1.53	0.37	+
Krt34	8.841	13.496	0.80	0.37	
Slc34a2	0.886	1.468	0.88	0.37	
Wfdc17	1.605	2.772	0.90	0.37	
Mir6899	0.840	2.754	1.30	0.37	
Rnd1	0.547	0.980	0.86	0.37	
Tmem215	0.137	0.266	0.93	0.37	
1700001L05Rik	0.252	0.455	0.92	0.36	

Myc	13.380	22.578	0.82	0.36		+
H1f0	259.390	423.587	0.82	0.36		
Akr1c14	11.517	17.908	0.83	0.36		
Gm20594	0.773	1.583	1.07	0.36		
Dsc3	15.006	23.350	0.84	0.36		
Mir431	15.421	21.794	0.85	0.36		
2010107G12Rik	0.182	0.357	0.97	0.36		
Trim34a	2.474	4.441	0.87	0.36		
Ackr2	0.316	0.571	0.90	0.36		
C2	6.993	11.893	0.80	0.36		
Ptgs2	0.494	0.882	0.88	0.36	+	+
AU022754	0.056	0.133	1.14	0.36		
Cited2	24.464	39.796	0.77	0.35		
4933406C10Rik	0.227	0.445	0.98	0.35		
Klf9	8.532	14.216	0.83	0.35		+
Adgrf4	2.372	3.769	0.77	0.35		
Irgm1	6.412	10.914	0.81	0.35		
Plac8	19.321	28.684	0.79	0.35		
Dusp15	0.032	0.079	1.22	0.35		
Prss50	0.081	0.197	1.15	0.35		
Bdnf	0.488	0.811	0.78	0.35		
Adgrl4	5.935	9.209	0.74	0.35		
1700065D16Rik	0.043	0.203	1.43	0.35		
Mir421	0.000	0.870	1.67	0.34		
Gpcpd1	6.964	11.539	0.79	0.34		
Ang3	0.239	0.569	1.16	0.34		
Prss51	0.140	0.299	1.16	0.34		
Kcna1	0.447	0.744	0.81	0.34		
Lce1e	34.881	54.563	0.79	0.34		
Unc13c	0.062	0.115	0.96	0.34		
Ndufa4l2	122.485	196.305	0.75	0.34		
Ackr3	11.767	19.850	0.79	0.34		+
Zfp109	0.875	1.604	0.87	0.34		

Gm4070	4.853	7.346	0.74	0.34		
AU015836	0.011	0.044	1.35	0.34		
Ifih1	1.605	2.715	0.82	0.34	+	+
Stfa3	1071.06<br>0	#######	0.81	0.34		
Mxi1	8.768	14.636	0.80	0.33		
Gsdma2	2.298	3.479	0.84	0.33		
Gpr27	1.292	2.455	0.90	0.33		
Lce1h	25.292	38.984	0.77	0.33		
Itgam	1.079	1.864	0.77	0.33		
Gpr132	0.260	0.461	0.86	0.33		
Ly6g6e	3.859	6.473	0.80	0.33		
Traf1	0.948	1.706	0.83	0.33		+
Perp	181.226	274.750	0.81	0.33		
Msl3l2	2.761	4.407	0.75	0.33		
Atp6v1c2	4.084	6.665	0.78	0.33		
Tgm7	0.066	0.145	1.08	0.33		
Vmn2r-ps54	0.118	0.224	0.92	0.33		
DQ267102	0.833	2.483	1.23	0.33		
Anxa8	27.685	46.276	0.82	0.33		
Gbp2b	0.084	0.172	1.02	0.33		
S1pr1	16.221	26.415	0.75	0.33		
Flt1	4.437	7.070	0.74	0.33		
Cyp17a1	1.504	2.418	0.82	0.32		
Olfr1417	0.000	0.070	1.59	0.32		
Gm19345	0.048	0.155	1.28	0.32		
DQ267100	1.363	3.414	1.18	0.32		
Irf4	3.512	4.522	0.76	0.32	+	
Dpy19l2	0.006	0.032	1.34	0.32		
Trib1	7.148	11.943	0.79	0.32		+
Syne3	2.904	4.611	0.77	0.32		
Amy2a5	0.242	0.349	0.86	0.32		
Sox21	4.193	6.256	0.76	0.32		
Gm15987	0.129	0.285	1.08	0.32		
Corin	1.555	1.992	0.77	0.32		
Stk17b	8.446	14.090	0.79	0.32		

Htr2a	0.317	0.549	0.85	0.32		
Xaf1	2.194	3.664	0.78	0.32	+	
A630019I02Rik	0.996	1.120	0.82	0.32		
Gbp4	1.639	2.525	0.76	0.32	+	
Clca3a1	5.937	10.083	0.77	0.32		
Hrnr	58.023	66.142	0.73	0.32		
Vdr	6.965	9.388	0.74	0.32		
Alox12b	9.782	15.107	0.76	0.32		
1500026H17Rik	0.099	0.203	0.99	0.32		
Gpr183	1.696	3.011	0.81	0.31		+
Sost	2.170	3.413	0.74	0.31		
Arid5a	2.399	3.944	0.79	0.31		
5930430L01Rik	0.649	0.984	0.80	0.31		
Krtap10-4	0.066	0.195	1.25	0.31		
Pik3ap1	1.756	2.756	0.80	0.31		
Asb16	0.916	1.472	0.82	0.31		
Trim69	0.043	0.100	1.25	0.31		
Slco2b1	6.603	10.717	0.74	0.31		
Spef2	0.025	0.049	1.04	0.31		
Syk	2.597	4.364	0.74	0.31		
Mafb	18.648	29.156	0.79	0.31		
Asprv1	264.758	385.560	0.76	0.31		
Hcar2	9.790	15.426	0.75	0.31		
Iigp1	3.590	5.736	0.80	0.31		
Dmrtb1	0.045	0.123	1.21	0.31		
Tcp11l2	10.245	16.940	0.76	0.30		
Trim25	8.023	13.163	0.76	0.30	+	
Mir196b	0.253	1.296	1.38	0.30		
H2-Q1	14.095	22.645	0.75	0.30		
Mbip	6.247	10.243	0.77	0.30		
B4galnt3	0.378	0.603	0.81	0.30		
Pou2f3	8.639	13.508	0.78	0.30		
H2-M9	0.256	0.535	0.96	0.30		
Krt33b	7.196	10.746	0.77	0.30		

Rpl10l	0.936	1.336	0.84	0.30	
Pvalb	4.063	4.175	0.75	0.30	
BC094916	0.378	0.562	0.88	0.30	
Serpina3f	0.559	0.991	0.84	0.30	
Mill2	2.559	4.151	0.77	0.30	
Pappa2	6.071	9.423	0.74	0.30	
Gm4787	0.237	0.406	0.93	0.30	
Hoxb5os	1.734	2.910	0.81	0.30	
Aox4	3.245	4.131	0.74	0.30	
Cd7	0.414	0.826	0.90	0.29	
Clca3a2	5.188	8.111	0.80	0.29	
Ccrl2	0.528	0.813	0.78	0.29	+
H2-Q9	17.848	28.251	0.72	0.29	
Med26	5.324	8.426	0.71	0.29	
Hsd17b13	0.104	0.207	1.01	0.29	
Acsbg1	6.863	10.855	0.76	0.29	
Klf2	26.925	42.530	0.72	0.29	+
Gvin1	4.851	7.344	0.73	0.29	
Psg16	0.005	0.039	1.53	0.29	
2310081J21Rik	1.739	2.525	0.85	0.29	
Klhl38	1.568	2.202	0.76	0.29	
Havcr2	0.312	0.558	0.83	0.29	
Slc23a1	0.077	0.154	1.03	0.29	
5430421N21Rik	8.366	12.379	0.80	0.29	
Kcnk15	0.026	0.107	1.35	0.29	
Amy2a4	0.103	0.148	0.86	0.29	
Mir7676-2	9.279	17.060	0.83	0.29	
Akr1c21	0.943	1.513	0.78	0.29	
Abca12	0.979	1.295	0.73	0.29	
4930558J18Rik	0.532	0.902	0.79	0.29	
Rnf19b	8.900	14.151	0.73	0.29	+
Acer1	3.635	5.189	0.72	0.29	
Mcoln3	1.197	1.712	0.75	0.28	

Trpm1	0.997	1.512	0.75	0.28		
Trpm2	0.140	0.257	0.84	0.28		
Klrb1c	0.110	0.209	0.91	0.28		
Tssk1	0.089	0.197	1.07	0.28		
Platr17	0.306	0.546	0.94	0.28		
H2-L	231.057	378.169	0.76	0.28		
Prkg2	0.554	0.896	0.74	0.28		
Slc16a3	20.078	33.160	0.75	0.28		
Cd6	0.028	0.065	1.17	0.28		
Plekho2	6.399	10.264	0.73	0.28		
Umod	0.133	0.251	0.94	0.28		
Hs6st3	0.057	0.136	1.14	0.28		
D930028M14Rik	0.040	0.123	1.21	0.28		
Nr4a3	0.895	1.282	0.76	0.28		+
Lor	638.332	850.436	0.72	0.28		
5430403N17Rik	0.019	0.060	1.29	0.28		
Kcnk7	2.647	3.926	0.77	0.28		
Amy2a3	0.103	0.148	0.85	0.28		
Amy2a2	0.103	0.148	0.82	0.28		
Sfn	127.104	187.228	0.73	0.28		
Slc39a12	0.062	0.132	1.04	0.28		
Tmevpg1	0.000	0.059	1.65	0.28		
Trim30a	2.283	3.618	0.73	0.28		
Ptafr	0.677	1.126	0.76	0.27		
Igbp1b	0.036	0.130	1.36	0.27		
Nxph4	2.112	3.565	0.73	0.27		
Ifitm3	213.724	347.028	0.73	0.27	+	
S100a7a	0.668	1.160	0.90	0.27		
Ctnna3	0.500	0.803	0.79	0.27		
Gda	1.400	2.392	0.77	0.27		
Ndrg1	28.121	46.511	0.75	0.27		
Sik1	16.195	25.227	0.71	0.27		+
Itgax	0.130	0.224	0.82	0.27		
Dusp6	18.418	29.071	0.71	0.27		

Lrriq3	0.162	0.274	0.92	0.27	
Tgm1	6.573	9.852	0.76	0.27	
Pmaip1	2.289	3.678	0.71	0.27	
Skint7	0.467	0.749	0.80	0.27	
Kcnh8	0.018	0.062	1.25	0.27	
Galnt9	2.156	3.220	0.71	0.27	
Gjb2	11.415	15.968	0.68	0.27	
Ptk6	0.261	0.472	0.83	0.27	
Rtp4	7.865	12.020	0.70	0.27	+
AW011738	0.752	1.252	0.76	0.27	
Ccl3	1.123	1.757	0.78	0.27	
Trim66	0.032	0.062	0.89	0.27	
Tuba3b	0.089	0.183	1.04	0.27	
Magel2	1.714	2.637	0.76	0.27	
Nol4	0.025	0.053	1.07	0.27	
BC027072	0.000	0.013	1.56	0.27	
Hesx1	0.095	0.233	1.14	0.27	
Atp6v0d2	0.052	0.123	1.08	0.26	
Klk7	37.039	53.151	0.69	0.26	
Cfd	585.585	528.048	0.70	0.26	
Ggn	0.357	0.598	0.81	0.26	
Gjb5	3.591	4.940	0.71	0.26	
Tlx3	0.042	0.115	1.29	0.26	
Nptx1	0.231	0.388	0.75	0.26	
Clip4	2.996	4.780	0.72	0.26	
1700092K14Rik	0.109	0.391	1.29	0.26	
4930444M15Rik	0.003	0.053	1.59	0.26	
Ezh1	6.293	10.099	0.72	0.26	
Tnfrsf13c	0.130	0.266	1.03	0.26	
Scel	4.947	7.860	0.70	0.26	
Chl1	1.176	1.905	0.72	0.26	
H2-D1	182.467	290.678	0.69	0.26	
Pgpep1l	0.490	0.877	0.91	0.26	
Bst1	0.228	0.415	0.84	0.26	

C130026I21Rik	1.348	2.012	0.71	0.25	
Klf3	11.613	17.655	0.64	0.25	
Aurkc	0.034	0.107	1.21	0.25	
Gjb4	3.870	5.505	0.75	0.25	
Il1f8	2.518	3.626	0.77	0.25	
Fzd5	1.537	2.480	0.74	0.25	
Pim3	22.754	34.670	0.68	0.25	
Tnfrsf11b	1.813	3.011	0.72	0.25	
9430083A17Rik	0.338	0.560	0.77	0.25	
Ccdc64b	2.470	3.864	0.71	0.25	
Nipal4	3.051	4.273	0.72	0.25	
1700029M20Rik	0.025	0.067	1.15	0.25	
Gm10677	0.744	1.195	0.72	0.25	
Spag6l	0.016	0.067	1.37	0.25	
Adam2	0.036	0.086	1.08	0.25	
Nrip1	3.179	4.802	0.69	0.25	
Gm7030	15.646	24.536	0.67	0.25	
Il17ra	3.760	5.745	0.70	0.25	
Hist1h1d	11.363	17.890	0.70	0.25	
Slc39a8	4.795	6.989	0.71	0.25	
Il33	1.853	2.811	0.67	0.25	
H2-Q7	17.674	27.975	0.69	0.25	
Fam3b	1.017	1.062	0.89	0.25	
Serping1	32.556	50.379	0.66	0.25	+
Hist1h1e	12.190	18.570	0.69	0.25	
Tgm5	2.479	3.735	0.74	0.25	
Adgrf2	0.195	0.342	0.88	0.25	
Nipal3	1.877	2.947	0.67	0.25	
Nell1	1.438	2.273	0.70	0.25	
H2-M10.5	1.107	1.576	0.71	0.25	
2810433D01Rik	0.798	1.371	0.75	0.24	
Abca6	0.870	1.437	0.72	0.24	
Efcab5	0.075	0.133	0.86	0.24	

Cdh26	0.175	0.288	0.82	0.24	
Ccl9	3.335	5.472	0.69	0.24	
Efcab9	0.035	0.144	1.32	0.24	
Gm5544	0.072	0.198	1.13	0.24	
Cacna2d4	0.000	0.011	1.53	0.24	
Grhl1	8.114	12.622	0.71	0.24	
Tmem252	7.849	11.895	0.68	0.24	
Rasgef1b	6.656	9.701	0.65	0.24	
AA986860	3.303	4.924	0.69	0.24	
Defb1	5.494	8.492	0.76	0.24	
Ltb4r1	1.503	2.454	0.74	0.24	
Kcnj15	0.823	1.258	0.72	0.24	
Gm11710	6.860	10.394	0.66	0.24	
Pik3c2g	0.034	0.067	0.89	0.24	
Il7r	0.685	1.140	0.74	0.24	+
Pmel	20.020	25.036	0.71	0.24	
Olig1	0.079	0.142	0.99	0.24	
Gucy2e	0.074	0.124	0.79	0.24	
Dyrk2	7.198	11.240	0.69	0.24	
Cd3e	0.198	0.384	0.87	0.23	
Wdr31	0.595	0.948	0.76	0.23	
Spry1	30.367	47.112	0.67	0.23	
Ace	4.695	7.369	0.68	0.23	
Dkk1	6.071	8.964	0.66	0.23	
Gbp2	3.828	5.655	0.70	0.23	
Cbx7	1.554	2.437	0.68	0.23	
Spidr	4.292	6.461	0.65	0.23	
Flcn	9.166	14.614	0.70	0.23	
Lbp	4.016	6.240	0.66	0.23	
Lce1d	78.747	113.786	0.65	0.23	
Enpp6	0.033	0.072	1.03	0.23	
Tekt5	1.388	1.958	0.72	0.23	
Elf3	4.014	1.796	0.73	0.23	
C1s2	6.558	10.190	0.75	0.23	
Ak5	0.875	1.413	0.70	0.23	
Cpne4	0.306	0.446	0.75	0.23	

Gm960	0.045	0.117	1.19	0.23
H2-Q5	6.242	9.833	0.73	0.23
Ccdc110	0.088	0.176	0.96	0.23
Hhat	2.223	3.459	0.67	0.23
Sptb	2.418	3.326	0.68	0.23
2610528A11Rik	17.041	16.527	0.63	0.22
Ptch1	10.669	15.279	0.67	0.22
C920025E04Rik	25.611	38.926	0.66	0.22
Krt32	1.514	2.265	0.78	0.22
Nppb	0.034	0.141	1.23	0.22
Snord57	1.792	3.848	1.08	0.22
Mir127	9.294	14.481	0.78	0.22
Mylip	14.688	22.989	0.67	0.22
Gm11992	0.742	1.111	0.68	0.22
Ly6i	5.383	8.433	0.69	0.22
C330013E15Rik	0.729	1.180	0.78	0.22
Pcsk5	2.514	3.762	0.66	0.22
Hsd11b1	2.084	3.263	0.66	0.22
Gsdma	9.146	13.136	0.71	0.22
Pkp1	44.168	62.958	0.70	0.22
Haao	0.395	0.702	0.86	0.22
Cacna2d2	0.662	1.026	0.73	0.22
Cst6	2.633	4.071	0.70	0.22
Lce1a2	110.895	156.099	0.66	0.22
Oas1d	0.071	0.152	1.05	0.22
Raet1b	2.567	4.347	0.75	0.22
4933433C11Rik	0.000	0.046	1.51	0.22
Cd19	0.084	0.181	0.94	0.22
1810006J02Rik	0.096	0.209	1.04	0.21
Rimbp3	0.039	0.075	0.97	0.21
Pla2g7	8.398	12.842	0.60	0.21

Xkrx	2.758	3.880	0.66	0.21		
Pinlyp	4.198	5.071	0.69	0.21		
Cybb	1.438	2.306	0.69	0.21		
Sec14l4	0.274	0.404	0.78	0.21		
Tmem71	0.338	0.541	0.75	0.21		
3425401B19Rik	2.898	3.710	0.65	0.21		
Ly9	0.334	0.561	0.74	0.21		
Stac2	0.515	0.795	0.67	0.21		
Fpr2	0.519	0.901	0.79	0.21		
Mboat4	0.050	0.120	1.09	0.21		
Lrrc8c	4.797	7.408	0.64	0.21		
Gm11937	0.000	0.167	1.40	0.21		
Pik3ip1	7.642	11.661	0.68	0.21		
Ly75	0.074	0.132	0.80	0.21		
2410004P03Rik	0.132	0.245	0.98	0.21		
Lrrc8e	1.259	2.017	0.73	0.21		
B430319G15Rik	0.555	0.883	0.68	0.21		
Lgmn	44.764	66.566	0.60	0.21		
Tnfsf10	0.847	1.266	0.67	0.21	+	
Acss3	1.285	1.682	0.67	0.21		
Ccdc113	0.233	0.416	0.87	0.21		
Popdc2	4.028	5.519	0.62	0.21		
Mbp	3.864	5.927	0.66	0.21		
Jup	35.077	51.823	0.70	0.21		
Gm4841	0.036	0.085	1.06	0.21		
Fosl1	0.643	1.073	0.71	0.21		+
Kctd11	8.030	12.337	0.66	0.21		
Tcf23	0.077	0.129	0.85	0.21		
4930426L09Rik	0.344	0.549	0.77	0.21		
Ms4a4c	1.139	1.829	0.68	0.20		
Tgm3	4.393	4.754	0.64	0.20		
Ang6	0.000	0.093	1.52	0.20		

Nlrp10	3.175	4.753	0.67	0.20	
Gm11711	6.860	10.394	0.67	0.20	
Crim1	5.832	8.697	0.63	0.20	
Acan	0.543	0.821	0.67	0.20	
C1s1	27.215	41.274	0.70	0.20	
BC100530	2317.980	#######	0.62	0.20	
Pramef12	0.737	1.030	0.74	0.20	
Foxd1	1.617	2.597	0.67	0.20	
H2-Q2	15.979	25.083	0.67	0.20	
Rnase2a	0.062	0.192	1.13	0.20	
Klk9	7.389	10.278	0.67	0.20	
Fam26e	3.456	5.416	0.66	0.20	
Il6st	27.588	40.377	0.62	0.20	+
Hhip	3.372	5.013	0.64	0.20	
Slfn8	0.928	1.467	0.68	0.20	
Pnpla7	2.352	3.712	0.64	0.20	
Odc1	91.417	132.811	0.60	0.20	
Sphk1	3.974	5.710	0.69	0.20	+
Mir7676-1	10.233	17.060	0.74	0.20	
2010106E10Rik	0.201	0.360	0.88	0.20	
Lrrc18	0.315	0.568	0.80	0.20	
Tppp	3.556	5.377	0.65	0.20	
Fosl2	20.744	32.326	0.67	0.19	+
Adam3	0.052	0.106	0.96	0.19	
Cdnf	0.609	0.958	0.70	0.19	
Gpx3	15.290	23.387	0.65	0.19	
Rsph4a	0.047	0.085	0.98	0.19	
Cfhr2	1.504	2.325	0.66	0.19	
Mir1199	0.000	0.370	1.37	0.19	
Dtx3l	2.413	3.492	0.62	0.19	
Pstpip1	1.833	2.774	0.63	0.19	
Prtg	0.121	0.204	0.75	0.19	
Wfdc12	0.645	1.064	0.78	0.19	
H2-T24	3.051	4.817	0.66	0.19	

Ildr2	0.764	1.222	0.73	0.19		
Klhdc1	1.534	2.397	0.67	0.19		
Sv2b	0.366	0.552	0.63	0.19		
5830411N06Rik	0.008	0.028	1.26	0.19		
Srf	13.728	20.657	0.60	0.19		
1810044D09Rik	2.135	3.627	0.76	0.19		
Hmgb1-rs17	0.123	0.228	0.86	0.19		
Barx2	19.399	27.080	0.65	0.19		
Ttc39c	2.254	3.372	0.63	0.19		
Car10	0.110	0.218	0.86	0.19		
Tmem221	0.524	0.873	0.74	0.19		
Uts2r	0.079	0.155	0.93	0.19		
Cxcr6	0.162	0.303	0.82	0.19		
Pla2g5	0.487	0.746	0.70	0.19		
Avpr1a	3.688	5.362	0.66	0.19		
Klf7	1.576	2.544	0.69	0.19		
Nfkbia	40.804	59.367	0.64	0.19	+	+
Clca2	1.015	1.613	0.67	0.18		
Ces2b	0.471	0.756	0.72	0.18		
Ppp1r14a	24.970	38.884	0.67	0.18		
Gm10789	0.036	0.072	0.99	0.18		
Npy1r	1.134	1.781	0.68	0.18		
H3f3b	462.308	676.361	0.66	0.18		
H2-Bl	9.908	15.050	0.62	0.18		
Stkld1	0.032	0.081	1.18	0.18		
Slc38a3	1.268	1.972	0.66	0.18		
Mir6357	1.448	2.966	0.96	0.18		
Tlr9	0.276	0.463	0.75	0.18		
4930413G21Rik	6.742	9.729	0.62	0.18		
Cdh3	15.335	20.748	0.65	0.18		
Ifitm10	0.288	0.430	0.78	0.18		
Ier3	13.225	19.711	0.66	0.18		+
Gm13889	21.973	34.206	0.64	0.18		

1700071M16Rik	0.406	0.678	0.71	0.18		
Cpeb1	0.287	0.474	0.76	0.18		
Adgre1	6.079	9.324	0.63	0.18		
Oas1a	6.712	10.084	0.62	0.18		
Bst2	58.786	87.050	0.61	0.18	+	
Dnajb7	0.036	0.111	1.22	0.18		
Gm11651	1.038	1.720	0.66	0.18		
Ero1l	13.596	20.210	0.62	0.18		
Slit3	12.006	17.510	0.64	0.18		
Slc4a8	0.262	0.404	0.68	0.18		
Hist1h1b	1.686	2.831	0.72	0.18		
Serpina6	0.029	0.090	1.15	0.18		
Stfa1	1431.810	#######	0.60	0.18		
Plek	5.738	8.682	0.61	0.18		+
Hsd17b14	1.564	2.405	0.66	0.18		
Serpina12	8.748	11.675	0.67	0.18		
Tnfrsf1b	3.095	4.624	0.60	0.18		
Spata20	0.064	0.128	0.92	0.18		
Ssfa2	9.873	14.832	0.65	0.17		
Rdh1	0.939	1.326	0.62	0.17		
Metrnl	10.714	16.054	0.59	0.17		
T	0.068	0.140	0.98	0.17		
Lao1	0.036	0.101	1.23	0.17		
Rtl1	12.904	15.099	0.58	0.17		
H1fx	57.848	80.652	0.59	0.17		
D830032E09Rik	0.012	0.050	1.24	0.17		
Grem1	1.509	1.815	0.65	0.17		
Ptprn	0.065	0.106	0.86	0.17		
Lrrn4cl	8.129	12.071	0.59	0.17		
Casz1	5.171	7.326	0.61	0.17		
Atp6v1b1	7.765	10.406	0.59	0.17		
Usp27x	1.503	2.380	0.64	0.17		
Vsig1	0.390	0.215	0.91	0.17		

Ly6a	108.532	167.643	0.66	0.17		
Syt15	1.668	2.549	0.63	0.17		
Fas	3.502	5.445	0.65	0.17	+	
Sp110	5.535	8.177	0.64	0.17	+	
Scrt1	0.043	0.088	0.93	0.17		
Pabpc5	0.018	0.054	1.15	0.17		
Tha1	2.066	3.162	0.70	0.17		
Dusp2	4.686	7.055	0.63	0.17		+
2310075C17Rik	0.287	0.589	0.96	0.17		
Scpep1os	6.802	9.809	0.60	0.17		
Sdr16c6	3.645	4.538	0.61	0.17		
A930041C12Rik	0.050	0.205	1.26	0.17		
Ccl20	0.067	0.192	1.11	0.17		+
Ms4a4d	2.802	4.275	0.62	0.17		
Lrrtm2	0.152	0.229	0.72	0.17		
1700001L19Rik	0.074	0.134	0.95	0.17		
Grm6	0.171	0.265	0.76	0.17		
Mfsd7a	0.956	1.564	0.69	0.17		
Nxph3	0.804	1.284	0.65	0.17		
Entpd3	0.237	0.319	0.75	0.17		
Exph5	0.466	0.695	0.59	0.16		
Steap2	0.466	0.670	0.58	0.16		
Fbxo24	0.219	0.367	0.80	0.16		
Cela1	2.042	2.929	0.68	0.16		
Scara5	2.951	4.477	0.63	0.16		
Casp14	28.067	37.681	0.63	0.16		
Il5	0.021	0.072	1.18	0.16		
Parp14	2.357	3.416	0.62	0.16	+	
Ccl5	0.344	0.664	0.94	0.16	+	+
Khnyn	4.690	6.976	0.61	0.16		
D6Ertd527e	0.331	0.524	0.76	0.16		
Dusp5	1.867	2.709	0.70	0.16		+
Lrig1	31.632	45.775	0.62	0.16		

Map3k9	0.615	0.897	0.60	0.16		
Prss22	1.040	1.465	0.66	0.16		
Gbp11	1.529	2.364	0.66	0.16		
Kcna4	0.131	0.209	0.74	0.16		
Cyp2ab1	0.071	0.153	1.02	0.16		
Lilrb4a	8.463	13.001	0.62	0.16		
4930515G16Rik	1.258	2.081	0.73	0.16		
Scn7a	2.071	2.702	0.58	0.16		
Fam13a	13.193	18.304	0.62	0.16		
Cyp2d13	0.212	0.375	0.80	0.16		
Satb1	3.739	5.376	0.60	0.16		
Cldn4	9.359	12.647	0.65	0.16		
Dpep1	25.355	35.774	0.60	0.16		
Egfr	5.092	7.355	0.61	0.16		
Oas1g	4.576	6.848	0.63	0.16		
Ypel3	54.329	80.798	0.61	0.16		
C1rl	1.588	2.387	0.65	0.16		
Olfml2a	7.421	10.583	0.60	0.16		
Kynu	0.053	0.091	0.82	0.16		+
Ablim2	2.236	3.222	0.64	0.16		
Abca1	9.046	13.292	0.64	0.16		+
Ifit2	5.687	8.883	0.64	0.16	+	+
Lama2	11.960	17.060	0.60	0.16		
Gpr157	2.264	3.327	0.62	0.16		
Stat3	21.929	31.612	0.62	0.16	+	
Epsti1	1.271	1.983	0.65	0.16	+	
Fxyd5	24.447	35.992	0.67	0.16		
H2-Eb1	5.277	8.199	0.65	0.16		
Gprc5d	0.132	0.255	0.89	0.15		
Tnfrsf12a	19.234	27.676	0.62	0.15		
C530044C16Rik	0.334	0.569	0.76	0.15		
Clic3	21.964	29.896	0.59	0.15		
Zfp395	20.393	29.440	0.61	0.15		
Gm14635	0.289	0.499	0.78	0.15		

E330020D12Rik	0.044	0.086	0.95	0.15		
A530013C23Rik	0.138	0.249	0.86	0.15		
Dyrk3	1.022	1.617	0.64	0.15		
H2-T23	58.046	85.014	0.63	0.15		
Scnn1a	1.940	2.854	0.63	0.15		
Amotl2	20.234	30.490	0.63	0.15		
Cbl	1.151	1.704	0.65	0.15		
Hopx	26.983	36.731	0.61	0.15		
Lilr4b	7.669	11.870	0.61	0.15		
Fam43a	13.537	19.477	0.60	0.15		
Trpc1	0.375	0.607	0.73	0.15		
Mgat5b	0.336	0.513	0.66	0.15		
Dusp14	12.766	17.622	0.60	0.15		
1700001K23Rik	0.099	0.271	1.20	0.14		
Il12a	0.039	0.112	1.09	0.14		
Icam1	11.717	16.412	0.56	0.14	+	+
Rho	0.076	0.143	0.84	0.14		
Ap5b1	0.866	1.297	0.62	0.14		
Ppp1r15a	18.964	24.559	0.55	0.14		+
Slc35d3	0.495	0.754	0.62	0.14		
Ms4a6d	7.989	11.540	0.60	0.14		
Tsga13	0.156	0.309	0.86	0.14		
Stard6	0.417	0.732	0.77	0.14		
Cd300lf	0.645	1.017	0.69	0.14		
Evpl	7.526	10.622	0.57	0.14		
Nacad	0.494	0.743	0.61	0.14		
Spsb1	5.373	8.233	0.61	0.14		+
Raet1e	2.325	3.511	0.61	0.14		
Nkain2	0.201	0.330	0.74	0.14		
Slc44a5	0.312	0.457	0.71	0.14		
Hephl1	0.431	0.637	0.61	0.14		
Pi16	19.172	26.070	0.58	0.14		
Chrna9	0.956	1.023	0.66	0.14		

Foxh1	0.059	0.129	1.04	0.14	
Mmrn2	9.091	12.838	0.57	0.14	
Kcne3	4.826	6.772	0.60	0.14	
Mir7015	6.217	10.674	0.81	0.14	
Prkch	9.701	13.956	0.59	0.14	
Adgrf5	11.465	17.050	0.60	0.14	
Rnf144b	3.967	5.572	0.55	0.14	
Fam227b	0.258	0.451	0.74	0.14	
Mir130c	21.572	32.785	0.62	0.14	
Trim21	1.796	2.638	0.61	0.14	+
Atp2a1	169.066	197.162	0.59	0.14	
Cldn23	2.059	2.821	0.60	0.14	
Sdr9c7	3.561	5.058	0.61	0.14	
Raet1c	2.303	3.390	0.62	0.14	
Mchr1	2.410	3.272	0.60	0.14	
Plcxd3	0.011	0.047	1.34	0.14	
Sp140	6.507	9.942	0.63	0.14	
Tll1	1.136	1.650	0.56	0.14	
Pcdh20	3.193	4.595	0.60	0.13	
Nmur1	0.102	0.199	0.90	0.13	
Bcl2a1b	1.830	2.250	0.68	0.13	
Nfe2l3	2.139	3.188	0.62	0.13	
Cpeb3	0.665	0.929	0.58	0.13	
Ttc9	3.834	5.514	0.59	0.13	
Nanog	0.060	0.128	0.95	0.13	
Slc35e4	4.087	5.748	0.57	0.13	
Acsbg2	0.006	0.034	1.25	0.13	
Zfp112	0.803	1.206	0.60	0.13	
Otol1	0.162	0.265	0.79	0.13	
Bmp6	3.206	4.910	0.61	0.13	
Pdcd1lg2	0.014	0.039	1.22	0.13	
Tmprss11e	0.084	0.142	0.85	0.13	
Il10ra	1.653	2.473	0.62	0.13	+
Fam13c	4.315	6.316	0.56	0.13	
Tas1r3	0.295	0.471	0.64	0.13	
Wnt2	5.082	7.429	0.59	0.13	

B230206H07Rik	1.737	2.523	0.56	0.13	
Prom2	3.697	5.388	0.56	0.13	
Tbc1d30	0.292	0.437	0.61	0.13	
Arl4c	17.159	24.814	0.58	0.13	
Ebi3	3.705	5.514	0.59	0.13	
Ephb6	10.470	14.306	0.54	0.13	
Krtap1-4	0.010	0.078	1.30	0.13	
Dhx58	2.686	3.992	0.57	0.13	+
Wnt2b	1.759	2.387	0.61	0.13	
A330093E20Rik	0.130	0.229	0.80	0.13	
Hsf2bp	0.553	0.830	0.61	0.13	
Crispld2	3.814	5.623	0.57	0.13	
Gm21284	0.086	0.153	0.84	0.13	
Raet1a	2.346	3.566	0.61	0.13	
Lepr	11.036	16.535	0.64	0.13	
Nrn1	21.165	30.377	0.54	0.12	
2310002L09Rik	2.252	2.992	0.59	0.12	
Trp63	18.767	25.708	0.57	0.12	
Slfn10-ps	0.466	0.708	0.64	0.12	
1700080N15Rik	0.000	0.152	1.43	0.12	
Asb10	0.536	0.818	0.68	0.12	
Mr1	6.350	9.433	0.59	0.12	
Tmco6	4.488	6.471	0.54	0.12	
Itpkc	6.753	9.511	0.54	0.12	
C430002N11Rik	0.242	0.411	0.77	0.12	
Cit	0.832	1.205	0.58	0.12	
Xirp1	5.892	7.540	0.58	0.12	
Ablim1	19.472	28.083	0.63	0.12	
Trp53inp1	6.743	9.788	0.57	0.12	
Map10	1.243	1.801	0.58	0.12	
Alox5ap	10.213	14.973	0.55	0.12	

Scn4b	0.608	0.805	0.63	0.12	
Myl7	0.519	0.859	0.83	0.12	
4931403G20Rik	0.273	0.440	0.67	0.12	
B3gnt5	1.370	1.655	0.59	0.12	
Tifa	1.190	1.846	0.65	0.12	
Ovol1	3.688	5.311	0.59	0.12	
Rhox3e	0.506	0.663	0.69	0.12	
Cd68	15.134	23.644	0.60	0.12	
Aoah	1.358	1.982	0.55	0.12	
Ephx1	15.636	22.658	0.60	0.12	
Irf9	7.331	10.510	0.57	0.12	+
Cers3	3.233	4.413	0.59	0.12	
Sfrp5	0.872	1.322	0.63	0.12	
Cyp2d26	1.036	1.299	0.61	0.12	
Stx11	1.546	2.226	0.57	0.12	
Pla2g2f	16.066	21.783	0.62	0.12	
B230307C23Rik	1.798	2.615	0.64	0.12	
C2cd4d	0.343	0.569	0.74	0.12	
Mag	0.596	0.908	0.66	0.12	
Gja1	117.093	158.792	0.54	0.12	
Cracr2a	0.187	0.363	0.89	0.12	
Gm4890	0.322	0.500	0.72	0.12	
1700099I09Rik	0.413	0.672	0.72	0.12	
Cfap45	0.466	0.738	0.69	0.11	
3110015C05Rik	0.023	0.094	1.14	0.11	
Tmeff2	10.045	13.859	0.53	0.11	
Cldn2	0.586	0.827	0.61	0.11	
Grrp1	6.609	9.868	0.54	0.11	
Timp3	46.587	64.987	0.56	0.11	
1700094J05Rik	1.467	2.122	0.63	0.11	
Lce1j	27.960	37.956	0.56	0.11	
Fbp1	2.418	3.278	0.62	0.11	

Arrdc3	27.375	36.478	0.55	0.11		
Osmr	2.474	3.537	0.54	0.11		
Gm16070	0.083	0.156	0.77	0.11		
Efemp1	33.074	47.156	0.59	0.11		
Cadm4	9.024	12.342	0.51	0.11		
Lce1i	47.787	65.112	0.59	0.11		
Pim1	12.167	17.297	0.58	0.11	+	
Atp2b3	0.268	0.376	0.62	0.11		
1810021B22Rik	2.039	2.898	0.63	0.11		
Dst	6.393	8.502	0.55	0.11		
Trf	296.885	338.973	0.54	0.11		
Hormad1	0.011	0.046	1.24	0.11		
Gbp7	3.231	4.362	0.59	0.11		
B3gnt3	4.963	7.178	0.60	0.11		
Tspan10	0.901	1.164	0.64	0.11		
Zbtb4	5.531	7.342	0.52	0.11		
Snora31	4.753	6.689	0.69	0.11		
Oas1c	0.637	0.964	0.58	0.11		
P4ha1	27.844	38.954	0.54	0.11		
Col2a1	53.104	67.348	0.50	0.11		
Ppfia2	0.206	0.305	0.61	0.11		
Asb11	1.457	2.025	0.59	0.11		
Bach2	4.297	5.651	0.55	0.11		
Tnfaip3	2.685	3.761	0.54	0.11	+	+
Igdcc3	0.128	0.217	0.74	0.11		
Plekhb2	3.297	4.364	0.51	0.10		
1700003F12Rik	1.489	2.255	0.62	0.10		
Per1	25.048	35.080	0.54	0.10		+
Tenm2	2.093	2.751	0.54	0.10		
Lad1	12.594	17.337	0.55	0.10		
Avpi1	19.226	27.477	0.61	0.10		
Btn1a1	0.151	0.256	0.73	0.10		
Gm15645	1.599	2.464	0.59	0.10		
Fcgr3	38.166	53.062	0.55	0.10		

Snord65	6.697	10.825	0.72	0.10
Tnmd	6.079	7.933	0.57	0.10
Adam8	1.394	1.972	0.57	0.10
Cd47	11.739	17.212	0.59	0.10
Mpeg1	2.149	3.095	0.54	0.10
Thbd	16.441	22.729	0.54	0.10
Rorc	2.950	4.180	0.60	0.10
Ccr1	1.151	1.666	0.57	0.10
4930483J18Rik	0.010	0.081	1.26	0.10
Zfhx2os	0.171	0.275	0.75	0.10
Zfp551	0.460	0.679	0.63	0.10
Tdo2	0.089	0.175	0.89	0.10
4930555B11Rik	0.063	0.129	1.03	0.10
Zfp383	3.836	5.450	0.53	0.10
Kcnip2	0.548	0.849	0.68	0.10
4930515G01Rik	0.460	0.740	0.66	0.10
Hebp2	7.494	9.876	0.54	0.10
Rnf17	0.042	0.077	0.85	0.10
Asb2	14.221	17.792	0.54	0.10
Gm11127	6.921	9.772	0.55	0.10
Selplg	3.163	4.747	0.58	0.10
Plekha4	3.838	5.567	0.56	0.10
Parp9	4.550	6.548	0.56	0.10
Ahr	9.018	12.835	0.55	0.10
Ica1l	0.219	0.320	0.67	0.10
Mpz	14.269	19.747	0.59	0.10
Agt	48.566	64.993	0.52	0.10
Plxdc2	6.242	8.774	0.56	0.10
Dsg3	1.889	2.387	0.51	0.10
Ptrh1	3.280	4.827	0.56	0.10
Xcr1	0.025	0.064	0.98	0.10
Zim1	9.744	12.897	0.50	0.10
Krt79	98.255	114.928	0.55	0.10

Mier1	30.355	36.214	0.52	0.09	
Narf	11.267	15.408	0.55	0.09	
2010107G23Rik	1.090	1.591	0.61	0.09	
Nrp2	17.871	24.600	0.54	0.09	
Ggt1	0.381	0.551	0.62	0.09	
Gm16793	0.429	0.663	0.65	0.09	
Sbspon	1.096	1.588	0.57	0.09	
Erich4	0.112	0.218	0.88	0.09	
Cyp2j9	1.773	2.652	0.56	0.09	
Adgrg6	1.196	1.728	0.54	0.09	
9930111J21Rik1	0.504	0.713	0.58	0.09	
Hk1	11.731	16.401	0.54	0.09	
Efhd1	5.508	7.545	0.49	0.09	
Adgrd1	5.191	6.961	0.52	0.09	
Abca8a	8.607	11.779	0.56	0.09	
Rnf125	3.785	5.385	0.55	0.09	
Egfbp2	2.082	3.140	0.61	0.09	
Agtrap	6.065	8.860	0.57	0.09	
Cntn4	1.038	1.494	0.56	0.09	
Ppp1r16b	1.860	2.613	0.54	0.09	
Uba7	4.055	6.028	0.58	0.09	
Rhox3f	0.103	0.238	0.94	0.09	
Gm16982	2.175	3.011	0.53	0.09	
F2rl1	4.017	5.627	0.57	0.09	+
Gata6	3.284	4.029	0.55	0.09	
Fam134b	8.757	12.294	0.54	0.09	
Kcnab1	0.418	0.602	0.58	0.09	
Bnip3	111.242	140.484	0.50	0.09	
Krtap6-5	0.000	0.108	1.36	0.09	
Sned1	0.948	1.404	0.59	0.09	
Ctnnd2	0.998	1.445	0.52	0.09	
Slfn5	5.601	7.432	0.54	0.09	
Foxo3	4.731	6.651	0.53	0.09	

5033403F01Rik	0.196	0.322	0.79	0.09
Oprd1	0.147	0.219	0.67	0.09
4930544G11Rik	0.048	0.122	1.12	0.09
Mir7041	34.225	49.157	0.58	0.09
Cnfn	40.589	56.659	0.59	0.09
Slc5a4a	0.003	0.032	1.27	0.09
Gm15413	0.003	0.034	1.27	0.09
Stoml3	0.068	0.179	0.99	0.09
Mafk	6.842	9.657	0.56	0.09
9630001P10Rik	0.002	0.024	1.24	0.09
Naif1	0.759	1.132	0.60	0.09
Elovl7	4.803	6.221	0.52	0.08
Pde8a	4.236	5.954	0.57	0.08
A730020M07Rik	0.029	0.072	1.00	0.08
Gm4980	5.044	6.835	0.52	0.08
Ctsl	84.710	118.496	0.57	0.08
Anks1b	0.062	0.096	0.66	0.08
A530032D15Rik	2.068	2.917	0.59	0.08
Atp13a4	1.068	1.461	0.59	0.08
Itprip	4.025	5.635	0.55	0.08
Etnppl	0.051	0.105	0.89	0.08
4930593A02Rik	0.040	0.141	1.20	0.08
Ptprb	4.592	6.306	0.53	0.08
Runx3	3.260	4.548	0.54	0.08
Igtp	6.836	9.912	0.56	0.08
Slfn1	0.689	1.080	0.64	0.08
Cyp26b1	27.131	33.438	0.53	0.08
Slc43a2	1.789	2.578	0.56	0.08
Ly6c1	59.467	80.655	0.50	0.08
Shisa3	0.980	1.321	0.60	0.08

Was	2.344	3.220	0.46	0.08		
Atp6v1a	16.340	22.873	0.56	0.08		
Zfp719	1.279	1.893	0.54	0.08		
Klhl3	0.336	0.514	0.70	0.08		
Bmp3	7.267	10.671	0.53	0.08		
Plekhb1	2.513	3.609	0.55	0.08		
Cacng1	23.263	30.040	0.55	0.08		
Mir1954	0.188	1.025	1.25	0.08		
Myh2	238.558	256.415	0.53	0.08		
Gbp3	4.279	5.909	0.56	0.08		
Cdc42ep3	9.236	13.331	0.53	0.08		
H6pd	12.256	17.483	0.59	0.08		
Gm2027	0.757	1.166	0.62	0.08		
Ifi202b	0.889	1.374	0.62	0.08		
Psapl1	24.333	30.860	0.50	0.08		
Rnd3	22.154	31.623	0.54	0.08		
Dkk3	16.221	22.856	0.59	0.08		
Ccr5	0.270	0.407	0.63	0.08		
Otud7a	0.373	0.497	0.61	0.08		
Rora	2.447	3.305	0.51	0.08		
Lpar5	0.355	0.582	0.68	0.08		
Efna3	5.781	7.807	0.53	0.08		
Lamb3	11.291	15.558	0.52	0.08		+
H2-M2	1.763	2.495	0.54	0.08		
Trib3	2.978	3.782	0.52	0.08		
Mc5r	0.634	1.005	0.60	0.08		
Ahrr	1.358	1.956	0.54	0.08		
Slc25a25	5.858	8.054	0.56	0.08		
Bglap	0.204	0.386	0.90	0.08		
A930007I19Rik	0.030	0.076	1.07	0.08		
Pcolce2	2.645	3.519	0.56	0.07		
Mir707	2.946	5.132	0.83	0.07		
Ddx58	3.635	5.109	0.51	0.07	+	+
Slc26a11	2.513	3.692	0.58	0.07		
Lin28b	0.032	0.065	0.87	0.07		
Birc2	7.697	10.717	0.48	0.07		+

Msr1	2.375	3.274	0.50	0.07
Gcnt2	1.424	2.016	0.52	0.07
Slc25a2	0.124	0.229	0.84	0.07
Phkg1	2.299	2.895	0.50	0.07
Pof1b	7.668	10.089	0.53	0.07
Fnip1	4.771	6.792	0.55	0.07
Pi15	4.622	6.095	0.56	0.07
Fam189a2	6.377	8.415	0.47	0.07
Bdkrb1	0.117	0.214	0.86	0.07
Angpt2	5.704	8.325	0.55	0.07
Card14	1.539	2.054	0.53	0.07
Prrg4	0.510	0.697	0.61	0.07
Ston1	4.256	5.740	0.50	0.07
Ccr10	0.337	0.511	0.69	0.07
Gm5416	47.732	54.939	0.48	0.07
P2ry13	0.817	1.212	0.58	0.07
Rab39	0.080	0.141	0.75	0.07
Mex3b	5.241	7.463	0.54	0.07
Plp1	5.072	7.214	0.50	0.07
Prdm14	0.037	0.076	0.98	0.07
Cyp2d37-ps	0.248	0.493	0.81	0.07
Mdfic	8.290	11.323	0.47	0.07
Abca5	1.597	2.220	0.54	0.07
Ifi47	2.172	3.054	0.54	0.07
Zc3hav1	3.591	5.080	0.54	0.07
Cd14	3.396	4.749	0.50	0.07
Ntn4	1.385	2.020	0.55	0.07
Csrp1	108.834	150.726	0.54	0.07
Rhov	5.922	8.086	0.52	0.07
Rasl11a	4.746	6.595	0.48	0.07
Prss35	7.310	10.236	0.53	0.07
Hoxb1	0.034	0.074	1.06	0.07
Rarres1	6.486	8.995	0.56	0.07
Mir3103	3.932	6.907	0.77	0.07
Spata31d1d	0.000	0.012	1.39	0.07
Cdh19	0.799	1.118	0.54	0.07

H2-M10.6	0.302	0.471	0.68	0.07		
Bcl3	2.675	3.735	0.55	0.07		+
Wnt3	2.577	3.579	0.52	0.06		
Colec11	0.836	1.195	0.60	0.06		
Sash1	15.953	20.971	0.46	0.06		
Ttc22	1.905	2.616	0.54	0.06		
Atp13a5	0.044	0.078	0.84	0.06		
Igf2bp2	16.064	21.092	0.47	0.06		
Gpr171	0.435	0.664	0.64	0.06		
Krt77	25.498	32.150	0.52	0.06		
Halr1	1.828	2.655	0.65	0.06		
Nrtn	10.880	14.690	0.53	0.06		
Klhl15	0.506	0.717	0.54	0.06		
AI413582	12.070	16.951	0.50	0.06		
Mir6927	2.045	4.035	0.83	0.06		
A830052D11Rik	0.419	0.683	0.64	0.06		
AF251705	1.751	2.631	0.55	0.06		
Zfp773	0.165	0.262	0.72	0.06		
2610016A17Rik	2.314	2.882	0.59	0.06		
Pxdc1	15.920	23.072	0.57	0.06		
2310050C09Rik	41.537	53.716	0.52	0.06		
Pamr1	10.752	14.600	0.54	0.06		
Asb4	1.321	1.845	0.53	0.06		
Apol7d	0.153	0.259	0.96	0.06		
Elovl4	20.428	26.459	0.54	0.06		
Jak2	8.238	11.295	0.50	0.06	+	
4930556M19Rik	0.227	0.360	0.62	0.06		
Gm11559	0.020	0.101	1.23	0.06		
Tnfrsf18	3.476	4.595	0.49	0.06		
Fam110a	9.247	12.940	0.53	0.06		
Gabra2	0.398	0.590	0.67	0.06		
Ptger4	5.042	6.888	0.49	0.06		+

Gm15441	52.527	67.859	0.45	0.06	
C1qc	66.857	92.491	0.55	0.06	
Ttll10	0.459	0.716	0.58	0.06	
Il27ra	0.617	0.920	0.58	0.06	
Cd86	2.937	4.053	0.49	0.06	+
Htr1b	0.458	0.664	0.64	0.06	
Sox5	0.728	1.019	0.53	0.06	
9430060I03Rik	0.833	1.186	0.54	0.06	
Eps8l1	4.464	6.061	0.53	0.06	
H60c	3.136	3.817	0.50	0.06	
Serpinb6d	0.520	0.751	0.60	0.06	
Mreg	2.981	3.986	0.50	0.06	
Lamc3	10.467	13.025	0.52	0.06	
Ncf1	3.986	5.203	0.42	0.06	
F13a1	41.516	55.496	0.51	0.06	
Hsf4	0.196	0.289	0.66	0.06	
Angptl3	0.037	0.096	1.04	0.06	
Igsf10	23.303	31.395	0.54	0.06	
LOC105245869	0.731	1.062	0.55	0.06	
Pthlh	3.595	4.824	0.55	0.06	
4933407L21Rik	0.122	0.230	0.92	0.06	
Epb4.2	0.031	0.063	0.86	0.06	
Lama3	2.748	3.697	0.49	0.06	
Tssk6	1.187	1.673	0.54	0.06	
Atg2b	3.189	4.415	0.51	0.06	
Aph1b	3.340	4.506	0.47	0.06	
Pramef8	3.856	5.410	0.50	0.06	
Pla2g4f	3.832	5.042	0.54	0.06	
Gnaz	3.118	4.244	0.48	0.06	
Abca8b	2.440	3.192	0.50	0.05	
Klf5	12.708	16.773	0.47	0.05	
Klf15	7.036	9.952	0.52	0.05	
Cyp39a1	3.890	5.193	0.46	0.05	
Aaed1	9.747	13.988	0.51	0.05	

Calm4	515.770	560.958	0.52	0.05
1700023F06Rik	0.242	0.386	0.77	0.05
4833412C05Rik	1.192	1.831	0.57	0.05
Sln	162.842	213.238	0.49	0.05
Hif3a	8.374	11.465	0.52	0.05
Tmem235	0.012	0.048	1.22	0.05
Pld3	22.667	30.477	0.48	0.05
Sntb2	3.697	5.221	0.52	0.05
Slc3a2	38.539	51.111	0.49	0.05
9530026P05Rik	0.281	0.465	0.70	0.05
Prdm8	0.665	0.890	0.51	0.05
Cep57l1	2.973	4.182	0.55	0.05
Hdc	101.546	132.453	0.49	0.05
Kif6	0.134	0.206	0.69	0.05
Phldb3	3.734	5.099	0.48	0.05
Chrdl1	5.924	7.671	0.48	0.05
Amz1	0.521	0.788	0.58	0.05
A930001C03Rik	0.478	0.794	0.70	0.05
Ccdc14	2.067	2.854	0.46	0.05
Rab25	22.217	28.044	0.48	0.05
Islr2	0.929	1.377	0.56	0.05
4930528A17Rik	0.727	1.059	0.56	0.05
Cbln2	1.211	1.717	0.55	0.05
Tbl1x	9.130	12.559	0.49	0.05
Acvr1b	6.799	9.159	0.48	0.05
Tex14	0.027	0.050	0.79	0.05
Cblc	1.860	2.546	0.51	0.05
Gm16532	0.010	0.040	1.09	0.05
Agpat4	12.105	16.867	0.53	0.05
Tspyl5	0.562	0.835	0.58	0.05
Nexn	22.069	26.341	0.43	0.05

C6	0.221	0.302	0.65	0.05		
Mir5130	3.584	6.034	0.72	0.05		
Ptpre	2.413	3.356	0.51	0.05		+
Slc30a1	5.194	7.112	0.48	0.05		
Rab3b	1.020	1.560	0.57	0.05		
Smco3	1.106	1.525	0.55	0.05		
Oaz3	0.164	0.302	0.82	0.05		
D930015M05Rik	0.660	1.037	0.61	0.05		
Gm19522	0.047	0.096	0.97	0.05		
Arhgap15	0.613	0.883	0.51	0.05		
Rdh9	2.362	2.948	0.49	0.05		
Prrg3	6.364	8.230	0.46	0.05		
Amica1	0.256	0.359	0.61	0.05		
Cltb	30.432	39.304	0.43	0.05		
Abcg3	0.719	1.060	0.59	0.05		
Lyz2	55.715	75.780	0.50	0.05		
Rgs1	2.478	3.529	0.53	0.05		
B2m	244.320	331.126	0.53	0.05	+	
C230037L18Rik	0.203	0.354	0.82	0.05		
Eepd1	8.189	10.453	0.51	0.05		
Apcdd1	70.850	94.425	0.51	0.05		
Anpep	26.422	34.769	0.44	0.05		
Pcp2	0.327	0.599	0.81	0.04		
Mir7050	46.150	65.005	0.52	0.04		
Ago2	3.791	5.029	0.49	0.04		
Ptx3	6.132	8.324	0.47	0.04		+
Slc6a4	13.934	17.784	0.47	0.04		
Hist3h2a	27.053	35.743	0.52	0.04		
Mypn	7.450	8.720	0.48	0.04		
Cmya5	5.554	6.433	0.50	0.04		
Slc37a2	1.446	2.027	0.50	0.04		
Hoxd3os1	0.609	0.971	0.66	0.04		
Trpm3	0.052	0.080	0.65	0.04		

E330009J07Rik	0.248	0.361	0.67	0.04	
Ipmk	4.091	5.683	0.51	0.04	
Cdkn2b	1.857	2.578	0.52	0.04	
Efhc1	0.733	1.038	0.59	0.04	
Fbxo2	0.166	0.304	0.74	0.04	
Gm13051	0.120	0.179	0.68	0.04	
Ace2	1.832	2.394	0.47	0.04	
C1qtnf4	2.308	3.293	0.52	0.04	
Derl3	0.262	0.429	0.72	0.04	
Dpp4	6.166	8.725	0.54	0.04	
Reps2	1.556	2.121	0.46	0.04	
Cadm1	6.530	8.730	0.48	0.04	
Dscaml1	0.050	0.083	0.72	0.04	
Mospd4	0.304	0.499	0.76	0.04	
Gpr143	0.436	0.587	0.65	0.04	
Cxcl14	85.465	112.460	0.54	0.04	
Glt1d1	0.328	0.509	0.64	0.04	
Il1rn	2.310	3.054	0.47	0.04	
Psmb8	9.857	13.297	0.51	0.04	+
Lrp5	14.654	19.590	0.47	0.04	
Cd160	0.153	0.242	0.73	0.04	
Kcnj5	0.012	0.029	0.98	0.04	
Il7	0.187	0.285	0.72	0.04	+
Cntn1	0.476	0.688	0.55	0.04	
Elk4	1.898	2.629	0.53	0.04	
Anxa1	128.282	165.165	0.46	0.04	
Tmem255b	0.730	1.117	0.62	0.04	
Pla2r1	1.057	1.487	0.51	0.04	
Lmx1a	0.456	0.647	0.53	0.04	
6720468P15Rik	0.346	0.494	0.63	0.04	
Gm4285	2.178	3.169	0.56	0.04	
Nupr1l	0.736	1.158	0.68	0.04	
Lamc1	53.850	70.640	0.48	0.04	
Fhl2	6.755	9.356	0.52	0.04	

Rnf31	4.299	5.944	0.48	0.04	+	
Unc5b	9.144	12.278	0.49	0.04		
Upp1	2.513	3.553	0.51	0.04	+	
Sprtn	2.607	3.687	0.52	0.04		
Slc17a7	0.139	0.225	0.71	0.04		
H2-Aa	9.023	12.265	0.48	0.04		
Exoc6b	8.617	11.524	0.49	0.04		
H2-M5	2.132	2.656	0.50	0.03		
Krt23	11.840	14.461	0.49	0.03		
U2surp	6.184	8.437	0.48	0.03		
Sppl2b	10.943	15.100	0.49	0.03		
Pir	3.033	4.102	0.48	0.03		
Cpne7	0.431	0.614	0.57	0.03		
Sparcl1	130.728	174.143	0.45	0.03		
Zfp93	1.909	2.756	0.49	0.03		
6030469F06Rik	0.014	0.057	1.23	0.03		
Slc40a1	12.888	16.709	0.47	0.03		
Ncf2	2.724	3.777	0.46	0.03		
Spon1	7.580	9.685	0.43	0.03		
Gabarapl1	26.350	35.744	0.44	0.03		
Bnipl	7.208	9.145	0.49	0.03		
Srrm4os	0.104	0.157	0.68	0.03		
Abca4	0.217	0.310	0.55	0.03		
Hbegf	2.407	3.368	0.53	0.03		+
Plek2	5.287	7.146	0.45	0.03		
Mir7012	0.252	1.033	1.21	0.03		
Edar	3.493	4.353	0.46	0.03		
Gpr33	0.000	0.032	1.34	0.03		
Tgfbr3	29.530	42.087	0.59	0.03		
Hivep3	0.178	0.244	0.53	0.03		
Iffo2	5.709	7.596	0.50	0.03		
Soga3	0.061	0.101	0.71	0.03		
Tlr7	0.846	1.207	0.49	0.03		
Il4ra	6.641	8.790	0.45	0.03		
Ier5	14.613	19.745	0.48	0.03		+

Dlx3	14.402	16.628	0.48	0.03
2010010A06Rik	2.255	3.002	0.51	0.03
Naprt	3.806	5.212	0.48	0.03
Samd5	0.903	1.236	0.50	0.03
Hps4	5.099	7.255	0.52	0.03
Ms4a6b	7.219	9.940	0.48	0.03
Plxna4os1	0.048	0.092	0.96	0.03
Itpr3	4.661	6.042	0.41	0.03
Pik3r5	0.253	0.369	0.55	0.03
Dnmt3l	0.222	0.330	0.60	0.03
Sema3e	1.001	1.375	0.51	0.03
Irf2bp2	28.900	40.073	0.51	0.03
Micalcl	0.805	1.158	0.58	0.03
Plod2	9.396	12.701	0.45	0.03
Sptbn2	2.964	4.022	0.56	0.03
Slc41a1	7.297	9.831	0.45	0.03
St14	7.421	9.512	0.47	0.03
Ptgfr	9.128	12.046	0.45	0.03
Tfpi2	3.908	5.334	0.49	0.03
Gm16157	0.024	0.082	1.21	0.03
Tspan11	10.695	14.518	0.51	0.03
Hs3st4	0.071	0.092	0.85	0.03
Lrp2	0.021	0.034	0.70	0.03
Dusp16	6.873	9.539	0.49	0.03
Zbtb38	1.313	1.821	0.52	0.03
Zbtb16	3.785	4.671	0.45	0.03
Grhl3	2.447	3.299	0.52	0.03
Hspb1	240.624	292.247	0.47	0.03
Gdf11	1.541	2.129	0.51	0.03
Bmp7	21.283	27.548	0.47	0.03
Kcnj3	0.227	0.325	0.53	0.03
C3ar1	2.374	3.209	0.45	0.03
Ak4	2.424	3.355	0.50	0.03
Anxa9	5.896	7.624	0.48	0.03
Serpinb9e	0.008	0.046	1.18	0.03

Brwd3	0.949	1.322	0.50	0.03		
Lpcat2	2.815	3.589	0.45	0.03		
Rag2	0.000	0.013	1.35	0.03		
Tmem154	2.294	3.132	0.47	0.02		
Lpar3	0.921	1.290	0.51	0.02		
Slc2a3	1.414	2.015	0.53	0.02		+
Nhlrc3	2.085	2.882	0.49	0.02		
Cwh43	3.016	3.736	0.44	0.02		
Rad9b	1.006	1.383	0.50	0.02		
7-Mar	10.225	13.324	0.42	0.02		
Robo1	8.515	10.913	0.45	0.02		
A630023P12Rik	0.067	0.171	0.96	0.02		
Dync2li1	6.422	8.867	0.52	0.02		
Tmem100	14.487	20.249	0.48	0.02		
Lrrc1	4.133	5.693	0.52	0.02		
Slamf1	0.036	0.074	0.89	0.02		
Tuft1	9.815	12.222	0.44	0.02		
Arhgap20	1.735	2.382	0.47	0.02		
Gpr37	0.627	0.866	0.51	0.02		
Myoz3	2.430	2.783	0.53	0.02		
Slco2a1	4.177	5.704	0.51	0.02		
Tbc1d15	10.395	13.901	0.43	0.02		
Serpina3g	2.492	3.508	0.54	0.02		
Cdcp1	1.090	1.494	0.51	0.02		
Maml3	2.376	3.032	0.45	0.02		
Ppp2r2c	1.123	1.558	0.52	0.02		
Rassf9	2.787	3.693	0.48	0.02		
Gatsl3	6.474	8.698	0.49	0.02		
Mir7018	7.148	9.851	0.60	0.02		
Adamtsl1	2.511	3.238	0.45	0.02		
Herc6	0.726	0.964	0.49	0.02	+	
Cacnb2	0.321	0.431	0.49	0.02		
Gm5084	2.918	3.956	0.50	0.02		
Peli3	0.464	0.694	0.65	0.02		
Abcb1a	1.253	1.740	0.47	0.02		

Wee1	7.482	9.951	0.46	0.02	
Sp6	3.315	4.068	0.49	0.02	
Malt1	2.592	3.542	0.45	0.02	
Npr3	2.688	3.787	0.48	0.02	
Gm21119	0.066	0.105	0.79	0.02	
Esco2	4.431	5.959	0.44	0.02	
BC048602	0.221	0.422	0.83	0.02	
H2-M3	3.392	4.670	0.49	0.02	
2900026A02Rik	16.352	21.602	0.49	0.02	
Gm5111	0.680	0.983	0.61	0.02	
Prex2	2.015	2.775	0.48	0.02	
Crct1	117.725	143.186	0.44	0.02	
Cd33	1.196	1.603	0.48	0.02	
Eml2	9.237	12.619	0.46	0.02	
Nfatc1	6.548	9.030	0.52	0.02	
Rlbp1	0.085	0.130	0.74	0.02	
Elfn2	0.058	0.082	0.75	0.02	
Exoc4	6.114	8.161	0.48	0.02	
Cysrt1	22.705	27.114	0.48	0.02	
Lcp2	2.305	3.179	0.47	0.02	+
Lims2	6.409	8.864	0.49	0.02	
4632404H12Rik	0.703	1.001	0.53	0.02	
Zfp939	1.130	1.647	0.54	0.02	
Lrrc48	0.585	0.795	0.51	0.02	
Bco1	0.096	0.165	0.76	0.02	
Ftcd	0.173	0.236	0.63	0.02	
Abhd4	20.392	26.605	0.43	0.01	
Dclre1c	1.518	2.048	0.49	0.01	
Rsph9	1.381	1.895	0.53	0.01	
Akr1cl	3.954	4.837	0.46	0.01	
Mir5113	11.475	16.124	0.54	0.01	
Kcnc4	2.134	2.392	0.45	0.01	
Cyp4f39	2.643	3.611	0.52	0.01	
Srrm4	0.203	0.292	0.59	0.01	

Il13ra1	11.067	15.501	0.48	0.01		
Gm16998	0.035	0.107	1.08	0.01		
Oas2	1.389	1.962	0.52	0.01	+	
Pstpip2	2.943	3.673	0.42	0.01		
Bicc1	4.503	6.176	0.51	0.01		
Slc27a6	5.137	6.785	0.42	0.01		
Pkp3	20.322	26.262	0.52	0.01		
Spice1	2.770	3.634	0.43	0.01		
Ly6c2	35.160	48.666	0.47	0.01		
Paqr5	2.848	3.488	0.46	0.01		
Oprl1	0.023	0.053	0.95	0.01		
H2-K1	124.618	162.550	0.43	0.01		
Lzts1	0.678	0.943	0.53	0.01		
Egln1	27.059	36.411	0.48	0.01		
Ifi35	9.793	13.675	0.44	0.01	+	
Tram2	1.377	1.823	0.53	0.01		
Npl	19.560	25.130	0.50	0.01		
Zbtb10	2.137	2.917	0.49	0.01		+
Cyp2b9	0.447	0.646	0.57	0.01		
9330182L06Rik	0.932	1.257	0.51	0.01		
Slc28a3	0.291	0.396	0.51	0.01		
Fam229a	0.000	0.118	1.24	0.01		
Abcg1	2.917	3.928	0.49	0.01		
Plet1	4.139	5.348	0.44	0.01		
Srsf5	115.994	142.665	0.42	0.01		
Slc11a1	1.778	2.315	0.43	0.01		
Tgfa	1.265	1.787	0.50	0.01		
Hspa2	4.687	6.329	0.43	0.01		
Gna15	2.443	3.551	0.56	0.01		
Arhgap26	1.235	1.713	0.48	0.01		
H2-Ab1	10.375	14.167	0.50	0.01		
Peg3	38.317	49.787	0.46	0.01		
C3	54.571	52.776	0.47	0.01		
Fgd6	1.299	1.879	0.51	0.01		
Hotairm1	7.311	10.190	0.52	0.01		

Mylk2	8.226	9.523	0.47	0.01	
H2-T10	13.779	18.004	0.47	0.01	
Frat2	3.897	5.146	0.46	0.01	
Npc1l1	0.151	0.212	0.57	0.01	
Magee2	0.293	0.438	0.57	0.01	
H2-T22	61.863	80.559	0.44	0.01	
Pirb	5.055	6.817	0.44	0.01	
Zfp169	0.495	0.690	0.54	0.01	
Bcl2a1d	1.560	1.938	0.53	0.01	
Idua	3.753	5.158	0.46	0.01	
Gm15446	0.891	1.165	0.51	0.01	
Neat1	10.054	12.731	0.43	0.01	
Myh9	36.392	46.108	0.42	0.01	
Fzd10	11.317	14.219	0.50	0.01	
Ip6k3	2.620	2.992	0.57	0.01	
Afp	0.003	0.032	1.32	0.01	
Cage1	0.184	0.278	0.54	0.01	
Rusc2	5.848	7.568	0.43	0.01	
Kcnh3	0.389	0.529	0.51	0.01	
Ero1lb	1.286	1.773	0.47	0.01	
Susd2	5.839	7.724	0.45	0.01	
Enpp3	3.027	4.298	0.51	0.01	
Tmem79	5.809	7.845	0.45	0.01	
Npas4	0.235	0.296	0.54	0.01	
Icosl	7.222	9.743	0.48	0.01	
Rhox3a	0.354	0.484	0.75	0.01	
Mir8099-2	15.156	21.168	0.47	0.00	
Kmt2e	10.031	13.180	0.49	0.00	
4930555K05Rik	0.030	0.122	1.22	0.00	
Tlr2	3.448	4.469	0.46	0.00	+
Sgk1	11.687	15.346	0.49	0.00	+
Cmip	15.474	19.941	0.43	0.00	
Ncam2	0.364	0.465	0.49	0.00	
Naga	25.520	33.797	0.45	0.00	
Hoxc8	9.305	13.208	0.54	0.00	

Zfp119b	2.869	3.756	0.50	0.00	
Sdr42e1	7.114	9.257	0.45	0.00	
Lrig3	11.111	14.886	0.45	0.00	
Ankrd50	12.022	15.831	0.46	0.00	
Sash3	1.529	2.038	0.43	0.00	
Samd9l	3.935	4.875	0.47	0.00	+
Cmah	0.819	0.998	0.42	0.00	
Cdc40	9.504	12.838	0.44	0.00	
Mir8099-1	15.156	21.168	0.47	0.00	
Rbfox1	1.932	2.348	0.45	0.00	
Podxl	9.577	13.288	0.48	0.00	
St8sia6	0.155	0.237	0.56	0.00	
Hip1r	6.401	8.479	0.43	0.00	
Acta1	2932.210	#######	0.42	0.00	
Tmem41a	7.438	9.291	0.40	0.00	
Trcg1	0.000	0.016	1.29	0.00	
Polg2	4.187	5.505	0.46	0.00	
Mpzl3	0.748	1.022	0.55	0.00	
Klhl36	2.272	3.045	0.46	0.00	
Irak3	2.282	3.028	0.44	0.00	
Cldn10	7.675	10.104	0.45	0.00	
LOC102634401	0.060	0.148	0.91	0.00	
Sun2	28.299	38.402	0.49	0.00	
Klk1b26	2.051	3.079	0.56	0.00	
Ank	10.332	13.255	0.42	0.00	
Cyp2b13	0.814	0.529	-0.54	0.00	
Cntf	1.160	0.805	-0.55	0.00	
2610206C17Rik	0.157	0.064	-0.89	0.00	
Apol8	0.117	0.036	-1.05	0.00	
AU021063	2.333	1.550	-0.52	0.00	
Bend7	0.717	0.465	-0.55	0.00	
1600002K03Rik	5.996	4.231	-0.50	0.00	

Fdft1	22.219	15.831	-0.45	0.00
Cebpg	11.299	8.140	-0.45	0.00
Arhgap20os	6.159	4.380	-0.51	0.00
Ucn2	5.913	3.739	-0.52	0.00
Adamts16	4.845	1.223	-0.51	0.00
Ndufb9	184.443	127.881	-0.45	0.00
Klk12	1.336	0.829	-0.55	0.00
Dcaf12l2	0.489	0.298	-0.57	0.00
Unc119	18.984	13.263	-0.46	0.00
Rilp	2.650	1.763	-0.47	0.00
Hist1h4k	12.213	8.782	-0.50	0.00
Pccb	26.313	18.131	-0.46	0.00
Hmgcr	11.879	8.286	-0.48	0.00
Slc26a6	1.360	0.968	-0.48	0.00
Ift20	36.779	25.113	-0.49	-0.01
Nkapl	0.588	0.394	-0.59	-0.01
Tmem169	0.195	0.110	-0.71	-0.01
Gm21293	1.484	1.014	-0.54	-0.01
Aars	21.372	14.479	-0.45	-0.01
3830408C21Rik	0.499	0.313	-0.63	-0.01
LOC100038947	0.982	0.530	-0.64	-0.01
Grin1	0.084	0.040	-0.77	-0.01
Them7	0.061	0.000	-1.56	-0.01
Lime1	6.447	4.679	-0.46	-0.01
Slx1b	3.935	2.757	-0.49	-0.01
Uchl3	19.385	13.719	-0.45	-0.01
Baat	0.077	0.023	-1.06	-0.01
Prkcz	0.875	0.597	-0.49	-0.01
Sirpb1b	0.834	0.457	-0.63	-0.01
Sdhb	106.823	72.925	-0.46	-0.01
Ccdc101	13.825	10.149	-0.47	-0.01
Crb2	0.123	0.073	-0.69	-0.01
Polr3e	8.492	6.105	-0.44	-0.01
Mir7084	42.476	30.220	-0.52	-0.01

Mir6936	2.987	0.816	-1.12	-0.01	
Ccdc58	11.508	8.095	-0.48	-0.01	
Dhcr7	15.678	11.456	-0.43	-0.01	
Ndufb3	181.211	125.587	-0.46	-0.01	
Pmepa1	12.168	8.389	-0.47	-0.01	+
Acacb	5.048	2.880	-0.47	-0.01	
Klk1b11	0.999	0.589	-0.64	-0.01	
Cox8a	467.428	339.107	-0.41	-0.01	
Pcsk1n	0.184	0.050	-0.99	-0.01	
Itih2	0.131	0.049	-0.91	-0.01	
Taf1c	3.208	2.309	-0.45	-0.01	
Zfp354b	0.901	0.601	-0.55	-0.01	
Mir6944	12.826	8.493	-0.58	-0.01	
Tmem265	7.816	5.450	-0.51	-0.01	
Tpgs2	2.215	1.535	-0.47	-0.01	
Alyref	84.056	60.053	-0.44	-0.01	
4930513N10Rik	0.443	0.283	-0.57	-0.01	
Fam229b	2.070	1.419	-0.55	-0.01	
Kcnh2	2.295	1.555	-0.49	-0.01	
Slc9a5	1.797	1.249	-0.52	-0.01	
Panx2	0.050	0.019	-0.93	-0.01	
Uqcr10	301.059	209.963	-0.46	-0.01	
Atp6v0a2	6.204	4.350	-0.49	-0.01	
Cops7b	6.828	4.869	-0.49	-0.01	
E530011L22Rik	1.705	1.267	-0.47	-0.01	
Magix	0.292	0.159	-0.70	-0.01	
Gata1	0.871	0.556	-0.57	-0.02	
Nlrc4	0.185	0.092	-0.72	-0.02	
Eci1	57.340	33.750	-0.47	-0.02	
Psd	4.243	2.995	-0.45	-0.02	
Gm12338	626.889	436.666	-0.45	-0.02	
Pcsk9	8.491	5.898	-0.48	-0.02	
Hsd3b3	0.183	0.072	-0.91	-0.02	
Cib1	16.903	12.066	-0.49	-0.02	

Grin2d	0.719	0.518	-0.53	-0.02
Dld	59.555	40.617	-0.45	-0.02
Ndufa12	76.817	52.399	-0.48	-0.02
Zfp133-ps	0.284	0.123	-0.88	-0.02
Oraov1	6.123	4.353	-0.50	-0.02
Ikzf1	1.616	1.155	-0.49	-0.02
Rarb	1.460	0.986	-0.50	-0.02
Rims4	0.362	0.132	-1.00	-0.02
Mir6948	31.827	21.680	-0.55	-0.02
Pdap1	50.838	35.969	-0.44	-0.02
Syce1l	0.585	0.318	-0.74	-0.02
Epyc	0.134	0.051	-0.94	-0.02
Gm9573	0.023	0.000	-1.49	-0.02
Taf5	4.662	3.247	-0.51	-0.02
Zfp697	0.642	0.388	-0.55	-0.02
Fam57b	1.865	1.171	-0.52	-0.02
Tecta	0.078	0.042	-0.77	-0.02
Ccdc62	0.675	0.472	-0.51	-0.02
Lrrtm4	0.269	0.077	-0.72	-0.02
Mir678	13.762	8.395	-0.61	-0.02
Suclg1	73.516	49.754	-0.46	-0.02
Gin1	2.677	1.857	-0.51	-0.02
Spata5	4.512	3.143	-0.46	-0.02
Fndc3c1	4.183	2.862	-0.47	-0.02
Catsper4	0.059	0.000	-1.54	-0.02
Mkrn2os	0.277	0.078	-0.79	-0.02
Sox11	2.571	1.768	-0.45	-0.02
Spata33	0.291	0.085	-1.16	-0.02
Pwwp2a	4.277	3.018	-0.49	-0.02
Lsm5	31.917	22.959	-0.48	-0.02
Serpinb9c	0.349	0.203	-0.66	-0.02
Foxc2	0.638	0.404	-0.58	-0.02
Cadps2	0.496	0.306	-0.54	-0.02
Stk33	0.108	0.037	-1.06	-0.02
Foxd2os	1.168	0.774	-0.56	-0.02
Mrpl12	86.843	60.813	-0.50	-0.02

Tmem69	5.174	3.705	-0.47	-0.02
Acat2	59.584	40.335	-0.47	-0.02
Rgl3	0.744	0.467	-0.58	-0.02
Vpreb3	0.277	0.073	-1.20	-0.02
Mir3076	21.686	11.752	-0.64	-0.02
Pdha2	0.539	0.309	-0.66	-0.02
Mir133b	1.627	0.556	-1.03	-0.02
Aplnr	25.624	18.382	-0.45	-0.03
Zranb2	18.111	12.660	-0.50	-0.03
2900009J06Rik	0.718	0.332	-0.92	-0.03
Kctd21	2.120	1.245	-0.52	-0.03
Cmc4	5.771	3.949	-0.53	-0.03
Scn11a	0.030	0.008	-1.17	-0.03
Idi2	0.058	0.000	-1.61	-0.03
Pop5	17.799	12.143	-0.51	-0.03
Atp5l	652.992	431.521	-0.48	-0.03
Cpvl	0.060	0.000	-1.55	-0.03
Dock5	0.977	0.678	-0.50	-0.03
Sgcz	0.063	0.018	-1.08	-0.03
Slc41a2	0.620	0.412	-0.56	-0.03
Cx3cr1	2.652	1.767	-0.50	-0.03
Mir1839	3.241	1.207	-0.96	-0.03
Gm8923	5.009	3.514	-0.46	-0.03
Snord123	2.016	0.501	-1.17	-0.03
Zmynd19	13.086	9.416	-0.47	-0.03
Lipn	0.673	0.323	-0.70	-0.03
Txlng	4.531	3.081	-0.47	-0.03
Trmt61a	2.868	1.983	-0.55	-0.03
Dmrt1	0.043	0.000	-1.54	-0.03
Vmn1r-ps79	0.030	0.000	-1.54	-0.03
Fam69b	5.184	3.573	-0.50	-0.03
Eps8l3	1.400	0.514	-0.59	-0.03
Nek8	3.330	2.306	-0.49	-0.03
Tmem86b	1.518	0.974	-0.58	-0.03
Cox6c	481.530	329.571	-0.46	-0.03

Zdhhc23	0.181	0.069	-0.98	-0.03
Fn3krp	3.818	2.608	-0.50	-0.03
Tmsb15b2	5.020	3.375	-0.52	-0.04
Adgrb2	2.378	1.616	-0.49	-0.04
Anxa13	0.304	0.036	-1.19	-0.04
Slco1a5	0.034	0.000	-1.49	-0.04
Prkar2b	32.102	18.144	-0.47	-0.04
Arxes1	37.902	21.868	-0.49	-0.04
Cxxc4	0.753	0.530	-0.51	-0.04
Gareml	0.212	0.138	-0.61	-0.04
Tmem216	4.980	3.477	-0.53	-0.04
Abhd11os	4.151	2.424	-0.53	-0.04
Cnot4	2.900	2.072	-0.46	-0.04
Meis2	5.568	3.560	-0.50	-0.04
Cpt1b	14.869	7.911	-0.50	-0.04
Cox7a2	259.387	179.559	-0.47	-0.04
Hist2h4	0.210	0.000	-1.48	-0.04
Zfp760	2.381	1.597	-0.52	-0.04
3110045C21Rik	3.373	2.194	-0.63	-0.04
Zfp322a	3.673	2.341	-0.55	-0.04
Ceacam10	0.333	0.084	-0.91	-0.04
Cdh23	0.064	0.031	-0.81	-0.04
Fam20c	4.375	3.033	-0.48	-0.04
Gins2	20.761	15.242	-0.45	-0.04
AI854517	0.023	0.000	-1.67	-0.04
Clybl	18.409	11.755	-0.52	-0.04
Defb14	0.847	0.200	-1.08	-0.04
Gzmk	0.129	0.000	-1.58	-0.04
Rangrf	10.810	7.724	-0.48	-0.04
Klhl25	5.089	3.428	-0.51	-0.05
Dnah5	0.008	0.000	-1.58	-0.05
Brms1l	7.837	5.453	-0.49	-0.05
Gm3704	0.405	0.248	-0.66	-0.05
Gm14393	1.108	0.629	-0.68	-0.05
Tmigd3	2.629	1.816	-0.50	-0.05

Acsm3	2.257	1.472	-0.51	-0.05
Shank2	0.103	0.053	-0.66	-0.05
Lypd2	1.235	0.582	-0.78	-0.05
Mup21	0.299	0.117	-0.94	-0.05
Cenpk	4.545	3.066	-0.56	-0.05
Gcsh	43.307	29.650	-0.48	-0.05
Lsp1	20.110	10.620	-0.48	-0.05
D930007P13Rik	0.317	0.166	-0.82	-0.05
Snora7a	2.668	1.196	-0.89	-0.05
Zfat	1.225	0.868	-0.54	-0.05
Tnfrsf26	1.331	0.940	-0.51	-0.05
Mllt11	4.748	3.164	-0.54	-0.05
Zbed4	8.176	5.474	-0.50	-0.05
Unc5d	0.206	0.124	-0.59	-0.05
Zfp663	0.154	0.087	-0.70	-0.05
Gstm7	5.727	3.968	-0.52	-0.05
Tmem29	3.174	2.201	-0.48	-0.05
Znhit1	52.709	34.783	-0.51	-0.05
Donson	3.967	2.703	-0.54	-0.05
2010016I18Rik	0.324	0.184	-0.76	-0.05
Ptpn7	0.766	0.506	-0.55	-0.05
Serpina3k	0.745	0.347	-0.68	-0.06
Decr1	22.941	14.283	-0.50	-0.06
EU599041	0.162	0.062	-0.98	-0.06
Fam129c	0.600	0.364	-0.64	-0.06
Orc1	1.314	0.893	-0.58	-0.06
Pomc	0.410	0.200	-0.84	-0.06
Tamm41	9.937	6.898	-0.51	-0.06
Mrpl18	55.045	39.269	-0.47	-0.06
A430078I02Rik	0.329	0.208	-0.63	-0.06
2010107E04Rik	254.988	163.849	-0.57	-0.06
Rprd1a	7.959	5.441	-0.55	-0.06
D11Wsu47e	3.687	2.489	-0.52	-0.06

1810011H11Rik	0.365	0.165	-0.85	-0.06
Lyrm1	1.866	1.240	-0.59	-0.06
Asic1	0.737	0.477	-0.59	-0.06
Taldo1	144.509	93.669	-0.48	-0.06
Jph4	0.779	0.506	-0.57	-0.06
Pdf	12.427	8.536	-0.52	-0.06
Nalcn	0.101	0.059	-0.69	-0.06
Tlx1	0.162	0.043	-1.07	-0.06
Timp4	4.340	2.972	-0.52	-0.06
Lactb	5.819	3.645	-0.52	-0.06
Mapk8ip2	0.058	0.012	-1.24	-0.06
Pop1	2.436	1.663	-0.57	-0.06
Gm21312	1.450	0.990	-0.53	-0.06
2310061I04Rik	13.377	8.743	-0.53	-0.07
Hadhb	60.351	35.884	-0.50	-0.07
Styk1	0.487	0.163	-0.67	-0.07
Acp5	19.078	11.372	-0.52	-0.07
Ccne2	5.605	3.791	-0.53	-0.07
1700049G17Rik	1.227	0.812	-0.55	-0.07
Btbd10	5.580	3.878	-0.50	-0.07
Polr3h	10.544	7.214	-0.54	-0.07
Prnd	9.930	6.469	-0.50	-0.07
Gm21304	1.484	1.014	-0.52	-0.07
Pdhx	12.055	7.933	-0.54	-0.07
Lag3	1.554	0.847	-0.60	-0.07
1110006O24Rik	1.512	0.889	-0.62	-0.07
Cnga2	0.032	0.000	-1.52	-0.07
Acads	32.283	20.304	-0.52	-0.07
Gm10941	0.771	0.403	-0.77	-0.07
Nudt7	3.440	2.152	-0.55	-0.07
Mrpl49	19.565	13.334	-0.50	-0.07
Klk1b3	0.993	0.596	-0.69	-0.07
Cyp2a4	0.063	0.000	-1.49	-0.07

Qrsl1	3.646	2.304	-0.62	-0.07
Dhrs4	13.526	9.091	-0.56	-0.07
AA413626	0.320	0.055	-1.31	-0.07
Foxa3	0.208	0.022	-1.26	-0.07
Gm20878	7.844	5.202	-0.57	-0.07
Tpsg1	5.876	3.820	-0.54	-0.07
Epha7	0.906	0.571	-0.52	-0.07
Cbln3	0.165	0.089	-0.70	-0.07
Snhg7	6.956	4.455	-0.59	-0.07
Wisp2	0.654	0.348	-0.75	-0.07
Zglp1	0.298	0.153	-0.83	-0.07
Msx1os	3.271	2.175	-0.58	-0.07
Serpini1	0.664	0.443	-0.60	-0.07
Aldh1b1	2.366	1.216	-0.59	-0.07
Polr2l	18.679	13.293	-0.52	-0.07
Xlr	0.252	0.131	-0.79	-0.07
2310034G01Rik	0.839	0.447	-0.77	-0.07
Pgp	17.292	11.829	-0.52	-0.07
D930048N14Rik	2.076	1.414	-0.54	-0.08
1110001J03Rik	32.358	21.086	-0.52	-0.08
Alkbh4	4.171	2.727	-0.57	-0.08
Slco5a1	0.401	0.261	-0.58	-0.08
Tshr	4.429	2.559	-0.55	-0.08
2810001G20Rik	6.582	4.476	-0.53	-0.08
Esr1	1.989	1.265	-0.55	-0.08
Aldh1a1	6.943	4.240	-0.53	-0.08
Gm15760	0.635	0.416	-0.60	-0.08
Cox10	5.360	3.583	-0.55	-0.08
Tmem97	22.786	15.242	-0.52	-0.08
Hist1h3e	6.715	4.642	-0.55	-0.08
Xlr4a	2.546	1.480	-0.64	-0.08
Nod2	0.759	0.496	-0.59	-0.08

Lrrc24	0.692	0.440	-0.62	-0.08
N6amt2	18.516	12.283	-0.54	-0.08
Ciapin1	4.125	2.669	-0.56	-0.08
Wbscr25	0.135	0.021	-1.31	-0.08
Ell3	2.541	1.672	-0.58	-0.08
Gpr149	0.092	0.013	-1.12	-0.08
Dkk4	0.446	0.218	-0.77	-0.08
Gm20765	1.450	0.990	-0.56	-0.08
Cyp1a1	0.309	0.174	-0.69	-0.08
A430046D13Rik	3.027	2.001	-0.55	-0.08
1810022K09Rik	42.839	28.850	-0.56	-0.08
Lin28a	0.046	0.013	-1.14	-0.08
Traip	3.726	2.320	-0.62	-0.08
Ociad2	1.920	1.221	-0.59	-0.08
Etfa	98.981	57.091	-0.58	-0.08
Akr1b7	1.336	0.759	-0.67	-0.08
Mapk10	0.158	0.089	-0.71	-0.09
Gcsam	1.004	0.622	-0.61	-0.09
Slc12a1	0.039	0.009	-1.27	-0.09
4930483K19Rik	1.460	0.873	-0.60	-0.09
Aco2	133.423	80.691	-0.51	-0.09
Atp5j2	355.753	239.390	-0.53	-0.09
Fam78a	1.409	0.868	-0.61	-0.09
Cib2	24.845	15.629	-0.61	-0.09
4933427G17Rik	0.128	0.047	-0.94	-0.09
Tmem56	0.402	0.262	-0.58	-0.09
Coq3	6.375	4.090	-0.52	-0.09
Gm1821	6143.350	#######	-0.58	-0.09
Bcl2l14	0.613	0.288	-0.69	-0.09
Cdh17	1.106	0.229	-0.66	-0.09
Tmigd1	0.827	0.500	-0.68	-0.09

Gm4477	0.329	0.121	-0.96	-0.09
Tecrl	0.934	0.474	-0.65	-0.09
Tnni1	47.806	28.999	-0.55	-0.09
Fam98a	10.440	6.985	-0.52	-0.09
Adamdec1	1.108	0.174	-0.78	-0.09
Sc5d	11.791	8.093	-0.51	-0.09
Mamld1	1.513	0.918	-0.62	-0.09
Klk1b27	1.054	0.596	-0.70	-0.09
Srebf1	56.774	34.071	-0.54	-0.09
Adora3	3.699	2.629	-0.51	-0.10
Fgf12	0.119	0.059	-0.82	-0.10
Ndufaf2	15.434	10.532	-0.57	-0.10
Pank1	2.191	1.469	-0.55	-0.10
Nkx2-3	0.474	0.104	-0.87	-0.10
Siglecf	0.180	0.088	-0.84	-0.10
Cnksr2	0.199	0.110	-0.70	-0.10
Nxpe2	0.284	0.111	-0.79	-0.10
Cox7b	168.150	108.417	-0.54	-0.10
4933400A11Rik	0.118	0.000	-1.68	-0.10
Gm12709	0.121	0.046	-1.05	-0.10
Tmem199	6.286	4.086	-0.61	-0.10
Ceacam2	4.327	1.175	-0.58	-0.10
Gm21586	7.844	5.202	-0.56	-0.10
Crip3	1.485	0.913	-0.69	-0.10
Aunip	2.731	1.839	-0.55	-0.10
Cyb5b	42.786	26.629	-0.55	-0.10
Ifltd1	0.207	0.086	-0.98	-0.10
2310068J16Rik	1.094	0.688	-0.66	-0.10
Exoc8	1.797	1.110	-0.59	-0.10
Nol10	5.182	3.646	-0.50	-0.10
Lctl	2.335	1.502	-0.61	-0.10
Mcpt9	27.884	17.783	-0.53	-0.10
Tarm1	0.337	0.138	-0.99	-0.10
Lin7a	1.066	0.648	-0.60	-0.10

Tmem151b	0.402	0.213	-0.66	-0.10		
Sh2b2	7.458	3.938	-0.54	-0.10		
Slc45a3	7.781	5.108	-0.55	-0.10		
Scin	0.712	0.272	-0.74	-0.10		
Gm9866	0.070	0.000	-1.58	-0.11		
Tmem45a2	0.086	0.014	-1.30	-0.11		
1700042O10Rik	0.560	0.266	-0.93	-0.11		
Smim10l2a	0.984	0.590	-0.69	-0.11		
Acaa2	103.720	64.991	-0.51	-0.11		
Sod2	21.614	13.605	-0.51	-0.11	+	+
Cyp3a41b	0.065	0.000	-1.60	-0.11		
Cdhr5	1.172	0.176	-0.82	-0.11		
Wfikkn2	1.400	0.741	-0.60	-0.11		
Spata24	4.622	2.911	-0.56	-0.11		
Ctcflos	0.684	0.298	-0.66	-0.11		
Gm867	1.502	0.825	-0.72	-0.11		
Brpf3	6.659	4.541	-0.54	-0.11		
Glyat	0.062	0.000	-1.51	-0.11		
Gm5136	0.703	0.395	-0.78	-0.11		
Slc17a3	0.029	0.000	-1.66	-0.11		
4933416E03Rik	0.200	0.059	-1.20	-0.11		
Kpna2	77.321	51.791	-0.54	-0.11		
Cdkl3	1.280	0.848	-0.55	-0.11		
Morn2	4.554	3.099	-0.58	-0.11		
Ndufb2	93.047	61.216	-0.56	-0.11		
Ofcc1	0.756	0.203	-0.68	-0.11		
Lsm6	16.006	11.032	-0.54	-0.11		
Asgr1	0.080	0.000	-1.61	-0.11		
Mogat2	0.319	0.162	-0.83	-0.11		
Tfrc	8.997	5.605	-0.59	-0.12		
Ccdc73	1.381	0.916	-0.60	-0.12		
Hmgcll1	0.131	0.055	-0.94	-0.12		
A930003A15Rik	2.222	1.430	-0.61	-0.12		

4933411E08Rik	0.054	0.000	-1.68	-0.12
Syndig1	0.670	0.365	-0.70	-0.12
Coq5	12.758	7.896	-0.57	-0.12
Neu2	0.638	0.350	-0.70	-0.12
4833415N18Rik	0.860	0.485	-0.78	-0.12
Gm3558	0.068	0.000	-1.83	-0.12
Wfdc3	0.125	0.000	-1.49	-0.12
Slc1a6	0.193	0.084	-0.94	-0.12
Six4	0.417	0.257	-0.66	-0.12
Upk1b	0.988	0.461	-0.68	-0.12
Slc25a20	16.996	10.740	-0.55	-0.12
1110019D14Rik	1.016	0.559	-0.68	-0.12
Bola3	66.965	42.866	-0.56	-0.12
A330041J22Rik	0.776	0.347	-0.77	-0.12
Nxnl1	0.401	0.198	-0.72	-0.12
Krt72	8.696	2.932	-0.62	-0.12
Awat2	0.944	0.415	-0.71	-0.12
Dhcr24	18.467	11.849	-0.58	-0.12
Cox8b	71.482	41.340	-0.53	-0.12
Nudt15	1.784	1.097	-0.58	-0.12
Gm13152	2.528	1.473	-0.65	-0.12
Plin5	2.318	0.886	-0.65	-0.12
Wdhd1	4.811	3.215	-0.52	-0.12
Adap1	1.968	1.176	-0.68	-0.12
Apoa4	0.045	0.000	-1.59	-0.12
Aifm1	15.433	9.752	-0.60	-0.12
2210406O10Rik	0.164	0.078	-0.88	-0.12
Adam11	2.317	1.227	-0.64	-0.12
Cbx8	5.365	3.354	-0.63	-0.12
Slc16a4	0.263	0.141	-0.79	-0.13
Npff	2.334	1.385	-0.68	-0.13

Gsg1	0.195	0.089	-0.97	-0.13
Sertm1	0.042	0.000	-1.74	-0.13
Sgk2	0.051	0.007	-1.45	-0.13
Gm6525	2.795	1.625	-0.69	-0.13
Tyw3	1.892	1.211	-0.65	-0.13
Guca1b	0.190	0.066	-1.11	-0.13
Phex	0.082	0.035	-0.89	-0.13
E130006D01Rik	0.127	0.024	-1.34	-0.13
Uqcrq	255.442	163.258	-0.60	-0.13
Oc90	0.063	0.000	-1.52	-0.13
4930554I06Rik	0.173	0.000	-1.66	-0.13
Ccdc125	1.596	1.043	-0.60	-0.13
Gabra4	0.270	0.107	-0.78	-0.13
Lbx2	0.276	0.081	-1.13	-0.13
2700081O15Rik	18.750	11.453	-0.61	-0.13
C030034L19Rik	0.082	0.028	-1.15	-0.13
Slco1b2	0.033	0.000	-1.68	-0.14
Ppef2	0.066	0.010	-1.47	-0.14
Rasl12	3.422	1.995	-0.60	-0.14
Dido1	2.768	1.783	-0.57	-0.14
LOC101055863	0.031	0.000	-1.56	-0.14
Izumo1	0.245	0.103	-0.96	-0.14
Bcs1l	6.432	4.324	-0.57	-0.14
Gm10012	282.349	185.526	-0.57	-0.14
Polr2k	20.596	12.954	-0.63	-0.14
Leng8	20.783	13.622	-0.61	-0.14
Ctcfl	0.124	0.048	-0.91	-0.14
Adam28	0.029	0.000	-1.55	-0.14
Ager	2.411	1.530	-0.65	-0.14
Speer8-ps1	0.197	0.039	-1.33	-0.14
Zfp101	4.199	2.622	-0.63	-0.14
Slc25a1	73.274	40.316	-0.60	-0.14

Rec114	0.676	0.324	-0.90	-0.14
Rundc3b	0.686	0.432	-0.65	-0.14
Cntn3	0.443	0.217	-0.66	-0.14
9030625G05Rik	0.123	0.000	-1.70	-0.14
Gm16973	4.007	2.609	-0.59	-0.15
Luc7l3	20.566	12.970	-0.61	-0.15
Pipox	0.346	0.174	-0.87	-0.15
3110039I08Rik	5.642	3.309	-0.57	-0.15
Ephb1	1.318	0.836	-0.61	-0.15
Crabp1	35.760	22.338	-0.59	-0.15
Aqp12	0.109	0.000	-1.69	-0.15
Nxf7	0.491	0.272	-0.84	-0.15
Kcnq1ot1	0.125	0.077	-0.60	-0.15
Pin4	23.409	14.567	-0.67	-0.15
Mtfp1	3.536	1.894	-0.62	-0.15
Trim40	0.078	0.000	-1.59	-0.15
Zbtb48	3.903	2.524	-0.60	-0.15
E330013P04Rik	0.665	0.383	-0.71	-0.15
Slc10a5	0.037	0.000	-1.76	-0.15
Slc24a3	3.414	1.932	-0.62	-0.15
Mir7079	7.013	3.194	-0.99	-0.15
S100a1	46.380	27.231	-0.61	-0.15
Pif1	2.142	1.341	-0.63	-0.15
Purg	5.191	3.204	-0.61	-0.15
2810454H06Rik	0.514	0.304	-0.74	-0.15
Mir7026	7.784	3.618	-0.97	-0.15
Akr1c12	2.455	1.146	-0.66	-0.15
Cpt2	20.578	11.721	-0.61	-0.15
Olfr920	0.255	0.112	-0.90	-0.15
Myh15	0.204	0.021	-1.04	-0.15
Cntnap4	0.164	0.032	-0.98	-0.15
Gm6592	0.255	0.000	-1.70	-0.15
Gm5771	0.137	0.000	-1.81	-0.16

Rbmx	18.242	11.633	-0.60	-0.16
Snhg3	21.839	14.018	-0.66	-0.16
Hoxb9	6.887	4.164	-0.63	-0.16
Nme5	0.636	0.319	-0.94	-0.16
Vtcn1	0.168	0.050	-0.95	-0.16
3000002C10Rik	1.216	0.655	-0.78	-0.16
Spata5l1	2.340	1.518	-0.64	-0.16
Evx1	0.121	0.015	-1.31	-0.16
Gm10069	7.135	4.754	-0.59	-0.16
Rasef	0.188	0.072	-0.90	-0.16
Ncbp2	26.723	16.649	-0.65	-0.16
Mir6951	11.829	6.758	-0.78	-0.16
Nox1	2.140	1.290	-0.65	-0.16
B230119M05Rik	0.264	0.090	-1.14	-0.16
Vwa5b2	0.128	0.065	-0.82	-0.16
Clstn3	3.469	1.639	-0.64	-0.16
Ttbk1	0.014	0.000	-1.74	-0.16
Acly	42.818	23.539	-0.68	-0.16
Idh3a	51.488	30.340	-0.59	-0.16
Slc25a31	0.102	0.021	-1.22	-0.16
Chsy3	1.449	0.795	-0.68	-0.16
Ppp1r3b	17.022	8.096	-0.59	-0.16
Gm11715	6.075	3.618	-0.68	-0.17
Gdf7	0.096	0.000	-1.71	-0.17
Nrsn2	0.210	0.032	-1.38	-0.17
Rgn	0.437	0.196	-0.90	-0.17
Prdm15	1.905	1.236	-0.62	-0.17
Abcg5	0.254	0.039	-1.11	-0.17
Rap1gap	1.435	0.571	-0.67	-0.17
Asic3	0.114	0.023	-1.32	-0.17
Arg1	2.916	0.850	-0.73	-0.17
4930452G13Rik	0.119	0.000	-1.82	-0.17
Usmg5	56.022	35.800	-0.59	-0.17

Dbi	595.587	318.408	-0.71	-0.17
Tbx22	0.045	0.006	-1.50	-0.17
Gm15217	0.527	0.229	-1.02	-0.17
Lrrtm3	0.505	0.292	-0.79	-0.17
Klf1	0.329	0.158	-0.86	-0.17
Adam4	0.171	0.066	-1.05	-0.17
Fcrla	0.087	0.013	-1.48	-0.17
Iglon5	1.713	1.047	-0.70	-0.17
Dtwd2	1.269	0.827	-0.64	-0.17
Tcte3	1.162	0.557	-0.89	-0.17
Ndufb6	105.279	65.818	-0.61	-0.18
Col9a3	3.212	1.657	-0.71	-0.18
Htr3b	0.067	0.000	-1.73	-0.18
Nnat	7.566	4.470	-0.63	-0.18
Cldn3	5.436	1.047	-0.74	-0.18
Uxt	4.383	2.672	-0.69	-0.18
Gne	3.746	2.263	-0.66	-0.18
Nrg4	3.009	1.840	-0.61	-0.18
Mir3101	2.627	1.002	-1.11	-0.18
Fga	0.139	0.026	-1.19	-0.18
Gm14322	1.902	1.140	-0.63	-0.18
Cisd3	30.753	18.145	-0.60	-0.18
Atp1b1	11.825	4.926	-0.66	-0.18
Aspa	2.284	1.319	-0.65	-0.18
Hist1h1t	0.132	0.000	-1.64	-0.18
Krt7	31.528	18.701	-0.62	-0.18
Col9a1	1.861	1.218	-0.59	-0.18
Eif2s3y	6.223	0.798	-0.77	-0.18
Apoo	10.894	6.763	-0.65	-0.19
Pla2g4c	0.259	0.133	-0.82	-0.19
Acaca	17.242	9.412	-0.66	-0.19
Muc20	0.059	0.010	-1.33	-0.19
Rfx8	0.151	0.055	-1.10	-0.19
Clec18a	0.056	0.000	-1.76	-0.19
Dscc1	2.768	1.717	-0.68	-0.19
Ildr1	0.624	0.188	-0.87	-0.19

AA467197	6.561	0.448	-1.04	-0.19
Camk2n2	1.527	0.859	-0.80	-0.19
Clec2f	0.188	0.000	-1.73	-0.19
Tfr2	0.284	0.161	-0.76	-0.19
Catsperg2	0.385	0.228	-0.75	-0.19
Vgf	0.118	0.043	-1.08	-0.19
4930412C18Rik	0.358	0.189	-0.79	-0.19
Dgcr8	7.745	4.814	-0.67	-0.19
Hist1h4j	19.180	10.947	-0.71	-0.19
Gm3448	1.296	0.689	-0.75	-0.19
5830418P13Rik	0.397	0.212	-0.79	-0.19
Tssk4	0.670	0.357	-0.81	-0.19
Unc5a	2.583	1.364	-0.65	-0.19
Dnajc15	31.953	19.281	-0.67	-0.19
D3Ertd751e	1.544	0.889	-0.71	-0.19
Prss2	0.111	0.000	-1.55	-0.20
C4bp	0.063	0.000	-1.56	-0.20
Cml2	0.097	0.034	-1.17	-0.20
Hoxd4	3.047	1.860	-0.68	-0.20
2610044O15Rik8	6.251	3.873	-0.67	-0.20
Lcat	1.492	0.874	-0.74	-0.20
Trmt1l	7.073	4.234	-0.65	-0.20
Alms1-ps2	15.041	9.756	-0.63	-0.20
Mir7116	53.460	31.165	-0.69	-0.20
Poln	0.836	0.492	-0.70	-0.20
Akr1c13	3.125	1.485	-0.67	-0.20
Ms4a2	2.626	1.597	-0.69	-0.20
Lrr1	2.421	1.483	-0.75	-0.20
Lix1	1.224	0.703	-0.74	-0.20
Ndufa4	572.073	350.690	-0.67	-0.20
3010001F23Rik	0.944	0.441	-0.86	-0.20
Prss1	0.140	0.000	-1.77	-0.20

Pitpnm2os1	0.113	0.027	-1.22	-0.20
Klk14	0.504	0.087	-1.17	-0.20
Fam209	0.431	0.136	-1.24	-0.21
6430584L05Rik	0.038	0.000	-1.62	-0.21
Xlr4c	2.159	1.207	-0.71	-0.21
Fabp4	2587.820	#######	-0.64	-0.21
Stra6	0.213	0.042	-1.05	-0.21
Htr7	0.736	0.408	-0.78	-0.21
Tnfrsf13b	0.280	0.059	-1.39	-0.21
Fam195a	20.841	10.559	-0.67	-0.21
Nme9	0.115	0.000	-1.76	-0.21
Lss	5.965	3.649	-0.66	-0.21
Serpina1e	1.978	0.770	-0.75	-0.21
Apob	0.027	0.006	-1.19	-0.21
Ndufaf4	4.859	2.979	-0.71	-0.21
Elavl3	0.073	0.026	-1.04	-0.21
Cpa6	0.754	0.329	-0.83	-0.21
Kcnk10	0.034	0.000	-1.67	-0.21
2610318N02Rik	1.185	0.644	-0.79	-0.21
Clhc1	0.076	0.020	-1.26	-0.21
A830082K12Rik	0.564	0.296	-0.81	-0.21
5031425F14Rik	0.289	0.094	-1.07	-0.21
Ces2d-ps	1.150	0.595	-0.81	-0.22
Idi1	34.747	21.129	-0.70	-0.22
Tmem200a	1.117	0.659	-0.70	-0.22
Asphd2	3.210	1.917	-0.69	-0.22
5730457N03Rik	0.052	0.000	-1.82	-0.22
C330018D20Rik	3.064	1.826	-0.72	-0.22
Rdh7	0.202	0.041	-1.28	-0.22

Mgam	0.333	0.082	-0.88	-0.22
A230072C01Rik	1.048	0.601	-0.71	-0.22
B3gat1	0.110	0.045	-1.03	-0.22
Gm8234	0.677	0.364	-0.84	-0.22
Mir692-3	42.422	23.249	-0.79	-0.22
Me1	38.668	20.890	-0.69	-0.22
Ppcs	6.530	3.887	-0.69	-0.22
Rab26os	11.118	6.349	-0.75	-0.22
Gpd1	219.451	82.303	-0.68	-0.22
Snord49a	3.382	0.711	-1.36	-0.22
Mir1306	4.492	1.674	-1.12	-0.22
Gins1	7.223	4.392	-0.70	-0.23
11-Mar	0.073	0.000	-1.71	-0.23
1700039E15Rik	0.062	0.000	-1.82	-0.23
Gm14288	7.223	3.913	-0.76	-0.23
Mvd	11.576	7.081	-0.69	-0.23
Hist2h2ac	26.010	14.031	-0.70	-0.23
Cpn1	0.060	0.000	-1.73	-0.23
B430212C06Rik	0.590	0.336	-0.77	-0.23
Mir5114	155.836	80.195	-0.78	-0.23
Timm9	13.757	8.404	-0.67	-0.23
Erich6	0.069	0.010	-1.52	-0.23
Gp9	0.312	0.109	-1.10	-0.23
Lbx1	1.537	0.714	-0.89	-0.23
BC025920	1.383	0.804	-0.74	-0.23
Clk4	15.927	9.412	-0.64	-0.23
Snora64	7.858	4.143	-0.84	-0.23
Cldn6	0.920	0.249	-0.89	-0.23
Olfr43	0.143	0.000	-1.63	-0.24
Cd209d	0.168	0.026	-1.39	-0.24
Myog	24.239	14.249	-0.71	-0.24
Gm3417	1.296	0.689	-0.78	-0.24
Pfkfb1	4.740	2.408	-0.74	-0.24

Rtn4ip1	3.322	1.929	-0.70	-0.24
Marveld3	0.738	0.378	-0.79	-0.24
Apex2	4.475	2.700	-0.73	-0.24
Topaz1	0.137	0.053	-0.97	-0.24
Aacs	23.822	11.764	-0.75	-0.24
Tusc5	10.416	4.404	-0.81	-0.24
Pcp4l1	6.199	3.694	-0.72	-0.24
Cyp2b23	0.835	0.434	-0.83	-0.24
Rprm	3.591	2.188	-0.73	-0.24
Wnt5b	2.299	1.387	-0.73	-0.24
2900076A07Rik	4.432	2.684	-0.73	-0.24
A230057D06Rik	0.188	0.055	-1.16	-0.25
Cldn22	0.346	0.089	-1.23	-0.25
9230102K24Rik	0.528	0.254	-0.91	-0.25
Hmga2-ps1	3.992	2.320	-0.74	-0.25
Dlx6os1	0.067	0.010	-1.36	-0.25
Cxcr5	0.849	0.463	-0.80	-0.25
Col24a1	1.009	0.577	-0.71	-0.25
Zfp566	2.027	1.185	-0.75	-0.25
Tcerg1l	0.653	0.086	-1.07	-0.25
Car5a	0.078	0.000	-1.61	-0.25
Kcnh4	0.054	0.012	-1.30	-0.25
Col8a2	3.873	1.399	-0.73	-0.25
Lrrc31	0.051	0.000	-1.53	-0.25
Bfsp2	0.346	0.140	-1.06	-0.26
A330048O09Rik	0.250	0.033	-1.58	-0.26
Msantd1	0.462	0.245	-0.84	-0.26
Lrrc4c	0.356	0.181	-0.84	-0.26
Insrr	0.043	0.009	-1.33	-0.26
Glis1	3.242	1.912	-0.75	-0.26
Apba2	0.537	0.225	-0.87	-0.26
Itgb2l	0.039	0.000	-1.75	-0.26

Kcng1	0.172	0.057	-1.18	-0.26
Lrrc63	0.053	0.000	-1.65	-0.26
Thrsp	115.972	44.560	-0.72	-0.26
Noxo1	1.708	0.926	-0.76	-0.26
Cck	0.271	0.077	-1.29	-0.26
Pld6	0.214	0.067	-1.13	-0.26
Klk1b21	1.500	0.659	-0.87	-0.26
Rragb	0.226	0.087	-1.05	-0.26
Alb	2.887	1.068	-0.79	-0.26
Cytl1	0.381	0.090	-1.25	-0.27
Dph2	2.863	1.701	-0.74	-0.27
Mmp8	0.443	0.234	-0.86	-0.27
Zfp658	1.322	0.776	-0.74	-0.27
Fam161b	0.513	0.276	-0.82	-0.27
Aldh1a7	24.558	12.408	-0.70	-0.27
B3galt5	1.015	0.248	-0.84	-0.27
Itpka	1.532	0.707	-0.81	-0.27
2500004C02Rik	1.490	0.836	-0.75	-0.27
Sarm1	0.220	0.115	-0.90	-0.27
Fdps	43.466	25.354	-0.75	-0.27
Mmp10	0.062	0.000	-1.75	-0.27
Upk1a	1.454	0.274	-0.99	-0.27
Mep1b	0.071	0.000	-1.70	-0.27
1700007G11Rik	0.137	0.000	-1.73	-0.27
Grin3a	0.242	0.106	-0.83	-0.27
6330409D20Rik	0.327	0.118	-1.14	-0.27
Pik3r3	2.817	1.651	-0.70	-0.27
Bcl2l10	1.003	0.413	-0.86	-0.28
Pcyt2	27.229	16.031	-0.70	-0.28
C2cd4b	0.154	0.033	-1.46	-0.28
4933417D19Rik	0.332	0.108	-1.17	-0.28
Tbx3os2	0.099	0.000	-1.79	-0.28

6430710C18Rik	0.058	0.000	-1.84	-0.28
Scd3	46.528	20.740	-0.71	-0.28
Chad	0.429	0.093	-1.15	-0.28
Tm6sf2	0.212	0.076	-1.19	-0.28
Tstd1	2.059	0.760	-0.96	-0.28
AI450353	0.882	0.426	-0.92	-0.28
Scd2	205.116	111.092	-0.69	-0.28
Catsperg1	0.868	0.488	-0.77	-0.28
C1ql2	0.245	0.033	-1.28	-0.28
Dner	0.321	0.170	-0.91	-0.29
P2rx5	1.732	0.896	-0.76	-0.29
Ngef	0.758	0.391	-0.86	-0.29
1500015A07Rik	2.019	1.182	-0.73	-0.29
Doc2g	3.125	1.719	-0.82	-0.29
Faiml	0.514	0.168	-1.21	-0.29
Csmd2	0.007	0.000	-1.71	-0.29
Hpx	0.509	0.194	-1.04	-0.29
Mab21l1	1.605	0.270	-0.88	-0.29
Slc29a4	0.553	0.253	-0.96	-0.30
Gm1965	0.053	0.000	-1.70	-0.30
Hoxa10	4.234	1.985	-0.76	-0.30
9130409J20Rik	0.241	0.029	-1.48	-0.30
Ndp	2.095	1.217	-0.78	-0.30
Sfpq	59.131	34.520	-0.73	-0.30
Mir6922	57.189	28.379	-0.81	-0.30
Gm4301	1.606	0.844	-0.85	-0.30
1700003M07Rik	0.513	0.247	-0.96	-0.30
Hgfac	1.186	0.231	-0.97	-0.31
Dzank1	0.179	0.077	-0.98	-0.31
Slc26a8	0.100	0.030	-1.30	-0.31
Nr1i2	0.462	0.183	-0.93	-0.31

9230105E05Rik	0.103	0.029	-1.25	-0.31
1810065E05Rik	0.221	0.000	-1.88	-0.31
Mir692-2	42.422	23.249	-0.78	-0.31
Spz1	0.111	0.000	-1.75	-0.31
Ceacam1	9.359	2.365	-0.78	-0.31
4930557K07Rik	1.173	0.426	-1.17	-0.31
Dapk2	1.167	0.623	-0.88	-0.31
Igfbpl1	0.055	0.000	-1.98	-0.31
Snord72	3.316	0.735	-1.42	-0.31
Dmgdh	0.040	0.000	-1.77	-0.31
Hsd11b2	3.897	2.014	-0.84	-0.32
Il13	0.290	0.091	-1.28	-0.32
Retnlg	1.105	0.471	-1.03	-0.32
Clec2g	1.755	0.961	-0.83	-0.32
6820408C15Rik	0.096	0.013	-1.55	-0.32
Abhd3	0.436	0.187	-1.02	-0.32
Dqx1	0.575	0.286	-0.93	-0.32
Rbp7	11.324	6.084	-0.83	-0.32
Hoxc11	1.011	0.169	-1.06	-0.32
Hist1h4d	10.432	5.629	-0.82	-0.32
Dcpp3	0.212	0.000	-1.77	-0.32
Dmrta2	0.161	0.060	-1.12	-0.32
Gm5868	0.351	0.096	-1.26	-0.32
Gm20597	0.086	0.032	-1.10	-0.33
3110040N11Rik	13.890	7.894	-0.78	-0.33
Nkain1	3.245	1.779	-0.81	-0.33
Clec12a	0.203	0.069	-1.19	-0.33
Prdm12	0.057	0.000	-1.84	-0.33
Gipc2	1.340	0.331	-0.96	-0.33
Muc19	0.193	0.097	-0.92	-0.33
Gm5485	0.351	0.000	-1.87	-0.33

Olfr224	0.518	0.210	-1.14	-0.33		
Shisa6	0.046	0.012	-1.32	-0.33		
Mthfd2	7.953	3.832	-0.80	-0.33	+	
Tmprss2	2.004	0.181	-1.00	-0.33		
B130034C11Rik	0.417	0.157	-1.18	-0.34		
Rdh18-ps	0.175	0.054	-1.07	-0.34		
Foxd2	2.252	0.858	-0.84	-0.34		
Mir6973b	4.234	1.377	-1.25	-0.34		
Gm5176	0.270	0.000	-1.94	-0.34		
Rpp25	0.348	0.131	-1.16	-0.34		
Tox3	0.901	0.449	-0.86	-0.34		
Smim22	0.868	0.185	-1.31	-0.34		
Dbx1	0.186	0.064	-1.18	-0.34		
Uty	1.027	0.325	-0.78	-0.34		
Car15	0.973	0.459	-0.97	-0.34		
Tbx3	5.402	2.314	-0.86	-0.34		
G0s2	108.472	32.439	-0.78	-0.34		+
Gm16853	0.847	0.422	-0.98	-0.34		
Gad2	0.043	0.012	-1.31	-0.34		
Als2cr12	0.614	0.238	-1.04	-0.35		
Myo5c	0.435	0.160	-0.89	-0.35		
Ptchd4	0.156	0.077	-0.94	-0.35		
Slc13a2os	0.061	0.000	-1.84	-0.35		
Gc	0.154	0.024	-1.47	-0.35		
Serpinc1	0.254	0.102	-1.10	-0.35		
Gjc2	1.231	0.645	-0.84	-0.35		
Faim2	0.053	0.009	-1.50	-0.35		
Parp11	2.608	1.355	-0.86	-0.36		
Sqle	20.513	11.884	-0.75	-0.36		
9530062K07Rik	0.322	0.084	-1.39	-0.36		
Gabra5	0.032	0.000	-1.58	-0.36		
Cutal	0.327	0.047	-1.27	-0.36		
Ascl2	0.634	0.273	-1.01	-0.36		
Syt8	0.394	0.164	-1.08	-0.36		

Cycs	143.591	73.700	-0.84	-0.36	
Sprr2h	4.938	2.507	-0.86	-0.37	
Kdm5d	0.896	0.117	-1.01	-0.37	
Slc38a5	0.084	0.000	-1.83	-0.37	
Lct	0.054	0.018	-1.26	-0.37	
Zfc3h1	4.643	2.493	-0.84	-0.37	
Tmem120a	23.741	12.121	-0.83	-0.37	
Cyp51	15.920	8.570	-0.82	-0.37	
Slc4a9	0.983	0.475	-0.92	-0.37	
Hottip	0.086	0.000	-1.93	-0.37	
Trpc4	0.126	0.049	-1.15	-0.37	
Hepacam2	0.974	0.148	-1.04	-0.37	
Tat	0.070	0.000	-1.96	-0.37	
Adra1b	0.087	0.025	-1.30	-0.37	
Eaf2	0.384	0.162	-1.14	-0.37	
Gckr	0.051	0.000	-1.77	-0.38	
Clk1	26.335	13.951	-0.84	-0.38	
Snhg4	4.017	2.048	-0.89	-0.38	
Ccdc162	0.145	0.048	-1.27	-0.38	
Ifitm5	1.464	0.591	-1.05	-0.38	
AV051173	1.689	0.812	-0.99	-0.38	
Pcdhb12	0.209	0.087	-1.06	-0.38	
Kctd4	0.901	0.439	-0.89	-0.38	
Cxcl3	10.305	1.407	-0.93	-0.38	+
Naip2	0.921	0.409	-0.88	-0.39	
Disp2	0.093	0.033	-1.13	-0.39	
Nags	0.076	0.000	-1.86	-0.39	
Capsl	0.483	0.186	-1.20	-0.39	
Calcb	1.112	0.412	-1.10	-0.39	
Isl1	3.697	1.056	-0.94	-0.39	
Foxa2	0.236	0.019	-1.50	-0.39	
Fam163a	0.068	0.011	-1.56	-0.39	
Ssxb3	0.231	0.000	-1.85	-0.39	
Cyp2c67	0.123	0.000	-1.91	-0.39	
Best1	0.377	0.159	-1.11	-0.40	
5033428I22Rik	0.294	0.069	-1.46	-0.40	

Kcng4	0.031	0.000	-1.81	-0.40
Lurap1	1.270	0.618	-0.94	-0.40
Neto1	0.192	0.062	-1.20	-0.40
Adamtsl2	0.869	0.442	-0.93	-0.40
Tmprss7	0.129	0.034	-1.43	-0.40
Cd27	0.556	0.252	-1.03	-0.40
Hes5	1.191	0.398	-1.14	-0.40
Cyp3a41a	0.065	0.000	-1.88	-0.40
Tcf15	3.604	1.751	-0.90	-0.40
Slc22a2	0.136	0.031	-1.33	-0.40
Cend1	0.104	0.000	-2.03	-0.41
Enkur	0.906	0.386	-1.05	-0.41
Ttc39a	1.247	0.532	-0.95	-0.41
Kcnb2	0.272	0.133	-1.00	-0.41
Ddx25	0.565	0.250	-1.05	-0.41
Cd226	0.045	0.000	-1.89	-0.41
Kng2	0.060	0.000	-1.95	-0.41
Tm7sf2	7.892	4.351	-0.86	-0.42
Dbh	0.090	0.010	-1.68	-0.42
Atp12a	1.904	0.257	-0.99	-0.42
Fam19a3	0.093	0.031	-1.25	-0.42
Gm11757	0.079	0.000	-1.84	-0.42
Gypa	0.283	0.109	-1.13	-0.42
H2-M10.3	0.253	0.042	-1.56	-0.42
Cyp4a32	0.272	0.081	-1.29	-0.42
Fads2	13.998	7.438	-0.87	-0.42
Iqcf1	0.195	0.000	-1.80	-0.42
Igfbp2	30.571	12.687	-0.89	-0.42
Snord12	4.644	1.336	-1.35	-0.42
Odf3l1	2.459	1.163	-0.93	-0.42
Slc35f3	0.143	0.032	-1.33	-0.43
Galntl6	0.039	0.004	-1.62	-0.43
Klk1b1	0.197	0.000	-1.89	-0.43
Lace1	3.736	1.885	-0.93	-0.43
Fbxl21	0.252	0.090	-1.21	-0.43
Lincenc1	0.041	0.000	-1.96	-0.43

Sap25	7.134	3.828	-0.89	-0.43
Lrtm1	0.228	0.096	-1.03	-0.43
Misp	1.132	0.154	-1.08	-0.43
Tbx20	0.016	0.000	-1.85	-0.43
Oscar	0.070	0.000	-1.99	-0.43
Ppp1r1b	2.411	0.370	-1.06	-0.43
Ffar4	2.764	1.092	-0.92	-0.44
Neto2	0.688	0.324	-0.98	-0.44
Msmo1	27.414	14.618	-0.89	-0.44
Cdkn2a	0.459	0.175	-1.21	-0.44
Hbb-b2	4572.850	#######	-0.95	-0.44
Gm4307	1.668	0.799	-0.97	-0.45
Mss51	0.805	0.386	-1.03	-0.45
Tmprss11g	0.114	0.015	-1.60	-0.45
Nr1h4	0.087	0.000	-1.88	-0.45
Rnf138rt1	0.153	0.018	-1.66	-0.45
Slc44a4	0.903	0.066	-1.37	-0.45
Dcx	0.776	0.351	-0.99	-0.45
Slc25a35	2.210	1.037	-0.95	-0.45
Rep15	1.648	0.337	-1.15	-0.45
1700092M07Rik	0.439	0.058	-1.55	-0.45
Slc6a12	0.080	0.009	-1.70	-0.45
Plekhs1	0.153	0.046	-1.35	-0.45
Mycbpap	0.165	0.053	-1.28	-0.45
Zc3h12d	0.101	0.032	-1.32	-0.45
Dpp10	0.116	0.019	-1.35	-0.45
3110079O15Rik	0.314	0.000	-1.99	-0.46
Spaca1	0.590	0.229	-1.13	-0.46
Spata22	0.160	0.017	-1.61	-0.46
Gck	0.781	0.350	-1.03	-0.46
Gm4312	1.606	0.844	-0.92	-0.46
Fgg	0.169	0.014	-1.72	-0.46
Rsrp1	92.191	45.068	-0.91	-0.46

Gm5577	1.118	0.480	-1.06	-0.46
B130024G19Rik	0.763	0.328	-1.09	-0.47
Dancr	7.313	3.722	-0.90	-0.47
2900005J15Rik	0.619	0.249	-1.12	-0.47
Ppp1r3d	4.010	1.846	-0.96	-0.47
Plg	0.061	0.000	-1.96	-0.47
2010003K11Rik	1.695	0.510	-1.17	-0.48
Ces1f	2.378	0.782	-0.99	-0.48
Sgpp2	0.421	0.110	-1.19	-0.48
Prr18	0.101	0.031	-1.42	-0.48
Nsdhl	16.959	8.355	-0.94	-0.48
Nabp1	11.377	3.649	-0.94	-0.48
Fam196a	0.032	0.000	-2.07	-0.48
BB014433	0.070	0.000	-1.81	-0.49
Acnat1	0.033	0.000	-1.87	-0.49
Tbx5	1.278	0.453	-0.96	-0.49
Fgf9	0.414	0.176	-1.08	-0.49
Muc6	0.047	0.011	-1.43	-0.49
Snhg11	1.721	0.628	-0.93	-0.49
Cemip	0.227	0.108	-1.03	-0.49
Rgs13	0.898	0.412	-1.04	-0.49
Bmp15	0.039	0.000	-1.85	-0.49
Trim9	0.420	0.144	-1.07	-0.49
Tinag	0.378	0.103	-1.30	-0.50
Mia	0.749	0.117	-1.57	-0.50
Med9os	0.620	0.167	-1.39	-0.50
Hbb-bt	4572.850	#######	-0.94	-0.50
Gm4305	1.668	0.799	-1.03	-0.50
Cadps	0.097	0.028	-1.40	-0.50
Btc	2.056	1.004	-1.02	-0.50
Tubb1	0.126	0.022	-1.64	-0.50
Hils1	0.249	0.051	-1.67	-0.51

Ffar2	2.970	1.365	-0.95	-0.51	
Cxcl15	0.248	0.021	-1.62	-0.51	
Mir143hg	4.386	1.965	-0.98	-0.51	
A4gnt	0.160	0.032	-1.54	-0.51	
Sncg	8.543	3.522	-1.05	-0.51	
Acss2	19.307	8.525	-0.93	-0.51	
Apoa5	0.081	0.000	-2.01	-0.51	
Pirt	0.309	0.102	-1.19	-0.52	
1700016K19Rik	0.146	0.000	-2.08	-0.52	
Hbb-b1	4217.480	#######	-0.99	-0.52	
E030013I19Rik	0.132	0.040	-1.35	-0.52	
Tmc5	0.443	0.174	-1.06	-0.52	
Fam135b	0.028	0.000	-2.00	-0.52	
Kera	3.011	1.107	-1.04	-0.52	
Trhr2	0.096	0.000	-2.02	-0.52	
Soat2	0.250	0.093	-1.27	-0.52	
Coro2a	1.570	0.636	-1.02	-0.52	
Igsf5	0.179	0.010	-1.77	-0.52	
Hcn1	0.079	0.017	-1.36	-0.53	
Cyp2d10	0.118	0.000	-2.01	-0.53	
Chil6	0.087	0.000	-2.00	-0.53	
2700038G22Rik	1.480	0.659	-1.13	-0.53	
Ldlr	15.148	7.695	-0.98	-0.53	+
Scd4	25.861	9.827	-0.98	-0.53	
Camp	7.818	3.495	-1.03	-0.54	
Gdap1	0.086	0.022	-1.42	-0.54	
Naip5	0.748	0.308	-1.06	-0.54	
Adgrb1	0.123	0.042	-1.28	-0.54	
Chil4	0.253	0.058	-1.55	-0.54	
Wasf3	0.403	0.148	-1.23	-0.54	
Snhg20	15.915	7.676	-1.04	-0.55	
Gale	16.767	7.512	-1.02	-0.55	
Lppr1	0.505	0.223	-1.12	-0.55	

Gm4302	1.652	0.767	-1.06	-0.55
Tmprss11b	0.245	0.010	-1.93	-0.56
Onecut2	0.011	0.000	-1.97	-0.56
Tbx4	1.634	0.365	-1.15	-0.56
Sox8	0.945	0.404	-1.10	-0.56
Lep	2.841	0.947	-1.10	-0.56
Abcc2	0.103	0.033	-1.30	-0.56
Hbb-bs	4164.760	#######	-1.02	-0.56
Prr22	0.705	0.261	-1.27	-0.57
2610028H24Rik	0.665	0.275	-1.22	-0.57
Ucp3	2.515	1.179	-1.05	-0.57
Mat1a	0.082	0.000	-2.16	-0.57
Pdss1	4.157	1.954	-1.07	-0.57
Cyp2e1	3.048	1.282	-1.07	-0.57
Dio1	0.126	0.000	-1.98	-0.57
Ltf	0.351	0.121	-1.28	-0.57
Scgn	0.122	0.000	-2.05	-0.58
Agpat2	59.895	21.420	-1.07	-0.58
Gm13238	1.389	0.512	-1.29	-0.58
Myt1l	0.021	0.000	-2.02	-0.58
E030030I06Rik	1.271	0.529	-1.12	-0.58
Sall4	0.075	0.013	-1.67	-0.58
Klk1b5	2.498	0.614	-1.23	-0.58
BC049762	0.237	0.025	-1.87	-0.58
Clec12b	2.139	0.884	-1.14	-0.58
Slc6a13	1.413	0.646	-1.10	-0.58
2510003B16Rik	1.109	0.402	-1.28	-0.58
Slc10a1	0.067	0.000	-1.99	-0.58
Kcnrg	0.238	0.060	-1.45	-0.59
Gm4303	1.668	0.799	-1.07	-0.59
Lect1	0.378	0.107	-1.45	-0.59
Rps6kl1	0.286	0.096	-1.34	-0.59
Mbd6	26.030	12.243	-1.07	-0.60

Best2	0.520	0.040	-1.58	-0.60	
Acmsd	0.065	0.000	-2.08	-0.60	
Upp2	0.158	0.024	-1.59	-0.60	
Fam162b	0.300	0.000	-2.01	-0.61	
Nell2	0.513	0.132	-1.22	-0.61	
Cacng5	0.041	0.000	-2.01	-0.61	
4930433N12Rik	0.366	0.112	-1.43	-0.61	
BC048671	0.371	0.095	-1.52	-0.61	
Upk3bl	0.717	0.106	-1.53	-0.62	
Zfp804a	0.107	0.021	-1.66	-0.63	
Col9a2	3.378	1.260	-1.08	-0.63	
Fam159a	0.185	0.000	-2.06	-0.63	
Acrbp	1.935	0.767	-1.18	-0.63	
Gm3336	0.588	0.038	-1.75	-0.64	
Gm13871	0.079	0.000	-2.03	-0.64	
Car2	11.378	2.007	-1.16	-0.64	
Fmr1nb	0.191	0.000	-2.33	-0.64	
Gm6086	0.303	0.060	-1.47	-0.64	
Pax9	0.079	0.000	-2.09	-0.64	
Fxyd4	0.602	0.040	-1.88	-0.65	
2810405F15Rik	0.236	0.043	-1.65	-0.66	
Fam196b	0.128	0.021	-1.57	-0.66	
Ttc21a	0.036	0.000	-2.23	-0.66	
Vil1	1.621	0.233	-1.24	-0.66	
Tmprss11d	0.155	0.000	-2.20	-0.67	
Nrsn1	0.078	0.000	-2.08	-0.67	
Ngb	0.162	0.014	-1.98	-0.67	
Il23a	0.269	0.065	-1.61	-0.67	+
Myo15	0.234	0.024	-1.37	-0.67	
Oit1	5.072	0.176	-1.53	-0.68	
Cyp2c69	0.176	0.000	-2.25	-0.68	
Tnfrsf25	0.882	0.336	-1.31	-0.68	
Myo7b	0.225	0.077	-1.33	-0.68	
Ttc25	0.153	0.039	-1.66	-0.68	

Vwde	0.039	0.000	-2.06	-0.68
Patl2	0.106	0.009	-2.02	-0.69
Ceacam20	0.058	0.000	-2.11	-0.69
Camkv	0.218	0.058	-1.50	-0.70
1700102P08Rik	0.353	0.060	-1.82	-0.70
Pbp2	0.171	0.000	-2.35	-0.70
Rxfp1	0.090	0.000	-2.19	-0.70
Pnma1	0.356	0.103	-1.53	-0.70
Cyp2a5	0.100	0.000	-2.16	-0.70
Acss2os	3.889	1.261	-1.26	-0.71
Vipr1	0.416	0.099	-1.30	-0.71
Pllp	1.953	0.432	-1.27	-0.71
Iqcd	0.384	0.119	-1.47	-0.71
Sirpb1a	0.615	0.143	-1.54	-0.71
Mir147	11.774	0.558	-1.86	-0.71
Otx1	0.055	0.000	-2.17	-0.71
Slc26a1	0.083	0.014	-1.73	-0.71
Ptprn2	0.169	0.028	-1.60	-0.72
Gm11538	0.094	0.000	-2.15	-0.72
Zmynd10	0.123	0.013	-1.97	-0.72
Slc17a4	0.104	0.000	-2.09	-0.72
Pls1	0.391	0.083	-1.39	-0.73
Gpx2-ps1	0.597	0.108	-1.60	-0.73
Sim2	0.665	0.054	-1.47	-0.73
Hoxa9	5.698	2.389	-1.18	-0.73
Tmem95	0.405	0.041	-1.99	-0.74
Irs4	0.200	0.056	-1.41	-0.74
Srd5a2	0.416	0.000	-2.32	-0.74
Ugt2b5	0.122	0.000	-2.26	-0.75
Gnrh1	1.370	0.380	-1.57	-0.75
Ctla4	0.220	0.046	-1.67	-0.75
Ppp1r14d	0.285	0.000	-2.26	-0.76
Lgals6	35.835	5.111	-1.21	-0.76
Cdr1	0.077	0.000	-2.22	-0.76
Tmem236	0.281	0.024	-1.74	-0.77

Hotair	0.385	0.020	-1.91	-0.77
Mug-ps1	0.051	0.000	-2.23	-0.77
Dbil5	0.645	0.115	-1.78	-0.78
A530053G22Rik	0.156	0.000	-2.42	-0.78
Myo1a	0.856	0.085	-1.52	-0.79
Ngp	1.181	0.399	-1.41	-0.79
Isx	0.144	0.000	-2.26	-0.79
Ccdc30	0.102	0.023	-1.71	-0.79
Apol7a	0.090	0.000	-2.22	-0.80
Insig1	77.475	27.669	-1.26	-0.81
Clic6	0.497	0.106	-1.49	-0.81
Gm2083	14.614	5.390	-1.28	-0.81
Slc39a4	0.855	0.119	-1.54	-0.81
Klhl32	0.187	0.037	-1.84	-0.81
Il25	0.192	0.000	-2.32	-0.81
Olfr789	0.183	0.000	-2.26	-0.81
Jakmip3	0.042	0.000	-2.31	-0.82
C230035I16Rik	0.619	0.107	-1.82	-0.82
Mas1	0.113	0.018	-1.83	-0.82
Ccdc150	0.049	0.000	-2.31	-0.82
Gdf6	0.400	0.075	-1.57	-0.83
Angptl7	1.017	0.107	-1.50	-0.83
Ces1d	12.345	2.253	-1.30	-0.83
Bicd1	0.081	0.021	-1.58	-0.84
Jakmip2	0.093	0.013	-1.93	-0.84
Fasn	68.383	19.091	-1.31	-0.84
Hba-a1	7543.530	#######	-1.32	-0.84
Scd1	500.926	123.891	-1.26	-0.84
Cyp4a31	0.176	0.026	-1.96	-0.84
Tmem171	0.959	0.095	-1.76	-0.84
Tnfaip8l3	0.297	0.079	-1.66	-0.85
Krt18	7.024	1.007	-1.36	-0.85
Gria1	0.193	0.026	-1.67	-0.85
Mup6	4.610	1.387	-1.50	-0.85

Hsf5	0.047	0.000	-2.32	-0.85
Mab21l2	0.155	0.000	-2.27	-0.85
Lgals4	28.393	4.135	-1.31	-0.86
AW549542	1.711	0.162	-1.60	-0.87
Plekhd1	0.146	0.025	-1.77	-0.87
Pde4c	0.352	0.097	-1.60	-0.87
9530091C08Rik	0.023	0.000	-2.35	-0.88
Chrna3	0.076	0.000	-2.39	-0.88
Hba-a2	7491.430	#######	-1.33	-0.88
Klk1b4	0.513	0.000	-2.31	-0.88
Ankrd63	0.113	0.027	-1.75	-0.88
Gm6484	22.843	4.840	-1.37	-0.90
Btnl5-ps	0.236	0.000	-2.42	-0.90
Hnf4g	0.075	0.000	-2.37	-0.90
Gm11758	0.077	0.000	-2.30	-0.90
Pnpla3	4.995	1.417	-1.35	-0.90
A530050N04Rik	0.205	0.000	-2.32	-0.91
Olah	0.157	0.000	-2.35	-0.91
Alas2	5.983	2.194	-1.40	-0.91
Aknad1	0.143	0.025	-1.90	-0.91
St8sia1	0.091	0.022	-1.68	-0.91
Mup10	13.222	4.597	-1.37	-0.91
Mup19	9.180	3.137	-1.43	-0.92
Slc4a5	0.043	0.000	-2.47	-0.93
Cyp2c40	0.233	0.000	-2.42	-0.93
Cyp4a14	0.116	0.000	-2.53	-0.93
Erc2	0.144	0.029	-1.72	-0.94
Igflr1	3.350	1.096	-1.49	-0.94
4930404I05Rik	0.869	0.225	-1.73	-0.94
Gm2381	0.110	0.022	-1.82	-0.95
Lrrc26	0.753	0.037	-2.08	-0.95
Gal3st2	1.156	0.232	-1.56	-0.95
Mup7	11.820	3.904	-1.44	-0.95

Xlr5b	0.185	0.000	-2.50	-0.95
Gm4832	8.946	2.862	-1.40	-0.95
Crp	0.108	0.000	-2.32	-0.95
Zfp872	0.138	0.015	-2.04	-0.96
Sycn	1.158	0.000	-2.41	-0.96
Zg16	1.361	0.035	-2.23	-0.97
Il4	0.867	0.183	-1.80	-0.97
Hba-x	146.067	45.834	-1.45	-0.98
Slpi	0.503	0.050	-2.06	-0.98
Xlr5a	0.121	0.000	-2.48	-0.98
Mup13	9.120	2.911	-1.55	-0.98
Defb4	0.716	0.000	-2.37	-0.98
H2-M10.4	0.308	0.000	-2.59	-0.98
Mbl2	0.322	0.000	-2.38	-0.98
Pdzd3	0.118	0.010	-2.14	-0.99
Kcnj16	0.191	0.011	-2.12	-0.99
Mup17	6.117	2.015	-1.49	-1.00
Dyrk4	0.215	0.021	-2.12	-1.00
Gsto2	0.517	0.111	-1.85	-1.00
Mup9	7.079	2.226	-1.53	-1.01
Tmprss11a	0.117	0.000	-2.45	-1.01
Ncan	0.114	0.015	-1.95	-1.01
Hcn4	0.098	0.012	-2.07	-1.01
Slco1c1	0.061	0.000	-2.49	-1.02
Chil3	0.702	0.158	-1.78	-1.02
Pzp	0.048	0.000	-2.45	-1.03
Elavl2	0.191	0.034	-1.82	-1.03
Gm3716	0.073	0.000	-2.48	-1.03
Cacna2d3	0.685	0.167	-1.62	-1.03
E330033B04Rik	0.174	0.043	-1.76	-1.04
Ascl1	0.455	0.078	-1.81	-1.04
Ces1b	0.454	0.078	-1.82	-1.05
Mir6398	2.478	0.000	-2.45	-1.05
Fam83e	0.933	0.068	-1.88	-1.05
Cox7a1	35.404	8.911	-1.53	-1.05

Ces1e	0.192	0.011	-2.29	-1.05
Mup2	10.885	3.488	-1.52	-1.06
Gm13177	0.163	0.000	-2.50	-1.06
Mep1a	0.248	0.000	-2.61	-1.06
Ugt2b35	0.083	0.000	-2.63	-1.07
Hnf1b	0.156	0.000	-2.53	-1.07
C330024C12Rik	0.270	0.032	-2.16	-1.07
Mup12	7.352	2.249	-1.61	-1.07
Pcp4	1.199	0.069	-2.18	-1.08
Pla2g2a	0.725	0.000	-2.56	-1.08
Mup1	6.734	2.044	-1.61	-1.08
Pyy	1.544	0.000	-2.69	-1.09
Car1	2.507	0.194	-1.85	-1.09
Ms4a8a	3.155	0.081	-2.02	-1.09
4932415M13Rik	0.074	0.000	-2.54	-1.10
Mup14	11.206	3.308	-1.59	-1.10
Foxl2os	0.209	0.006	-2.38	-1.11
Unc5cl	0.104	0.000	-2.56	-1.11
Ptgdr	0.285	0.035	-2.10	-1.11
Mup15	10.366	3.160	-1.65	-1.12
Mug2	0.065	0.000	-2.67	-1.12
Rnf186	0.574	0.000	-2.79	-1.12
Trim67	0.107	0.025	-1.86	-1.12
Tbx21	0.164	0.017	-2.17	-1.12
Ppp1r3fos	0.152	0.026	-2.02	-1.13
BC021891	0.071	0.008	-2.23	-1.13
LOC100048884	11.541	3.503	-1.60	-1.13
Btbd17	0.212	0.029	-2.16	-1.13
Mup11	10.324	2.872	-1.67	-1.13
Crmp1	0.584	0.154	-1.78	-1.13
A230028O05Rik	0.087	0.000	-2.65	-1.14
Gsta4	6.875	1.449	-1.67	-1.14

Slc6a7	0.111	0.000	-2.63	-1.15
Anks4b	0.305	0.000	-2.58	-1.16
Gm24148	5.121	0.000	-2.67	-1.16
Hoxa13	0.588	0.000	-2.63	-1.16
Insl5	0.881	0.000	-2.56	-1.17
Sall1	0.586	0.017	-2.08	-1.17
Cyp4a10	0.268	0.032	-2.24	-1.17
Vax1	0.671	0.022	-2.29	-1.18
Kng1	0.106	0.000	-2.66	-1.18
D630033O11Rik	0.674	0.077	-2.28	-1.18
Snora33	19.407	5.247	-1.80	-1.19
Mogat1	0.655	0.091	-2.12	-1.20
Icam4	0.273	0.000	-2.74	-1.20
Entpd8	0.452	0.010	-2.40	-1.20
Guca2a	0.979	0.000	-2.68	-1.21
Fcgbp	3.304	0.064	-1.86	-1.21
B3gnt7	3.481	0.289	-1.80	-1.21
Pklr	0.203	0.000	-2.73	-1.22
Otop1	0.294	0.021	-2.28	-1.22
Cdhr2	0.245	0.000	-2.85	-1.22
Tusc1	60.346	4.055	-1.70	-1.22
Clca4b	0.201	0.000	-2.81	-1.23
Mup8	7.422	2.034	-1.74	-1.23
Cyp3a11	0.361	0.000	-2.74	-1.23
Pak7	0.097	0.009	-2.34	-1.24
Gm1123	0.487	0.000	-2.75	-1.25
Foxf2	2.425	0.065	-2.13	-1.25
Crybb3	0.955	0.186	-2.07	-1.26
Necab3	0.627	0.122	-2.07	-1.26
Ush1c	0.249	0.000	-2.82	-1.27
Sult1d1	0.265	0.000	-2.71	-1.27
Mrgprb8	0.474	0.059	-2.30	-1.27
Gm10549	0.087	0.000	-2.69	-1.28
Phox2b	0.227	0.000	-2.70	-1.28
Serpina1d	2.662	0.397	-1.89	-1.30

Nlrp6	0.127	0.000	-2.90	-1.30
Fendrr	0.464	0.007	-2.55	-1.31
Tmem45b	5.411	0.672	-1.91	-1.31
Ppara	3.831	0.919	-1.79	-1.33
Vip	0.366	0.000	-2.78	-1.33
Hoxd12	0.666	0.017	-2.38	-1.34
Hoxa11os	2.397	0.108	-2.12	-1.34
Slc6a14	0.777	0.090	-2.04	-1.35
Klk1	11.785	0.679	-1.92	-1.35
Trim31	0.179	0.000	-2.86	-1.35
Tcp10c	0.119	0.000	-2.88	-1.35
Ces1g	0.641	0.067	-2.17	-1.36
Mup3	0.735	0.049	-2.48	-1.38
Sult4a1	0.196	0.018	-2.47	-1.38
Hand2	2.112	0.335	-1.94	-1.39
Mup16	7.073	1.576	-1.99	-1.39
Sucnr1	0.739	0.084	-2.30	-1.40
Foxl2	0.414	0.000	-2.81	-1.40
Colec10	0.108	0.000	-2.99	-1.42
Spdef	0.672	0.048	-2.35	-1.42
Rimklb	0.274	0.011	-2.52	-1.42
Sp9	0.256	0.016	-2.53	-1.42
Elavl4	0.153	0.015	-2.39	-1.42
Clrn3	0.396	0.000	-2.81	-1.43
Adgrg7	0.313	0.000	-2.84	-1.43
Il17b	1.266	0.159	-2.38	-1.43
Gldn	12.529	3.256	-1.90	-1.43
Sult1b1	0.405	0.000	-2.97	-1.43
Htr3a	0.232	0.000	-2.71	-1.43
Tcp10a	0.148	0.009	-2.71	-1.43
Isl2	0.476	0.000	-2.83	-1.44
Cldn7	5.447	0.288	-2.10	-1.47
Ihh	0.552	0.018	-2.56	-1.47
AI506816	0.760	0.135	-2.19	-1.48
Gpx2	1.895	0.083	-2.46	-1.48
Gdap1l1	0.102	0.000	-2.86	-1.50

H2-M10.1	0.264	0.000	-2.87	-1.51
Hnf4a	0.918	0.040	-2.35	-1.51
Gm9994	0.862	0.056	-2.56	-1.51
Snord55	15.027	2.543	-2.31	-1.52
Grin1os	0.100	0.000	-3.04	-1.52
Muc4	0.077	0.004	-2.60	-1.52
Cidea	32.209	3.124	-2.03	-1.53
Grm7	0.148	0.000	-2.98	-1.54
Aoc1	1.234	0.015	-2.56	-1.54
Ankrd34a	15.444	3.579	-2.02	-1.54
Gcnt3	0.411	0.000	-3.24	-1.54
Hao2	0.516	0.000	-3.01	-1.54
Dnajc22	0.221	0.000	-2.93	-1.55
Hpse2	0.630	0.020	-2.68	-1.57
Snord34	53.278	6.344	-2.31	-1.60
Mup5	0.351	0.000	-3.08	-1.61
B230209E15Rik	1.800	0.364	-2.22	-1.62
Aqp8	0.481	0.000	-3.08	-1.63
Pigr	0.483	0.017	-2.66	-1.63
Tcp10b	0.203	0.011	-2.81	-1.64
Ugt2b34	1.237	0.014	-2.76	-1.65
Ttr	0.890	0.036	-2.77	-1.66
Tm4sf20	0.891	0.000	-3.14	-1.67
Gsc	0.898	0.038	-2.82	-1.67
Serpina1c	3.345	0.402	-2.25	-1.68
Fut9	0.065	0.000	-3.23	-1.68
BC030870	0.876	0.000	-3.23	-1.69
Cyp2c68	0.577	0.000	-3.15	-1.72
Clec2h	2.280	0.010	-3.08	-1.72
Krt84	0.408	0.034	-2.61	-1.72
Serpina1b	5.260	0.510	-2.30	-1.72
Cyp2u1	0.208	0.009	-2.86	-1.73
1700028M03Rik	0.954	0.000	-3.11	-1.73
Serpina1a	2.424	0.284	-2.35	-1.73

Six2	1.150	0.031	-2.68	-1.74
Pla2g10	1.218	0.000	-3.29	-1.75
Shisa8	0.188	0.000	-3.09	-1.79
Mptx2	3.091	0.000	-3.25	-1.80
Gm5475	0.176	0.000	-3.22	-1.81
Adad2	0.714	0.000	-3.42	-1.82
Sprr3	2.279	0.000	-3.44	-1.86
Gucy2c	0.306	0.006	-3.18	-1.87
Psca	5.641	0.051	-2.97	-1.89
Phgr1	4.442	0.000	-3.43	-1.89
Prss32	4.018	0.030	-3.00	-1.91
Cd177	0.192	0.000	-3.42	-1.91
Slc15a2	2.811	0.384	-2.44	-1.92
Tcf21	2.034	0.000	-3.37	-1.93
Mug1	0.172	0.000	-3.36	-1.96
Birc7	3.277	0.000	-3.55	-1.98
Gm10024	0.705	0.000	-3.46	-2.00
Reg4	4.613	0.000	-3.48	-2.01
Aqp5	0.759	0.043	-2.96	-2.01
Gm10548	0.581	0.067	-2.81	-2.03
9530053A07Rik	0.384	0.006	-3.22	-2.06
Cdx1	2.464	0.000	-3.78	-2.09
Sftpd	2.311	0.000	-3.69	-2.22
Mup4	0.823	0.025	-3.34	-2.22
Hoxb13	3.509	0.000	-3.70	-2.23
Hoxd13	2.211	0.000	-3.79	-2.40
Cckar	0.419	0.015	-3.49	-2.41
Muc2	5.098	0.016	-3.45	-2.42
Sval1	16.609	0.000	-3.98	-2.50
Krt9	1.202	0.009	-3.77	-2.53
Tspan1	4.905	0.022	-3.59	-2.55
Csn3	0.754	0.000	-3.98	-2.56
Slc51a	2.055	0.000	-4.04	-2.60
Cryba2	55.634	0.092	-3.65	-2.64
Muc13	6.729	0.015	-3.86	-2.68

Pmp2	37.554	0.048	-3.67	-2.68
Dmbt1	7.064	0.007	-3.89	-2.70
Spink4	21.042	0.000	-3.99	-2.70
Gpa33	2.535	0.000	-4.09	-2.71
Ccl22	4.195	0.073	-3.63	-2.74
Ucp1	17.376	0.080	-3.54	-2.75
Mptx1	18.105	0.000	-4.26	-2.77
Foxa1	0.849	0.000	-4.29	-2.81
Agr2	21.479	0.000	-4.37	-2.82
Sspo	1.059	0.098	-3.35	-2.83
Glrp1	14.092	1.250	-3.43	-2.94
Spink1	118.122	0.052	-4.27	-2.97
Hoxa11	1.967	0.029	-3.85	-2.99
D130043K22Rik	0.679	0.004	-4.17	-3.05
Tff3	41.135	0.000	-4.51	-3.05
Srrm3	1.198	0.050	-3.90	-3.12
Hoxd11	0.608	0.000	-4.72	-3.21
Clca1	16.539	0.008	-4.52	-3.32
Ces1c	0.857	0.000	-4.83	-3.44
Lypd8	51.637	0.000	-5.02	-3.55

Log2FC: log2-fold change

GFOLD (0.1): conservatively estimated log2-fold change at q = 0.1 confidence level

IFNg: HALLMARK_INTERFERON_GAMMA_RESPONSE

NFKB: HALLMARK_TNFA_SIGNALING_VIA_NFKB
